# Cancer care under systemic shock: utilization declines and cost increases during the COVID-19 pandemic in Colombia, evidence from matched administrative cohorts

**DOI:** 10.1186/s13690-026-01922-2

**Published:** 2026-05-28

**Authors:** Daniel Medina-Gaspar, Valeria Bejarano, Luis Orozco, Meisser Madera, Paul Rodríguez, Ricardo Bruges, Sara Atehortúa, Giancarlo Romano

**Affiliations:** 1 Universidad EAFIT, Medellín, Colombia; 2 National University of Colombia, Bogotá, Colombia; 3 School of Economics, Universidad de Antioquia, Medellín, Colombia; 4 Instituto de Evaluación Tecnológica en Salud (IETS), Bogotá, Colombia; 5 Universidad del Rosario, Bogotá, Colombia; 6 Pontificia Universidad Javeriana, Bogotá, Colombia; 7 Faculty of Dentistry, Universidad de Cartagena, Cartagena, Colombia

**Keywords:** Covid-19, Cancer-care, Impact evaluation, Health care utilization, Health care utilization costs, Pandemic impact

## Abstract

**Background:**

The COVID-19 pandemic, declared by WHO in March 2020, has had a significant impact on global health systems, including cancer care in Colombia. This study examines the impact of the pandemic on service delivery, health outcomes, and risk management for breast, cervical, colorectal, stomach, prostate, and lung cancers.

**Methods:**

Using administrative data, we applied propensity score matching for individual-level to compare pre- and post-pandemic cohorts from 2017 to 2021, alongside with a fixed effect model to state-level covering 2018 to 2021.

**Results:**

Our findings reveal a consistent decline in cancer care services, except for a notable increase in the likelihood of receiving palliative care consultations and chemotherapy for breast, cervical, and colorectal cancers. When examining the intensive margin, measured by the frequency of use per patient, the results indicate an increase in both consultations and chemotherapy, reflecting higher spending on these services and a rise in average total expenditure per patient for most cancers studied. At the state level, similar trends were observed, with rising cancer prevalence and increased mortality rates for most cancers, except stomach cancer.

**Conclusions:**

These findings highlight the urgent need for a resilient health system capable of mitigating the impact of future health crises on chronic disease management, and particularly in cancer.

**Supplementary Information:**

The online version contains supplementary material available at 10.1186/s13690-026-01922-2.

**Table:** 

Text box 1. Contributions to the literature
• Provides one of the first quantitative evaluations of how COVID-19 affected cancer care in a middle-income country using linked administrative data.
• Shows that systemic shocks may reduce service volumes while increasing treatment intensity and per-patient costs.
• Demonstrates how combining patient-level and regional analyses can strengthen evidence on health system resilience.
• Highlights the importance of protecting chronic disease pathways and cancer registries during public health emergencies.

## Introduction

The COVID-19 pandemic triggered an unprecedented health crisis, widely regarded as the most significant global public health emergency in recent history [1]. Its effects have persisted through 2024 and are expected to continue shaping the coming years, driving a profound transformation across various sectors of society. Global authorities have focused their efforts on containing and mitigating the virus's spread, implementing measures such as border closures, social distancing, selective isolation, and quarantine [2]. The healthcare sector, in particular, has faced numerous challenges, adapting to maintain patient care by prioritizing urgent cases while scaling back on diagnosis, control, and prevention efforts [3]. Additionally, many patients with other illnesses, such as cancer, delayed seeking care, largely due to fears of COVID-19 infection [4]. Rather than a single-channel shock, COVID-19 generated simultaneous disruptions on both the supply and demand sides of the health system. These included the reallocation of health-care personnel and infrastructure toward COVID-19 care, temporary reductions in provider capacity, changes in referral pathways, behavioral responses to perceived risk, and broader socioeconomic stresses affecting care-seeking.

This paper analyses the effects of the COVID-19 pandemic on cancer care in Colombia. Specifically, we study how the pandemic has affected health services utilization, health outcomes and risk management in breast, cervical, colorectal, stomach, prostate and lung cancers. This was done by tracking individuals based on multiple administrative data sources and aggregated data at the departmental level (like the state level in the United States).

Two methods were used. First, we used propensity score matching (PSM) to compare two cohorts (pre-COVID and post-COVID) and thus estimate the effects of the pandemic compared to a pre-pandemic scenario. The pre-COVID cohort (control group) was constructed with information from cancer patients before the pandemic from 2017 to 2019, and the post-COVID cohort (treatment group) corresponds to patients exposed to the pandemic conditions between 2020 and 2021.

Second, since there was no access to health outcome information at the individual level, we estimated changes in health outcomes (mortality, incidence, and prevalence) and cancer risk management by a state-level panel model from 2018–2021. Combining these approaches allows us to enrich our findings on service utilization with insights into health outcomes and management.

This article contributes to the literature by providing the first evidence in Colombia on outcome changes for six cancers during the COVID-19 pandemic. To our knowledge, we have created a unique individual-level database linking clinical and demographic characteristics with health service delivery variables, which has not been done before. Additionally, by using multiple sources, we have identified key health service codes associated with cancer, enabling the construction of highly specialized variables. Finally, this study employs multiple approaches that complement the analysis and enhance the robustness of findings for a complex type of pathology influenced by numerous factors.

The rest of the paper is organized as follows. Section "Background" describes the data used. Section "Empirical strategy" explains the data preparation and the construction of key variables for the empirical analysis. Section "Data" details the empirical strategy. Section "Variables construction" presents the descriptive statistics of the variables used and the balance tests for the PSM model. Section "Description of the samples for analysis" presents the results. Section "Results" discusses the findings. Finally, Section "Discussion" provides conclusions.

## Background

Our study aligns with global and regional evidence documenting how COVID-19-related restrictions disrupted diagnosis, management, and outcomes in oncology patients. Worldwide, lockdowns led to delays in diagnostic, control, and preventive services, with variable consequences for clinical outcomes [5–12]. A systematic review concluded that the pandemic likely caused delays in skin cancer diagnosis and treatment [10], while other reviews suggest that for some cancers—such as lung cancer—the decline in screening and diagnostic testing did not immediately translate into worse short-term clinical outcomes [11]. Similar patterns have been documented for melanoma and keratinocyte carcinomas, where diagnostic delays did not substantially worsen tumor characteristics [8, 9].

In Latin America, the impact has been even more pronounced. A multi-country assessment of eight LAC nations documented severe disruptions to routine health services during the pandemic and highlighted the heterogeneous institutional capacity to respond [12]. Studies from Chile also reveal substantial inequities: vulnerable populations faced significant reductions in access to cancer care [13], and gender disparities widened for time-sensitive conditions [14]. A recent analysis of South America found shifts in the place of death among cancer patients during the pandemic, indicating the profound effects of overburdened health systems [15]. More broadly, regional syntheses show reductions of 28–100% in diagnostic and treatment activities, including mammography, PAP smears, cancer surgeries, chemotherapy, and first consults [16]. These findings underscore the need for analyses that quantify the health and economic implications of service deferral and care reconfiguration in the post-pandemic period.

In Colombia, the pandemic also stressed the health system in unprecedented ways. For instance, hospital bed capacity underwent rapid expansion and reorganization during COVID-19, as shown by interrupted time-series evidence documenting structural changes in system readiness [17]. Understanding this context is essential because the Colombian healthcare system is characterized by managed competition between insurers (HMO), who administer the health benefits plan for their enrolled population. Cancer care is financed through the same scheme, with clinical information and treatment histories captured in the CAC registry and service-level transactions captured in the Suficiencia database [6, 7, 18]. These data systems differ in purpose and completeness: CAC focuses on clinical follow-up and risk-adjustment monitoring, while Suficiencia captures claims and expenditures for affiliates in the contributory regime.

Cancer services in Colombia are delivered by a mix of public and private providers accredited in the REPS [12], and clinical pathways involve multiple interactions—including diagnosis, staging, early detection programs, oncology consultations, therapies, and follow-up—which generate the administrative footprints used in this study. However, the system’s decentralization and the heterogeneity in provider capacity mean that disruptions can produce substantial variation across regions, insurers, and patient groups. Providing this institutional background clarifies the origin and structure of the administrative data employed in our analysis and helps interpret the effects observed in service utilization and health outcomes.

**G**iven this context, our work contributes by quantifying changes in cancer-related service use and population-level indicators during the pandemic, using two complementary analytic strategies—PSM at the individual level and fixed-effects models at the departmental level—to capture both micro- and macro-level disruptions in Colombia’s cancer care system [5, 19].

## Empirical strategy

Our analysis follows two distinct strategies. First, to assess the causal impact of the COVID-19 Pandemic on utilization measures of cancer care services we use a PSM using individual level data. Second, we used a two-way linear panel analysis to assess changes in population-level health outcomes. The aggregate time shocks let us capture the COVID shock conditional on fixed overtime differences across states.

### Propensity score matching for individual-level estimations

In this study, we employ a propensity score matching (PSM) strategy for the period spanning the onset of the COVID-19 pandemic, following the methodological approach proposed by Webb et al. [20] and implemented using STATA psmatch2 [21]. Individuals are matched at two distinct time points to construct comparable pre-pandemic and pandemic-period cohorts. The analysis combines two administrative data sources: the oncology records from the Cuenta de Alto Costo (CAC, 2017–2019), which provide detailed clinical and demographic information, and the Suficiencia-UPC database (2017–2021), which records health service utilization and expenditures for a subset of health maintenance organizations (EPS). This linkage allows us to use pre-COVID confounders from CAC and post-COVID outcomes from Suficiencia in a consistent way.

**Fig. 1:**
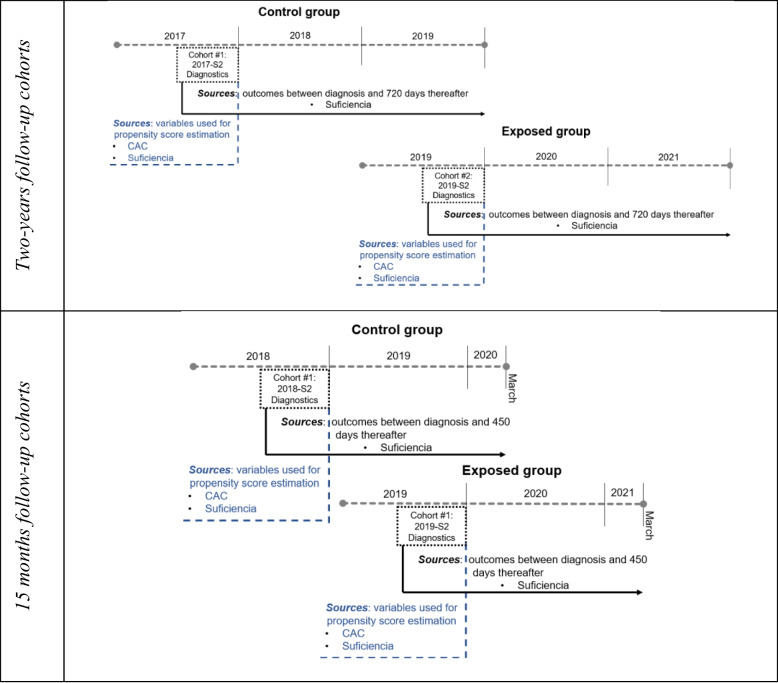
Definition of pre- and post-COVID cohorts for cancer patients in Colombia, individual-level administrative data, 2017–2021. Notes: The figure illustrates the construction of control (pre-COVID) and exposed (post-COVID) cohorts using administrative data from the Cuenta de Alto Costo (CAC) and Suficiencia databases in Colombia. Two cohort definitions are used: (i) two-year follow-up cohorts (2YC), with outcomes measured up to 720 days after diagnosis; and (ii) fifteen-month follow-up cohorts (15MC), with outcomes measured up to 450 days after diagnosis. Control cohorts include patients diagnosed before March 2020, while exposed cohorts include patients diagnosed prior to the pandemic but followed during the COVID-19 period (2020–2021). Propensity score matching variables are derived from CAC and Suficiencia data

As summarized in Fig. 1, we constructed two groups of cohorts for evaluation. First, the two-year follow-up cohorts (2YC): the cohort from the second half of 2017 (control or pre-COVID-19 cohort) was followed for up to two years, ending in December 2019, while the cohort from the second half of 2019 (exposed or post-COVID-19 cohort) was tracked for up to two years, concluding in December 2021. Individuals in the exposed cohort were therefore subjected to the COVID-19 impact for a minimum of 16 months (for diagnoses in July 2019) and a maximum of 20 months (for diagnoses in December 2019). Second, the fifteen-month follow-up cohorts (15MC): the cohort from the second half of 2018 (control or pre-COVID-19 cohort) was followed for up to 15 months, ending in March 2020, while the cohort from the second half of 2019 (exposed or post-COVID-19 cohort) was tracked for the same period, concluding in March 2021. In this case, individuals in the exposed cohort experienced the COVID-19 impact for between 7 months (diagnoses in July 2019) and 12 months (diagnoses in December 2019).

The propensity score was estimated using a logistic regression model including age, sex, cancer type, insurance regime, and baseline service utilization measures. These covariates capture observable risk composition and enrolment conditions that influence access to oncology services. We intentionally excluded provider-level characteristics from the matching procedure. Changes in provider assignment and contractual arrangements formed part of the health-system adjustment during the pandemic, and conditioning on these variables would constrain the very mechanisms through which COVID-19 affected care delivery and could bias the estimated effects toward zero.

Matching was implemented using nearest-neighbor matching with five neighbors (NN-5). This specification provided a good compromise between bias reduction and sample retention and performed well relative to alternative matching algorithms. Common support was assessed graphically and through overlap diagnostics, and post-matching balance was evaluated using standardized mean differences and variance ratios. All covariates met conventional balance thresholds (standardized mean difference < 0.1), indicating that the matched samples were well balanced across observable characteristics.

The estimate of interest is the average treatment effect on the treated (ATT), which captures the change experienced by patients diagnosed before the pandemic when exposed to pandemic-period conditions. This matching strategy ensures that comparisons across the 2YC and 15MC cohorts reflect differences attributable to pandemic-related disruptions rather than shifts in observable patient composition.

### Fixed effect model to state-level estimations

We employed a fixed effects model at the state (means department) *(i)* and year *(t)* levels to evaluate the impact of the COVID-19 pandemic on health outcomes in cancer and risk management indicators, the dependent variable $({y}_{it})$. Our dataset spans from 2018 to 2021, capturing both pre- and post-pandemic periods. The model specification is as follows:

$$\begin{aligned}{y}_{it}=&\alpha +{\beta }_{1}Pos{t}_{2020}+\beta {X}_{it}+{\delta }_{i}\\&+{\gamma }_{2019}+{\gamma }_{2020}+{\gamma }_{2021}+{\varepsilon }_{it}\end{aligned}$$

where *α* is a constant term, $Pos{t}_{2020}$ is a dummy variable indicating the post-pandemic period (taking the value of 1 for years 2020 and later, and 0 otherwise), ${X}_{it}$ is a vector of control variables, *β* represents the associated vector of coefficients, ${\delta }_{i}$ denotes department fixed effects, ${\gamma }_{t}$ captures year fixed effects, and ${\varepsilon }_{it}$ is the error term. The coefficient ${\beta }_{1}$ quantifies the average change in the outcome variable attributable to the pandemic.

## Data

### Individual-level data

At the individual level, we employed two databases to track cancer patients, gathering their demographic characteristics and health service utilization. The first database, from the CAC [18], covers the period from 2017 to 2019 and contains clinical and demographic information, including cancer type, date of diagnosis, and prior cancer history, sourced from the National Cancer Information System. The second database, the Suficiencia database [22], spans 2017 to 2021 and registers the information of HMO that manage the provision of the health benefits plan of around 60% of the patients with capacity to pay. To preserve the consistency along the analysis, we selected the five HMOs that are always in the database for the reference period of the study. This database provides details on the costs of health services rendered, along with anonymized patient characteristics (such as identification, age, and sex), service attributes (type, scope, institution code, etc.), and technology (identified by a unique identification code).

### State-level data

Due to data restrictions, we do not have information to evaluate health outcomes at the individual-level. For this reason, we complemented the analysis at the individual-level with state-level analysis using a panel database for the period 2018–2021 by combining various data sources:

- CAC Annexes: for each cancer analyzed, indicators for mortality, prevalence, new cases and the specific risk management (the latter, except for lung cancer) [23].
- Departamento Administrativo Nacional de Estadística (DANE) Multidimensional Poverty Annexes: Barriers to access to health services and multidimensional poverty indicators [24].
- DANE National Accounts: Gross domestic product (GDP) per capita [25].
- DANE Vital Statistics: the mortality data for each cancer analyzed [26].
- Registro Especial de Prestadores de Servicios de Salud—(REPS): Data on the installed capacity of providers that offered services associated with malignant tumor diagnoses [27].

## Variables construction

### Individual-level data

We constructed five variables of interest to measure individual usage and costs linked to cancer-related technologies. These variables were constructed using a four-stage process.

First, we selected individuals who were likely to have one of the cancers under analysis based on the health services that they demanded, identified by their corresponding International Classification of Diseases (ICD) version 10 code (Annex 1).

Second, we constructed a short list of the principal health services that are directly associated with cancer care, which was done by reviewing literature such as clinical practice guidelines [17, 28–31], Asociación Colombiana de Hematología y Oncología -ACHO- [32], CAC [33], US National Cancer Institute [34] and Global Burden of Diseases [35]. The list was then reviewed by the expert oncologists and epidemiologists of the team.

Third, we grouped the medical procedures into: consultations (surgical, palliative care, follow-up and radiology), follow-up and control (dissemination, diagnostic, laboratory and imaging tests), hospitalization, therapies (monotherapy, polytherapy and teletherapy), and other specific procedures according to the type of cancer. We use the identifiers of the procedures and medications for their selection and extraction (Annex 2).

Fourth, for each procedure, we built three types of outcome variables: i) whether the patient receives at least one procedure; ii) the frequency of procedures; and iii) the payment made by HMO for the procedure (as a proxy for change in expenditure). These variables are assessed over a maximum range of care time (720 days or 450, depending on the court). We computed costs using standardized values per procedure derived from the average reported expenditure for such a procedure in the dataset (in the given year.

To determine if a given procedure is linked to the specific cancer, we defined a specific time window determine by the date of diagnosis, we created a dummy variable ${P}_{itjn}$ that takes the value of 1 if the n-th medical procedure *j* in the year t was conducted for individual *i* after the diagnosis date (${T}_{diag, i}$) and within 720 days (or 450 days, depending on the cohort) from the diagnosis date, and 0 otherwise:

$${P}_{itjn}=\left\{\begin{array}{c}1, if ({T}_{jtin}>{T}_{diag, i})\wedge {T}_{jtin}\le \left({T}_{diag, i}+cohort days\right).\\ 0, otherwise\end{array}\right.$$

To evaluate changes in expenditure over time, we constructed a measure for each procedure using the available information on each patient and deflating it with the Consumer Price Index (CPI) of each year. Then, we calculated two standardized measures of real expenditure for all patients. The first measure is the median of real expenditure per procedure for each year t, capturing annual variations in real costs:

$$\begin{aligned}{Exp1}_{tj}=&Median\Big({\{Exp}_{1,tj,1},{Exp}_{1,tj,2},\dots ,\\&{Exp}_{2,tj,1},{Exp}_{1,tj,2},\dots ,{Exp}_{i,t,j,n}\}\Big).\end{aligned}$$

The second measure is the median real expenditure per procedure across all the years observed, which does not account for these annual changes:

$$\begin{aligned}{Exp2}_{j}=&Median\Big({\{Exp}_{\mathrm{1,2017},j,1},{Exp}_{\mathrm{1,2017},j,2},\dots ,\\&{Exp}_{\mathrm{2,2017},j,1},{Exp}_{2,2017,j,2}, \dots ,{Exp}_{i,t,j,n}\}\Big).\end{aligned}$$

With these measures we constructed the five variables of interest as follows:

 1 st measure: patients have at least one treatment or procedure *j* between the date of diagnosis and 720 days (or 450) thereafter

$${M1}_{ij}=\left\{\begin{array}{l}1, if {\sum }_{t=1}^{y}{\sum }_{n=1}^{m}{P}_{itjn}>0.\\ 0, otherwise\end{array}\right.$$

2nd measure: frequencies of treatment and procedure j between the date of diagnosis and 720 days (or 450) thereafte*r*

$${M2}_{ji}={\sum }_{t=1}^{y}{\sum }_{n=1}^{m}{P}_{itjn}.$$

3rd measure: real expenditure of treatment and procedure *j* between the date of diagnosis and 720 days (or 450) thereafter

$$\begin{array}{c}{M3a}_{ji}=\sum\nolimits_{t=1}^y\sum\nolimits_{n=1}^m{Exp1}_{jt}\ast P_{itjn},\\{M3b}_i=\sum\nolimits_{t=1}^y\sum\nolimits_{n=1}^m{Exp2}_j\ast P_{itjn}.\end{array}$$

Finally, we calculated variables for total patient expenditure for all services provided in the system and the procedures specific to the cancer analyzed.

4th measure: total real expenditure for all treatments and procedures owns of each cancer of patient *i* between the date of diagnosis and 720 days (or 450) thereafter

$$\begin{array}{c}{M4a}_i=\sum\nolimits_{t=1}^y\sum\nolimits_{j=1}^l\sum\nolimits_{n=1}^m{Exp1}_{jt}\ast P_{itjn},\\{M4b}_i=\sum\nolimits_{t=1}^y\sum\nolimits_{j=1}^l\sum\nolimits_{n=1}^m{Exp2}_j\ast P_{itjn}.\end{array}$$

$${M4b}_{i}={\sum }_{t=1}^{y}{\sum }_{j=1}^{l}{\sum }_{n=1}^{m}{Exp2}_{j}*{P}_{itjn}.$$

5th measure: total real expenditure for all health services of patient *i* between the date of diagnosis and 720 days (or 450) thereafter

$${M5a}_{i}={\sum }_{t=1}^{y}{\sum }_{k=1}^{h}{\sum }_{n=1}^{m}{Exp1}_{kt}*{P}_{itkn},$$

$${M5b}_{i}={\sum }_{t=1}^{y}{\sum }_{k=1}^{h}{\sum }_{n=1}^{m}{Exp2}_{k}*{P}_{itkn}.$$

### State-level data

We used four indicators to explore the impact on health outcomes at the state-level for each type of cancer in people affiliated to the health system: mortality rate per 100,000 people (enrolled in the health system) with data from CAC and DANE, incidence rate per 100,000 people, prevalence rate per 100,000 people, and risk management indicators. Mortality data in Colombia may differ from one source to another. These differences arise from variations in data collection methodologies and sources. The DANE relies on death certificates registered through the Ministry of health system, which may have delays or underreporting in certain areas. In contrast, the CAC aggregates data from HMO and other health entities, potentially offering more timely and specific information but possibly missing cases outside the formal healthcare system.

Likewise, we defined the cancer-type specific indicators. For breast cancer, we assess the timeliness of cancer care in days, the percentage of women diagnosed with advanced-stage cancer, and the lethality rate for advanced-stage cancer. For cervical cancer, we look at the timeliness of cancer care in days, the percentage of women with early-stage cancer receiving curative treatment, and the percentage of women with advanced-stage cancer receiving chemoradiation and brachytherapy. For colorectal and stomach cancers, we measure the timeliness of treatment in days, the percentage of new cases identified at early stages, and the percentage of Stage I-III patients undergoing curative surgery. For prostate cancer, we evaluate the timeliness of diagnosis and treatment in days, and the percentage of patients with advanced stages (III-IV).

## Description of the samples for analysis

### Individual-level data

Table 1 presents the variables and their categories, as well as indicates which variables were used for each type of cancer. Using this set of variables, we estimated the propensity score employing a five-nearest-neighbor (NN-5) approach. Given the extensive nature of the descriptive statistics tables, which include balance tests before and after matching for each propensity score variable, these tables are provided in Annex 3. Generally, for all cancers analyzed within each cohort group, the standardized bias significantly decreases after matching, with most variables achieving acceptable levels below 10%. However, specific age groups and regions show standardized biases exceeding 10%−20% for cervical, colorectal, and prostate cancers. For stomach and lung cancers, due to the smaller number of observations, more variables exhibit standardized differences above 10%.

**Table 1:** Pre-treatment variables used for propensity score estimation in six cancer types in Colombia, individual-level administrative data, 2017–2019

Variable	Options	Colorectal	Cervical	Stomach	Breast	Prostate	Lung
Sex	i) Male ii) Female	Yes	No	Yes	No	No	Yes
Cancer stage	In situ to IV-V	Yes	Yes	Yes	Yes	Yes	Yes
Medical intervention objective	i) Pre-treatment observation ii) Curative or palliative treatment iii) Follow-up after initial treatment	Yes	Yes	No	Yes	Yes	No
Treatment objective	i) Curative ii) Palliative	Yes	Yes	Yes	Yes	Yes	Yes
Gaps days between diagnostic and appointment with oncologist	i) < 16 days ii) 15–30 days iii) + 30 days	Yes	Yes	Yes	Yes	Yes	Yes
HER2 tumor marker tests	i) Her2-Positive ii) Her2-borderline iii) Her2-negative	No	No	No	Yes	No	No
Age at diagnosis	Age groups in five-year ranges up to age 74, and then 74 +	Yes (+ 50)	Yes (+ 30)	Yes (+ 45)	Yes (+ 30)	Yes (+ 50)	Yes (+ 50)
EPS (insurer company)	5 EPS options of the contributory scheme	Yes	Yes	Yes	Yes	Yes	Yes
Residence region	Antioquia, Bogotá, Valle del Cauca, North states, Center States, Southwest States and Southeast States	Yes	Yes	Yes	Yes	Yes	Yes
Lineal distance to nearest main city from residence (km)	i) < 20 km ii) 20–59 km iii) 60–179 km iv) + 179 km	Yes	Yes	Yes	Yes	Yes	Yes
Quarter within cohort at diagnostic	Quarter III Quater IV	Yes	Yes	Yes	Yes	Yes	Yes

This table reports pre-diagnosis and pre-treatment characteristics used to estimate the propensity score for each cancer type in Colombia (cohorts 2017-S2, 2018-S2, 2019-S2; see Fig. 1). The unit of observation is the individual patient. Variables are obtained from the Cuenta de Alto Costo (CAC) registry, which provides clinical and demographic information prior to the COVID-19 pandemic, and Suficiencia, which presents healthcare usage information. Linear distance to the nearest main city was constructed using Google Maps. Age categories begin at the threshold indicated in parentheses and were defined based on the distribution of cases within each cancer type. EPS refers to Entidad Promotora de Salud (insurance provider) in the contributory regime. Regional categories correspond to standard geographic groupings used in the Colombian health system

After balancing (Annex 4), we observed that for most cancers, we had more than 270 treated and 250 untreated individuals, resulting in over 500 observations in the common support. However, stomach and lung cancers had fewer observations, with fewer than 200 treated and untreated individuals, consistent with their lower incidence rates. We analyzed new cases from 2019 in the CAC reports and found that the number of treated individuals on common support in our estimations was relatively high in relation to these new cases: 65% for breast cancer and 70% for cervical cancer, while other cancers had treated individuals ranging from 39 to 48%. We used half of the new cases as a reference point, given that our analysis only considered the second semester of 2019 in 2-years cohort. In all cases, the number of people on treatment was greater than the number of people off treatment, so estimation with replacement was necessary. Despite this, the tests suggest a good balance among matched individuals.

### State-level data

Table 2 presents the descriptive statistics of the variables used in the state-level panel data model. Generally, most indicators show an increase after 2020, except for mortality rates in stomach and lung cancer according to DANE Vital Statistics data; early-stage cases also decreased for colorectal and stomach cancers. The dispersion around the mean of the indicators reveals significant regional heterogeneity, which diminishes in the most variables after 2020. Most indicators exhibit minimum values of 0, corresponding to states with relatively few and young inhabitants (Amazonas, Guainía, Guaviare, San Andrés, Vaupés, and Vichada) that presented few cases. These states are excluded from the estimates.

**Table 2:** Descriptive statistics of cancer outcomes before and after COVID-19 in Colombia, state-level panel data, 2018–2021

	Pre-2020					Post-2020				
Variable	N	Mean	S.D	Min	Max	N	Mean	S.D	Min	Max
*Breast*										
CAC mortality rate—x100k affiliates	66	6.99	4.21	0.00	15.28	66	11.03	6.27	0.00	26.33
VS mortality rate—x100k affiliates	60	11.20	5.92	0.79	24.24	63	11.77	5.80	2.20	24.48
New case reported rate—x100k affiliates	66	13.85	10.08	0.00	43.51	66	17.05	10.23	0.00	39.44
Prevalence rate- x100k affiliates	66	155.81	103.27	4.52	374.46	66	212.94	129.31	4.69	471.40
Timeliness of cancer care (days)	61	69.54	16.07	26.00	117.42	63	75.01	21.67	7.00	169.00
Women diagnosed with advanced-stage (%)	61	57.08	18.91	0.00	100.00	64	61.05	18.69	0.00	100.00
Fatality rate advanced-stage (%)	62	6.30	2.73	0.00	12.07	64	8.45	3.79	0.00	25.42
*Cervical*										
CAC mortality rate—x100k affiliates	66	3.81	1.96	0.00	9.03	66	6.23	5.63	0.00	42.12
VS mortality rate—x100k affiliates	65	6.66	2.96	1.18	13.90	63	7.32	3.15	0.72	18.76
New case reported rate—x100k affiliates	66	5.80	2.83	0.00	14.03	66	6.46	4.81	0.00	37.52
Prevalence rate- x100k affiliates	66	49.49	18.20	4.74	80.01	66	73.56	72.75	4.92	479.78
Timeliness of cancer care (days)	59	78.14	21.26	21.00	138.00	58	77.66	22.58	0.00	145.00
Women with early-stage receiving curative treatment (%)	55	31.99	25.64	0.00	100.00	52	54.17	27.18	0.00	100.00
Women with advanced-stage receiving chemoradiotherapy with brachytherapy (%)	59	3.94	7.29	0.00	40.00	58	24.64	26.20	0.00	100.00
*Colorectal*										
CAC mortality rate—x100k affiliates	66	2.50	1.90	0.00	7.72	66	3.81	2.40	0.00	9.62
VS mortality rate—x100k affiliates	62	5.84	3.50	0.56	14.36	62	6.08	3.52	0.92	15.19
New case reported rate—x100k affiliates	66	3.47	2.65	0.00	10.30	66	4.35	3.10	0.00	12.16
Prevalence rate- x100k affiliates	66	24.00	17.39	0.00	59.65	66	33.36	22.58	0.89	82.40
Timeliness of treatment (days)	58	51.20	23.71	0.00	111.00	62	68.30	43.77	2.00	323.00
New cases identified at early stages (%)	59	43.71	17.40	0.00	100.00	59	29.12	17.01	0.00	100.00
Stage I-III patients with curative surgery (%)	59	30.12	20.63	0.00	100.00	58	43.83	20.57	0.00	100.00
*Stomach*										
CAC mortality rate—x100k affiliates	66	2.61	1.98	0.00	8.28	66	3.97	2.89	0.00	10.12
VS mortality rate—x100k affiliates	61	9.21	5.20	0.77	18.60	63	8.88	5.42	0.89	21.23
New case reported rate—x100k affiliates	65	2.72	2.09	0.00	9.21	66	3.20	2.45	0.00	10.29
Prevalence rate- x100k affiliates	66	11.99	9.37	0.00	32.53	66	18.45	15.21	0.00	62.02
Timeliness of treatment (days)	57	42.24	24.36	0.00	137.00	57	51.75	17.84	11.00	101.50
New cases identified at early stages (%)	58	27.43	18.03	0.00	100.00	59	15.31	15.24	0.00	100.00
Stage 0-III patients with curative surgery (%)	51	35.02	21.78	0.00	100.00	55	51.13	27.51	0.00	100.00
*Prostate*										
CAC mortality rate—x100k affiliates	66	4.90	3.24	0.00	12.82	66	8.63	5.44	0.00	21.93
VS mortality rate—x100k affiliates	63	11.20	4.79	1.66	19.84	61	12.37	4.56	2.22	23.20
New case reported rate—x100k affiliates	66	7.62	6.09	0.00	33.60	66	8.48	6.25	0.00	24.16
Prevalence rate- x100k affiliates	66	83.45	57.44	0.00	197.07	66	119.75	79.84	0.00	306.69
Timeliness of diagnosis (days)	58	63.43	34.60	0.00	161.00	56	64.91	24.80	0.00	164.54
Timeliness of treatment (days)	57	68.49	32.07	0.00	248.00	56	93.96	31.78	9.00	250.00
Patients with advanced-stages (III-IV) (%)	60	40.26	23.62	0.00	100.00	58	43.91	19.55	0.00	100.00
*Lung*										
CAC mortality rate—x100k affiliates	66	1.43	1.22	0.00	6.23	66	2.19	1.77	0.00	8.45
VS mortality rate—x100k affiliates	62	7.34	4.39	0.78	19.43	60	7.14	4.56	0.90	24.36
New case reported rate—x100k affiliates	66	1.35	1.21	0.00	5.72	66	1.73	1.57	0.00	7.43
Prevalence rate- x100k affiliates	66	4.55	3.74	0.00	17.15	66	6.03	4.70	0.00	23.51

This table reports descriptive statistics for state-level cancer indicators in Colombia using panel data for the period 2018–2021. The unit of observation is a state-year. “Pre-2020” corresponds to the pre-COVID period (2018–2019), while “Post-2020” corresponds to the COVID period (2020–2021). Mortality rates from CAC are expressed per 100,000 affiliates, whereas mortality rates from Vital Statistics (VS) are expressed per 100,000 inhabitants. Incidence, prevalence, and stage-related indicators follow CAC definitions. “Timeliness” refers to the average number of days from diagnosis to treatment (or care initiation), depending on the cancer type. Differences in sample sizes reflect missing observations in administrative sources. These descriptive differences are formally evaluated using fixed-effects models in Table 4

## Results

### Propensity score matching

In general terms, for most outcomes in each cancer type, we find no significant effects of the COVID-19 emergency on changes in the provision of health services, beyond a few specific services for each cancer type. Our approach allows us to examine both the extensive and intensive margins of service utilization. For the cohort with a longer time span, the gap is approximately 24 months, while the other cohort has a 15-month gap. These cohorts were matched using clinical and demographic variables. Although this does not guarantee perfect identification, the size of the exposure and the short time span of the cohorts provide evidence of the pandemic's impact on cancer management and outcomes.

Table 3 summarizes the most relevant statistically significant estimates, while the full set appears in Annex 5. The table reports ATT values (post-COVID minus matched pre-COVID), their standard errors and the mean potential outcome for the matched pre-COVID cohort. To guide interpretation, consider radiology consultations for breast cancer. In the 15MC cohort, the ATT is not statistically significant (ATT = − 0.003), implying no detectable difference in the probability of receiving at least one radiology consultation. In contrast, in the 2YC cohort the ATT equals 0.083, meaning that patients diagnosed in the post-COVID period were 8.3 percentage points more likely to receive at least one radiology consultation, relative to a baseline of 55.6% (P.O. mean = 0.556).

**Table 3:** Propensity score–matched ATT estimates of the impact of COVID-19 on cancer service use and costs in Colombia, individual-level administrative data, cohorts 2017–2021

	Cohort 2 years follow-up −2YC				Cohort 15 months follow-up −15MC			
Outcomes	ATT		S.E	P.O. mean	ATT		S.E	P.O. mean
*Breast*								
Radiology consultation (binary)	0.083	***	0.018	0.556	−0.003		0.028	0.575
Follow-up consultation (binary)	0.094	***	0.003	0.798	0.032		0.021	0.817
Quadrantectomy (binary)	−0.044	**	0.021	0.213	−0.053	**	0.022	0.212
Screening (binary)	0.061	***	0.023	0.245	0.014		0.023	0.251
Laboratory tests (binary)	0.038	***	0.004	0.837	0.031		0.022	0.816
Monotherapy (binary)	0.108	***	0.017	0.279	0.073	***	0.023	0.226
Teletherapy (binary)	0.151	***	0.021	0.520	−0.021		0.028	0.600
Radiotherapy (binary)	0.115	***	0.011	0.719	0.025		0.024	0.789
Surgical consultation Frequency	0.925	***	0.127	1.500	0.072		0.134	1.652
Palliative care consultation Frequency	0.169	***	0.057	0.308	0.049	*	0.029	0.174
Radiology consultation Frequency	0.149	***	0.034	0.886	0.021		0.044	0.730
Follow-up consultation Frequency	4.056	***	0.287	4.214	1.164	***	0.218	4.286
Quadrantectomy Frequency	−0.051	*	0.027	0.248	−0.060	**	0.026	0.246
Spread procedures Frequency	8.675	***	1.151	19.823	2.424	***	0.876	17.793
Mammography Frequency	0.434	***	0.103	2.312	0.065		0.082	1.364
Monotherapy Frequency	0.722	***	0.173	1.217	0.243	***	0.082	0.622
Teletherapy Frequency	0.195	***	0.037	0.663	−0.079	*	0.040	0.787
Radiotherapy Frequency	1.025	***	0.323	6.056	−0.438	*	0.266	5.839
Emergency consultations frequency	−0.549	***	0.122	1.685	−0.255	***	0.097	1.074
Tamoxifen Rx frequency	0.967	***	0.291	4.585	0.087		0.177	2.246
Trastuzumab Rx frequency	−0.096		0.222	0.713	0.200	***	0.075	0.221
Surgical consultation real expenditure (stand.)	59,173	***	12,346	82,058	5,859		13,862	103,953
Follow-up consultation real expenditure (stand.)	212,280	***	19,783	290,150	22,504		18,510	313,260
Quadrantectomy real expenditure (stand.)	40,706		49,341	299,260	−297,345	***	92,136	625,934
Screening real expenditure (stand.)	95,330	***	35,418	192,930	39,634		30,022	201,732
Spread procedures real expenditure (stand.)	656,250	***	121,028	1,400,198	44,951		106,752	1,557,350
Diagnostic imaging real expenditure (stand.)	195,506	***	55,520	531,808	72,951	*	42,784	395,452
Laboratory tests real expenditure (stand.)	874,927	***	115,902	1,112,654	398,750	***	139,757	1,369,888
Mammography real expenditure (stand.)	54,915	***	18,283	240,522	14,822		16,458	183,356
Monotherapy real expenditure (stand.)	1,352,626	**	620,093	2,043,669	762,523	**	313,444	841,087
Teletherapy real expenditure (stand.)	1,961,307	***	320,439	4,405,748	−624,967		380,292	5,688,960
Radiotherapy real expenditure (stand.)	3,413,199	***	1,228,250	13,911,487	−1,609,676		1,021,826	14,805,536
Tamoxifen Rx real expenditure per person (stand.)	45,687	***	4,217	26,695	15,198	***	2,463	16,517
Trastuzumab Rx real expenditure per person (stand.)	−815,097		1,492,437	4,828,376	1,446,411	***	489,186	1,352,424
Real expenditure per person (stand.)	4,599,719	***	1,700,235	24,856,782	1,859,456	**	941,007	21,167,384
Cancer care real expenditure per person (stand., procedures only)	3,701,119	***	465,265	12,142,053	−312,897		507,395	14,073,674
Cancer care real expenditure per person (stand., drugs only)	−988,492		1,283,670	4,860,572	1,149,288	***	423,741	1,579,307
*Colorectal*								
Colectomy Frequency	0.119	***	0.045	0.197	0.058		0.041	0.244
Surgical consultation Frequency	1.152	***	0.168	0.782	0.608	***	0.118	0.691
Palliative care consultation Frequency	0.261	***	0.092	0.395	0.040		0.054	0.331
Follow-up consultation Frequency	1.728	***	0.479	3.574	0.490	*	0.266	2.786
Spread procedures Frequency	7.191	***	2.742	27.216	−2.598		1.744	24.397
Laboratory tests Frequency	0.958	*	0.570	6.252	−0.732	**	0.369	5.138
Emergency consultations frequency	−0.811	***	0.305	2.607	−0.150		0.157	1.582
Home care frequency	1.329	***	0.404	1.152	0.188		0.345	1.471
Colectomy real expenditure (stand.)	1,522,159	***	514,472	1,560,796	371,961		611,236	2,693,722
Colonoscopy real expenditure (stand.)	112,898	**	55,862	330,907	152,662	*	82,507	255,150
Surgical consultation real expenditure (stand.)	71,197	***	10,941	53,327	34,445	***	8,634	51,637
Diagnostic imaging real expenditure (stand.)	772,115	***	154,335	1,062,946	97,607		149,830	1,021,480
Laboratory tests real expenditure (stand.)	172,503	***	46,036	418,435	−32,779		42,470	429,272
Teletherapy real expenditure (stand.)	1,374,331	***	370,706	878,769	460,257		336,783	1,585,750
Morphine Rx real expenditure per person (stand.)	9,519	**	4,460	6,310	8,186	***	3,035	2,110
Cancer care real expenditure per person (stand., procedures only)	2,602,779	***	951,704	9,737,771	−101,742		713,564	10,562,701
*Cervical*								
Conization (binary)	0.086	**	0.035	0.104	0.063	**	0.028	0.127
Surgical consultation (binary)	0.030	***	0.010	0.002	0.012		0.009	0.006
Palliative care consultation (binary)	0.085	***	0.028	0.087	0.028		0.028	0.119
Hysterectomy (binary)	−0.041	***	0.010	0.238	0.106	***	0.034	0.088
Teletherapy (binary)	0.219	***	0.034	0.154	−0.029		0.038	0.395
Brachytherapy Frequency	−0.267	*	0.145	0.607	−0.169	**	0.073	0.505
Conization Frequency	0.104	***	0.039	0.111	0.082	***	0.032	0.133
Palliative care consultation Frequency	0.241	**	0.103	0.189	0.153	**	0.063	0.138
Follow-up consultation Frequency	1.394	***	0.324	2.778	0.562	***	0.191	2.259
Spread procedures Frequency	2.389	***	0.891	9.855	0.491		0.694	7.667
Hysterectomy Frequency	−0.041	***	0.010	0.238	0.106	***	0.034	0.088
Teletherapy Frequency	0.256	***	0.039	0.164	−0.015		0.048	0.420
Morphine Rx frequency	0.166	**	0.081	0.160	0.116	**	0.056	0.103
Conization real expenditure (stand.)	−36,794		69,098	194,411	70,640	***	24,021	86,977
Surgical consultation real expenditure (stand.)	3,247	***	1,099	47	975		808	628
Follow-up consultation real expenditure (stand.)	96,245	***	20,813	152,933	32,767	**	16,158	144,459
Spread procedures real expenditure (stand.)	449,543	***	136,340	540,812	234,247	**	106,531	450,410
Hysterectomy real expenditure (stand.)	−213,948	***	60,281	718,676	270,904	**	107,175	229,025
Diagnostic imaging real expenditure (stand.)	364,594	***	109,292	432,393	−194,209	*	110,491	700,242
Teletherapy real expenditure (stand.)	2,183,906	***	370,244	1,180,533	−496,169		574,804	3,687,344
Radiotherapy real expenditure (stand.)	2,817,990	***	598,901	3,742,538	−681,141		837,805	6,713,717
Morfina Rx real expenditure per person (stand.)	7,816	**	3,143	2,819	5,251	*	2,694	2,523
Real expenditure per person (stand., procedures only)	2,684,024	***	969,216	9,204,985	−250,855		841,703	10,342,414
Cancer care real expenditure per person (stand.)	2,436,406	***	724,044	6,599,643	−105,150		736,287	8,330,842
Cancer care real expenditure per person (stand., procedures only)	2,476,947	***	707,200	6,463,004	−61,895		715,915	8,247,908
*Lung*								
Inpatient services (binary)	0.036		0.064	0.647	0.194	***	0.069	0.423
Follow-up consultation Frequency	3.133	***	0.800	2.566	1.236	**	0.566	2.530
Follow-up consultation real expenditure (stand.)	170,153	***	38,636	140,126	83,368	***	27,202	133,984
Diagnostic imaging real expenditure (stand.)	1,374,718	***	295,545	732,831	677,286	**	284,000	895,800
*Prostate*								
Brachytherapy (binary)	0.033	***	0.012	0.010	0.037	***	0.010	0.003
Monotherapy (binary)	−0.064	*	0.034	0.316	0.062	**	0.027	0.138
Brachytherapy Frequency	0.034	***	0.012	0.011	0.039	***	0.011	0.003
Follow-up consultation Frequency	0.486	**	0.242	1.239	0.315	**	0.135	0.728
Monotherapy Frequency	−0.119		0.157	1.310	0.439	***	0.099	0.284
Polytherapy Frequency	−0.338	**	0.155	0.780	−0.160	*	0.094	0.517
Radiotherapy Frequency	−0.441	*	0.227	2.659	0.319	**	0.145	1.264
Brachytherapy real expenditure (stand.)	342,723	***	104,102	27,913	361,643	***	103,414	2,282
Monotherapy real expenditure (stand.)	393,177	***	135,615	429,463	403,981	***	90,023	107,600
Teletherapy real expenditure (stand.)	1,319,341	***	472,848	3,221,937	238,522		450,127	3,559,950
Radiotherapy real expenditure (stand.)	2,225,969	***	582,426	4,243,561	946,323		591,005	4,333,519
*Stomach*								
Surgical consultation (binary)	0.118	**	0.060	0.247	0.118	**	0.048	0.230
Follow-up consultation (binary)	−0.113	***	0.044	0.838	−0.087	**	0.041	0.776
Gastrectomy (binary)	−0.021		0.053	0.311	0.101	***	0.039	0.183
Follow-up consultation Frequency	1.497	***	0.476	2.843	−0.020		0.390	3.283
Gastrectomy Frequency	0.029		0.059	0.331	0.151	***	0.051	0.191
Emergency consultations frequency	−0.959	**	0.379	2.964	−0.562	*	0.288	2.288
Surgical consultation real expenditure (stand.)	25,735	***	5,783	20,320	18,929	***	5,220	19,012
Laboratory tests real expenditure (stand.)	63,584	***	23,250	72,150	−17,687		56,672	135,105

Each row reports the estimated Average Treatment Effect on the Treated (ATT) comparing patients diagnosed before the COVID-19 pandemic and exposed to pandemic-period conditions with matched pre-pandemic patients in Colombia. Estimates are presented separately for two-year (2YC) and fifteen-month (15MC) follow-up cohorts constructed from administrative data spanning 2017–2021. The “P.O. mean” corresponds to the mean potential outcome for the matched pre-COVID cohort. Matching was performed using nearest-neighbor propensity score matching with replacement. Robust standard errors are reported in parentheses. Statistical significance is indicated as follows: * *p* < 0.10, ** *p* < 0.05, *** *p* < 0.01. Estimates were obtained using the STATA module psmatch2 (Leuven and Sianesi, 2018)

Overall, the pattern that emerges from Table 3 is one of heterogeneous and cancer-specific adjustments in service use, rather than a uniform disruption. The short-run cohort (15MC) shows few and inconsistent effects, consistent with the immediate contraction of services during the strictest phase of the pandemic. In contrast, the two-year cohort (2YC) displays clearer signals of care reorganization and accumulated demand.

#### Extensive margin

Changes in the probability of receiving at least one service are relatively limited but meaningful in specific cancers. The most consistent increases occur in breast, cervical and stomach cancer for selected diagnostic and treatment events, while lung cancer shows a short-run increase in inpatient care. The fact that extensive-margin effects are sparse in the short-run but emerge more clearly with longer follow-up suggests that patients eventually returned to the system, but often with greater needs or delayed presentations, rather than simply resuming pre-pandemic patterns of care.

#### Intensive margin

In contrast, the intensive margin shows stronger and more systematic increases across several cancers. Frequencies of follow-up consultations, palliative care, surgical encounters and spread-related procedures rise in breast, cervical and colorectal cancers, reflecting more resource-intensive care per patient. These increases are consistent with two mechanisms: (i) case worsening due to diagnostic and treatment delays; and (ii) restructuring of care pathways, where providers concentrated multiple services within fewer contacts or relied more on modalities such as teletherapy or radiotherapy. Together, these results support the idea that the pandemic did not merely reduce service use but altered the intensity and composition of care, particularly in cancers where timely treatment is critical.

#### Total patient expenditures

Expenditure patterns mirror these findings. Increases are most pronounced in follow-up care, diagnostic imaging, radiotherapy and teletherapy, with several cancers exhibiting substantially higher per-patient spending in the post-COVID cohort. These rises are consistent with more complex clinical presentations, greater reliance on technology-intensive treatments and shifts toward modalities less affected by supply constraints during the pandemic. Importantly, the expenditure increases are not uniform across cancers or services: rather, they reflect targeted pressure points in the care continuum, which helps explain the uneven but meaningful changes in intensive-margin use. These results provide quantitative evidence of the economic consequences of delayed or reconfigured cancer care, themes revisited in the Discussion.

### State-level model

Table 4 presents the state-level fixed-effects estimates for all cancers; the corresponding models including the full set of controls are reported in Annex 5. To illustrate how to interpret these coefficients, we use the case of breast cancer mortality in column (1). This means that, after accounting for state and year fixed effects, the breast cancer mortality rate recorded in the CAC registry was 5.09 deaths per 100,000 affiliates higher in the post-COVID period relative to the pre-COVID years. Given a sample mean of 11.68, this reflects a substantial relative increase. Column (2) reports the corresponding mortality rate based on vital statistics (VS), yielding an estimated increase of 3.05 deaths per 100,000 inhabitants in the post-COVID period. Both estimates are statistically significant and indicate that the rise in breast cancer mortality observed after the onset of the pandemic is consistently captured across the two independent data sources (CAC and VS).

**Table 4:** State-level fixed-effects estimates of the association between the COVID-19 period and cancer outcomes in Colombia, state-level panel data 2018–2021

	Health outcomes								CAC Risk-management outcomes					
	(1)	(2)	(3)	(4)	(5)	(6)	(7)	(8)	(9)	(10)	(11)	(12)	(13)	(14)
Breast														
Variables	Mortality rate—CAC		Mortality rate—VS		Incidence rate		Prevalence rate		Timeliness of cancer care (days)		Women diagnosed with advanced-stage (%)		Fatality rate advanced-stage (%)	
Post-Covid indicator	5.09***	3.05***	1.03***	0.64	−0.16	−5.04**	85.33***	62.73***	0.88	2.10	5.59***	−0.73	1.05**	1.41
	(0.68)	(1.02)	(0.34)	(0.76)	(0.91)	(1.91)	(7.51)	(9.71)	(2.40)	(5.61)	(1.82)	(4.59)	(0.41)	(0.96)
Observations	108	108	108	108	108	108	108	108	108	108	108	108	108	108
R-squared	0.777	0.810	0.095	0.150	0.553	0.635	0.844	0.868	0.090	0.143	0.154	0.246	0.168	0.221
Number of Units	27	27	27	27	27	27	27	27	27	27	27	27	27	27
State FE	Yes	Yes	Yes	Yes	Yes	Yes	Yes	Yes	Yes	Yes	Yes	Yes	Yes	Yes
Year FE	Yes	Yes	Yes	Yes	Yes	Yes	Yes	Yes	Yes	Yes	Yes	Yes	Yes	Yes
Controls	No	Yes	No	Yes	No	Yes	No	Yes	No	Yes	No	Yes	No	Yes
Sample mean	11.68	11.68	14.42	14.42	22.80	22.80	265.1	265.1	69.61	69.61	54.16	54.16	7.296	7.296
**Cervical**														
Variables	Mortality rate—CAC		Mortality rate—VS		Incidence rate		Prevalence rate		Timeliness of treatment (days)		Women with early-stage receiving curative treatment (%)		Women with advanced-stage receiving chemoradiation and brachytherapy (%)	
Post-Covid indicator	1.88***	2.36***	0.42	0.44	−0.98**	−0.98	14.72***	13.28***	−5.25	−4.54	18.02***	29.61***	39.05***	35.83***
	(0.28)	(0.66)	(0.26)	(0.45)	(0.37)	(0.94)	(1.63)	(3.14)	(3.55)	(7.61)	(5.14)	(9.70)	(2.73)	(4.83)
Observations	108	108	108	108	108	108	108	108	108	108	100	100	107	107
R-squared	0.523	0.550	0.039	0.120	0.362	0.385	0.774	0.781	0.111	0.168	0.265	0.334	0.874	0.881
Number of Units	27	27	27	27	27	27	27	27	27	27	27	27	27	27
State FE	Yes	Yes	Yes	Yes	Yes	Yes	Yes	Yes	Yes	Yes	Yes	Yes	Yes	Yes
Year FE	Yes	Yes	Yes	Yes	Yes	Yes	Yes	Yes	Yes	Yes	Yes	Yes	Yes	Yes
Controls	No	Yes	No	Yes	No	Yes	No	Yes	No	Yes	No	Yes	No	Yes
Sample mean	4.647	4.647	7.136	7.136	6.604	6.604	64.66	64.66	76.66	76.66	46.52	46.52	14.49	14.49
**Colorectal**	Mortality rate—CAC		Mortality rate—VS		Incidence rate		Prevalence rate		Timeliness of treatment (days)		New cases identified at early stages (%)		Stage I-III patients with curative surgery (%)	
Post-Covid indicator	2.05***	1.26***	0.28	0.13	0.13	−1.34**	15.30***	11.51***	17.42***	12.63**	−17.77***	−26.99***	16.18***	17.50**
	(0.21)	(0.30)	(0.17)	(0.50)	(0.36)	(0.59)	(1.98)	(1.90)	(1.97)	(5.05)	(2.73)	(5.93)	(3.01)	(7.72)
Observations	108	108	108	108	108	108	108	108	108	108	108	108	108	108
R-squared	0.733	0.805	0.057	0.082	0.450	0.547	0.799	0.873	0.543	0.573	0.478	0.507	0.360	0.367
Number of Units	27	27	27	27	27	27	27	27	27	27	27	27	27	27
State FE	Yes	Yes	Yes	Yes	Yes	Yes	Yes	Yes	Yes	Yes	Yes	Yes	Yes	Yes
Year FE	Yes	Yes	Yes	Yes	Yes	Yes	Yes	Yes	Yes	Yes	Yes	Yes	Yes	Yes
Controls	No	Yes	No	Yes	No	Yes	No	Yes	No	Yes	No	Yes	No	Yes
Sample mean	4.644	4.644	8.076	8.076	6.012	6.012	43.68	43.68	56.04	56.04	35.85	35.85	40.24	40.24
**Prostate**														
Variables	Mortality rate—CAC		Mortality rate—VS		Incidence rate		Prevalence rate		Timeliness of diagnosis (days)		Timeliness of treatment (days)		Patients with advanced-stages (III-IV) (%)	
Post-Covid indicator	6.92***	5.05***	1.13***	−0.10	−1.57*	−3.87***	66.90***	41.19***	−1.83	−3.06	32.75***	51.02***	10.32***	8.81
	(0.74)	(0.95)	(0.38)	(0.65)	(0.81)	(1.34)	(8.97)	(7.39)	(4.19)	(13.48)	(3.42)	(9.91)	(3.20)	(6.83)
Observations	108	108	108	108	104	104	108	108	104	104	105	105	106	106
R-squared	0.821	0.890	0.226	0.369	0.538	0.594	0.808	0.852	0.135	0.146	0.461	0.539	0.179	0.198
Number of Units	27	27	27	27	26	26	27	27	27	27	27	27	27	27
State FE	Yes	Yes	Yes	Yes	Yes	Yes	Yes	Yes	Yes	Yes	Yes	Yes	Yes	Yes
Year FE	Yes	Yes	Yes	Yes	Yes	Yes	Yes	Yes	Yes	Yes	Yes	Yes	Yes	Yes
Controls	No	Yes	No	Yes	No	Yes	No	Yes	No	Yes	No	Yes	No	Yes
Sample mean	9.191	9.191	14.03	14.03	12.47	12.47	154.5	154.5	66.01	66.01	81.05	81.05	36.43	36.43
**Stomach**	Mortality rate—CAC		Mortality rate—VS		Incidence rate		Prevalence rate		Timeliness of treatment (days)		New cases identified at early stages (%)			
Post-Covid indicator	1.44***	0.51	−0.12	−0.37	−0.05	−1.18**	8.30***	7.59***	19.37***	11.15*	−14.76***	−15.43***		
	(0.19)	(0.32)	(0.31)	(0.81)	(0.26)	(0.44)	(1.31)	(2.42)	(2.57)	(6.27)	(1.88)	(4.39)		
Observations	108	108	108	108	108	108	108	108	108	108	108	108		
R-squared	0.553	0.641	0.012	0.019	0.411	0.506	0.540	0.559	0.537	0.561	0.464	0.490		
Number of Units	27	27	27	27	27	27	27	27	27	27	27	27		
State FE	Yes	Yes	Yes	Yes	Yes	Yes	Yes	Yes	Yes	Yes	Yes	Yes		
Year FE	Yes	Yes	Yes	Yes	Yes	Yes	Yes	Yes	Yes	Yes	Yes	Yes		
Controls	No	Yes	No	Yes	No	Yes	No	Yes	No	Yes	No	Yes		
Sample mean	4.267	4.267	10.75	10.75	4.057	4.057	20.25	20.25	48.75	48.75	23.11	23.11		
**Lung**	Mortality rate—CAC		Mortality rate—VS		Incidence rate		Prevalence rate							
Post-Covid indicator	0.87***	0.41*	−0.98***	−0.62	0.04	−0.48*	2.10***	1.26**						
	(0.23)	(0.23)	(0.20)	(0.40)	(0.23)	(0.23)	(0.63)	(0.57)						
Observations	104	104	104	104	104	104	108	108						
R-squared	0.499	0.713	0.186	0.206	0.342	0.632	0.572	0.683						
Number of Units	26	26	26	26	26	26	27	27						
State FE	Yes	Yes	Yes	Yes	Yes	Yes	Yes	Yes						
Year FE	Yes	Yes	Yes	Yes	Yes	Yes	Yes	Yes						
Controls	No	Yes	No	Yes	No	Yes	No	Yes						
Sample mean	2.757	2.757	9.211	9.211	2.461	2.461	8.447	8.447						

Each column reports the estimated association between a post-COVID indicator (equal to 1 for years 2020–2021 and 0 for 2018–2019) and cancer outcomes in Colombia using state-level panel data. The unit of observation is a state-year. Models include state and year fixed effects, and columns alternate between specifications without and with additional controls. Outcomes include mortality (Cuenta de Alto Costo, CAC, and Vital Statistics, VS), incidence, prevalence, timeliness of care, early detection, stage distribution, and treatment indicators. Coefficients represent absolute changes relative to the pre-COVID period. Sample means correspond to the pre-COVID period. Robust standard errors are reported in parentheses. Statistical significance is indicated as follows: * *p* < 0.10, ** *p* < 0.05, *** *p* < 0.01

#### Health outcomes

A consistent pattern across cancers is that CAC-based mortality and prevalence indicators tend to increase, while the VS registry shows more moderate or null effects for mortality. Incidence rates generally show small negative or null changes, but these must be interpreted cautiously because several cancers (e.g., colorectal, stomach, lung) exhibit a downward shift in 2019, captured by the year fixed effect in all models. As a result, incidence and prevalence coefficients partly reflect both pandemic-related changes and pre-existing trends, rather than purely COVID-19 effects.

#### Mortality: CAC vs Vital Statistics

Increases in mortality are more pronounced in CAC than in VS across nearly all cancers. For breast, cervical, colorectal, prostate and lung cancer, CAC-based mortality rises between roughly 1.4 and 6.9 deaths per 100,000, whereas VS-based mortality increases are generally smaller and, in some cancers, not significant. This divergence likely reflects differences in registry coverage and reporting practices: VS is less sensitive to short-term shocks in access to cancer care and may capture excess mortality from COVID-19 more cleanly, while CAC mortality, being linked to service provision and reporting, may be more reactive to disruptions in care pathways and follow-up. The consistent direction of effects across both sources, however, suggests a genuine deterioration in mortality outcomes, even if magnitudes differ.

#### Risk-management indicators

Several indicators of care quality and timeliness show evidence of deterioration during the pandemic. Across cervical, colorectal, prostate and stomach cancers, we observe longer treatment delays, even after accounting for state-level fixed characteristics and national time shocks. Early detection declines in colorectal and stomach cancers, while the share of patients receiving timely curative surgery also falls in colorectal cancer. At the same time, the probability of presenting with advanced-stage disease or receiving treatment for advanced disease (e.g., chemoradiation, brachytherapy) tends to rise in multiple cancers.

## Discussion

This study examined how the COVID-19 pandemic affected cancer care in Colombia by combining individual-level and state-level analyses for six cancers. Using PSM with NN-5 on linked CAC and HMO Suficiencia data, we compared pre- and post-COVID cohorts to study changes in service use, expenditure and selected clinical outcomes. In parallel, we estimated state-level fixed-effects models with CAC and DANE indicators, enriched with contextual information from VS, GDP and REPS, to capture changes in mortality, incidence, prevalence and risk-management indicators. Taken together, these approaches provide a coherent picture of how the pandemic reshaped both the volume and the intensity of oncology care.

At the individual level, the main message is not a uniform collapse of cancer care, but a redistribution of when and how patients were treated. We find relatively few and cancer-specific changes in the extensive margin, with modest increases in the probability of receiving certain diagnostic or treatment services, particularly in breast, cervical and stomach cancers, and a short-run rise in inpatient care for lung cancer. In contrast, the intensive margin shows clearer and more systematic increases in the frequency of follow-up consultations, palliative care, surgical encounters, spread-related procedures and radiotherapy or teletherapy, especially for breast, cervical and colorectal cancers. These patterns are consistent with patients returning to the system with more advanced needs after delays in diagnosis or treatment, and with providers reorganizing care by concentrating multiple services into fewer but more intense episodes. The expenditure results mirror this picture: higher per-patient spending is concentrated in follow-up, diagnostic imaging and high-cost treatments, which is compatible with both greater clinical complexity and a reconfiguration of care pathways during and after the pandemic.

The state-level analyses complement these findings by documenting changes in population-level health outcomes and risk-management indicators. Across most cancers, CAC-based mortality and prevalence rates increase in the post-COVID period, while VS-based mortality shows smaller and sometimes non-significant changes. Incidence rates tend to decline or remain flat, although this must be interpreted cautiously because several cancers show a downward shift already in 2019, captured by the year fixed effects. The divergence between CAC and VS mortality probably reflects differences in registry coverage, in how cancer and COVID-19 deaths are coded, and in how sensitive each source is to disruptions in access and follow-up. Risk-management indicators point towards deterioration in several dimensions: longer treatment delays for cervical, colorectal, prostate and stomach cancers, lower early detection in colorectal and stomach cancers, and higher probabilities of advanced-stage presentation or treatment for advanced disease. Although some of these indicators are volatile, the overall pattern is consistent with the individual-level evidence of delayed diagnosis and more intensive care once patients re-entered the system.

These results should be read alongside several important limitations. First, even though we exploited detailed clinical information and geographic variables in the CAC data for the PSM, the microdata made available to us was rich, but it is limited to basic demographic characteristics and the register of hospital capacity reported by hospitals. As a result, we could not condition on time-varying provider capacity due to availability of oncology workforce or local congestion; this is particularly relevant in a setting where oncology services were competing with COVID-19 care. At the state level, we partly address this by including contextual controls and fixed effects, but residual confounding by unobserved supply shocks cannot be ruled out. Second, we modelled the pandemic as a single post-2020 shock. In reality, COVID-19 arrived in waves and peak intensity varied across states and over time. We do not observe reliable state-level series of COVID-19 cases, hospitalizations or ICU occupancy linked to our cancer indicators, so we cannot formally distinguish the effects of high-intensity waves from those of later, milder phases. Future work using time-varying measures of epidemic intensity or approaches that contrast states with different COVID burdens, such as quantile-type comparisons by epidemic severity, could provide additional robustness. A third limitation concerns the use of CAC indicators for incidence and prevalence. CAC is not a population-based cancer registry, and its newly reported cases capture only a fraction of the true number of incident cancers. Comparisons with GLOBOCAN suggest substantial under-ascertainment that varies by cancer type, which means that CAC-based incidence and prevalence should be interpreted as measures of detected and reported cases within the insured population rather than as estimates of population incidence or prevalence. Moreover, because CAC is a passive registry fed by HMO reporting, it was itself affected by the same disruptions in service use that we study. This is especially relevant for the post-pandemic cohort, where changes in CAC indicators may conflate true changes in disease burden with changes in detection, coding and reporting. For these reasons, we place greater weight on the direction and internal consistency of patterns across sources and outcomes than on the absolute levels of CAC incidence and prevalence.

Additional constraints arise from the structure of our data. The individual-level analysis relies on five HMO in the contributory regime, covering around 60% of affiliates in that scheme and excluding the subsidized regime. This introduces a selection towards formally employed populations and insurers that tend to have better cancer-management indicators, limiting external validity. Socioeconomic variables are not observed at the individual level, so they could not be included in the PSM despite their importance for prevention and timely care. Our cohorts include only newly diagnosed cases to avoid overlap between control and exposed groups, which, together with relatively low case counts for stomach and lung cancer, leads to limited sample sizes and reduced statistical power for some outcomes. The construction of expenditure variables required standardizing payments reported in the Suficiencia database, which reflect HMO contracts rather than underlying economic costs, and medication codes were sometimes incomplete, particularly for the longer follow-up cohort. All these factors mean that our estimates should be interpreted as indicative of patterns and mechanisms rather than as precise measures of causal effects or levels.

Despite these limitations, the study has several strengths that are uncommon in the region. It makes systematic use of administrative data sources that are often analyzed in isolation, linking CAC, HMO Suficiencia, DANE and REPS information to follow cancer patients over time and to place individual-level changes in a broader population context. It applies PSM and state-level fixed-effects models to six high-priority cancers, using NN-5 matching to construct comparable pre- and post-COVID cohorts and state-level models that jointly consider mortality, incidence, prevalence and risk-management indicators. It does so in a setting where oncology care was already under pressure before the pandemic and in which formal, quantitative assessments of COVID-19 impacts have been scarce. The convergence between individual-level and state-level findings, and between service-use, expenditure and outcome indicators, strengthens the internal consistency of our conclusions.

From a policy perspective, our results underscore that the main risk of a shock such as COVID-19 is not only an immediate reduction in service volumes, but the combination of delayed detection, more advanced disease at presentation and more intensive, costly care for those who eventually reach the system. Health systems that rely on administrative data like those from CAC and HMO must anticipate that both care and registries will be disrupted in future crises, and should invest in maintaining at least minimal diagnostic, follow-up and reporting capabilities during emergencies. Planning for reserve capacity in oncology, protecting critical diagnostic and treatment pathways, and expanding the use of telemedicine and decentralized diagnostics could help reduce the build-up of undetected or undertreated cancer cases. More broadly, the experience documented here highlights the importance of strengthening cancer information systems and routinely linking administrative data sources, so that policymakers can monitor not only the direct effects of a shock like COVID-19, but also its indirect impact on chronic conditions whose consequences may unfold over many years.

## Conclusion

This study significantly contributes to the existing literature by providing empirical evidence on the pandemic’s impact on cancer care in a developing country. The disruptions observed in 2020 should not be attributed solely to mobility restrictions. Rather, they reflect a multifaceted shock combining supply-side constraints, behavioural responses to perceived risk, reallocation of health-care resources, and broader socioeconomic pressures. Understanding these interacting mechanisms is essential for interpreting the patterns we document and for designing policy responses that can mitigate similar crises in the future.

The decline in overall demand, coupled with an increase in the intensity of patient demand for health services and higher expenditures for more severe cancer cases, highlights the importance of maintaining continuity of care for chronic diseases during systemic shocks. These findings also suggest that the magnitude of disruptions and recovery trajectories may vary with the intensity of local COVID-19 waves and regional stress on health-care capacity, a dimension that future research could explore in greater detail. Importantly, the pandemic also triggered rapid system-level adaptations like rapid telemedicine adoption [19]. These transformations interacted with the disruptions identified in our analysis and may partially explain the heterogeneous recovery across cancer-related services.

By addressing the challenges revealed during the pandemic through effective policy interventions and innovative models of care, health systems can strengthen their resilience and improve outcomes for cancer patients in future health emergencies [36]. Understanding the impact of the COVID-19 pandemic on cancer care thus provides essential insights for developing strategies that enhance preparedness, continuity, and adaptability in the face of global health challenges.

## Supplementary Information

Additional file 1.

## Data Availability

The data that support the findings of this study are available from Ministerio de Salud y Protección Social de Colombia and Cuenta de Alto Costo (CAC) but restrictions apply to the availability of these data, which were used under license for the current study, and so are not publicly available. Data are however available from the authors upon reasonable request and with permission of Ministerio de Salud y Protección Social de Colombia and Cuenta de Alto Costo (CAC). Programming language: Initial data processing was performed using R software (version 4.3.1; R Core Team, 2023). Final statistical analysis was performed using STATA 18.

## Abbreviations

15MC: Fifteen-month follow-up cohorts
2YC: Two-year follow-up cohorts
ACHO: Asociación Colombiana de Hematología y Oncología
ATT: Average treatment effect on the treated
CAC: Cuenta de Alto Costo
CPI: Consumer Price Index
DANE: Departamento Administrativo Nacional de Estadística
EPS: Entidad Promotora de Salud
GDP: Gross domestic product
HMO: Health maintenance organizations
ICD: International Classification of Diseases
ICU: Intensive Care Unit
NN-5: Five-nearest-neighbor
PSM: Propensity score matching
REPS: Registro Especial de Prestadores de Servicios de Salud
VS: Vital Statistics
