# Supplementary material for: Cancer care under systemic shock: utilization declines and cost increases during the COVID-19 pandemic in Colombia, evidence from matched administrative cohorts
Source: Arch Public Health. 2026 May 28;84:170. doi: 10.1186/s13690-026-01922-2 (PMC13421995; doi:10.1186/s13690-026-01922-2)
Supplement: Supplementary file 1 — Additional file 1. [file 13690_2026_1922_MOESM1_ESM.pdf]

Article title: The impact of COVID-19 emergency on cancer care in Colombia using propensity score matching and fixed effect model.

Journal name: International Journal of Health Economics and Management

Author names: Daniel Medina-Gaspar, Valeria Bejarano, Luis Orozco, Meisser Madera, Paul Rodríguez, Ricardo Bruges, Sara Atehortúa and Giancarlo Romano.

Affiliation and e-mail address of the corresponding author: Luis Orozco, Instituto de Evaluación Tecnológica en Salud (IETS), lesteban.orozco@udea.edu.co.

Supplementary material
Annex 1. Selection of International Classification of Diseases (ICD) cancer codes
Table A1. ICD codes considered for the analysis

ICD10	Description	Cancer
C160	Malignant neoplasm of cardia	Stomach
C161	Malignant neoplasm of fundus of stomach	Stomach
C162	Malignant neoplasm of body of stomach	Stomach
C163	Malignant neoplasm of pyloric antrum	Stomach
C164	Malignant neoplasm of pylorus	Stomach
C165	Malignant neoplasm of lesser curvature of stomach, unspecified	Stomach
C166	Malignant neoplasm of greater curvature of stomach, unspecified	Stomach
C169	Malignant neoplasm of stomach, unspecified	Stomach
C180	Malignant neoplasm of cecum	Colorectal
C181	Malignant neoplasm of appendix	Colorectal
C182	Malignant neoplasm of ascending colon	Colorectal
C183	Malignant neoplasm of hepatic flexure	Colorectal
C184	Malignant neoplasm of transverse colon	Colorectal
C185	Malignant neoplasm of splenic flexure	Colorectal
C186	Malignant neoplasm of descending colon	Colorectal
C187	Malignant neoplasm of sigmoid colon	Colorectal
C189	Malignant neoplasm of colon, unspecified	Colorectal
C340	Malignant neoplasm: Main bronchus	Lung
C341	Malignant neoplasm: Upper lobe, bronchus or lung	Lung
C342	Malignant neoplasm of middle lobe, bronchus or lung	Lung
C343	Malignant neoplasm: Lower lobe, bronchus or lung	Lung
C349	Malignant neoplasm: Bronchus or lung, unspecified	Lung
C500	Malignant neoplasm of nipple and areola	Breast
C501	Malignant neoplasm of central portion of breast	Breast
C502	Malignant neoplasm of upper-inner quadrant of breast	Breast
C503	Malignant neoplasm of lower-inner quadrant of breast	Breast
C504	Malignant neoplasm of upper-outer quadrant of breast	Breast
C505	Malignant neoplasm of lower-outer quadrant of breast	Breast

C506	Malignant neoplasm of axillary tail of breast	Breast
C508	Malignant neoplasm of overlapping sites of breast	Breast
C509	Malignant neoplasm of breast of unspecified site	Breast
C530	Malignant neoplasm of endocervix	Cervical
C531	Malignant neoplasm of exocervix	Cervical
C539	Malignant neoplasm of cervix uteri, unspecified	Cervical
C61	Malignant neoplasm of prostate	Prostate
D022	Carcinoma in situ: Bronchus and lung	Lung
D050	Lobular carcinoma in situ of breast	Breast
D051	Intraductal carcinoma in situ of breast	Breast
D057	Other carcinoma in situ of breast	Breast
D059	Carcinoma in situ of breast, unspecified	Breast
D060	Carcinoma in situ of endocervix	Cervical
D061	Carcinoma in situ of exocervix	Cervical
D067	Carcinoma in situ of other parts of cervix	Cervical
D069	Carcinoma in situ of cervix, unspecified	Cervical
D075	Carcinoma in situ of prostate	Prostate

Annex 2. Selection of medical procedures and drugs by type of cancer

We identified a set of interventions and treatments common to the six types of cancer analyzed. These procedures include surgical consultations, palliative care consultations, follow-up consultations, and radiology consultations, which are essential for cancer treatment assessment and monitoring. Additionally, spread procedures encompass a variety of interventions aimed at controlling cancer spread. Furthermore, diagnostic imaging tests and inpatient services are present to measure early detection and timely treatment of the disease. Finally, therapies such as monotherapy, polytherapy and teletherapy are pivotal in cancer treatment. We analyzed the following specific procedures for each cancer:

- *Colorectal*: cecectomy, colectomy, and colonoscopy. Cecectomy and colectomy are surgical removal procedures aimed at addressing cancerous tissue in the cecum and colon, respectively. Meanwhile, colonoscopy serves as a diagnostic procedure used to examine the colon and rectum for abnormalities or signs of cancer.
- *Cervical*: cervicovaginal cytology, conization, hysterectomy, lymphadenectomy, and brachytherapy. First one serves as a screening test, while conization, hysterectomy and lymphadenectomy are surgery procedure. Brachytherapy delivers targeted radiation.
- *Stomach*: endoscopy, gastrectomy, and lymphadenectomy. Endoscopy is a diagnostic procedure. Gastrectomy involves the surgical removal of part or all the stomach affected by cancer, and Lymphadenectomy is a surgical procedure to remove lymph nodes near the stomach to determine if cancer has spread.
- *Breast:* breast reconstruction, mastectomy, mammography, and quadrantectomy. Breast reconstruction is a surgical mama reconstruction procedure, while Mastectomy and Quadrantectomy involves the surgical removal of all, or part of the breast affected by cancer. Mammography is a diagnostic imaging test.
- *Prostate:* prostatectomy involves the surgical removal of the prostate gland.
- *Lung*: bronchoscopy, pleural surgery, thoracentesis, and pleurodesis. Bronchoscopy aids in diagnosis, while pleural surgery addresses pleural complications. Thoracentesis removes excess fluid, and pleurodesis prevents its recurrence.

For the selection and extraction of health services, we used identifiers in the Colombian health system. Specifically, we utilize the unique medication codes (CUM, in Spanish) for drugs, and the unique classification of health procedures (CUPS, in Spanish) for medical procedures. For the analysis, changes in the identifiers or description of drugs and procedures over time were taken into account.

Table A2.1. Medical procedures considered for the analysis

Cancer	CUPS	Description CUPS	Variable	PSM
Breast	890232	CONSULTA DE PRIMERA VEZ POR ESPECIALISTA EN CIRUGÍA DE MAMA Y TUMORES DE TEJIDOS BLANDOS	Surgical consultation	cons_cirugia
Breast	890237	CONSULTA DE PRIMERA VEZ POR ESPECIALISTA EN CIRUGÍA ONCOLÓGICA	Surgical consultation	cons_cirugia
Breast	890239	CONSULTA DE PRIMERA VEZ POR ESPECIALISTA EN CIRUGÍA PLÁSTICA, ESTÉTICA Y RECONSTRUCTIVA	Surgical consultation	cons_cirugia

Breast	890243	CONSULTA DE PRIMERA VEZ POR ESPECIALISTA EN DOLOR Y CUIDADOS PALIATIVOS	Palliative care consultation	cons_paliativo
Breast	890248	CONSULTA DE PRIMERA VEZ POR ESPECIALISTA EN GENETICA MEDICA	Follow-up consultation	cons_seguim
Breast	890255	CONSULTA DE PRIMERA VEZ POR ESPECIALISTA EN MASTOLOGÍA	Follow-up consultation	cons_seguim
Breast	890278	CONSULTA DE PRIMERA VEZ POR ESPECIALISTA EN ONCOLOGÍA	Follow-up consultation	cons_seguim
Breast	890287	CONSULTA DE PRIMERA VEZ POR ESPECIALISTA EN RADIOTERAPIA	Radiology consultation	cons_radio
Breast	890332	CONSULTA DE CONTROL O DE SEGUIMIENTO POR ESPECIALISTA EN CIRUGIA DE MAMA Y TUMORES DE TEJIDOS BLANDOS	Surgical consultation	cons_cirugia
Breast	890337	CONSULTA DE CONTROL O DE SEGUIMIENTO POR ESPECIALISTA EN CIRUGÍA ONCOLÓGICA	Surgical consultation	cons_cirugia
Breast	890339	CONSULTA DE CONTROL O DE SEGUIMIENTO POR ESPECIALISTA EN CIRUGIA PLASTICA, ESTETICA Y RECONSTRUCTIVA	Surgical consultation	cons_cirugia
Breast	890343	CONSULTA DE CONTROL O DE SEGUIMIENTO POR ESPECIALISTA EN DOLOR Y CUIDADOS PALIATIVOS	Palliative care consultation	cons_paliativo
Breast	890348	CONSULTA DE CONTROL O DE SEGUIMIENTO POR ESPECIALISTA EN GENETICA MEDICA	Follow-up consultation	cons_seguim
Breast	890355	CONSULTA DE CONTROL O DE SEGUIMIENTO POR ESPECIALISTA EN MASTOLOGÍA	Follow-up consultation	cons_seguim
Breast	890378	CONSULTA DE CONTROL O DE SEGUIMIENTO POR ESPECIALISTA EN ONCOLOGÍA	Follow-up consultation	cons_seguim
Breast	890387	CONSULTA DE CONTROL O DE SEGUIMIENTO POR ESPECIALISTA EN RADIOTERAPIA	Radiology consultation	cons_radio
Breast	890432	INTERCONSULTA POR ESPECIALISTA EN CIRUGÍA DE MAMA Y TUMORES DE TEJIDOS BLANDOS	Surgical consultation	cons_cirugia
Breast	890437	INTERCONSULTA POR ESPECIALISTA EN CIRUGÍA ONCOLÓGICA	Surgical consultation	cons_cirugia
Breast	890439	INTERCONSULTA POR ESPECIALISTA EN CIRUGÍA PLÁSTICA, ESTÉTICA Y RECONSTRUCTIVA	Surgical consultation	cons_cirugia
Breast	890443	INTERCONSULTA POR ESPECIALISTA EN DOLOR Y CUIDADOS PALIATIVOS	Palliative care consultation	cons_paliativo
Breast	890448	INTERCONSULTA POR ESPECIALISTA EN GENÉTICA MÉDICA	Follow-up consultation	cons_seguim
Breast	890455	INTERCONSULTA POR ESPECIALISTA EN MASTOLOGÍA	Follow-up consultation	cons_seguim
Breast	890478	INTERCONSULTA POR ESPECIALISTA EN ONCOLOGÍA	Follow-up consultation	cons_seguim
Breast	890487	INTERCONSULTA POR ESPECIALISTA EN RADIOTERAPIA	Radiology consultation	cons_radio
Breast	852100	RESECCIÓN LOCAL DE LESIÓN DE MAMA SOD	Quadrantectomy	cuadrantectomia
Breast	852200	RESECCION DE CUADRANTE DE MAMA SOD	Quadrantectomy	cuadrantectomia
Breast	852201	RESECCIÓN DE CUADRANTE DE MAMA	Quadrantectomy	cuadrantectomia

Breast	852202	RESECCIÓN DE CUADRANTE DE MAMA CON CONDUCTOS TERMINALES	Quadrantectomy	cuadrantecto mia
Breast	852300	MASTECTOMIA SUBTOTAL SOD	Quadrantectomy	cuadrantecto mia
Breast	852401	ESCISIÓN DE PEZÓN ACCESORIO O SUPERNUMERARIO	Quadrantectomy	cuadrantecto mia
Breast	852500	ESCISION DE PEZON SOD	Quadrantectomy	cuadrantecto mia
Breast	851101	BIOPSIA POR PUNCION CON AGUJA FINA DE MAMA	Screening	diagnostico
Breast	851102	BIOPSIA DE MAMA CON AGUJA (TRUCUT)	Screening	diagnostico
Breast	851103	BIOPSIA DE MAMA POR ESTEREOTAXIA	Screening	diagnostico
Breast	851200	BIOPSIA ABIERTA DE MAMA SOD	Screening	diagnostico
Breast	851301	LOCALIZACION DE LESION NO PALPABLE DE MAMA CON ARPON U OTRO DISPOSITIVO	Screening	diagnostico
Breast	851302	LOCALIZACION DE LESION NO PALPABLE DE MAMA POR ESTEREOTAXIA	Screening	diagnostico
Breast	851303	LOCALIZACION DE LESION NO PALPABLE DE MAMA RADIOGUIADA	Screening	diagnostico
Breast	340903	RECONSTRUCCION DE LA PARED TORACICA ANTERIOR CON COLGAJO (MUSCULAR O DE EPIPLON)	Spread procedures	diseminacio n
Breast	345001	TORACENTESIS DIAGNOSTICA	Spread procedures	diseminacio n
Breast	345002	TORACENTESIS DE DRENAJE O DESCOMPRESIVA	Spread procedures	diseminacio n
Breast	345102	PLEURECTOMIA PARIETAL POR TORACOSCOPIA	Spread procedures	diseminacio n
Breast	345201	PLEURODESIS QUIMICA VIA ABIERTA	Spread procedures	diseminacio n
Breast	345202	PLEURODESIS QUIMICA POR TORACOSCOPIA	Spread procedures	diseminacio n
Breast	345302	DECORTICACION PULMONAR POR TORACOSCOPIA	Spread procedures	diseminacio n
Breast	345403	BIOPSIAS DE PLEURA POR TORACOSCOPIA	Spread procedures	diseminacio n
Breast	401001	BIOPSIA DE GANGLIO LINFATICO CENTINELA CON TINCION	Spread procedures	diseminacio n
Breast	401002	BIOPSIA DE GANGLIO LINFATICO CENTINELA CON RADIOMARCACION	Spread procedures	diseminacio n
Breast	401002	BIOPSIA DE GANGLIO LINFATICO CENTINELA CON RADIOMARCACION	Spread procedures	diseminacio n
Breast	401101	BIOPSIA DE GANGLIO LINFATICO SUPERFICIAL	Spread procedures	diseminacio n
Breast	401102	BIOPSIA DE GANGLIO LINFATICO PROFUNDO	Spread procedures	diseminacio n
Breast	402201	ESCISION DE GANGLIO LINFATICO MAMARIO INTERNO	Spread procedures	diseminacio n
Breast	402301	ESCISION DE GANGLIO LINFATICO AXILAR VIA ABIERTA	Spread procedures	diseminacio n
Breast	405101	VACIAMIENTO RADICAL LINFATICO AXILAR VIA ABIERTA	Spread procedures	diseminacio n
Breast	405202	VACIAMIENTO RADICAL LINFATICO (LINFADENECTOMIA) DE MEDIASTINO VIA TORACOSCOPICA	Spread procedures	diseminacio n

Breast	441302	ESOFAGOGASTRODUODENOSCOPIA [EGD] CON O SIN BIOPSIA	Spread procedures	diseminacio n
Breast	452301	COLONOSCOPIA TOTAL	Spread procedures	diseminacio n
Breast	452305	COLONOSCOPIA TOTAL CON O SIN BIOPSIA	Spread procedures	diseminacio n
Breast	482101	PROCTOSIGMOIDOSCOPIA TRANSABDOMINAL	Spread procedures	diseminacio n
Breast	501002	BIOPSIA CERRADA (PERCUTANEA) (AGUJA) DE HIGADO	Spread procedures	diseminacio n
Breast	542801	PARACENTESIS ABDOMINAL DIAGNOSTICA VIA PERCUTANEA	Spread procedures	diseminacio n
Breast	542802	PARACENTESIS ABDOMINAL TERAPEUTICA VIA PERCUTANEA	Spread procedures	diseminacio n
Breast	543301	ESCISION DE LESION AMPLIA EN LA PARED ABDOMINAL CON ROTACION DE COLGAJO	Spread procedures	diseminacio n
Breast	547503	PLASTIA DE PARED ABDOMINAL VIA ABIERTA	Spread procedures	diseminacio n
Breast	547505	RECONSTRUCCION DE PARED ABDOMINAL ANATOMICA Y FUNCIONAL VIA ABIERTA	Spread procedures	diseminacio n
Breast	669102	SALPINGO-OOFORECTOMIA UNILATERAL POR LAPAROSCOPIA	Spread procedures	diseminacio n
Breast	669201	SALPINGO-OOFORECTOMIA BILATERAL POR LAPAROTOMIA	Spread procedures	diseminacio n
Breast	669202	SALPINGO-OOFORECTOMIA BILATERAL POR LAPAROSCOPIA	Spread procedures	diseminacio n
Breast	681105	BIOPSIA DE ENDOMETRIO	Spread procedures	diseminacio n
Breast	681201	HISTEROSCOPIA	Spread procedures	diseminacio n
Breast	682302	RESECCION DE POLIPO ENDOMETRIAL POR HISTEROSCOPIA	Spread procedures	diseminacio n
Breast	684003	HISTERECTOMIA TOTAL POR LAPAROTOMIA	Spread procedures	diseminacio n
Breast	684020	HISTERECTOMIA TOTAL POR LAPAROSCOPIA	Spread procedures	diseminacio n
Breast	684103	HISTERECTOMIA TOTAL ABDOMINAL AMPLIADA POR LAPAROTOMIA	Spread procedures	diseminacio n
Breast	690103	LEGRADO UTERINO GINECOLOGICO	Spread procedures	diseminacio n
Breast	702203	COLPOSCOPIA	Spread procedures	diseminacio n
Breast	707703	COLPOPEXIA POR LAPAROSCOPIA	Spread procedures	diseminacio n
Breast	872002	RADIOGRAFIA DE ABDOMEN SIMPLE	Spread procedures	diseminacio n
Breast	872011	RADIOGRAFIA DE ABDOMEN SIMPLE CON PROYECCIONES ADICIONALES (SERIE DE ABDOMEN AGUDO)	Spread procedures	diseminacio n

Breast	879111	TOMOGRAFIA COMPUTADA DE CRANEO SIMPLE	Spread procedures	diseminacio n
Breast	879112	TOMOGRAFIA COMPUTADA DE CRANEO CON CONTRASTE	Spread procedures	diseminacio n
Breast	879113	TOMOGRAFIA COMPUTADA DE CRANEO SIMPLE Y CON CONTRASTE	Spread procedures	diseminacio n
Breast	879201	TOMOGRAFIA COMPUTADA DE COLUMNA SEGMENTOS CERVICAL, TORACICO, LUMBAR O SACRO, POR CADA NIVEL (TRES ESPACIOS)	Spread procedures	diseminacio n
Breast	879205	TOMOGRAFIA COMPUTADA DE COLUMNA SEGMENTOS CERVICAL, TORACICO, LUMBAR O SACRO, COMPLEMENTO A MIELOGRAFIA (CADA SEGMENTO)	Spread procedures	diseminacio n
Breast	879410	TOMOGRAFÍA COMPUTADA DE ABDOMEN SUPERIOR	Spread procedures	diseminacio n
Breast	879420	TOMOGRAFIA COMPUTADA DE ABDOMEN Y PELVIS (ABDOMEN TOTAL)	Spread procedures	diseminacio n
Breast	879460	TOMOGRAFIA COMPUTADA DE PELVIS	Spread procedures	diseminacio n
Breast	881213	ECOGRAFIA ENDOSCOPICA DE MEDIASTINO, TRAQUEA Y BRONQUIOS	Spread procedures	diseminacio n
Breast	881301	ECOGRAFIA DE TEJIDOS BLANDOS DE PARED ABDOMINAL Y DE PELVIS	Spread procedures	diseminacio n
Breast	881302	ECOGRAFIA DE ABDOMEN TOTAL	Spread procedures	diseminacio n
Breast	881302	ECOGRAFIA DE ABDOMEN TOTAL	Spread procedures	diseminacio n
Breast	881305	ECOGRAFIA DE ABDOMEN SUPERIOR	Spread procedures	diseminacio n
Breast	881306	ECOGRAFIA DE HIGADO, PANCREAS, VIA BILIAR Y VESICULA	Spread procedures	diseminacio n
Breast	881402	ECOGRAFIA PELVICA GINECOLOGICA TRANSABDOMINAL	Spread procedures	diseminacio n
Breast	883101	RESONANCIA MAGNETICA DE CEREBRO	Spread procedures	diseminacio n
Breast	883401	RESONANCIA MAGNETICA DE ABDOMEN	Spread procedures	diseminacio n
Breast	886011	OSTEODENSITOMETRIA POR TC	Spread procedures	diseminacio n
Breast	886012	OSTEODENSITOMETRIA POR ABSORCION DUAL	Spread procedures	diseminacio n
Breast	886013	OSTEODENSITOMETRIA Y COMPOSICION CORPORAL (TEJIDOS BLANDOS)	Spread procedures	diseminacio n
Breast	903809	BILIRRUBINAS TOTAL Y DIRECTA	Spread procedures	diseminacio n
Breast	903856	NITROGENO UREICO	Spread procedures	diseminacio n
Breast	903857	NITROGENO UREICO EN ORINA DE 24 HORAS	Spread procedures	diseminacio n
Breast	903866	TRANSAMINASA GLUTAMICO-PIRUVICA [ALANINO AMINO TRANSFERASA]	Spread procedures	diseminacio n

Breast	903867	TRANSAMINASA GLUTAMICO OXALACETICA [ASPARTATO AMINO TRANSFERASA]	Spread procedures	diseminacion
Breast	903876	CREATININA EN ORINA PARCIAL	Spread procedures	diseminacion
Breast	903881	CREATINA EN ORINA DE 24 HORAS	Spread procedures	diseminacion
Breast	903895	CREATININA EN SUERO U OTROS FLUIDOS	Spread procedures	diseminacion
Breast	871121	RADIOGRAFIA DE TORAX (P.A. O A.P. Y LATERAL, DECUBITO LATERAL, OBLICUAS O LATERAL)	Diagnostic imaging	imag_diagnos
Breast	871401	TOMOGRAFÍA COMPUTADA DE TÓRAX	Diagnostic imaging	imag_diagnos
Breast	871402	TOMOGRAFÍA COMPUTADA DE TÓRAX	Diagnostic imaging	imag_diagnos
Breast	879301	TOMOGRAFIA COMPUTADA DE TORAX	Diagnostic imaging	imag_diagnos
Breast	879391	TOMOGRAFIA COMPUTADA DE TORAX EXTENDIDO AL ABDOMEN SUPERIOR CON SUPRARRENALES	Diagnostic imaging	imag_diagnos
Breast	879601	TOMOGRAFIA POR EMISION DE POSITRONES [PET-TC]	Diagnostic imaging	imag_diagnos
Breast	879910	TOMOGRAFIA COMPUTADA EN RECONSTRUCCION TRIDIMENSIONAL	Diagnostic imaging	imag_diagnos
Breast	879990	TOMOGRAFIA COMPUTADA COMO GUIA PARA PROCEDIMIENTOS	Diagnostic imaging	imag_diagnos
Breast	881202	ECOCARDIOGRAMA TRANSTORACICO	Diagnostic imaging	imag_diagnos
Breast	881203	ECOCARDIOGRAMA TRANSTORACICO CON CONTRASTE	Diagnostic imaging	imag_diagnos
Breast	881204	ECOCARDIOGRAMA TRANSTORACICO TRIDIMENSIONAL	Diagnostic imaging	imag_diagnos
Breast	881211	ECOGRAFIA DE TORAX (PERICARDIO O PLEURA)	Diagnostic imaging	imag_diagnos
Breast	881212	ECOGRAFIA DE OTROS SITIOS TORACICOS	Diagnostic imaging	imag_diagnos
Breast	881214	ECOCARDIOGRAMA TRANSTORACICO CON ANALISIS DE LA DEFORMIDAD MIOCARDICA	Diagnostic imaging	imag_diagnos
Breast	883220	RESONANCIA MAGNETICA DE COLUMNA TORACICA SIMPLE	Diagnostic imaging	imag_diagnos
Breast	883221	RESONANCIA MAGNETICA DE COLUMNA TORACICA CON CONTRASTE	Diagnostic imaging	imag_diagnos
Breast	883230	RESONANCIA MAGNETICA DE COLUMNA LUMBOSACRA SIMPLE	Diagnostic imaging	imag_diagnos
Breast	883231	RESONANCIA MAGNETICA DE COLUMNA LUMBAR CON CONTRASTE	Diagnostic imaging	imag_diagnos
Breast	883301	RESONANCIA MAGNÉTICA DEL TÓRAX	Diagnostic imaging	imag_diagnos
Breast	890602	CUIDADO (MANEJO) INTRAHOSPITALARIO POR MEDICINA ESPECIALIZADA	Inpatient services	internacion
Breast	107M01	INTERNACIÓN EN UNIDAD DE CUIDADO INTERMEDIO ADULTO	Inpatient services	internacion
Breast	10A001	INTERNACIÓN COMPLEJIDAD ALTA HABITACION UNIPERSONAL (INCLUYE AISLAMIENTO)	Inpatient services	internacion

Breast	10A002	INTERNACIÓN COMPLEJIDAD ALTA HABITACION BIPERSONAL	Inpatient services	internacion
Breast	10A003	INTERNACIÓN COMPLEJIDAD ALTA TRES CAMAS	Inpatient services	internacion
Breast	10A004	INTERNACIÓN COMPLEJIDAD ALTA CUATRO O MÁS CAMAS	Inpatient services	internacion
Breast	10A005	INTERNACIÓN EN UNIDAD DE TRASPLANTE	Inpatient services	internacion
Breast	10M001	INTERNACIÓN COMPLEJIDAD MEDIANA HABITACION UNIPERSONAL (INCLUYE AISLAMIENTO)	Inpatient services	internacion
Breast	10M002	INTERNACIÓN COMPLEJIDAD MEDIANA HABITACION BIPERSONAL	Inpatient services	internacion
Breast	10M003	INTERNACIÓN COMPLEJIDAD MEDIANA HABITACION TRES CAMAS	Inpatient services	internacion
Breast	10M004	INTERNACIÓN COMPLEJIDAD MEDIANA HABITACION CUATRO O MÁS CAMAS	Inpatient services	internacion
Breast	110A01	INTERNACIÓN EN UNIDAD DE CUIDADO INTENSIVO ADULTOS	Inpatient services	internacion
Breast	121M01	INTERNACIÓN DE PACIENTE CRÓNICO TERMINAL SIN VENTILADOR COMPLEJIDAD MEDIANA	Inpatient services	internacion
Breast	121M02	INTERNACIÓN DE PACIENTE CRÓNICO TERMINAL CON VENTILADOR COMPLEJIDAD MEDIANA	Inpatient services	internacion
Breast	S11201	INTERNACIÓN COMPLEJIDAD MEDIANA HABITACION UNIPERSONAL (INCLUYE AISLAMIENTO)	Inpatient services	internacion
Breast	S11202	INTERNACIÓN COMPLEJIDAD MEDIANA HABITACION BIPERSONAL	Inpatient services	internacion
Breast	S11203	INTERNACIÓN COMPLEJIDAD MEDIANA HABITACION TRES CAMAS	Inpatient services	internacion
Breast	S11204	INTERNACIÓN COMPLEJIDAD MEDIANA HABITACION CUATRO O MÁS CAMAS	Inpatient services	internacion
Breast	S11301	INTERNACIÓN COMPLEJIDAD ALTA HABITACION UNIPERSONAL (INCLUYE AISLAMIENTO)	Inpatient services	internacion
Breast	S11302	INTERNACIÓN COMPLEJIDAD ALTA HABITACION BIPERSONAL	Inpatient services	internacion
Breast	S11303	INTERNACIÓN COMPLEJIDAD ALTA TRES CAMAS	Inpatient services	internacion
Breast	S11304	INTERNACIÓN COMPLEJIDAD ALTA CUATRO O MÁS CAMAS	Inpatient services	internacion
Breast	S12103	INTERNACIÓN EN UNIDAD DE CUIDADO INTENSIVO ADULTOS	Inpatient services	internacion
Breast	S12203	INTERNACIÓN EN UNIDAD DE CUIDADO INTERMEDIO ADULTO	Inpatient services	internacion
Breast	S12600	INTERNACIÓN EN UNIDAD DE TRASPLANTE	Inpatient services	internacion
Breast	898003	ESTUDIO DE COLORACION BASICA EN CITOLOGIA POR ASPIRACION DE CUALQUIER TEJIDO U ORGANO [BACAF]	Laboratory tests	laboratorio
Breast	898007	ESTUDIO DE COLORACION HISTOQUIMICA EN CITOLOGIA POR ASPIRACION DE CUALQUIER TEJIDO U ORGANO [BACAF]	Laboratory tests	laboratorio
Breast	898011	ESTUDIO DE COLORACION INMUNOHISTOQUIMICA EN CITOLOGIA POR ASPIRACION DE CUALQUIER TEJIDO U ORGANO [BACAF]	Laboratory tests	laboratorio
Breast	898033	ESTUDIO DE RECEPTORES HORMONALES EN CITOLOGIA	Laboratory tests	laboratorio
Breast	898101	ESTUDIO DE COLORACION BASICA EN BIOPSIA	Laboratory tests	laboratorio
Breast	898102	ESTUDIO DE COLORACION HISTOQUIMICA EN BIOPSIA	Laboratory tests	laboratorio
Breast	898102	ESTUDIO DE COLORACION HISTOQUIMICA EN BIOPSIA	Laboratory tests	laboratorio
Breast	898103	ESTUDIO DE COLORACION INMUNOHISTOQUIMICA EN BIOPSIA	Laboratory tests	laboratorio
Breast	898104	ESTUDIO DE COLORACION DE INMUNOFLUORESCENCIA EN BIOPSIA	Laboratory tests	laboratorio
Breast	898105	ESTUDIO DE BIOLOGIA MOLECULAR EN BIOPSIA	Laboratory tests	laboratorio
Breast	898106	ESTUDIO DE CITOMETRIA DE FLUJO EN BIOPSIA	Laboratory tests	laboratorio
Breast	898107	ESTUDIO DE MICROSCOPIA ELECTRONICA EN BIOPSIA	Laboratory tests	laboratorio
Breast	898110	ESTUDIO DE RECEPTORES HORMONALES EN BIOPSIA	Laboratory tests	laboratorio
Breast	898110	ESTUDIO DE RECEPTORES HORMONALES EN BIOPSIA	Laboratory tests	laboratorio
Breast	898201	ESTUDIO DE COLORACION BASICA EN ESPECIMEN DE RECONOCIMIENTO	Laboratory tests	laboratorio
Breast	898202	ESTUDIO DE COLORACION HISTOQUIMICA EN ESPECIMEN DE RECONOCIMIENTO	Laboratory tests	laboratorio
Breast	898203	ESTUDIO DE COLORACION INMUNOHISTOQUIMICA EN ESPECIMEN DE RECONOCIMIENTO	Laboratory tests	laboratorio
Breast	898205	ESTUDIO DE BIOLOGIA MOLECULAR EN ESPECIMEN DE RECONOCIMIENTO	Laboratory tests	laboratorio
Breast	898210	ESTUDIO DE RECEPTORES HORMONALES EN ESPECIMEN DE RECONOCIMIENTO	Laboratory tests	laboratorio
Breast	898210	ESTUDIO DE RECEPTORES HORMONALES EN ESPECIMEN DE RECONOCIMIENTO	Laboratory tests	laboratorio
Breast	898221	ESTUDIO DE COLORACION BASICA EN ESPECIMEN CON MULTIPLE MUESTREO	Laboratory tests	laboratorio
Breast	898221	ESTUDIO DE COLORACION BASICA EN ESPECIMEN CON MULTIPLE MUESTREO	Laboratory tests	laboratorio
Breast	898222	ESTUDIO DE COLORACION HISTOQUIMICA EN ESPECIMEN CON MULTIPLE MUESTREO	Laboratory tests	laboratorio
Breast	898226	ESTUDIO DE CITOMETRIA DE FLUJO EN ESPECIMEN CON MULTIPLE MUESTREO	Laboratory tests	laboratorio

Breast	898230	ESTUDIO DE RECEPTORES HORMONALES EN ESPECIMEN CON MULTIPLE MUESTREO	Laboratory tests	laboratorio
Breast	898230	ESTUDIO DE RECEPTORES HORMONALES EN ESPECIMEN CON MULTIPLE MUESTREO	Laboratory tests	laboratorio
Breast	898241	ESTUDIO DE COLORACION BASICA EN ESPECIMEN CON RESECCION DE MARGENES	Laboratory tests	laboratorio
Breast	898242	ESTUDIO DE COLORACION HISTOQUIMICA EN ESPECIMEN CON RESECCION DE MARGENES	Laboratory tests	laboratorio
Breast	898243	ESTUDIO DE COLORACION INMUNOHISTOQUIMICA EN ESPECIMEN CON RESECCION DE MARGENES	Laboratory tests	laboratorio
Breast	898250	ESTUDIO DE RECEPTORES HORMONALES EN ESPECIMEN CON RESECCION DE MARGENES	Laboratory tests	laboratorio
Breast	898250	ESTUDIO DE RECEPTORES HORMONALES EN ESPECIMEN CON RESECCION DE MARGENES	Laboratory tests	laboratorio
Breast	898262	ESTUDIO DE COLORACION HISTOQUIMICA EN ESPECIMEN CON MAPEO	Laboratory tests	laboratorio
Breast	904105	HORMONA FOLICULO ESTIMULANTE	Laboratory tests	laboratorio
Breast	904506	ESTROGENOS (ESTRADIOL 17 BETA)	Laboratory tests	laboratorio
Breast	906604	ANTIGENO DE CANCER DE MAMA [CA 15-3] SEMIAUTOMATIZADO O AUTOMATIZADO	Laboratory tests	laboratorio
Breast	908413	ERBB2 [HER-2/neu] (ONCOGEN) HIBRIDACION "IN SITU"	Laboratory tests	laboratorio
Breast	908420	ESTUDIOS MOLECULARES DE GENES (ESPECIFICOS)	Laboratory tests	laboratorio
Breast	908433	BRCA1 Y BRCA2 SECUENCIACION COMPLETA	Laboratory tests	laboratorio
Breast	920402	VENTRICULOGRAFIA DE PRIMER PASO	Laboratory tests	laboratorio
Breast	920901	GAMAGRAFIA OSEA (CORPORAL TOTAL O SEGMENTARIA)	Laboratory tests	laboratorio
Breast	920902	GAMAGRAFIA OSEA DE TRES FASES	Laboratory tests	laboratorio
Breast	920903	GAMAGRAFIA OSEA CON SPECT	Laboratory tests	laboratorio
Breast	876801	MAMOGRAFIA UNILATERAL O DE PIEZA QUIRURGICA	Mammography	mamografia
Breast	876802	MAMOGRAFIA BILATERAL	Mammography	mamografia
Breast	881201	ECOGRAFIA DE MAMA, CON TRANSDUCTOR DE 7 MHZ O MAS	Mammography	mamografia
Breast	883351	RESONANCIA MAGNETICA DE MAMA	Mammography	mamografia
Breast	854001	MASTECTOMIA SUBCUTANEA CON RECONSTRUCCION SIMULTANEA	Mastectomy	mastectomia
Breast	854101	MASTECTOMIA SIMPLE UNILATERAL	Mastectomy	mastectomia
Breast	854102	MASTECTOMIA SIMPLE UNILATERAL POR GLANDULA SUPERNUMERARIA	Mastectomy	mastectomia
Breast	854103	MASTECTOMIA SIMPLE UNILATERAL CON PRESERVACION DE PIEL O COMPLEJO AREOLA PEZON	Mastectomy	mastectomia
Breast	854201	MASTECTOMIA SIMPLE BILATERAL	Mastectomy	mastectomia
Breast	854202	MASTECTOMIA SIMPLE BILATERAL POR GLANDULA SUPERNUMERARIA	Mastectomy	mastectomia
Breast	854203	MASTECTOMIA SIMPLE BILATERAL CON PRESERVACION DE PIEL O COMPLEJO AREOLA PEZON	Mastectomy	mastectomia
Breast	854301	MASTECTOMIA SIMPLE CON ESCISION DE GANGLIOS LINFATICOS REGIONALES	Mastectomy	mastectomia
Breast	854401	MASTECTOMIA SIMPLE AMPLIADA BILATERAL VIA ABIERTA	Mastectomy	mastectomia
Breast	854501	ESCISION DE MAMA, MUSCULOS PECTORALES Y GANGLIO LINFATICO REGIONALES	Mastectomy	mastectomia
Breast	854502	MASTECTOMIA RADICAL MODIFICADA UNILATERAL	Mastectomy	mastectomia
Breast	854601	MASTECTOMIA RADICAL BILATERAL VIA ABIERTA	Mastectomy	mastectomia
Breast	854701	ESCISIÓN DE MAMA, MÚSCULOS, GANGLIOS LINFÁTICOS (AXILARES, CLAVICULARES, SUPRACLAVICULARES, MAMARIOS INTERNOS Y MEDIASTÍNICOS)	Mastectomy	mastectomia
Breast	854801	MASTECTOMIA RADICAL AMPLIADA BILATERAL VIA ABIERTA	Mastectomy	mastectomia
Breast	992503	MONOTERAPIA ANTINEOPLÁSICA DE BAJA TOXICIDAD	Monoterapy	monoterapia
Breast	992504	POLITERAPIA ANTINEOPLÁSICA DE BAJA TOXICIDAD	Polyterapy	politerapia
Breast	992505	POLITERAPIA ANTINEOPLÁSICA DE ALTA TOXICIDAD	Polyterapy	politerapia
Breast	992506	ADMINISTRACIÓN (INFUSIÓN O PERFUSIÓN) DE TERAPIA ANTINEOPLÁSICA INTRARTERIAL (REGIONAL)	Monoterapy	monoterapia
Breast	992509	MONOTERAPIA ANTINEOPLÁSICA DE BAJA TOXICIDAD	Monoterapy	monoterapia
Breast	992511	MONOTERAPIA ANTINEOPLÁSICA DE ALTA TOXICIDAD	Monoterapy	monoterapia
Breast	922201	TELETERAPIA CON ORTOVOLTAJE	Teletherapy	teleterapia
Breast	922321	TELETERAPIA CON COBALTO (PLANEACIÓN COMPUTARIZADA BIDIMENSIONAL Y SIMULACIÓN CONVENCIONAL)	Teletherapy	teleterapia
Breast	922322	TELETERAPIA CON COBALTO (PLANEACIÓN COMPUTARIZADA TRIDIMENSIONAL Y SIMULACIÓN VIRTUAL)	Teletherapy	teleterapia
Breast	922441	TELETERAPIA CON ACELERADOR LINEAL (PLANEACIÓN COMPUTARIZADA BIDIMENSIONAL Y SIMULACIÓN CONVENCIONAL) TÉCNICA RADIOTERAPIA CONVENCIONAL	Teletherapy	teleterapia

Breast	922442	TELETERAPIA CON ACELERADOR LINEAL (PLANEACIÓN COMPUTARIZADA TRIDIMENSIONAL Y SIMULACIÓN VIRTUAL) TÉCNICA RADIOTERAPIA CONVENCIONAL	Teletherapy	teleterapia
Breast	922443	TELETERAPIA CON ACELERADOR LINEAL (PLANEACION COMPUTARIZADA TRIDIMENSIONAL Y SIMULACION VIRTUAL) TECNICA CONFORMACIONAL [3D - CRT]	Teletherapy	teleterapia
Breast	922444	TELETERAPIA CON ACELERADOR LINEAL (PLANEACIÓN COMPUTARIZADA TRIDIMENSIONAL Y SIMULACIÓN VIRTUAL) TÉCNICA RADIOTERAPIA DE INTENSIDAD MODULADA [IMRT]	Teletherapy	teleterapia
Breast	922445	TELETERAPIA CON ACELERADOR LINEAL (PLANEACIÓN COMPUTARIZADA TRIDIMENSIONAL Y SIMULACIÓN VIRTUAL) TÉCNICA RADIOTERAPIA GUIADA POR IMÁGENES [IGRT]	Teletherapy	teleterapia
Breast	922446	TELETERAPIA CON ACELERADOR LINEAL (PLANEACIÓN COMPUTARIZADA TRIDIMENSIONAL Y SIMULACIÓN VIRTUAL) TÉCNICA RADIOTERAPIA - ARCOTERAPIA DE MODULACIÓN VOLUMÉTRICA [VMAT]	Teletherapy	teleterapia
Breast	922447	IRRADIACIÓN CORPORAL TOTAL	Irradiation	irradiacion
Breast	922448	IRRADIACIÓN CUTÁNEA TOTAL	Irradiation	irradiacion
Breast	922449	TELETERAPIA CON ACELERADOR LINEAL (PLANEACIÓN COMPUTARIZADA TRIDIMENSIONAL Y SIMULACIÓN VIRTUAL) TÉCNICA RADIOTERAPIA HELICOIDAL	Teletherapy	teleterapia
Breast	922504	TELETERAPIA CON ACELERADOR LINEAL DE ELECTRONES (PLANEACION COMPUTARIZADA BIDIMENSIONAL Y SIMULACION CONVENCIONAL)	Teletherapy	teleterapia
Breast	922505	TELETERAPIA CON ACELERADOR LINEAL DE ELECTRONES (PLANEACION COMPUTARIZADA TRIDIMENSIONAL Y SIMULACIÓN VIRTUAL)	Teletherapy	teleterapia
Breast	922506	RADIOTERAPIA INTRAOPERATORIA	Teletherapy	teleterapia
Breast	922601	BRAQUITERAPIA INTERSTICIAL (PLANEACION COMPUTARIZADA TRIDIMENSIONAL Y SIMULACION VIRTUAL) CON ALTA TASA DE DOSIS	Brachytherapy	braquiterapi a
Breast	922602	BRAQUITERAPIA INTERSTICIAL (PLANEACION COMPUTARIZADA TRIDIMENSIONAL Y SIMULACIÓN VIRTUAL) CON BAJA TASA DE DOSIS	Brachytherapy	braquiterapi a
Breast	922603	BRAQUITERAPIA INTRALUMINAL (PLANEACION COMPUTARIZADA BIDIMENSIONAL Y SIMULACION CONVENCIONAL) CON ALTA TASA DE DOSIS	Brachytherapy	braquiterapi a
Breast	922604	BRAQUITERAPIA INTRALUMINAL CON BAJA TASA DE DOSIS	Brachytherapy	braquiterapi a
Breast	922605	BRAQUITERAPIA INTRACAVITARIA (PLANEACION COMPUTARIZADA BIDIMENSIONAL Y SIMULACION CONVENCIONAL) CON ALTA TASA DE DOSIS	Brachytherapy	braquiterapi a
Breast	922606	BRAQUITERAPIA INTRACAVITARIA (PLANEACION COMPUTARIZADA BIDIMENSIONAL Y SIMULACION CONVENCIONAL) CON BAJA TASA DE DOSIS	Brachytherapy	braquiterapi a
Breast	922607	BRAQUITERAPIA INTRACAVITARIA (PLANEACION COMPUTARIZADA TRIDIMENSIONAL Y SIMULACION VIRTUAL) CON ALTA TASA DE DOSIS	Brachytherapy	braquiterapi a
Breast	922608	BRAQUITERAPIA INTRACAVITARIA (PLANEACIÓN COMPUTARIZADA TRIDIMENSIONAL Y SIMULACIÓN VIRTUAL) CON BAJA TASA DE DOSIS	Brachytherapy	braquiterapi a
Breast	922609	BRAQUITERAPIA INTRALUMINAL (PLANEACIÓN COMPUTARIZADA TRIDIMENSIONAL Y SIMULACIÓN VIRTUAL) CON ALTA TASA DE DOSIS	Brachytherapy	braquiterapi a
Breast	922611	BRAQUITERAPIA DE CONTACTO (PLANEACION COMPUTARIZADA BIDIMENSIONAL Y SIMULACION CONVENCIONAL) CON ALTA TASA DE DOSIS	Brachytherapy	braquiterapi a
Breast	922612	BRAQUITERAPIA DE CONTACTO (PLANEACIÓN COMPUTARIZADA TRIDIMENSIONAL Y SIMULACIÓN VIRTUAL) CON ALTA TASA DE DOSIS	Brachytherapy	braquiterapi a
Breast	922613	BRAQUITERAPIA EPIESCLERAL DE CONTACTO (PLANEACIÓN COMPUTARIZADA TRIDIMENSIONAL Y SIMULACIÓN VIRTUAL) CON BAJA TASA DE DOSIS	Brachytherapy	braquiterapi a
Breast	922614	BRAQUITERAPIA METABÓLICA	Brachytherapy	braquiterapi a
Breast	922615	BRAQUITERAPIA INTERSTICIAL (PLANEACIÓN COMPUTARIZADA BIDIMENSIONAL Y SIMULACIÓN CONVENCIONAL) CON ALTA TASA DE DOSIS	Brachytherapy	braquiterapi a
Breast	922616	BRAQUITERAPIA INTERSTICIAL (PLANEACIÓN COMPUTARIZADA TRIDIMENSIONAL Y SIMULACIÓN VIRTUAL) CON ALTA TASA DE DOSIS	Brachytherapy	braquiterapi a

Breast	922800	TERAPIA CON RADIOISOTOPOS SOD	Radiotherapy	terapia
Breast	922801	TERAPIA CON RADIOISÓTOPOS	Radiotherapy	terapia
Breast	922805	TERAPIA CON ITRIUM 90	Radiotherapy	terapia
Breast	922810	TERAPIA CON METAIODOBENCILGUANIDINA SUPRARRENAL	Radiotherapy	terapia
Breast	922830	TERAPIA DE METÁSTASIS CON ESTRONCIO	Radiotherapy	terapia
Breast	853401	MAMOPLASTIA ONCOLOGICA UNILATERAL	Breast reconstruction	reconstr_mama
Breast	853402	MAMOPLASTIA ONCOLOGICA BILATERAL	Breast reconstruction	reconstr_mama
Breast	857201	RECONSTRUCCIÓN DE MAMA UNILATERAL CON COLGAJO	Breast reconstruction	reconstr_mama
Breast	857202	RECONSTRUCCIÓN DE MAMA BILATERAL CON COLGAJO	Breast reconstruction	reconstr_mama
Breast	857203	RECONSTRUCCIÓN DE MAMA UNILATERAL CON TEJIDO AUTÓLOGO	Breast reconstruction	reconstr_mama
Breast	857204	RECONSTRUCCIÓN DE MAMA BILATERAL CON TEJIDO AUTÓLOGO	Breast reconstruction	reconstr_mama
Breast	858200	INJERTO DE GROSOR PARCIAL EN LA PIEL DE LA MAMA SOD	Breast reconstruction	reconstr_mama
Breast	858300	INJERTO DE GROSOR TOTAL EN LA PIEL DE LA MAMA SOD	Breast reconstruction	reconstr_mama
Breast	858401	COLGAJO LOCAL EN LA MAMA	Breast reconstruction	reconstr_mama
Breast	858403	COLGAJO LOCAL MUSCULOCUTÁNEO EN LA MAMA	Breast reconstruction	reconstr_mama
Breast	858405	COLGAJO LIBRE CON TECNICA MICROVASCULAR EN LA MAMA	Breast reconstruction	reconstr_mama
Breast	858701	RECONSTRUCCIÓN DEL COMPLEJO AREOLA PEZÓN	Breast reconstruction	reconstr_mama
Breast	867104	COLGAJO COMPUESTO A DISTANCIA, EN VARIOS TIEMPOS	Breast reconstruction	reconstr_mama
Breast	867105	COLGAJO LIBRE CUTANEO CON TECNICA MICROVASCULAR	Breast reconstruction	reconstr_mama
Breast	867106	COLGAJO LIBRE COMPUESTO CON TECNICA MICROVASCULAR	Breast reconstruction	reconstr_mama
Breast	867107	COLGAJO NEUROVASCULAR (EN ISLA)	Breast reconstruction	reconstr_mama
Breast	867201	COLGAJO LOCAL DE PIEL COMPUESTO DE VECINDAD HASTA DE DOS CENTIMETROS CUADRADOS	Breast reconstruction	reconstr_mama
Breast	867202	COLGAJO LOCAL DE PIEL COMPUESTO DE VECINDAD ENTRE DOS A CINCO CENTIMETROS CUADRADOS	Breast reconstruction	reconstr_mama
Breast	867203	COLGAJO LOCAL DE PIEL COMPUESTO DE VECINDAD ENTRE CINCO A DIEZ CENTIMETROS CUADRADOS	Breast reconstruction	reconstr_mama
Cervical	892901	TOMA NO QUIRURGICA DE MUESTRA O TEJIDO VAGINAL PARA ESTUDIO CITOLOGICO	Cervicovaginal cytology	citologia_cervi
Cervical	898001	ESTUDIO DE COLORACIÓN BÁSICA EN CITOLOGÍA VAGINAL TUMORAL O FUNCIONAL	Cervicovaginal cytology	citologia_cervi
Cervical	898005	ESTUDIO DE COLORACION HISTOQUIMICA EN CITOLOGIA VAGINAL TUMORAL O FUNCIONAL	Cervicovaginal cytology	citologia_cervi

Cervical	898009	ESTUDIO DE COLORACION INMUNOHISTOQUIMICA EN CITOLOGIA VAGINAL TUMORAL O FUNCIONAL	Cervicovaginal cytology	citologia_cervi
Cervical	898015	ESTUDIO ANATOMOPATOLÓGICO BÁSICO EN CITOLOGÍA CERVICOVAGINAL MANUAL	Cervicovaginal cytology	citologia_cervi
Cervical	898016	ESTUDIO ANATOMOPATOLÓGICO BÁSICO EN CITOLOGÍA CERVICOVAGINAL SEMIAUTOMATIZADO O AUTOMATIZADO	Cervicovaginal cytology	citologia_cervi
Cervical	672001	CONIZACIÓN CERVICAL	Conization	conizacion
Cervical	673101	ESCISIÓN DE PÓLIPO EN CUELLO UTERINO [CÉRVIX]	Conization	conizacion
Cervical	673102	RESECCION DE LESION CUELLO UTERINO	Conization	conizacion
Cervical	673102	RESECCION DE LESION CUELLO UTERINO	Conization	conizacion
Cervical	673201	ABLACIÓN DE LESIÓN O TEJIDO DE CUELLO UTERINO	Conization	conizacion
Cervical	673210	ABLACIÓN DE LESIÓN O TEJIDO DE CUELLO UTERINO	Conization	conizacion
Cervical	674001	AMPUTACIÓN DE CUELLO O TRAQUELECTOMÍA POR LAPAROTOMÍA	Conization	conizacion
Cervical	674002	AMPUTACIÓN DE CUELLO O TRAQUELECTOMÍA POR LAPAROTOMÍA	Conization	conizacion
Cervical	674003	AMPUTACIÓN DE CUELLO O TRAQUELECTOMÍA POR LAPAROSCOPIA	Conization	conizacion
Cervical	674004	AMPUTACIÓN DE CUELLO O TRAQUELECTOMÍA POR VÍA VAGINAL	Conization	conizacion
Cervical	674011	AMPUTACION DE CUELLO O TRAQUELECTOMIA POR LAPAROTOMIA	Conization	conizacion
Cervical	674101	ESCISIÓN DE MUÑÓN CERVICAL POR LAPAROTOMÍA	Conization	conizacion
Cervical	674102	ESCISIÓN DE MUÑÓN CERVICAL POR LAPAROSCOPIA	Conization	conizacion
Cervical	674103	ESCISIÓN DE MUÑÓN CERVICAL POR VÍA VAGINAL	Conization	conizacion
Cervical	674501	TRAQUELECTOMIA RADICAL POR LAPAROTOMIA	Conization	conizacion
Cervical	674511	TRAQUELECTOMIA RADICAL POR LAPAROSCOPIA	Conization	conizacion
Cervical	890237	CONSULTA DE PRIMERA VEZ POR ESPECIALISTA EN CIRUGÍA ONCOLÓGICA	Surgical consultation	cons_cirugia
Cervical	890243	CONSULTA DE PRIMERA VEZ POR ESPECIALISTA EN DOLOR Y CUIDADOS PALIATIVOS	Palliative care consultation	cons_paliativo
Cervical	890248	CONSULTA DE PRIMERA VEZ POR ESPECIALISTA EN GENETICA MEDICA	Follow-up consultation	cons_seguim
Cervical	890250	CONSULTA DE PRIMERA VEZ POR ESPECIALISTA EN GINECOLOGÍA Y OBSTETRICIA	Follow-up consultation	cons_seguim
Cervical	890278	CONSULTA DE PRIMERA VEZ POR ESPECIALISTA EN ONCOLOGÍA	Follow-up consultation	cons_seguim
Cervical	890287	CONSULTA DE PRIMERA VEZ POR ESPECIALISTA EN RADIOTERAPIA	Radiology consultation	cons_radio
Cervical	890337	CONSULTA DE CONTROL O DE SEGUIMIENTO POR ESPECIALISTA EN CIRUGÍA ONCOLÓGICA	Surgical consultation	cons_cirugia
Cervical	890343	CONSULTA DE CONTROL O DE SEGUIMIENTO POR ESPECIALISTA EN DOLOR Y CUIDADOS PALIATIVOS	Palliative care consultation	cons_paliativo
Cervical	890348	CONSULTA DE CONTROL O DE SEGUIMIENTO POR ESPECIALISTA EN GENETICA MEDICA	Follow-up consultation	cons_seguim
Cervical	890350	CONSULTA DE CONTROL O DE SEGUIMIENTO POR ESPECIALISTA EN GINECOLOGÍA Y OBSTETRICIA	Follow-up consultation	cons_seguim
Cervical	890378	CONSULTA DE CONTROL O DE SEGUIMIENTO POR ESPECIALISTA EN ONCOLOGÍA	Follow-up consultation	cons_seguim
Cervical	890387	CONSULTA DE CONTROL O DE SEGUIMIENTO POR ESPECIALISTA EN RADIOTERAPIA	Radiology consultation	cons_radio
Cervical	890437	INTERCONSULTA POR ESPECIALISTA EN CIRUGÍA ONCOLÓGICA	Surgical consultation	cons_cirugia

Cervical	890443	INTERCONSULTA POR ESPECIALISTA EN DOLOR Y CUIDADOS PALIATIVOS	Palliative care consultation	cons_paliati vo
Cervical	890448	INTERCONSULTA POR ESPECIALISTA EN GENÉTICA MÉDICA	Follow-up consultation	cons_seguim
Cervical	890450	INTERCONSULTA POR ESPECIALISTA EN GINECOLOGÍA Y OBSTETRICIA	Follow-up consultation	cons_seguim
Cervical	890478	INTERCONSULTA POR ESPECIALISTA EN ONCOLOGÍA	Follow-up consultation	cons_seguim
Cervical	890487	INTERCONSULTA POR ESPECIALISTA EN RADIOTERAPIA	Radiology consultation	cons_radio
Cervical	33201	BIOPSIA DE TUMOR INTRADURALES (INTRAMEDULARES Y EXTRAMEDULARES) CERVICALES, DORSALES, LUMBOSACROS O COCCÍGEOS	Screening	diagnostico
Cervical	670101	DILATACIÓN Y CURETAJE DEL MUÑÓN CERVICAL	Screening	diagnostico
Cervical	671201	BIOPSIA EN SACABOCADO DE CUELLO UTERINO	Screening	diagnostico
Cervical	671202	BIOPSIA DE CUELLO UTERINO CIRCUNFERENCIAL	Screening	diagnostico
Cervical	681200	HISTEROSCOPIA	Screening	diagnostico
Cervical	681201	HISTEROSCOPIA	Screening	diagnostico
Cervical	702203	COLPOSCOPIA	Screening	diagnostico
Cervical	457101	COLECTOMIA PARCIAL CON COLOSTOMIA O ILEOSTOMIA Y FISTULA MUCOSA VIA ABIERTA	Spread procedures	diseminacio n
Cervical	461301	COLOSTOMIA VIA ABIERTA	Spread procedures	diseminacio n
Cervical	461302	COLOSTOMIA VIA LAPAROSCOPICA	Spread procedures	diseminacio n
Cervical	552101	NEFROSCOPIA VIA PERCUTANEA	Spread procedures	diseminacio n
Cervical	552101	NEFROSCOPIA VIA PERCUTANEA	Spread procedures	diseminacio n
Cervical	558604	ANASTOMOSIS PIELO-URETERO-VESICAL VÍA LAPAROSCÓPICA	Spread procedures	diseminacio n
Cervical	583203	RESECCIÓN O ABLACIÓN ABIERTA DE LESIÓN O TEJIDO URETRAL	Spread procedures	diseminacio n
Cervical	599110	ESCISIÓN DE TUMOR RETROPERITONEAL CON DISECCIÓN DE GRANDES VASOS	Spread procedures	diseminacio n
Cervical	652301	RESECCIÓN DE TUMOR DE OVARIO POR LAPAROTOMÍA	Spread procedures	diseminacio n
Cervical	652302	RESECCIÓN DE TUMOR DE OVARIO POR LAPAROSCOPIA	Spread procedures	diseminacio n
Cervical	653101	OOFORECTOMÍA UNILATERAL POR LAPAROTOMÍA	Spread procedures	diseminacio n
Cervical	653102	OOFORECTOMÍA UNILATERAL POR LAPAROSCOPIA	Spread procedures	diseminacio n
Cervical	655101	OOFORECTOMÍA BILATERAL POR LAPAROTOMÍA	Spread procedures	diseminacio n
Cervical	655102	OOFORECTOMÍA BILATERAL POR LAPAROSCOPIA	Spread procedures	diseminacio n
Cervical	657801	OOFOROPEXIA UNILATERAL POR LAPAROTOMÍA	Spread procedures	diseminacio n

Cervical	657802	OOFOROPEXIA UNILATERAL POR LAPAROSCOPIA	Spread procedures	diseminacio n
Cervical	657803	OOFOROPEXIA BILATERAL POR LAPAROTOMÍA	Spread procedures	diseminacio n
Cervical	657804	OOFOROPEXIA BILATERAL POR LAPAROSCOPIA	Spread procedures	diseminacio n
Cervical	662100	ABLACIÓN U OCLUSIÓN DE TROMPA DE FALOPIO ÚNICA POR LAPAROTOMÍA	Spread procedures	diseminacio n
Cervical	662101	ABLACIÓN U OCLUSIÓN DE TROMPA DE FALOPIO ÚNICA POR LAPAROTOMÍA	Spread procedures	diseminacio n
Cervical	662200	ABLACIÓN U OCLUSIÓN DE TROMPA DE FALOPIO BILATERAL POR LAPAROTOMÍA	Spread procedures	diseminacio n
Cervical	662201	ABLACIÓN U OCLUSIÓN DE TROMPA DE FALOPIO BILATERAL POR LAPAROTOMÍA	Spread procedures	diseminacio n
Cervical	662202	ABLACIÓN U OCLUSIÓN DE TROMPA DE FALOPIO BILATERAL POR LAPAROSCOPIA	Spread procedures	diseminacio n
Cervical	663100	ABLACIÓN U OCLUSIÓN DE TROMPA DE FALOPIO ÚNICA POR LAPAROTOMÍA	Spread procedures	diseminacio n
Cervical	682510	ABLACIÓN ENDOMETRIAL O ENDOMETRECTOMÍA POR HISTEROSCOPIA	Spread procedures	diseminacio n
Cervical	690101	LEGRADO UTERINO GINECOLÓGICO	Spread procedures	diseminacio n
Cervical	690102	LEGRADO UTERINO GINECOLÓGICO	Spread procedures	diseminacio n
Cervical	690103	LEGRADO UTERINO GINECOLÓGICO	Spread procedures	diseminacio n
Cervical	694101	HISTERORRAFIA POR LAPAROTOMÍA	Spread procedures	diseminacio n
Cervical	702101	VAGINOSCOPIA	Spread procedures	diseminacio n
Cervical	702110	VAGINOSCOPIA	Spread procedures	diseminacio n
Cervical	703302	RESECCIÓN O ABLACIÓN DE LESIÓN O TEJIDO VAGINAL	Spread procedures	diseminacio n
Cervical	703321	RESECCIÓN O ABLACIÓN DE LESIÓN O TEJIDO VAGINAL	Spread procedures	diseminacio n
Cervical	703340	RESECCIÓN DE TUMOR MALIGNO DE VAGINA	Spread procedures	diseminacio n
Cervical	704001	VAGINECTOMÍA O COLPECTOMÍA TOTAL	Spread procedures	diseminacio n
Cervical	704002	VAGINECTOMÍA O COLPECTOMÍA PARCIAL	Spread procedures	diseminacio n
Cervical	704100	VAGINECTOMÍA O COLPECTOMÍA TOTAL	Spread procedures	diseminacio n
Cervical	706102	VAGINOPLASTIA VÍA PERINEAL	Spread procedures	diseminacio n
Cervical	708102	VAGINECTOMÍA O COLPECTOMÍA PARCIAL	Spread procedures	diseminacio n

Cervical	715001	VULVECTOMÍA SUPERFICIAL UNILATERAL	Spread procedures	diseminacio n
Cervical	715002	VULVECTOMÍA SUPERFICIAL BILATERAL	Spread procedures	diseminacio n
Cervical	715003	VULVECTOMÍA SIMPLE UNILATERAL	Spread procedures	diseminacio n
Cervical	715004	VULVECTOMÍA SIMPLE BILATERAL	Spread procedures	diseminacio n
Cervical	715007	VULVECTOMÍA RADICAL	Spread procedures	diseminacio n
Cervical	716120	VULVECTOMÍA SUPERFICIAL UNILATERAL	Spread procedures	diseminacio n
Cervical	750101	LEGRADO UTERINO OBSTÉTRICO POSPARTO O POSABORTO POR DILATACIÓN Y CURETAJE	Spread procedures	diseminacio n
Cervical	750105	LEGRADO UTERINO OBSTÉTRICO POSPARTO O POSABORTO POR ASPIRACIÓN AL VACÍO	Spread procedures	diseminacio n
Cervical	871040	RADIOGRAFIA DE COLUMNA LUMBOSACRA	Spread procedures	diseminacio n
Cervical	871121	RADIOGRAFIA DE TORAX (P.A. O A.P. Y LATERAL, DECUBITO LATERAL, OBLICUAS O LATERAL)	Spread procedures	diseminacio n
Cervical	872002	RADIOGRAFÍA DE ABDOMEN SIMPLE	Spread procedures	diseminacio n
Cervical	872011	RADIOGRAFÍA DE ABDOMEN SIMPLE CON PROYECCIONES ADICIONALES (SERIE DE ABDOMEN AGUDO)	Spread procedures	diseminacio n
Cervical	872105	RADIOGRAFIA DE COLON POR ENEMA CON DOBLE CONTRASTE	Spread procedures	diseminacio n
Cervical	873411	RADIOGRAFIA DE CADERA O ARTICULACION COXO-FEMORAL (AP, LATERAL)	Spread procedures	diseminacio n
Cervical	873412	RADIOGRAFIA DE CADERA COMPARATIVA	Spread procedures	diseminacio n
Cervical	876801	MAMOGRAFIA UNILATERAL O DE PIEZA QUIRURGICA	Spread procedures	diseminacio n
Cervical	876802	MAMOGRAFIA BILATERAL	Spread procedures	diseminacio n
Cervical	877812	PIELOGRAFIA A TRAVES DE TUBO DE NEFROSTOMIA	Spread procedures	diseminacio n
Cervical	877816	PIELOGRAFIA PERCUTANEA	Spread procedures	diseminacio n
Cervical	877901	HISTEROSALPINGOGRAFÍA	Spread procedures	diseminacio n
Cervical	879111	TOMOGRAFIA COMPUTADA DE CRANEO SIMPLE	Spread procedures	diseminacio n
Cervical	879112	TOMOGRAFIA COMPUTADA DE CRANEO CON CONTRASTE	Spread procedures	diseminacio n
Cervical	879113	TOMOGRAFIA COMPUTADA DE CRANEO SIMPLE Y CON CONTRASTE	Spread procedures	diseminacio n
Cervical	879301	TOMOGRAFIA COMPUTADA DE TORAX	Spread procedures	diseminacio n

Cervical	879301	TOMOGRAFIA COMPUTADA DE TORAX	Spread procedures	diseminacio n
Cervical	879391	TOMOGRAFIA COMPUTADA DE TORAX EXTENDIDO AL ABDOMEN SUPERIOR CON SUPRARRENALES	Spread procedures	diseminacio n
Cervical	879410	TOMOGRAFIA COMPUTADA DE ABDOMEN SUPERIOR	Spread procedures	diseminacio n
Cervical	879411	TOMOGRAFIA COMPUTADA DE INTESTINO [ENTEROTC]	Spread procedures	diseminacio n
Cervical	879430	TOMOGRAFIA COMPUTADA DE VIAS URINARIAS [UROTC]	Spread procedures	diseminacio n
Cervical	879431	UROGRAFIA CON TOMOGRAFIA COMPUTADA	Spread procedures	diseminacio n
Cervical	879910	TOMOGRAFIA COMPUTADA EN RECONSTRUCCION TRIDIMENSIONAL	Spread procedures	diseminacio n
Cervical	879990	TOMOGRAFIA COMPUTADA COMO GUIA PARA PROCEDIMIENTOS	Spread procedures	diseminacio n
Cervical	881141	ECOGRAFIA DE TIROIDES CON TRANSDUCTOR DE 7 MHZ O MAS	Spread procedures	diseminacio n
Cervical	881202	ECOCARDIOGRAMA TRANSTORACICO	Spread procedures	diseminacio n
Cervical	881302	ECOGRAFÍA DE ABDOMEN TOTAL (HÍGADO, PÁNCREAS, VESÍCULA, VÍAS BILIARES, RIÑONES, BAZO, GRANDES VASOS, PELVIS Y FLANCOS)	Spread procedures	diseminacio n
Cervical	881305	ECOGRAFÍA DE ABDOMEN SUPERIOR (HÍGADO, PÁNCREAS, VÍAS BILIARES, RIÑONES, BAZO Y GRANDES VASOS)	Spread procedures	diseminacio n
Cervical	881306	ECOGRAFÍA DE HIGADO, PÁNCREAS, VÍA BILIAR Y VESÍCULA	Spread procedures	diseminacio n
Cervical	881312	ECOGRAFÍA ENDOSCÓPICA DE ESÓFAGO	Spread procedures	diseminacio n
Cervical	881313	ECOGRAFÍA DE ABDOMEN (PÍLORO)	Spread procedures	diseminacio n
Cervical	881314	ECOGRAFÍA ENDOSCÓPICA DE ESTÓMAGO O DUODENO	Spread procedures	diseminacio n
Cervical	881317	ECOGRAFÍA ENDOSCÓPICA BILIOPANCREÁTICA	Spread procedures	diseminacio n
Cervical	881318	ECOGRAFÍA DE RECTO	Spread procedures	diseminacio n
Cervical	881319	ECOGRAFÍA ENDOSCÓPICA DE RECTO	Spread procedures	diseminacio n
Cervical	881320	ECOGRAFÍA DE ANO	Spread procedures	diseminacio n
Cervical	881321	ECOGRAFÍA LAPAROSCÓPICA DE ABDOMEN	Spread procedures	diseminacio n
Cervical	881331	ECOGRAFÍA DE RIÑONES, BAZO, AORTA O ADRENALES	Spread procedures	diseminacio n
Cervical	881332	ECOGRAFÍA DE VÍAS URINARIAS (RIÑONES, VEJIGA Y PRÓSTATA TRANSABDOMINAL)	Spread procedures	diseminacio n
Cervical	881333	MEDICIÓN DE EYECCIÓN URETERAL	Spread procedures	diseminacio n

Cervical	881340	ECOGRAFÍA DE ABDOMEN (MASAS ABDOMINALES Y DE RETROPERITONEO)	Spread procedures	diseminacio n
Cervical	881362	ECOGRAFÍA DE TEJIDOS BLANDOS DE ABDOMEN CON ANÁLISIS DOPPLER	Spread procedures	diseminacio n
Cervical	881431	ECOGRAFÍA OBSTÉTRICA TRANSABDOMINAL	Spread procedures	diseminacio n
Cervical	881432	ECOGRAFÍA OBSTÉTRICA TRANSVAGINAL	Spread procedures	diseminacio n
Cervical	881434	PERFIL BIOFÍSICO	Spread procedures	diseminacio n
Cervical	881435	ECOGRAFÍA OBSTÉTRICA CON EVALUACIÓN DE CIRCULACIÓN PLACENTARIA Y FETAL	Spread procedures	diseminacio n
Cervical	881436	ECOGRAFIA OBSTETRICA CON TRANSLUCENCIA NUCAL	Spread procedures	diseminacio n
Cervical	881437	ECOGRAFIA OBSTETRICA CON DETALLE ANATOMICO	Spread procedures	diseminacio n
Cervical	882308	ECOGRAFIA DOPPLER DE VASOS ARTERIALES DE MIEMBROS INFERIORES	Spread procedures	diseminacio n
Cervical	882317	ECOGRAFIA DOPPLER DE VASOS VENOSOS DE MIEMBROS INFERIORES	Spread procedures	diseminacio n
Cervical	882318	ECOGRAFIA DOPPLER DE VASOS VENOSOS DE MIEMBRO INFERIOR	Spread procedures	diseminacio n
Cervical	882840	ECOGRAFIA DOPPLER COMO GUIA PARA PROCEDIMIENTOS	Spread procedures	diseminacio n
Cervical	883101	RESONANCIA MAGNETICA DE CEREBRO	Spread procedures	diseminacio n
Cervical	883210	RESONANCIA MAGNETICA DE COLUMNA CERVICAL SIMPLE	Spread procedures	diseminacio n
Cervical	883220	RESONANCIA MAGNETICA DE COLUMNA TORACICA SIMPLE	Spread procedures	diseminacio n
Cervical	883221	RESONANCIA MAGNETICA DE COLUMNA TORACICA CON CONTRASTE	Spread procedures	diseminacio n
Cervical	883230	RESONANCIA MAGNETICA DE COLUMNA LUMBOSACRA SIMPLE	Spread procedures	diseminacio n
Cervical	883230	RESONANCIA MAGNETICA DE COLUMNA LUMBOSACRA SIMPLE	Spread procedures	diseminacio n
Cervical	883231	RESONANCIA MAGNETICA DE COLUMNA LUMBAR CON CONTRASTE	Spread procedures	diseminacio n
Cervical	883301	RESONANCIA MAGNETICA DEL TORAX	Spread procedures	diseminacio n
Cervical	883401	RESONANCIA MAGNETICA DE ABDOMEN	Spread procedures	diseminacio n
Cervical	883522	RESONANCIA MAGNETICA DE ARTICULACIONES DE MIEMBRO INFERIOR (ESPECIFICO)	Spread procedures	diseminacio n
Cervical	886011	OSTEODENSITOMETRIA POR TC	Spread procedures	diseminacio n
Cervical	886012	OSTEODENSITOMETRIA POR ABSORCION DUAL	Spread procedures	diseminacio n

Cervical	895100	ELECTROCARDIOGRAMA DE RITMO O DE SUPERFICIE SOD	Spread procedures	diseminacio n
Cervical	901235	UROCULTIVO (ANTIBIOGRAMA DE DISCO)	Spread procedures	diseminacio n
Cervical	901236	UROCULTIVO (ANTIBIOGRAMA CONCENTRACION MINIMA INHIBITORIA AUTOMATIZADO)	Spread procedures	diseminacio n
Cervical	901237	UROCULTIVO (ANTIBIOGRAMA CONCENTRACION MINIMA INHIBITORIA MANUAL)	Spread procedures	diseminacio n
Cervical	903103	ACIDO 5 HIDROXI INDOLACETICO EN ORINA DE 24 HORAS	Spread procedures	diseminacio n
Cervical	903856	NITROGENO UREICO	Spread procedures	diseminacio n
Cervical	903857	NITROGENO UREICO EN ORINA DE 24 HORAS	Spread procedures	diseminacio n
Cervical	903866	TRANSAMINASA GLUTAMICO-PIRUVICA [ALANINO AMINO TRANSFERASA]	Spread procedures	diseminacio n
Cervical	903867	TRANSAMINASA GLUTAMICO OXALACETICA [ASPARTATO AMINO TRANSFERASA]	Spread procedures	diseminacio n
Cervical	903895	CREATININA EN SUERO U OTROS FLUIDOS	Spread procedures	diseminacio n
Cervical	976102	EXTRACCION DE DISPOSITIVO DE NEFROSTOMIA	Spread procedures	diseminacio n
Cervical	683101	HISTERECTOMIA SUBTOTAL O SUPRACERVICAL POR LAPAROTOMIA	Hysterectomy	histerectomi a
Cervical	683111	HISTERECTOMIA SUBTOTAL O SUPRACERVICAL, POR LAPAROSCOPIA	Hysterectomy	histerectomi a
Cervical	684001	HISTERECTOMIA TOTAL ABDOMINAL CON REMOCION DE MOLA O FETO MUERTO POR LAPAROTOMIA	Hysterectomy	histerectomi a
Cervical	684002	HISTERECTOMÍA TOTAL POR LAPAROTOMÍA	Hysterectomy	histerectomi a
Cervical	684003	HISTERECTOMIA TOTAL POR LAPAROTOMIA	Hysterectomy	histerectomi a
Cervical	684020	HISTERECTOMIA TOTAL POR LAPAROSCOPIA	Hysterectomy	histerectomi a
Cervical	684101	HISTERECTOMÍA TOTAL ABDOMINAL AMPLIADA POR LAPAROTOMÍA	Hysterectomy	histerectomi a
Cervical	684102	EXTIRPACIÓN TOTAL DE ÚTERO [HISTERECTOMÍA] ABDOMINAL AMPLIADA	Hysterectomy	histerectomi a
Cervical	684103	HISTERECTOMIA TOTAL ABDOMINAL AMPLIADA POR LAPAROTOMIA	Hysterectomy	histerectomi a
Cervical	684104	HISTERECTOMIA TOTAL ABDOMINAL AMPLIADA POR LAPAROSCOPIA	Hysterectomy	histerectomi a
Cervical	685101	HISTERECTOMÍA POR VÍA VAGINAL	Hysterectomy	histerectomi a
Cervical	685102	HISTERECTOMIA POR VIA VAGINAL	Hysterectomy	histerectomi a
Cervical	685130	HISTERECTOMÍA POR VÍA VAGINAL	Hysterectomy	histerectomi a

Cervical	686001	HISTERECTOMIA RADICAL POR LAPAROTOMIA	Hysterectomy	histerectomi a
Cervical	686011	HISTERECTOMIA RADICAL POR LAPAROSCOPIA	Hysterectomy	histerectomi a
Cervical	686101	HISTERECTOMÍA RADICAL MODIFICADA POR LAPAROTOMÍA	Hysterectomy	histerectomi a
Cervical	686102	HISTERECTOMÍA RADICAL MODIFICADA POR LAPAROTOMÍA	Hysterectomy	histerectomi a
Cervical	686110	HISTERECTOMIA RADICAL MODIFICADA POR LAPAROSCOPIA	Hysterectomy	histerectomi a
Cervical	687000	HISTERECTOMÍA RADICAL POR VÍA VAGINAL	Hysterectomy	histerectomi a
Cervical	687001	HISTERECTOMIA RADICAL POR VIA VAGINAL	Hysterectomy	histerectomi a
Cervical	688101	EXENTERACIÓN O EVISCERACIÓN PÉLVICA FEMENINA TOTAL O COMPLETA	Hysterectomy	histerectomi a
Cervical	688201	EXENTERACIÓN ANTERIOR: ÚTERO Y VEJIGA	Hysterectomy	histerectomi a
Cervical	688301	EXENTERACIÓN POSTERIOR: ÚTERO Y RECTO	Hysterectomy	histerectomi a
Cervical	879201	TOMOGRAFIA COMPUTADA DE COLUMNA SEGMENTOS CERVICAL, TORACICO, LUMBAR O SACRO, POR CADA NIVEL (TRES ESPACIOS)	Diagnostic imaging	imag_diagno s
Cervical	879420	TOMOGRAFIA COMPUTADA DE ABDOMEN Y PELVIS (ABDOMEN TOTAL)	Diagnostic imaging	imag_diagno s
Cervical	879460	TOMOGRAFIA COMPUTADA DE PELVIS	Diagnostic imaging	imag_diagno s
Cervical	879601	TOMOGRAFIA POR EMISION DE POSITRONES [PET-TC]	Diagnostic imaging	imag_diagno s
Cervical	881301	ECOGRAFÍA DE TEJIDOS BLANDOS DE PARED ABDOMINAL Y DE PELVIS	Diagnostic imaging	imag_diagno s
Cervical	881360	ECOGRAFÍA PELVICA CON ANÁLISIS DOPPLER	Diagnostic imaging	imag_diagno s
Cervical	881390	ECOGRAFÍA DEL ABDOMEN Y PELVIS COMO GUÍA DE PROCEDIMIENTO QUIRÚRGICO O INTERVENCIONISTA	Diagnostic imaging	imag_diagno s
Cervical	881401	ECOGRAFIA PELVICA GINECOLOGICA TRANSVAGINAL	Diagnostic imaging	imag_diagno s
Cervical	881402	ECOGRAFIA PELVICA GINECOLOGICA TRANSABDOMINAL	Diagnostic imaging	imag_diagno s
Cervical	881403	ECOGRAFÍA PÉLVICA GINECOLÓGICA (ESTUDIO INTEGRAL FOLICULAR CON ECO VAGINAL)	Diagnostic imaging	imag_diagno s
Cervical	881410	ECOGRAFÍA PÉLVICA GINECOLÓGICA (HISTEROSONOGRAFÍA O HISTEROSALPINGOSONOGRAFÍA)	Diagnostic imaging	imag_diagno s
Cervical	881411	ECOGRAFÍA DINÁMICA DE PISO PÉLVICO	Diagnostic imaging	imag_diagno s
Cervical	881412	ECOGRAFÍA DE MAPEO PÉLVICO	Diagnostic imaging	imag_diagno s
Cervical	883440	RESONANCIA MAGNETICA DE PELVIS	Diagnostic imaging	imag_diagno s
Cervical	890602	CUIDADO (MANEJO) INTRAHOSPITALARIO POR MEDICINA ESPECIALIZADA	Inpatient services	internacion

Cervical	107M01	INTERNACIÓN EN UNIDAD DE CUIDADO INTERMEDIO ADULTO	Inpatient services	internacion
Cervical	10A001	INTERNACIÓN COMPLEJIDAD ALTA HABITACION UNIPERSONAL (INCLUYE AISLAMIENTO)	Inpatient services	internacion
Cervical	10A002	INTERNACIÓN COMPLEJIDAD ALTA HABITACION BIPERSONAL	Inpatient services	internacion
Cervical	10A003	INTERNACIÓN COMPLEJIDAD ALTA TRES CAMAS	Inpatient services	internacion
Cervical	10A004	INTERNACIÓN COMPLEJIDAD ALTA CUATRO O MÁS CAMAS	Inpatient services	internacion
Cervical	10A005	INTERNACIÓN EN UNIDAD DE TRASPLANTE	Inpatient services	internacion
Cervical	10M001	INTERNACIÓN COMPLEJIDAD MEDIANA HABITACION UNIPERSONAL (INCLUYE AISLAMIENTO)	Inpatient services	internacion
Cervical	10M002	INTERNACIÓN COMPLEJIDAD MEDIANA HABITACION BIPERSONAL	Inpatient services	internacion
Cervical	10M003	INTERNACIÓN COMPLEJIDAD MEDIANA HABITACION TRES CAMAS	Inpatient services	internacion
Cervical	10M004	INTERNACIÓN COMPLEJIDAD MEDIANA HABITACION CUATRO O MÁS CAMAS	Inpatient services	internacion
Cervical	110A01	INTERNACIÓN EN UNIDAD DE CUIDADO INTENSIVO ADULTOS	Inpatient services	internacion
Cervical	121M01	INTERNACIÓN DE PACIENTE CRÓNICO TERMINAL SIN VENTILADOR COMPLEJIDAD MEDIANA	Inpatient services	internacion
Cervical	121M02	INTERNACIÓN DE PACIENTE CRÓNICO TERMINAL CON VENTILADOR COMPLEJIDAD MEDIANA	Inpatient services	internacion
Cervical	S11201	INTERNACIÓN COMPLEJIDAD MEDIANA HABITACION UNIPERSONAL (INCLUYE AISLAMIENTO)	Inpatient services	internacion
Cervical	S11202	INTERNACIÓN COMPLEJIDAD MEDIANA HABITACION BIPERSONAL	Inpatient services	internacion
Cervical	S11203	INTERNACIÓN COMPLEJIDAD MEDIANA HABITACION TRES CAMAS	Inpatient services	internacion
Cervical	S11204	INTERNACIÓN COMPLEJIDAD MEDIANA HABITACION CUATRO O MÁS CAMAS	Inpatient services	internacion
Cervical	S11301	INTERNACIÓN COMPLEJIDAD ALTA HABITACION UNIPERSONAL (INCLUYE AISLAMIENTO)	Inpatient services	internacion
Cervical	S11302	INTERNACIÓN COMPLEJIDAD ALTA HABITACION BIPERSONAL	Inpatient services	internacion
Cervical	S11303	INTERNACIÓN COMPLEJIDAD ALTA TRES CAMAS	Inpatient services	internacion
Cervical	S11304	INTERNACIÓN COMPLEJIDAD ALTA CUATRO O MÁS CAMAS	Inpatient services	internacion
Cervical	S12103	INTERNACIÓN EN UNIDAD DE CUIDADO INTENSIVO ADULTOS	Inpatient services	internacion
Cervical	S12203	INTERNACIÓN EN UNIDAD DE CUIDADO INTERMEDIO ADULTO	Inpatient services	internacion
Cervical	S12600	INTERNACIÓN EN UNIDAD DE TRASPLANTE	Inpatient services	internacion
Cervical	892904	TECNICAS DE INSPECCION VISUAL CON ACIDO ACETICO Y LUGOL	Laboratory tests	laboratorio
Cervical	898002	ESTUDIO DE COLORACION BASICA EN CITOLOGIA DE LIQUIDO CORPORAL O SECRECION	Laboratory tests	laboratorio
Cervical	898003	ESTUDIO DE COLORACION BASICA EN CITOLOGIA POR ASPIRACION DE CUALQUIER TEJIDO U ORGANO [BACAF]	Laboratory tests	laboratorio
Cervical	898006	ESTUDIO DE COLORACION HISTOQUIMICA EN CITOLOGIA DE LIQUIDO CORPORAL O SECRECION	Laboratory tests	laboratorio
Cervical	898007	ESTUDIO DE COLORACION HISTOQUIMICA EN CITOLOGIA POR ASPIRACION DE CUALQUIER TEJIDO U ORGANO [BACAF]	Laboratory tests	laboratorio
Cervical	898010	ESTUDIO DE COLORACION INMUNOHISTOQUIMICA EN CITOLOGIA DE LIQUIDO CORPORAL O SECRECION	Laboratory tests	laboratorio
Cervical	898011	ESTUDIO DE COLORACIÓN INMUNOHISTOQUÍMICA EN CITOLOGÍA POR ASPIRACIÓN DE CUALQUIER TEJIDO U ÓRGANO [BACAF]	Laboratory tests	laboratorio
Cervical	898014	ESTUDIO DE COLORACIÓN DE INMUNOFLUORESCENCIA EN CITOLOGÍA DE LÍQUIDO CORPORAL O SECRECIÓN	Laboratory tests	laboratorio
Cervical	898017	ESTUDIO ANATOMOPATOLÓGICO EN CITOLOGÍA POR TINCIÓN DE HISTOQUÍMICA (ESPECÍFICO)	Laboratory tests	laboratorio
Cervical	898018	ESTUDIO ANATOMOPATOLÓGICO EN CITOLOGÍA O MIELOGRAMA POR INMUNOHISTOQUÍMICA (MARCADOR ESPECÍFICO)	Laboratory tests	laboratorio
Cervical	898019	ESTUDIO ANATOMOPATOLÓGICO BÁSICO EN CITOLOGÍA DE OTRO ESPÉCIMEN POR MÉTODO MANUAL	Laboratory tests	laboratorio
Cervical	898020	ESTUDIO ANATOMOPATOLÓGICO BÁSICO EN CITOLOGÍA DE OTRO ESPÉCIMEN POR MÉTODO SEMIAUTOMATIZADO O AUTOMATIZADO	Laboratory tests	laboratorio
Cervical	898030	ESTUDIO DE BIOLOGÍA MOLECULAR EN CITOLOGÍA	Laboratory tests	laboratorio
Cervical	898031	ESTUDIO DE CITOMETRÍA DE FLUJO EN CITOLOGÍA	Laboratory tests	laboratorio
Cervical	898101	ESTUDIO DE COLORACION BASICA EN BIOPSIA	Laboratory tests	laboratorio
Cervical	898102	ESTUDIO DE COLORACION HISTOQUIMICA EN BIOPSIA	Laboratory tests	laboratorio
Cervical	898103	ESTUDIO DE COLORACION INMUNOHISTOQUIMICA EN BIOPSIA	Laboratory tests	laboratorio
Cervical	898201	ESTUDIO DE COLORACION BASICA EN ESPECIMEN DE RECONOCIMIENTO	Laboratory tests	laboratorio
Cervical	898202	ESTUDIO DE COLORACION HISTOQUIMICA EN ESPECIMEN DE RECONOCIMIENTO	Laboratory tests	laboratorio
Cervical	898203	ESTUDIO DE COLORACION INMUNOHISTOQUIMICA EN ESPECIMEN DE RECONOCIMIENTO	Laboratory tests	laboratorio

Cervical	898206	ESTUDIO DE CITOMETRIA DE FLUJO EN ESPECIMEN DE RECONOCIMIENTO	Laboratory tests	laboratorio
Cervical	898221	ESTUDIO DE COLORACION BASICA EN ESPECIMEN CON MULTIPLE MUESTREO	Laboratory tests	laboratorio
Cervical	898222	ESTUDIO DE COLORACION HISTOQUIMICA EN ESPECIMEN CON MULTIPLE MUESTREO	Laboratory tests	laboratorio
Cervical	898241	ESTUDIO DE COLORACION BASICA EN ESPECIMEN CON RESECCION DE MARGENES	Laboratory tests	laboratorio
Cervical	898242	ESTUDIO DE COLORACION HISTOQUIMICA EN ESPECIMEN CON RESECCION DE MARGENES	Laboratory tests	laboratorio
Cervical	898243	ESTUDIO DE COLORACION INMUNOHISTOQUIMICA EN ESPECIMEN CON RESECCION DE MARGENES	Laboratory tests	laboratorio
Cervical	903822	CREATINA	Laboratory tests	laboratorio
Cervical	903823	CREATININA DEPURACION	Laboratory tests	laboratorio
Cervical	903823	CREATININA DEPURACION	Laboratory tests	laboratorio
Cervical	903824	CREATININA EN ORINA DE 24 HORAS	Laboratory tests	laboratorio
Cervical	906603	ANTIGENO CARCINOEMBRIONARIO SEMIAUTOMATIZADO O AUTOMATIZADO	Laboratory tests	laboratorio
Cervical	906605	ANTIGENO DE CANCER DE OVARIO [CA 125] SEMIAUTOMATIZADO O AUTOMATIZADO	Laboratory tests	laboratorio
Cervical	908436	DETECCIÓN Virus del Papiloma Humano PRUEBAS DE ADN	Laboratory tests	laboratorio
Cervical	920805	GAMAGRAFIA DE FILTRACION GLOMERULAR	Laboratory tests	laboratorio
Cervical	405101	VACIAMIENTO RADICAL LINFÁTICO AXILAR VÍA ABIERTA	Lymphadenectomy	linfadenecto mia
Cervical	405201	VACIAMIENTO RADICAL LINFÁTICO (LINFADENECTOMÍA) DE MEDIASTINO VÍA ABIERTA	Lymphadenectomy	linfadenecto mia
Cervical	405202	VACIAMIENTO RADICAL LINFÁTICO (LINFADENECTOMÍA) DE MEDIASTINO VÍA TORACOSCÓPICA	Lymphadenectomy	linfadenecto mia
Cervical	405202	VACIAMIENTO RADICAL LINFÁTICO (LINFADENECTOMÍA) DE MEDIASTINO VÍA TORACOSCÓPICA	Lymphadenectomy	linfadenecto mia
Cervical	405203	VACIAMIENTO RADICAL LINFÁTICO (LINFADENECTOMÍA) DE MEDIASTINO POR MEDIASTINOSCOPIA	Lymphadenectomy	linfadenecto mia
Cervical	405301	LINFADENECTOMIA RADICAL INGUINOFEMORAL, UNILATERAL	Lymphadenectomy	linfadenecto mia
Cervical	405302	LINFADENECTOMIA RADICAL INGUINOFEMORAL O ILIACA BILATERAL	Lymphadenectomy	linfadenecto mia
Cervical	405304	LINFADENECTOMÍA RADICAL INGUINOFEMORAL, UNILATERAL VÍA ABIERTA	Lymphadenectomy	linfadenecto mia
Cervical	405305	LINFADENECTOMÍA RADICAL INGUINOFEMORAL, UNILATERAL VÍA LAPAROSCÓPICA	Lymphadenectomy	linfadenecto mia
Cervical	405306	LINFADENECTOMÍA RADICAL INGUINOILÍACO BILATERAL VÍA ABIERTA	Lymphadenectomy	linfadenecto mia
Cervical	405307	LINFADENECTOMÍA RADICAL INGUINOILÍACO BILATERAL VÍA LAPAROSCÓPICA	Lymphadenectomy	linfadenecto mia
Cervical	405401	LINFADENECTOMIA RADICAL PELVICA	Lymphadenectomy	linfadenecto mia
Cervical	405402	LINFADENECTOMIA RADICAL EXTRAPERITONEAL	Lymphadenectomy	linfadenecto mia
Cervical	405404	LINFADENECTOMÍA RADICAL ABDOMINAL VÍA ABIERTA	Lymphadenectomy	linfadenecto mia
Cervical	405405	LINFADENECTOMÍA RADICAL ABDOMINAL VÍA LAPAROSCÓPICA	Lymphadenectomy	linfadenecto mia
Cervical	405406	LINFADENECTOMÍA RADICAL PÉLVICA VÍA ABIERTA	Lymphadenectomy	linfadenecto mia
Cervical	405407	LINFADENECTOMÍA RADICAL PÉLVICA VÍA LAPAROSCÓPICA	Lymphadenectomy	linfadenecto mia

Cervical	405408	LINFADENECTOMÍA RADICAL EXTRAPERITONEAL VÍA ABIERTA	Lymphadenectomy	linfadenecto mia
Cervical	405409	LINFADENECTOMÍA RADICAL EXTRAPERITONEAL VÍA LAPAROSCÓPICA	Lymphadenectomy	linfadenecto mia
Cervical	405411	LINFADENECTOMÍA RADICAL ABDOMINO INGUINAL VÍA ABIERTA	Lymphadenectomy	linfadenecto mia
Cervical	405412	LINFADENECTOMÍA RADICAL ABDOMINO INGUINAL VÍA LAPAROSCÓPICA	Lymphadenectomy	linfadenecto mia
Cervical	405501	RESECCION RADICAL DE GANGLIOS LINFATICOS RETROPERITONEALES	Lymphadenectomy	linfadenecto mia
Cervical	405502	RESECCIÓN RADICAL DE GANGLIOS LINFÁTICOS RETROPERITONEALES VÍA ABIERTA	Lymphadenectomy	linfadenecto mia
Cervical	405503	LINFADENECTOMÍA RETROPERITONEAL VÍA LAPAROSCÓPICA	Lymphadenectomy	linfadenecto mia
Cervical	405601	VACIAMIENTO RADICAL EPITROCLEAR VÍA ABIERTA	Lymphadenectomy	linfadenecto mia
Cervical	405602	VACIAMIENTO RADICAL POPLITEO VÍA ABIERTA	Lymphadenectomy	linfadenecto mia
Cervical	992503	MONOTERAPIA ANTINEOPLÁSICA DE BAJA TOXICIDAD	Monoterapy	monoterapia
Cervical	992504	POLITERAPIA ANTINEOPLÁSICA DE BAJA TOXICIDAD	Polyterapy	politerapia
Cervical	992505	POLITERAPIA ANTINEOPLÁSICA DE ALTA TOXICIDAD	Polyterapy	politerapia
Cervical	992506	ADMINISTRACIÓN (INFUSIÓN O PERFUSIÓN) DE TERAPIA ANTINEOPLÁSICA INTRARTERIAL (REGIONAL)	Monoterapy	monoterapia
Cervical	992509	MONOTERAPIA ANTINEOPLÁSICA DE BAJA TOXICIDAD	Monoterapy	monoterapia
Cervical	992511	MONOTERAPIA ANTINEOPLÁSICA DE ALTA TOXICIDAD	Monoterapy	monoterapia
Cervical	922201	TELETERAPIA CON ORTOVOLTAJE	Teletherapy	teleterapia
Cervical	922321	TELETERAPIA CON COBALTO (PLANEACIÓN COMPUTARIZADA BIDIMENSIONAL Y SIMULACIÓN CONVENCIONAL)	Teletherapy	teleterapia
Cervical	922322	TELETERAPIA CON COBALTO (PLANEACIÓN COMPUTARIZADA TRIDIMENSIONAL Y SIMULACIÓN VIRTUAL)	Teletherapy	teleterapia
Cervical	922441	TELETERAPIA CON ACELERADOR LINEAL (PLANEACIÓN COMPUTARIZADA BIDIMENSIONAL Y SIMULACIÓN CONVENCIONAL) TÉCNICA RADIOTERAPIA CONVENCIONAL	Teletherapy	teleterapia
Cervical	922442	TELETERAPIA CON ACELERADOR LINEAL (PLANEACIÓN COMPUTARIZADA TRIDIMENSIONAL Y SIMULACIÓN VIRTUAL) TÉCNICA RADIOTERAPIA CONVENCIONAL	Teletherapy	teleterapia
Cervical	922443	TELETERAPIA CON ACELERADOR LINEAL (PLANEACION COMPUTARIZADA TRIDIMENSIONAL Y SIMULACION VIRTUAL) TECNICA CONFORMACIONAL [3D - CRT]	Teletherapy	teleterapia
Cervical	922444	TELETERAPIA CON ACELERADOR LINEAL (PLANEACIÓN COMPUTARIZADA TRIDIMENSIONAL Y SIMULACIÓN VIRTUAL) TÉCNICA RADIOTERAPIA DE INTENSIDAD MODULADA [IMRT]	Teletherapy	teleterapia
Cervical	922445	TELETERAPIA CON ACELERADOR LINEAL (PLANEACIÓN COMPUTARIZADA TRIDIMENSIONAL Y SIMULACIÓN VIRTUAL) TÉCNICA RADIOTERAPIA GUIADA POR IMÁGENES [IGRT]	Teletherapy	teleterapia
Cervical	922446	TELETERAPIA CON ACELERADOR LINEAL (PLANEACIÓN COMPUTARIZADA TRIDIMENSIONAL Y SIMULACIÓN VIRTUAL) TÉCNICA RADIOTERAPIA - ARCOTERAPIA DE MODULACIÓN VOLUMÉTRICA [VMAT]	Teletherapy	teleterapia
Cervical	922447	IRRADIACIÓN CORPORAL TOTAL	Irradiation	irradiacion
Cervical	922448	IRRADIACIÓN CUTÁNEA TOTAL	Irradiation	irradiacion
Cervical	922449	TELETERAPIA CON ACELERADOR LINEAL (PLANEACIÓN COMPUTARIZADA TRIDIMENSIONAL Y SIMULACIÓN VIRTUAL) TÉCNICA RADIOTERAPIA HELICOIDAL	Teletherapy	teleterapia
Cervical	922504	TELETERAPIA CON ACELERADOR LINEAL DE ELECTRONES (PLANEACION COMPUTARIZADA BIDIMENSIONAL Y SIMULACION CONVENCIONAL)	Teletherapy	teleterapia
Cervical	922505	TELETERAPIA CON ACELERADOR LINEAL DE ELECTRONES (PLANEACION COMPUTARIZADA TRIDIMENSIONAL Y SIMULACIÓN VIRTUAL)	Teletherapy	teleterapia
Cervical	922506	RADIOTERAPIA INTRAOPERATORIA	Teletherapy	teleterapia

Cervical	922601	BRAQUITERAPIA INTERSTICIAL (PLANEACION COMPUTARIZADA TRIDIMENSIONAL Y SIMULACION VIRTUAL) CON ALTA TASA DE DOSIS	Brachytherapy	braquiterapi a
Cervical	922602	BRAQUITERAPIA INTERSTICIAL (PLANEACION COMPUTARIZADA TRIDIMENSIONAL Y SIMULACIÓN VIRTUAL) CON BAJA TASA DE DOSIS	Brachytherapy	braquiterapi a
Cervical	922603	BRAQUITERAPIA INTRALUMINAL (PLANEACION COMPUTARIZADA BIDIMENSIONAL Y SIMULACION CONVENCIONAL) CON ALTA TASA DE DOSIS	Brachytherapy	braquiterapi a
Cervical	922604	BRAQUITERAPIA INTRALUMINAL CON BAJA TASA DE DOSIS	Brachytherapy	braquiterapi a
Cervical	922605	BRAQUITERAPIA INTRACAVITARIA (PLANEACION COMPUTARIZADA BIDIMENSIONAL Y SIMULACION CONVENCIONAL) CON ALTA TASA DE DOSIS	Brachytherapy	braquiterapi a
Cervical	922606	BRAQUITERAPIA INTRACAVITARIA (PLANEACION COMPUTARIZADA BIDIMENSIONAL Y SIMULACION CONVENCIONAL) CON BAJA TASA DE DOSIS	Brachytherapy	braquiterapi a
Cervical	922607	BRAQUITERAPIA INTRACAVITARIA (PLANEACION COMPUTARIZADA TRIDIMENSIONAL Y SIMULACION VIRTUAL) CON ALTA TASA DE DOSIS	Brachytherapy	braquiterapi a
Cervical	922608	BRAQUITERAPIA INTRACAVITARIA (PLANEACIÓN COMPUTARIZADA TRIDIMENSIONAL Y SIMULACIÓN VIRTUAL) CON BAJA TASA DE DOSIS	Brachytherapy	braquiterapi a
Cervical	922609	BRAQUITERAPIA INTRALUMINAL (PLANEACIÓN COMPUTARIZADA TRIDIMENSIONAL Y SIMULACIÓN VIRTUAL) CON ALTA TASA DE DOSIS	Brachytherapy	braquiterapi a
Cervical	922611	BRAQUITERAPIA DE CONTACTO (PLANEACION COMPUTARIZADA BIDIMENSIONAL Y SIMULACION CONVENCIONAL) CON ALTA TASA DE DOSIS	Brachytherapy	braquiterapi a
Cervical	922612	BRAQUITERAPIA DE CONTACTO (PLANEACIÓN COMPUTARIZADA TRIDIMENSIONAL Y SIMULACIÓN VIRTUAL) CON ALTA TASA DE DOSIS	Brachytherapy	braquiterapi a
Cervical	922613	BRAQUITERAPIA EPIESCLERAL DE CONTACTO (PLANEACIÓN COMPUTARIZADA TRIDIMENSIONAL Y SIMULACIÓN VIRTUAL) CON BAJA TASA DE DOSIS	Brachytherapy	braquiterapi a
Cervical	922614	BRAQUITERAPIA METABÓLICA	Brachytherapy	braquiterapi a
Cervical	922615	BRAQUITERAPIA INTERSTICIAL (PLANEACIÓN COMPUTARIZADA BIDIMENSIONAL Y SIMULACIÓN CONVENCIONAL) CON ALTA TASA DE DOSIS	Brachytherapy	braquiterapi a
Cervical	922616	BRAQUITERAPIA INTERSTICIAL (PLANEACIÓN COMPUTARIZADA TRIDIMENSIONAL Y SIMULACIÓN VIRTUAL) CON ALTA TASA DE DOSIS	Brachytherapy	braquiterapi a
Cervical	922800	TERAPIA CON RADIOISOTOPOS SOD	Radiotherapy	terapia
Cervical	922801	TERAPIA CON RADIOISÓTOPOS	Radiotherapy	terapia
Cervical	922805	TERAPIA CON ITRIUM 90	Radiotherapy	terapia
Cervical	922810	TERAPIA CON METAIODOBENCILGUANIDINA SUPRARRENAL	Radiotherapy	terapia
Cervical	922830	TERAPIA DE METÁSTASIS CON ESTRONCIO	Radiotherapy	terapia
Colorectal	454201	RESECCION ENDOSCOPICA DE LESIONES EN SIGMOIDE	Cecectomy	cecectomia
Colorectal	457200	CECECTOMIA SOD	Cecectomy	cecectomia
Colorectal	457201	CECECTOMÍA VÍA ABIERTA	Cecectomy	cecectomia
Colorectal	457202	CECECTOMÍA VÍA LAPAROSCÓPICA	Cecectomy	cecectomia
Colorectal	457600	SIGMOIDECTOMIA SOD	Cecectomy	cecectomia
Colorectal	457601	SIGMOIDECTOMIA VÍA ABIERTA	Cecectomy	cecectomia
Colorectal	457602	SIGMOIDECTOMIA VÍA LAPAROSCÓPICA	Cecectomy	cecectomia
Colorectal	454101	RESECCIÓN DE LESIÓN O TEJIDO DE INTESTINO GRUESO VÍA ABIERTA	Colectomy	colectomia
Colorectal	454102	RESECCIÓN DE LESIÓN O TEJIDO DE INTESTINO GRUESO VÍA LAPAROSCÓPICA	Colectomy	colectomia
Colorectal	454202	RESECCION ENDOSCOPICA DE LESIONES DE COLON	Colectomy	colectomia
Colorectal	454205	MUCOSECTOMÍA DE COLON O RECTO VÍA ENDOSCÓPICA	Colectomy-related	rel_colectom ia
Colorectal	454206	DISECCIÓN DE LA SUBMUCOSA EN COLON O RECTO VÍA ENDOSCÓPICA	Colectomy-related	rel_colectom ia

Colorectal	454207	RESECCIÓN DE LESIÓN DE INTESTINO GRUESO VÍA ENDOSCÓPICA (1- 3)	Colectomy	colectomia
Colorectal	454208	RESECCIÓN DE LESIÓN DE INTESTINO GRUESO VÍA ENDOSCÓPICA (4 - 10)	Colectomy	colectomia
Colorectal	454209	RESECCIÓN DE LESIÓN DE INTESTINO GRUESO VÍA ENDOSCÓPICA (11 O MÁS)	Colectomy	colectomia
Colorectal	454210	ABLACIÓN DE LESIÓN EN INTESTINO GRUESO VÍA ENDOSCÓPICA	Colectomy-related	rel_colectom ia
Colorectal	457000	COLECTOMIA PARCIAL CON COLOSTOMIA Y CIERRE DE SEGMENTO DISTAL [HARTMAN] SOD	Colectomy	colectomia
Colorectal	457001	COLECTOMÍA PARCIAL CON COLOSTOMIA Y CIERRE DE SEGMENTO DISTAL [HARTMAN] VÍA ABIERTA	Colectomy	colectomia
Colorectal	457002	COLECTOMÍA PARCIAL CON COLOSTOMIA Y CIERRE DE SEGMENTO DISTAL [HARTMAN] VÍA LAPAROSCÓPICA	Colectomy	colectomia
Colorectal	457101	COLECTOMÍA PARCIAL CON COLOSTOMÍA O ILEOSTOMÍA Y FÍSTULA MUCOSA VÍA ABIERTA	Colectomy	colectomia
Colorectal	457102	COLECTOMÍA PARCIAL CON COLOSTOMÍA O ILEOSTOMÍA Y FÍSTULA MUCOSA VÍA LAPAROSCÓPICA	Colectomy	colectomia
Colorectal	457300	HEMICOLECTOMIA DERECHA SOD	Colectomy	colectomia
Colorectal	457301	HEMICOLECTOMIA DERECHA VIA ABIERTA	Colectomy	colectomia
Colorectal	457302	HEMICOLECTOMIA DERECHA VIA LAPAROSCOPICA	Colectomy	colectomia
Colorectal	457400	RESECCION DE COLON TRANSVERSO SOD	Colectomy	colectomia
Colorectal	457401	RESECCIÓN DE COLON TRANSVERSO VÍA ABIERTA	Colectomy	colectomia
Colorectal	457402	RESECCIÓN DE COLON TRANSVERSO VÍA LAPAROSCÓPICA	Colectomy	colectomia
Colorectal	457500	HEMICOLECTOMIA IZQUIERDA SOD	Colectomy	colectomia
Colorectal	457501	HEMICOLECTOMIA IZQUIERDA VIA ABIERTA	Colectomy	colectomia
Colorectal	457502	HEMICOLECTOMIA IZQUIERDA VIA LAPAROSCOPICA	Colectomy	colectomia
Colorectal	457901	RESECCION PARCIAL DE COLON POR LAPAROSCOPIA	Colectomy	colectomia
Colorectal	452301	COLONOSCOPIA TOTAL	Colonoscopy	colonoscopi a
Colorectal	452302	COLONOSCOPIA TOTAL	Colonoscopy	colonoscopi a
Colorectal	452303	COLONOSCOPIA CON MAGNIFICACION O CROMOENDOSCOPICA	Colonoscopy	colonoscopi a
Colorectal	452304	MARCACIÓN DE LESIÓN EN COLON VÍA ENDOSCÓPICA	Colonoscopy	colonoscopi a
Colorectal	452305	COLONOSCOPIA TOTAL CON O SIN BIOPSIA	Colonoscopy	colonoscopi a
Colorectal	890234	CONSULTA DE PRIMERA VEZ POR ESPECIALISTA EN CIRUGÍA GASTROINTESTINAL	Surgical consultation	cons_cirugia
Colorectal	890237	CONSULTA DE PRIMERA VEZ POR ESPECIALISTA EN CIRUGÍA ONCOLÓGICA	Surgical consultation	cons_cirugia
Colorectal	890241	CONSULTA DE PRIMERA VEZ POR ESPECIALISTA EN COLOPROCTOLOGIA	Surgical consultation	cons_cirugia
Colorectal	890243	CONSULTA DE PRIMERA VEZ POR ESPECIALISTA EN DOLOR Y CUIDADOS PALIATIVOS	Palliative care consultation	cons_paliati vo
Colorectal	890248	CONSULTA DE PRIMERA VEZ POR ESPECIALISTA EN GENETICA MEDICA	Follow-up consultation	cons_seguim
Colorectal	890246	CONSULTA DE PRIMERA VEZ POR ESPECIALISTA EN GASTROENTEROLOGÍA	Follow-up consultation	cons_seguim
Colorectal	890278	CONSULTA DE PRIMERA VEZ POR ESPECIALISTA EN ONCOLOGÍA	Follow-up consultation	cons_seguim
Colorectal	890287	CONSULTA DE PRIMERA VEZ POR ESPECIALISTA EN RADIOTERAPIA	Radiology consultation	cons_radio
Colorectal	890334	CONSULTA DE CONTROL O DE SEGUIMIENTO POR ESPECIALISTA EN CIRUGÍA GASTROINTESTINAL	Surgical consultation	cons_cirugia

Colorectal	890337	CONSULTA DE CONTROL O DE SEGUIMIENTO POR ESPECIALISTA EN CIRUGÍA ONCOLÓGICA	Surgical consultation	cons_cirugia
Colorectal	890341	CONSULTA DE CONTROL O DE SEGUIMIENTO POR ESPECIALISTA EN COLOPROCTOLOGÍA	Surgical consultation	cons_cirugia
Colorectal	890343	CONSULTA DE CONTROL O DE SEGUIMIENTO POR ESPECIALISTA EN DOLOR Y CUIDADOS PALIATIVOS	Palliative care consultation	cons_paliativo
Colorectal	890348	CONSULTA DE CONTROL O DE SEGUIMIENTO POR ESPECIALISTA EN GENÉTICA MÉDICA	Follow-up consultation	cons_seguim
Colorectal	890346	CONSULTA DE CONTROL O DE SEGUIMIENTO POR ESPECIALISTA EN GASTROENTEROLOGÍA	Follow-up consultation	cons_seguim
Colorectal	890378	CONSULTA DE CONTROL O DE SEGUIMIENTO POR ESPECIALISTA EN ONCOLOGÍA	Follow-up consultation	cons_seguim
Colorectal	890387	CONSULTA DE CONTROL O DE SEGUIMIENTO POR ESPECIALISTA EN RADIOTERAPIA	Radiology consultation	cons_radio
Colorectal	890434	INTERCONSULTA POR ESPECIALISTA EN CIRUGÍA GASTROINTESTINAL	Surgical consultation	cons_cirugia
Colorectal	890437	INTERCONSULTA POR ESPECIALISTA EN CIRUGÍA ONCOLÓGICA	Surgical consultation	cons_cirugia
Colorectal	890443	INTERCONSULTA POR ESPECIALISTA EN DOLOR Y CUIDADOS PALIATIVOS	Palliative care consultation	cons_paliativo
Colorectal	890448	INTERCONSULTA POR ESPECIALISTA EN GENÉTICA MÉDICA	Follow-up consultation	cons_seguim
Colorectal	890446	INTERCONSULTA POR ESPECIALISTA EN GASTROENTEROLOGÍA	Follow-up consultation	cons_seguim
Colorectal	890478	INTERCONSULTA POR ESPECIALISTA EN ONCOLOGÍA	Follow-up consultation	cons_seguim
Colorectal	890487	INTERCONSULTA POR ESPECIALISTA EN RADIOTERAPIA	Radiology consultation	cons_radio
Colorectal	423204	RESECCION DE LESION O TUMOR DE ESOFAGO CERVICAL VIA ABIERTA	Spread procedures	diseminacion
Colorectal	423205	RESECCION DE LESION O TUMOR DE ESOFAGO TORACICO VIA ABIERTA	Spread procedures	diseminacion
Colorectal	423206	RESECCION DE LESION O TUMOR DE ESOFAGO TORACICO VIA TORACOSCOPICA	Spread procedures	diseminacion
Colorectal	423207	RESECCION DE LESION O TUMOR DE ESOFAGO ABDOMINAL VIA ABIERTA	Spread procedures	diseminacion
Colorectal	423208	RESECCION DE LESION O TUMOR DE ESOFAGO ABDOMINAL VIA LAPAROSCOPICA	Spread procedures	diseminacion
Colorectal	423301	POLIPECTOMÍA DE ESÓFAGO VÍA ENDOSCÓPICA	Spread procedures	diseminacion
Colorectal	423308	RESECCIÓN DE LESIÓN MUCOSA DE ESÓFAGO VÍA ENDOSCÓPICA	Spread procedures	diseminacion
Colorectal	423309	RESECCIÓN DE LESIÓN SUBMUCOSA DE ESÓFAGO VÍA ENDOSCÓPICA	Spread procedures	diseminacion
Colorectal	452401	SIGMOIDOSCOPIA FLEXIBLE O RIGIDA	Spread procedures	diseminacion
Colorectal	860102	BIOPSIA INCISIONAL O ESCISIONAL DE PIEL, TEJIDO CELULAR SUBCUTANEO O MUCOSA (CON SUTURA)	Spread procedures	diseminacion

Colorectal	890233	CONSULTA DE PRIMERA VEZ POR ESPECIALISTA EN CIRUGIA DE TORAX	Spread procedures	diseminacio n
Colorectal	890333	CONSULTA DE CONTROL O DE SEGUIMIENTO POR ESPECIALISTA EN CIRUGIA DE TORAX	Spread procedures	diseminacio n
Colorectal	903809	BILIRRUBINAS TOTAL Y DIRECTA	Spread procedures	diseminacio n
Colorectal	903822	CREATINA	Spread procedures	diseminacio n
Colorectal	903823	CREATININA DEPURACION	Spread procedures	diseminacio n
Colorectal	903824	CREATININA EN ORINA DE 24 HORAS	Spread procedures	diseminacio n
Colorectal	903828	DESHIDROGENASA LACTICA	Spread procedures	diseminacio n
Colorectal	903829	DESHIDROGENASA LACTICA ISOENZIMAS	Spread procedures	diseminacio n
Colorectal	903830	FOSFATASA ACIDA	Spread procedures	diseminacio n
Colorectal	903833	FOSFATASA ALCALINA	Spread procedures	diseminacio n
Colorectal	903834	FOSFATASA ALCALINA ESPECIFICA DE HUESO	Spread procedures	diseminacio n
Colorectal	903856	NITROGENO UREICO	Spread procedures	diseminacio n
Colorectal	903857	NITROGENO UREICO EN ORINA DE 24 HORAS	Spread procedures	diseminacio n
Colorectal	903866	TRANSAMINASA GLUTAMICO-PIRUVICA [ALANINO AMINO TRANSFERASA]	Spread procedures	diseminacio n
Colorectal	903867	TRANSAMINASA GLUTAMICO OXALACETICA [ASPARTATO AMINO TRANSFERASA]	Spread procedures	diseminacio n
Colorectal	903876	CREATININA EN ORINA PARCIAL	Spread procedures	diseminacio n
Colorectal	903881	CREATINA EN ORINA DE 24 HORAS	Spread procedures	diseminacio n
Colorectal	903895	CREATININA EN SUERO U OTROS FLUIDOS	Spread procedures	diseminacio n
Colorectal	879301	TOMOGRAFIA COMPUTADA DE TORAX	Spread procedures	diseminacio n
Colorectal	872002	RADIOGRAFIA DE ABDOMEN SIMPLE	Diagnostic imaging	imag_diagno s
Colorectal	872104	RADIOGRAFIA DE COLON POR ENEMA O COLON POR INGESTA	Diagnostic imaging	imag_diagno s
Colorectal	872105	RADIOGRAFIA DE COLON POR ENEMA CON DOBLE CONTRASTE	Diagnostic imaging	imag_diagno s
Colorectal	879410	TOMOGRAFIA COMPUTADA DE ABDOMEN SUPERIOR	Diagnostic imaging	imag_diagno s
Colorectal	879420	TOMOGRAFIA COMPUTADA DE ABDOMEN Y PELVIS (ABDOMEN TOTAL)	Diagnostic imaging	imag_diagno s

Colorectal	879460	TOMOGRAFIA COMPUTADA DE PELVIS	Diagnostic imaging	imag_diagnos
Colorectal	879601	TOMOGRAFIA POR EMISION DE POSITRONES [PET-TC]	Diagnostic imaging	imag_diagnos
Colorectal	881301	ECOGRAFIA DE TEJIDOS BLANDOS DE PARED ABDOMINAL Y DE PELVIS	Diagnostic imaging	imag_diagnos
Colorectal	881302	ECOGRAFIA DE ABDOMEN TOTAL	Diagnostic imaging	imag_diagnos
Colorectal	881305	ECOGRAFIA DE ABDOMEN SUPERIOR	Diagnostic imaging	imag_diagnos
Colorectal	881319	ECOGRAFIA ENDOSCOPICA DE RECTO	Diagnostic imaging	imag_diagnos
Colorectal	881390	ECOGRAFIA DEL ABDOMEN Y PELVIS COMO GUIA DE PROCEDIMIENTO QUIRURGICO O INTERVENCIONISTA	Diagnostic imaging	imag_diagnos
Colorectal	883401	RESONANCIA MAGNETICA DE ABDOMEN	Diagnostic imaging	imag_diagnos
Colorectal	883440	RESONANCIA MAGNETICA DE PELVIS	Diagnostic imaging	imag_diagnos
Colorectal	890602	CUIDADO (MANEJO) INTRAHOSPITALARIO POR MEDICINA ESPECIALIZADA	Inpatient services	internacion
Colorectal	107M01	INTERNACIÓN EN UNIDAD DE CUIDADO INTERMEDIO ADULTO	Inpatient services	internacion
Colorectal	10A001	INTERNACIÓN COMPLEJIDAD ALTA HABITACION UNIPERSONAL (INCLUYE AISLAMIENTO)	Inpatient services	internacion
Colorectal	10A002	INTERNACIÓN COMPLEJIDAD ALTA HABITACION BIPERSONAL	Inpatient services	internacion
Colorectal	10A003	INTERNACIÓN COMPLEJIDAD ALTA TRES CAMAS	Inpatient services	internacion
Colorectal	10A004	INTERNACIÓN COMPLEJIDAD ALTA CUATRO O MÁS CAMAS	Inpatient services	internacion
Colorectal	10A005	INTERNACIÓN EN UNIDAD DE TRASPLANTE	Inpatient services	internacion
Colorectal	10M001	INTERNACIÓN COMPLEJIDAD MEDIANA HABITACION UNIPERSONAL (INCLUYE AISLAMIENTO)	Inpatient services	internacion
Colorectal	10M002	INTERNACIÓN COMPLEJIDAD MEDIANA HABITACION BIPERSONAL	Inpatient services	internacion
Colorectal	10M003	INTERNACIÓN COMPLEJIDAD MEDIANA HABITACION TRES CAMAS	Inpatient services	internacion
Colorectal	10M004	INTERNACIÓN COMPLEJIDAD MEDIANA HABITACION CUATRO O MÁS CAMAS	Inpatient services	internacion
Colorectal	110A01	INTERNACIÓN EN UNIDAD DE CUIDADO INTENSIVO ADULTOS	Inpatient services	internacion
Colorectal	121M01	INTERNACIÓN DE PACIENTE CRÓNICO TERMINAL SIN VENTILADOR COMPLEJIDAD MEDIANA	Inpatient services	internacion
Colorectal	121M02	INTERNACIÓN DE PACIENTE CRÓNICO TERMINAL CON VENTILADOR COMPLEJIDAD MEDIANA	Inpatient services	internacion
Colorectal	S11201	INTERNACIÓN COMPLEJIDAD MEDIANA HABITACION UNIPERSONAL (INCLUYE AISLAMIENTO)	Inpatient services	internacion
Colorectal	S11202	INTERNACIÓN COMPLEJIDAD MEDIANA HABITACION BIPERSONAL	Inpatient services	internacion
Colorectal	S11203	INTERNACIÓN COMPLEJIDAD MEDIANA HABITACION TRES CAMAS	Inpatient services	internacion
Colorectal	S11204	INTERNACIÓN COMPLEJIDAD MEDIANA HABITACION CUATRO O MÁS CAMAS	Inpatient services	internacion
Colorectal	S11301	INTERNACIÓN COMPLEJIDAD ALTA HABITACION UNIPERSONAL (INCLUYE AISLAMIENTO)	Inpatient services	internacion
Colorectal	S11302	INTERNACIÓN COMPLEJIDAD ALTA HABITACION BIPERSONAL	Inpatient services	internacion
Colorectal	S11303	INTERNACIÓN COMPLEJIDAD ALTA TRES CAMAS	Inpatient services	internacion
Colorectal	S11304	INTERNACIÓN COMPLEJIDAD ALTA CUATRO O MÁS CAMAS	Inpatient services	internacion
Colorectal	S12103	INTERNACIÓN EN UNIDAD DE CUIDADO INTENSIVO ADULTOS	Inpatient services	internacion
Colorectal	S12203	INTERNACIÓN EN UNIDAD DE CUIDADO INTERMEDIO ADULTO	Inpatient services	internacion
Colorectal	S12600	INTERNACIÓN EN UNIDAD DE TRASPLANTE	Inpatient services	internacion
Colorectal	898101	ESTUDIO DE COLORACION BASICA EN BIOPSIA	Laboratory tests	laboratorio
Colorectal	898102	ESTUDIO DE COLORACION HISTOQUIMICA EN BIOPSIA	Laboratory tests	laboratorio
Colorectal	898103	ESTUDIO DE COLORACION INMUNOHISTOQUIMICA EN BIOPSIA	Laboratory tests	laboratorio
Colorectal	898201	ESTUDIO DE COLORACION BASICA EN ESPECIMEN DE RECONOCIMIENTO	Laboratory tests	laboratorio
Colorectal	898202	ESTUDIO DE COLORACION HISTOQUIMICA EN ESPECIMEN DE RECONOCIMIENTO	Laboratory tests	laboratorio

Colorectal	898203	ESTUDIO DE COLORACION INMUNOHISTOQUIMICA EN ESPECIMEN DE RECONOCIMIENTO	Laboratory tests	laboratorio
Colorectal	898221	ESTUDIO DE COLORACION BASICA EN ESPECIMEN CON MULTIPLE MUESTREO	Laboratory tests	laboratorio
Colorectal	898222	ESTUDIO DE COLORACION HISTOQUIMICA EN ESPECIMEN CON MULTIPLE MUESTREO	Laboratory tests	laboratorio
Colorectal	898223	ESTUDIO DE COLORACION INMUNOHISTOQUIMICA EN ESPECIMEN CON MULTIPLE MUESTREO	Laboratory tests	laboratorio
Colorectal	898241	ESTUDIO DE COLORACION BASICA EN ESPECIMEN CON RESECCION DE MARGENES	Laboratory tests	laboratorio
Colorectal	898241	ESTUDIO DE COLORACION BASICA EN ESPECIMEN CON RESECCION DE MARGENES	Laboratory tests	laboratorio
Colorectal	898242	ESTUDIO DE COLORACION HISTOQUIMICA EN ESPECIMEN CON RESECCION DE MARGENES	Laboratory tests	laboratorio
Colorectal	898243	ESTUDIO DE COLORACION INMUNOHISTOQUIMICA EN ESPECIMEN CON RESECCION DE MARGENES	Laboratory tests	laboratorio
Colorectal	906603	ANTIGENO CARCINOEMBRIONARIO SEMIAUTOMATIZADO O AUTOMATIZADO	Laboratory tests	laboratorio
Colorectal	906606	ANTIGENO DE CANCER DE TUBO DIGESTIVO [CA 19-9] SEMIAUTOMATIZADO O AUTOMATIZADO	Laboratory tests	laboratorio
Colorectal	907008	SANGRE OCULTA EN MATERIA FECAL [GUAYACO O EQUIVALENTE]	Laboratory tests	laboratorio
Colorectal	907009	SANGRE OCULTA EN MATERIA FECAL (DETERMINACION DE HEMOGLOBINA HUMANA ESPECIFICA)	Laboratory tests	laboratorio
Colorectal	907012	SANGRE OCULTA EN MATERIA FECAL [GUAYACO O EQUIVALENTE] SERIADO TRES MUESTRAS	Laboratory tests	laboratorio
Colorectal	992503	MONOTERAPIA ANTINEOPLÁSICA DE BAJA TOXICIDAD	Monoterapy	monoterapia
Colorectal	992504	POLITERAPIA ANTINEOPLÁSICA DE BAJA TOXICIDAD	Polyterapy	politerapia
Colorectal	992505	POLITERAPIA ANTINEOPLÁSICA DE ALTA TOXICIDAD	Polyterapy	politerapia
Colorectal	992506	ADMINISTRACIÓN (INFUSIÓN O PERFUSIÓN) DE TERAPIA ANTINEOPLÁSICA INTRARTERIAL (REGIONAL)	Monoterapy	monoterapia
Colorectal	992509	MONOTERAPIA ANTINEOPLÁSICA DE BAJA TOXICIDAD	Monoterapy	monoterapia
Colorectal	992511	MONOTERAPIA ANTINEOPLÁSICA DE ALTA TOXICIDAD	Monoterapy	monoterapia
Colorectal	922201	TELETERAPIA CON ORTOVOLTAJE	Teletherapy	teleterapia
Colorectal	922321	TELETERAPIA CON COBALTO (PLANEACIÓN COMPUTARIZADA BIDIMENSIONAL Y SIMULACIÓN CONVENCIONAL)	Teletherapy	teleterapia
Colorectal	922322	TELETERAPIA CON COBALTO (PLANEACIÓN COMPUTARIZADA TRIDIMENSIONAL Y SIMULACIÓN VIRTUAL)	Teletherapy	teleterapia
Colorectal	922441	TELETERAPIA CON ACELERADOR LINEAL (PLANEACIÓN COMPUTARIZADA BIDIMENSIONAL Y SIMULACIÓN CONVENCIONAL) TÉCNICA RADIOTERAPIA CONVENCIONAL	Teletherapy	teleterapia
Colorectal	922442	TELETERAPIA CON ACELERADOR LINEAL (PLANEACIÓN COMPUTARIZADA TRIDIMENSIONAL Y SIMULACIÓN VIRTUAL) TÉCNICA RADIOTERAPIA CONVENCIONAL	Teletherapy	teleterapia
Colorectal	922443	TELETERAPIA CON ACELERADOR LINEAL (PLANEACION COMPUTARIZADA TRIDIMENSIONAL Y SIMULACION VIRTUAL) TECNICA CONFORMACIONAL [3D - CRT]	Teletherapy	teleterapia
Colorectal	922444	TELETERAPIA CON ACELERADOR LINEAL (PLANEACIÓN COMPUTARIZADA TRIDIMENSIONAL Y SIMULACIÓN VIRTUAL) TÉCNICA RADIOTERAPIA DE INTENSIDAD MODULADA [IMRT]	Teletherapy	teleterapia
Colorectal	922445	TELETERAPIA CON ACELERADOR LINEAL (PLANEACIÓN COMPUTARIZADA TRIDIMENSIONAL Y SIMULACIÓN VIRTUAL) TÉCNICA RADIOTERAPIA GUIADA POR IMÁGENES [IGRT]	Teletherapy	teleterapia
Colorectal	922446	TELETERAPIA CON ACELERADOR LINEAL (PLANEACIÓN COMPUTARIZADA TRIDIMENSIONAL Y SIMULACIÓN VIRTUAL) TÉCNICA RADIOTERAPIA - ARCOTERAPIA DE MODULACIÓN VOLUMÉTRICA [VMAT]	Teletherapy	teleterapia
Colorectal	922447	IRRADIACIÓN CORPORAL TOTAL	Irradiation	irradiacion
Colorectal	922448	IRRADIACIÓN CUTÁNEA TOTAL	Irradiation	irradiacion
Colorectal	922449	TELETERAPIA CON ACELERADOR LINEAL (PLANEACIÓN COMPUTARIZADA TRIDIMENSIONAL Y SIMULACIÓN VIRTUAL) TÉCNICA RADIOTERAPIA HELICOIDAL	Teletherapy	teleterapia
Colorectal	922504	TELETERAPIA CON ACELERADOR LINEAL DE ELECTRONES (PLANEACION COMPUTARIZADA BIDIMENSIONAL Y SIMULACION CONVENCIONAL)	Teletherapy	teleterapia
Colorectal	922505	TELETERAPIA CON ACELERADOR LINEAL DE ELECTRONES (PLANEACION COMPUTARIZADA TRIDIMENSIONAL Y SIMULACIÓN VIRTUAL)	Teletherapy	teleterapia
Colorectal	922506	RADIOTERAPIA INTRAOPERATORIA	Teletherapy	teleterapia
Colorectal	922601	BRAQUITERAPIA INTERSTICIAL (PLANEACION COMPUTARIZADA TRIDIMENSIONAL Y SIMULACION VIRTUAL) CON ALTA TASA DE DOSIS	Brachytherapy	braquiterapi a
Colorectal	922602	BRAQUITERAPIA INTERSTICIAL (PLANEACION COMPUTARIZADA TRIDIMENSIONAL Y SIMULACIÓN VIRTUAL) CON BAJA TASA DE DOSIS	Brachytherapy	braquiterapi a

Colorectal	922603	BRAQUITERAPIA INTRALUMINAL (PLANEACION COMPUTARIZADA BIDIMENSIONAL Y SIMULACION CONVENCIONAL) CON ALTA TASA DE DOSIS	Brachytherapy	braquiterapi a
Colorectal	922604	BRAQUITERAPIA INTRALUMINAL CON BAJA TASA DE DOSIS	Brachytherapy	braquiterapi a
Colorectal	922605	BRAQUITERAPIA INTRACAVITARIA (PLANEACION COMPUTARIZADA BIDIMENSIONAL Y SIMULACION CONVENCIONAL) CON ALTA TASA DE DOSIS	Brachytherapy	braquiterapi a
Colorectal	922606	BRAQUITERAPIA INTRACAVITARIA (PLANEACION COMPUTARIZADA BIDIMENSIONAL Y SIMULACION CONVENCIONAL) CON BAJA TASA DE DOSIS	Brachytherapy	braquiterapi a
Colorectal	922607	BRAQUITERAPIA INTRACAVITARIA (PLANEACION COMPUTARIZADA TRIDIMENSIONAL Y SIMULACION VIRTUAL) CON ALTA TASA DE DOSIS	Brachytherapy	braquiterapi a
Colorectal	922608	BRAQUITERAPIA INTRACAVITARIA (PLANEACIÓN COMPUTARIZADA TRIDIMENSIONAL Y SIMULACIÓN VIRTUAL) CON BAJA TASA DE DOSIS	Brachytherapy	braquiterapi a
Colorectal	922609	BRAQUITERAPIA INTRALUMINAL (PLANEACIÓN COMPUTARIZADA TRIDIMENSIONAL Y SIMULACIÓN VIRTUAL) CON ALTA TASA DE DOSIS	Brachytherapy	braquiterapi a
Colorectal	922611	BRAQUITERAPIA DE CONTACTO (PLANEACION COMPUTARIZADA BIDIMENSIONAL Y SIMULACION CONVENCIONAL) CON ALTA TASA DE DOSIS	Brachytherapy	braquiterapi a
Colorectal	922612	BRAQUITERAPIA DE CONTACTO (PLANEACIÓN COMPUTARIZADA TRIDIMENSIONAL Y SIMULACIÓN VIRTUAL) CON ALTA TASA DE DOSIS	Brachytherapy	braquiterapi a
Colorectal	922613	BRAQUITERAPIA EPIESCLERAL DE CONTACTO (PLANEACIÓN COMPUTARIZADA TRIDIMENSIONAL Y SIMULACIÓN VIRTUAL) CON BAJA TASA DE DOSIS	Brachytherapy	braquiterapi a
Colorectal	922614	BRAQUITERAPIA METABÓLICA	Brachytherapy	braquiterapi a
Colorectal	922615	BRAQUITERAPIA INTERSTICIAL (PLANEACIÓN COMPUTARIZADA BIDIMENSIONAL Y SIMULACIÓN CONVENCIONAL) CON ALTA TASA DE DOSIS	Brachytherapy	braquiterapi a
Colorectal	922616	BRAQUITERAPIA INTERSTICIAL (PLANEACIÓN COMPUTARIZADA TRIDIMENSIONAL Y SIMULACIÓN VIRTUAL) CON ALTA TASA DE DOSIS	Brachytherapy	braquiterapi a
Colorectal	922800	TERAPIA CON RADIOISOTOPOS SOD	Radiotherapy	terapia
Colorectal	922801	TERAPIA CON RADIOISÓTOPOS	Radiotherapy	terapia
Colorectal	922805	TERAPIA CON ITRIUM 90	Radiotherapy	terapia
Colorectal	922810	TERAPIA CON METAIODOBENCILGUANIDINA SUPRARRENAL	Radiotherapy	terapia
Colorectal	922830	TERAPIA DE METÁSTASIS CON ESTRONCIO	Radiotherapy	terapia
Lung	332001	BRONCOSCOPIA FIBRO-OPTICA CON PUNCION (ASPIRACION) TRANSTRAQUEAL O TRANSBRONQUIAL CON AGUJA	Bronchoscopy	broncoscopi a
Lung	332101	BRONCOSCOPIA A TRAVES DE ESTOMA ARTIFICIAL	Bronchoscopy	broncoscopi a
Lung	332201	BRONCOSCOPIA CON LAVADO BRONQUIAL	Bronchoscopy	broncoscopi a
Lung	332202	BRONCOSCOPIA	Bronchoscopy	broncoscopi a
Lung	332203	BRONCOSCOPIA CON LAVADO BRONCOALVEOLAR	Bronchoscopy	broncoscopi a
Lung	332204	BRONCOSCOPIA CON CEPILLADO	Bronchoscopy	broncoscopi a
Lung	332205	BRONCOSCOPIA CON APLICACION O RETIRO DE FUENTE RADIACTIVA	Bronchoscopy	broncoscopi a
Lung	332206	BRONCOSCOPIA CON PUNCION (ASPIRACION) TRANSTRAQUEAL	Bronchoscopy	broncoscopi a
Lung	332207	BRONCOSCOPIA CON PUNCION (ASPIRACION) TRANSBRONQUIAL	Bronchoscopy	broncoscopi a

Lung	332208	BRONCOSCOPIA CON AUTOFLUORESCENCIA	Bronchoscopy	broncoscopi a
Lung	332209	BRONCOSCOPIA CON TOMOGRAFIA DE COHERENCIA OPTICA	Bronchoscopy	broncoscopi a
Lung	332210	BRONCOSCOPIA CON TERMOPLASTIA BRONQUIAL	Bronchoscopy	broncoscopi a
Lung	332301	BRONCOSCOPIA RIGIDA CON LAVADO BRONQUIAL	Bronchoscopy	broncoscopi a
Lung	332302	EXPLORACION ENDOSCOPICA DE BRONQUIOS [BRONCOSCOPIA] RIGIDA	Bronchoscopy	broncoscopi a
Lung	345100	PLEURECTOMIA PARIETAL SOD	Pleural surgery	cirug_pleura
Lung	345101	PLEURECTOMÍA PARIETAL VÍA ABIERTA	Pleural surgery	cirug_pleura
Lung	345102	PLEURECTOMIA PARIETAL POR TORACOSCOPIA	Pleural surgery	cirug_pleura
Lung	345300	DECORTICACION PULMONAR SOD	Pleural surgery	cirug_pleura
Lung	345301	DECORTICACIÓN PULMONAR VÍA ABIERTA	Pleural surgery	cirug_pleura
Lung	345302	DECORTICACIÓN PULMONAR POR TORACOSCOPIA	Pleural surgery	cirug_pleura
Lung	345501	RESECCIÓN DE TUMOR DE PLEURA VÍA ABIERTA	Pleural surgery	cirug_pleura
Lung	345502	RESECCIÓN DE TUMOR DE PLEURA POR TORACOSCOPIA	Pleural surgery	cirug_pleura
Lung	345503	LISIS DE ADHERENCIAS PLEURALES VÍA ABIERTA	Pleural surgery	cirug_pleura
Lung	345504	LISIS DE ADHERENCIAS PLEURALES POR TORACOSCOPIA	Pleural surgery	cirug_pleura
Lung	890212	CONSULTA DE PRIMERA VEZ POR TERAPIA RESPIRATORIA	Follow-up consultation	cons_seguim
Lung	890233	CONSULTA DE PRIMERA VEZ POR ESPECIALISTA EN CIRUGÍA DE TÓRAX	Surgical consultation	cons_cirugia
Lung	890237	CONSULTA DE PRIMERA VEZ POR ESPECIALISTA EN CIRUGÍA ONCOLÓGICA	Surgical consultation	cons_cirugia
Lung	890239	CONSULTA DE PRIMERA VEZ POR ESPECIALISTA EN CIRUGÍA PLÁSTICA, ESTÉTICA Y RECONSTRUCTIVA	Surgical consultation	cons_cirugia
Lung	890243	CONSULTA DE PRIMERA VEZ POR ESPECIALISTA EN DOLOR Y CUIDADOS PALIATIVOS	Palliative care consultation	cons_paliati vo
Lung	890248	CONSULTA DE PRIMERA VEZ POR ESPECIALISTA EN GENETICA MEDICA	Follow-up consultation	cons_seguim
Lung	890271	CONSULTA DE PRIMERA VEZ POR ESPECIALISTA EN NEUMOLOGIA	Follow-up consultation	cons_seguim
Lung	890278	CONSULTA DE PRIMERA VEZ POR ESPECIALISTA EN ONCOLOGÍA	Follow-up consultation	cons_seguim
Lung	890287	CONSULTA DE PRIMERA VEZ POR ESPECIALISTA EN RADIOTERAPIA	Radiology consultation	cons_radio
Lung	890312	CONSULTA DE CONTROL O DE SEGUIMIENTO POR TERAPIA RESPIRATORIA	Follow-up consultation	cons_seguim
Lung	890333	CONSULTA DE CONTROL O DE SEGUIMIENTO POR ESPECIALISTA EN CIRUGÍA DE TÓRAX	Surgical consultation	cons_cirugia
Lung	890337	CONSULTA DE CONTROL O DE SEGUIMIENTO POR ESPECIALISTA EN CIRUGÍA ONCOLÓGICA	Surgical consultation	cons_cirugia
Lung	890339	CONSULTA DE CONTROL O DE SEGUIMIENTO POR ESPECIALISTA EN CIRUGÍA PLÁSTICA, ESTÉTICA Y RECONSTRUCTIVA	Surgical consultation	cons_cirugia
Lung	890343	CONSULTA DE CONTROL O DE SEGUIMIENTO POR ESPECIALISTA EN DOLOR Y CUIDADOS PALIATIVOS	Palliative care consultation	cons_paliati vo

Lung	890348	CONSULTA DE CONTROL O DE SEGUIMIENTO POR ESPECIALISTA EN GENÉTICA MÉDICA	Follow-up consultation	cons_seguim
Lung	890371	CONSULTA DE CONTROL O DE SEGUIMIENTO POR ESPECIALISTA EN NEUMOLOGIA	Follow-up consultation	cons_seguim
Lung	890378	CONSULTA DE CONTROL O DE SEGUIMIENTO POR ESPECIALISTA EN ONCOLOGIA	Follow-up consultation	cons_seguim
Lung	890387	CONSULTA DE CONTROL O DE SEGUIMIENTO POR ESPECIALISTA EN RADIOTERAPIA	Radiology consultation	cons_radio
Lung	890412	INTERCONSULTA POR TERAPIA RESPIRATORIA	Follow-up consultation	cons_seguim
Lung	890433	INTERCONSULTA POR ESPECIALISTA EN CIRUGÍA DE TÓRAX	Surgical consultation	cons_cirugia
Lung	890437	INTERCONSULTA POR ESPECIALISTA EN CIRUGÍA ONCOLÓGICA	Surgical consultation	cons_cirugia
Lung	890439	INTERCONSULTA POR ESPECIALISTA EN CIRUGÍA PLÁSTICA, ESTÉTICA Y RECONSTRUCTIVA	Surgical consultation	cons_cirugia
Lung	890443	INTERCONSULTA POR ESPECIALISTA EN DOLOR Y CUIDADOS PALIATIVOS	Palliative care consultation	cons_paliati vo
Lung	890448	INTERCONSULTA POR ESPECIALISTA EN GENÉTICA MÉDICA	Follow-up consultation	cons_seguim
Lung	890471	INTERCONSULTA POR ESPECIALISTA EN NEUMOLOGÍA	Follow-up consultation	cons_seguim
Lung	890478	INTERCONSULTA POR ESPECIALISTA EN ONCOLOGÍA	Follow-up consultation	cons_seguim
Lung	890487	INTERCONSULTA POR ESPECIALISTA EN RADIOTERAPIA	Radiology consultation	cons_radio
Lung	332401	BIOPSIA DE BRONQUIO VIA ENDOSCOPICA	Screening	diagnostico
Lung	332501	BIOPSIA DE BRONQUIO VIA ABIERTA	Screening	diagnostico
Lung	332601	BIOPSIA CERRADA DE PULMON VIA PERCUTANEA	Screening	diagnostico
Lung	332703	BIOPSIA DE PULMON VIA ENDOSCOPICA	Screening	diagnostico
Lung	332704	BIOPSIA DE PULMON POR TORACOSCOPIA	Screening	diagnostico
Lung	332801	BIOPSIA DE PULMON VIA ABIERTA	Screening	diagnostico
Lung	332902	EXTRACCION DE CUERPO EXTRAÑO DE BRONQUIO O PULMON VIA ENDOSCOPICA	Screening	diagnostico
Lung	340201	TORACOSTOMIA EXPLORATORIA	Screening	diagnostico
Lung	340301	TORACOSTOMIA VIA ABIERTA CON RESECCION COSTAL	Screening	diagnostico
Lung	340401	TORACOSTOMIA CERRADA PARA DRENAJE	Screening	diagnostico
Lung	341001	MEDIASTINOSCOPIA DIAGNOSTICA	Screening	diagnostico
Lung	342101	TORACOSCOPIA DIAGNOSTICA	Screening	diagnostico
Lung	342101	TORACOSCOPIA DIAGNOSTICA	Screening	diagnostico
Lung	345401	BIOPSIA DE PLEURA PERCUTANEA	Screening	diagnostico
Lung	345403	BIOPSIAS DE PLEURA POR TORACOSCOPIA	Screening	diagnostico
Lung	340501	BIOPSIA DE LESION DE PARED TORACICA VIA PERCUTANEA	Spread procedures	diseminacio n
Lung	341106	EXPLORACION Y DRENAJE DE MEDIASTINO POR TORACOSCOPIA	Spread procedures	diseminacio n
Lung	341201	BIOPSIA DE ORGANO O TEJIDO DE MEDIASTINO VIA PERCUTANEA	Spread procedures	diseminacio n
Lung	341203	BIOPSIA DE ORGANO O TEJIDO DE MEDIASTINO POR MEDIASTINOSCOPIA	Spread procedures	diseminacio n

Lung	341204	BIOPSIA DE ORGANO O TEJIDO DE MEDIASTINO POR TORACOSCOPIA	Spread procedures	diseminacio n
Lung	341401	RESECCION DE TUMOR MALIGNO DEL MEDIASTINO POR TORACOTOMIA	Spread procedures	diseminacio n
Lung	341402	RESECCION DE TUMOR MALIGNO DEL MEDIASTINO POR ESTERNOTOMIA	Spread procedures	diseminacio n
Lung	341403	RESECCION DE TUMOR MALIGNO DEL MEDIASTINO POR TORACOSCOPIA	Spread procedures	diseminacio n
Lung	405201	VACIAMIENTO RADICAL LINFATICO (LINFADENECTOMIA) DE MEDIASTINO VIA ABIERTA	Spread procedures	diseminacio n
Lung	405202	VACIAMIENTO RADICAL LINFATICO (LINFADENECTOMIA) DE MEDIASTINO VIA TORACOSCOPICA	Spread procedures	diseminacio n
Lung	879410	TOMOGRAFIA COMPUTADA DE ABDOMEN SUPERIOR	Spread procedures	diseminacio n
Lung	879420	TOMOGRAFIA COMPUTADA DE ABDOMEN Y PELVIS (ABDOMEN TOTAL)	Spread procedures	diseminacio n
Lung	879420	TOMOGRAFIA COMPUTADA DE ABDOMEN Y PELVIS (ABDOMEN TOTAL)	Spread procedures	diseminacio n
Lung	881301	ECOGRAFIA DE TEJIDOS BLANDOS DE PARED ABDOMINAL Y DE PELVIS	Spread procedures	diseminacio n
Lung	881302	ECOGRAFIA DE ABDOMEN TOTAL	Spread procedures	diseminacio n
Lung	881305	ECOGRAFIA DE ABDOMEN SUPERIOR	Spread procedures	diseminacio n
Lung	881306	ECOGRAFIA DE HIGADO, PANCREAS, VIA BILIAR Y VESICULA	Spread procedures	diseminacio n
Lung	881332	ECOGRAFIA DE VIAS URINARIAS (RIÑONES, VEJIGA Y PROSTATA TRANSABDOMINAL)	Spread procedures	diseminacio n
Lung	881390	ECOGRAFIA DEL ABDOMEN Y PELVIS COMO GUIA DE PROCEDIMIENTO QUIRURGICO O INTERVENCIONISTA	Spread procedures	diseminacio n
Lung	881401	ECOGRAFIA PELVICA GINECOLOGICA TRANSVAGINAL	Spread procedures	diseminacio n
Lung	883101	RESONANCIA MAGNETICA DE CEREBRO	Spread procedures	diseminacio n
Lung	883401	RESONANCIA MAGNETICA DE ABDOMEN	Spread procedures	diseminacio n
Lung	883401	RESONANCIA MAGNETICA DE ABDOMEN	Spread procedures	diseminacio n
Lung	883440	RESONANCIA MAGNETICA DE PELVIS	Spread procedures	diseminacio n
Lung	883440	RESONANCIA MAGNETICA DE PELVIS	Spread procedures	diseminacio n
Lung	903856	NITROGENO UREICO	Spread procedures	diseminacio n
Lung	903857	NITROGENO UREICO EN ORINA DE 24 HORAS	Spread procedures	diseminacio n
Lung	903866	TRANSAMINASA GLUTAMICO-PIRUVICA [ALANINO AMINO TRANSFERASA]	Spread procedures	diseminacio n

Lung	903867	TRANSAMINASA GLUTAMICO OXALACETICA [ASPARTATO AMINO TRANSFERASA]	Spread procedures	diseminacio n
Lung	903876	CREATININA EN ORINA PARCIAL	Spread procedures	diseminacio n
Lung	903881	CREATINA EN ORINA DE 24 HORAS	Spread procedures	diseminacio n
Lung	903895	CREATININA EN SUERO U OTROS FLUIDOS	Spread procedures	diseminacio n
Lung	871121	RADIOGRAFÍA DE TÓRAX (P.A. O A.P. Y LATERAL, DECÚBITO LATERAL, OBLICUAS O LATERAL)	Diagnostic imaging	imag_diagno s
Lung	879301	TOMOGRAFIA COMPUTADA DE TORAX	Diagnostic imaging	imag_diagno s
Lung	879301	TOMOGRAFÍA COMPUTADA DE TÓRAX	Diagnostic imaging	imag_diagno s
Lung	879391	TOMOGRAFIA COMPUTADA DE TORAX EXTENDIDO AL ABDOMEN SUPERIOR CON SUPRARRENALES	Diagnostic imaging	imag_diagno s
Lung	879601	TOMOGRAFIA POR EMISION DE POSITRONES [PET-TC]	Diagnostic imaging	imag_diagno s
Lung	881202	ECOCARDIOGRAMA TRANSTORACICO	Diagnostic imaging	imag_diagno s
Lung	881202	ECOCARDIOGRAMA TRANSTORACICO	Diagnostic imaging	imag_diagno s
Lung	881203	ECOCARDIOGRAMA TRANSTORACICO CON CONTRASTE	Diagnostic imaging	imag_diagno s
Lung	881204	ECOCARDIOGRAMA TRANSTORACICO TRIDIMENSIONAL	Diagnostic imaging	imag_diagno s
Lung	881205	ECOCARDIOGRAMA TRANSESOFAGICO	Diagnostic imaging	imag_diagno s
Lung	881207	ECOCARDIOGRAMA TRANSESOFAGICO TRIDIMENSIONAL	Diagnostic imaging	imag_diagno s
Lung	881211	ECOGRAFIA DE TORAX (PERICARDIO O PLEURA)	Diagnostic imaging	imag_diagno s
Lung	881212	ECOGRAFIA DE OTROS SITIOS TORACICOS	Diagnostic imaging	imag_diagno s
Lung	881213	ECOGRAFIA ENDOSCOPICA DE MEDIASTINO, TRAQUEA Y BRONQUIOS	Diagnostic imaging	imag_diagno s
Lung	881213	ECOGRAFIA ENDOSCOPICA DE MEDIASTINO, TRAQUEA Y BRONQUIOS	Diagnostic imaging	imag_diagno s
Lung	883210	RESONANCIA MAGNETICA DE COLUMNA CERVICAL SIMPLE	Diagnostic imaging	imag_diagno s
Lung	883211	RESONANCIA MAGNETICA DE COLUMNA CERVICAL CON CONTRASTE	Diagnostic imaging	imag_diagno s
Lung	883220	RESONANCIA MAGNETICA DE COLUMNA TORACICA SIMPLE	Diagnostic imaging	imag_diagno s
Lung	883221	RESONANCIA MAGNETICA DE COLUMNA TORACICA CON CONTRASTE	Diagnostic imaging	imag_diagno s
Lung	883230	RESONANCIA MAGNETICA DE COLUMNA LUMBOSACRA SIMPLE	Diagnostic imaging	imag_diagno s

Lung	883231	RESONANCIA MAGNETICA DE COLUMNA LUMBAR CON CONTRASTE	Diagnostic imaging	imag_diagnos
Lung	890602	CUIDADO (MANEJO) INTRAHOSPITALARIO POR MEDICINA ESPECIALIZADA	Inpatient services	internacion
Lung	107M01	INTERNACIÓN EN UNIDAD DE CUIDADO INTERMEDIO ADULTO	Inpatient services	internacion
Lung	10A001	INTERNACIÓN COMPLEJIDAD ALTA HABITACION UNIPERSONAL (INCLUYE AISLAMIENTO)	Inpatient services	internacion
Lung	10A002	INTERNACIÓN COMPLEJIDAD ALTA HABITACION BIPERSONAL	Inpatient services	internacion
Lung	10A003	INTERNACIÓN COMPLEJIDAD ALTA TRES CAMAS	Inpatient services	internacion
Lung	10A004	INTERNACIÓN COMPLEJIDAD ALTA CUATRO O MÁS CAMAS	Inpatient services	internacion
Lung	10A005	INTERNACIÓN EN UNIDAD DE TRASPLANTE	Inpatient services	internacion
Lung	10M001	INTERNACIÓN COMPLEJIDAD MEDIANA HABITACION UNIPERSONAL (INCLUYE AISLAMIENTO)	Inpatient services	internacion
Lung	10M002	INTERNACIÓN COMPLEJIDAD MEDIANA HABITACION BIPERSONAL	Inpatient services	internacion
Lung	10M003	INTERNACIÓN COMPLEJIDAD MEDIANA HABITACION TRES CAMAS	Inpatient services	internacion
Lung	10M004	INTERNACIÓN COMPLEJIDAD MEDIANA HABITACION CUATRO O MÁS CAMAS	Inpatient services	internacion
Lung	110A01	INTERNACIÓN EN UNIDAD DE CUIDADO INTENSIVO ADULTOS	Inpatient services	internacion
Lung	121M01	INTERNACIÓN DE PACIENTE CRÓNICO TERMINAL SIN VENTILADOR COMPLEJIDAD MEDIANA	Inpatient services	internacion
Lung	121M02	INTERNACIÓN DE PACIENTE CRÓNICO TERMINAL CON VENTILADOR COMPLEJIDAD MEDIANA	Inpatient services	internacion
Lung	S11201	INTERNACIÓN COMPLEJIDAD MEDIANA HABITACION UNIPERSONAL (INCLUYE AISLAMIENTO)	Inpatient services	internacion
Lung	S11202	INTERNACIÓN COMPLEJIDAD MEDIANA HABITACION BIPERSONAL	Inpatient services	internacion
Lung	S11203	INTERNACIÓN COMPLEJIDAD MEDIANA HABITACION TRES CAMAS	Inpatient services	internacion
Lung	S11204	INTERNACIÓN COMPLEJIDAD MEDIANA HABITACION CUATRO O MÁS CAMAS	Inpatient services	internacion
Lung	S11301	INTERNACIÓN COMPLEJIDAD ALTA HABITACION UNIPERSONAL (INCLUYE AISLAMIENTO)	Inpatient services	internacion
Lung	S11302	INTERNACIÓN COMPLEJIDAD ALTA HABITACION BIPERSONAL	Inpatient services	internacion
Lung	S11303	INTERNACIÓN COMPLEJIDAD ALTA TRES CAMAS	Inpatient services	internacion
Lung	S11304	INTERNACIÓN COMPLEJIDAD ALTA CUATRO O MÁS CAMAS	Inpatient services	internacion
Lung	S12103	INTERNACIÓN EN UNIDAD DE CUIDADO INTENSIVO ADULTOS	Inpatient services	internacion
Lung	S12203	INTERNACIÓN EN UNIDAD DE CUIDADO INTERMEDIO ADULTO	Inpatient services	internacion
Lung	S12600	INTERNACIÓN EN UNIDAD DE TRASPLANTE	Inpatient services	internacion
Lung	898101	ESTUDIO DE COLORACION BASICA EN BIOPSIA	Laboratory tests	laboratorio
Lung	898102	ESTUDIO DE COLORACION HISTOQUIMICA EN BIOPSIA	Laboratory tests	laboratorio
Lung	898103	ESTUDIO DE COLORACION INMUNOHISTOQUIMICA EN BIOPSIA	Laboratory tests	laboratorio
Lung	898104	ESTUDIO DE COLORACION DE INMUNOFLUORESCENCIA EN BIOPSIA	Laboratory tests	laboratorio
Lung	898105	ESTUDIO DE BIOLOGIA MOLECULAR EN BIOPSIA	Laboratory tests	laboratorio
Lung	898201	ESTUDIO DE COLORACION BASICA EN ESPECIMEN DE RECONOCIMIENTO	Laboratory tests	laboratorio
Lung	898202	ESTUDIO DE COLORACION HISTOQUIMICA EN ESPECIMEN DE RECONOCIMIENTO	Laboratory tests	laboratorio
Lung	898203	ESTUDIO DE COLORACION INMUNOHISTOQUIMICA EN ESPECIMEN DE RECONOCIMIENTO	Laboratory tests	laboratorio
Lung	898205	ESTUDIO DE BIOLOGIA MOLECULAR EN ESPECIMEN DE RECONOCIMIENTO	Laboratory tests	laboratorio
Lung	898241	ESTUDIO DE COLORACION BASICA EN ESPECIMEN CON RESECCION DE MARGENES	Laboratory tests	laboratorio
Lung	898242	ESTUDIO DE COLORACION HISTOQUIMICA EN ESPECIMEN CON RESECCION DE MARGENES	Laboratory tests	laboratorio
Lung	898243	ESTUDIO DE COLORACION INMUNOHISTOQUIMICA EN ESPECIMEN CON RESECCION DE MARGENES	Laboratory tests	laboratorio
Lung	898262	ESTUDIO DE COLORACION HISTOQUIMICA EN ESPECIMEN CON MAPEO	Laboratory tests	laboratorio
Lung	898263	ESTUDIO DE COLORACION INMUNOHISTOQUIMICA EN ESPECIMEN CON MAPEO	Laboratory tests	laboratorio
Lung	903852	LIQUIDO PLEURAL (EXAMEN FISICO Y CITOQUIMICO CON RECUENTO Y MORFOLOGIA DE LEUCOCITOS GLUCOSA Y DESHIDROGENASA LACTICA)	Laboratory tests	laboratorio
Lung	906603	ANTIGENO CARCINOEMBRIONARIO SEMIAUTOMATIZADO O AUTOMATIZADO	Laboratory tests	laboratorio
Lung	908420	ESTUDIOS MOLECULARES DE GENES (ESPECIFICOS)	Laboratory tests	laboratorio
Lung	908422	ESTUDIO MOLECULAR DE EXONES (ESPECIFICOS)	Laboratory tests	laboratorio
Lung	920901	GAMAGRAFÍA ÓSEA (CORPORAL TOTAL O SEGMENTARIA)	Laboratory tests	laboratorio
Lung	324101	LOBECTOMIA SEGMENTARIA VIA ABIERTA	Lobectomy	lobectomia

Lung	324102	LOBECTOMIA SEGMENTARIA POR TORACOSCOPIA	Lobectomy	lobectomia
Lung	324103	RESECCION EN CUÑA VIA ABIERTA	Lobectomy	lobectomia
Lung	324104	RESECCION EN CUÑA POR TORACOSCOPIA	Lobectomy	lobectomia
Lung	324105	RESECCION DE METASTASIS PULMONARES VIA ABIERTA	Lobectomy	lobectomia
Lung	324106	RESECCION DE METASTASIS PULMONARES POR TORACOSCOPIA	Lobectomy	lobectomia
Lung	324200	LOBECTOMIA TOTAL PULMONAR SOD	Lobectomy	lobectomia
Lung	324201	LOBECTOMÍA TOTAL PULMONAR VÍA ABIERTA	Lobectomy	lobectomia
Lung	324202	LOBECTOMIA TOTAL PULMONAR POR TORACOSCOPIA	Lobectomy	lobectomia
Lung	324203	BILOBECTOMÍA PULMONAR VÍA ABIERTA	Lobectomy	lobectomia
Lung	324204	BILOBECTOMÍA PULMONAR POR TORACOSCOPIA	Lobectomy	lobectomia
Lung	324205	LOBECTOMÍA TOTAL PULMONAR (DONANTE VIVO) VÍA ABIERTA	Lobectomy	lobectomia
Lung	324206	LOBECTOMÍA TOTAL PULMONAR (DONANTE VIVO) POR TORACOSCOPIA	Lobectomy	lobectomia
Lung	893701	VOLÚMENES PULMONARES POR PLETISMOGRAFIA, PRE Y POST BRONCODILATADORES	Respiratory tests	med_respiratoria
Lung	893702	VOLÚMENES PULMONARES POR GASES (HELIO OTROS.), PRE Y POST BRONCODILATADORES	Respiratory tests	med_respiratoria
Lung	893703	ESPIROMETRÍA	Respiratory tests	med_respiratoria
Lung	893801	CONSUMO DE OXÍGENO Y PRODUCCIÓN DE CO2 EN REPOSO	Respiratory tests	med_respiratoria
Lung	893802	CURVA DE HIPEROXIA	Respiratory tests	med_respiratoria
Lung	893803	MEDICIÓN DE LA FRACCIÓN EXHALADA DE ÓXIDO NÍTRICO POST ESTÍMULO	Respiratory tests	med_respiratoria
Lung	893804	COOXIMETRÍA	Respiratory tests	med_respiratoria
Lung	893805	ESPIROMETRÍA O CURVA DE FLUJO VOLUMEN PRE Y POST BRONCODILATADORES	Respiratory tests	med_respiratoria
Lung	893806	CAPACIDAD DE DIFUSIÓN CON MONÓXIDO DE CARBONO	Respiratory tests	med_respiratoria
Lung	893807	DISTENSIBILIDAD PULMONAR CON BALÓN ESOFÁGICO	Respiratory tests	med_respiratoria
Lung	893808	ESPIROMETRÍA O CURVA DE FLUJO VOLUMEN SIMPLE	Respiratory tests	med_respiratoria
Lung	893809	RESISTENCIA DE VÍAS AÉREAS POR PLETISMOGRAFÍA SIMPLE	Respiratory tests	med_respiratoria
Lung	893810	MEDICIÓN DE LA FUERZA MUSCULAR RESPIRATORIA (PIM-PEM AJUSTADO CON VOLÚMENES PULMONARES)	Respiratory tests	med_respiratoria
Lung	893811	MEDICIÓN DE PRESIÓN INSPIRATORIA Y ESPIRATORIA MÁXIMA CON EQUIPO PORTATIL	Respiratory tests	med_respiratoria
Lung	893812	REGISTRO DE OXIMETRÍA CUTÁNEA	Respiratory tests	med_respiratoria
Lung	893813	RESISTENCIA DE LAS VÍAS AÉREAS POR PLETISMOGRAFÍA PRE Y POST BRONCODILATADORES	Respiratory tests	med_respiratoria
Lung	893814	PRESIÓN TRANSDIAFRAGMÁTICA CON BALONES	Respiratory tests	med_respiratoria
Lung	893815	PRUEBA DE BRONCOPROVOCACIÓN ESPECÍFICA (ALERGENO) O INESPECÍFICA	Respiratory tests	med_respiratoria

Lung	893816	RESPUESTA VENTILATORIA A LA HIPOXIA	Respiratory tests	med_respirat oria
Lung	893817	RESPUESTA VENTILATORIA A LA HIPERCAPNIA	Respiratory tests	med_respirat oria
Lung	893818	MEDICIÓN NO INVASIVA DE CO2 O CAPNOGRAFIA	Respiratory tests	med_respirat oria
Lung	893819	MEDICIÓN DE LA FRACCIÓN EXHALADA DE ÓXIDO NÍTRICO	Respiratory tests	med_respirat oria
Lung	893819	MEDICIÓN DE LA FRACCIÓN EXHALADA DE ÓXIDO NÍTRICO	Respiratory tests	med_respirat oria
Lung	893820	PRUEBA DE BRONCOMOTRICIDAD CON EJERCICIO	Respiratory tests	med_respirat oria
Lung	893820	PRUEBA DE BRONCOMOTRICIDAD CON EJERCICIO	Respiratory tests	med_respirat oria
Lung	893821	PRUEBA DE BRONCOMOTRICIDAD CON EJERCICIO Y MONITOREO	Respiratory tests	med_respirat oria
Lung	893821	PRUEBA DE BRONCOMOTRICIDAD CON EJERCICIO Y MONITOREO	Respiratory tests	med_respirat oria
Lung	893822	VENTILACIÓN VOLUNTARIA MÁXIMA	Respiratory tests	med_respirat oria
Lung	893823	MEDICIÓN DE CALORIMETRÍA INDIRECTA	Respiratory tests	med_respirat oria
Lung	893824	PRUEBA DE HIDRÓGENO ESPIRADO	Respiratory tests	med_respirat oria
Lung	893825	OSCILOMETRÍA DE IMPULSO	Respiratory tests	med_respirat oria
Lung	894101	PRUEBA DE EJERCICIO CARDIO-PULMONAR INTEGRADA (ERGOESPIROMETRIA)	Respiratory tests	med_respirat oria
Lung	894102	PRUEBA DE ESFUERZO CARDIOVASCULAR	Respiratory tests	med_respirat oria
Lung	894104	PRUEBA DE ESFUERZO EN FASES DE MASTERS	Respiratory tests	med_respirat oria
Lung	894401	OTRA PRUEBA DE ESFUERZO CARDIOVASCULAR	Respiratory tests	med_respirat oria
Lung	894402	PRUEBA DE CAMINATA DE 6 MINUTOS	Respiratory tests	med_respirat oria
Lung	894403	PRUEBA DE 12 MINUTOS [COOPER]	Respiratory tests	med_respirat oria
Lung	895001	MONITOREO ELECTROCARDIOGRÁFICO CONTINUO (HOLTER)	Respiratory tests	med_respirat oria
Lung	895002	MONITOREO ELECTROCARDIOGRÁFICO DE EVENTOS	Respiratory tests	med_respirat oria
Lung	895003	PRUEBA DE MESA BASCULANTE	Respiratory tests	med_respirat oria
Lung	895004	MONITOREO AMBULATORIO DE PRESIÓN ARTERIAL SISTÉMICA	Respiratory tests	med_respirat oria
Lung	895005	MONITOREO AMBULATORIO DE PRESIÓN ARTERIAL CENTRAL	Respiratory tests	med_respirat oria

Lung	895006	MONITOREO CARDÍACO CON TRANSMISIÓN REMOTA	Respiratory tests	med_respiratoria
Lung	895100	ELECTROCARDIOGRAMA DE RITMO O DE SUPERFICIE SOD	Respiratory tests	med_respiratoria
Lung	895201	ELECTROCARDIOGRAMA DE ALTA RESOLUCIÓN (ESTUDIO DE POTENCIALES TARDÍOS)	Respiratory tests	med_respiratoria
Lung	895300	VECTOCARDIOGRAMA (CON ECG) SOD	Respiratory tests	med_respiratoria
Lung	895400	MONITORIZACIÓN ELECTROCARDIOGRÁFICA (TELEMETRÍA)	Respiratory tests	med_respiratoria
Lung	895401	MONITORIZACIÓN ELECTROCARDIOGRÁFICA (TELEMETRÍA)	Respiratory tests	med_respiratoria
Lung	895500	FONOCARDIOGRAMA Y PULSOS SOD	Respiratory tests	med_respiratoria
Lung	895700	APICOGRAMA (CON ELECTRODO DE ECG) SOD	Respiratory tests	med_respiratoria
Lung	895801	ULTRASONIDO INTRAVASCULAR DIAGNÓSTICO	Respiratory tests	med_respiratoria
Lung	895901	MEDICIÓN DE PRESIÓN DE MUÑÓN CAROTÍDEO	Respiratory tests	med_respiratoria
Lung	895902	MEDICIÓN DE SATURACIÓN VENOSA CEREBRAL	Respiratory tests	med_respiratoria
Lung	895903	AURICULOGRAMA IZQUIERDO	Respiratory tests	med_respiratoria
Lung	895910	PRUEBAS DE FUNCIÓN AUTONÓMICA CARDIOVASCULAR	Respiratory tests	med_respiratoria
Lung	895911	ESTUDIO HEMODINÁMICO NO INVASIVO PARA MEDICIONES VOLUMÉTRICAS INTRATORÁCICAS (CARDIOIMPEDANCIA)	Respiratory tests	med_respiratoria
Lung	895912	ESTUDIO HEMODINÁMICO NO INVASIVO PARA MEDICIONES DE PRESIONES CENTRALES	Respiratory tests	med_respiratoria
Lung	895913	MEDICIÓN NO INVASIVA DE RIGIDEZ VASCULAR	Respiratory tests	med_respiratoria
Lung	345001	TORACENTESIS DIAGNÓSTICA	Thoracentesis	toracentesis
Lung	345002	TORACENTESIS DE DRENAJE O DESCOMPRESIVA	Thoracentesis	toracentesis
Lung	345201	PLEURODESIS QUIMICA VIA ABIERTA	Pleurodesis	pleurodesis
Lung	345202	PLEURODESIS QUIMICA POR TORACOSCOPIA	Pleurodesis	pleurodesis
Lung	345203	PLEURODESIS QUÍMICA POR TORACOSTOMÍA CERRADA	Pleurodesis	pleurodesis
Lung	345204	PLEURODESIS MECÁNICA VÍA ABIERTA	Pleurodesis	pleurodesis
Lung	345205	PLEURODESIS MECÁNICA POR TORACOSCOPIA	Pleurodesis	pleurodesis
Lung	992503	MONOTERAPIA ANTINEOPLÁSICA DE BAJA TOXICIDAD	Monoterapy	monoterapia
Lung	992504	POLITERAPIA ANTINEOPLÁSICA DE BAJA TOXICIDAD	Polyterapy	politerapia
Lung	992505	POLITERAPIA ANTINEOPLÁSICA DE ALTA TOXICIDAD	Polyterapy	politerapia
Lung	992506	ADMINISTRACIÓN (INFUSIÓN O PERFUSIÓN) DE TERAPIA ANTINEOPLÁSICA INTRARTERIAL (REGIONAL)	Monoterapy	monoterapia
Lung	992509	MONOTERAPIA ANTINEOPLÁSICA DE BAJA TOXICIDAD	Monoterapy	monoterapia
Lung	992511	MONOTERAPIA ANTINEOPLÁSICA DE ALTA TOXICIDAD	Monoterapy	monoterapia
Lung	922201	TELETERAPIA CON ORTOVOLTAJE	Teletherapy	teleterapia
Lung	922321	TELETERAPIA CON COBALTO (PLANEACIÓN COMPUTARIZADA BIDIMENSIONAL Y SIMULACIÓN CONVENCIONAL)	Teletherapy	teleterapia
Lung	922322	TELETERAPIA CON COBALTO (PLANEACIÓN COMPUTARIZADA TRIDIMENSIONAL Y SIMULACIÓN VIRTUAL)	Teletherapy	teleterapia

Lung	922441	TELETERAPIA CON ACELERADOR LINEAL (PLANEACIÓN COMPUTARIZADA BIDIMENSIONAL Y SIMULACIÓN CONVENCIONAL) TÉCNICA RADIOTERAPIA CONVENCIONAL	Teletherapy	teleterapia
Lung	922442	TELETERAPIA CON ACELERADOR LINEAL (PLANEACIÓN COMPUTARIZADA TRIDIMENSIONAL Y SIMULACIÓN VIRTUAL) TÉCNICA RADIOTERAPIA CONVENCIONAL	Teletherapy	teleterapia
Lung	922443	TELETERAPIA CON ACELERADOR LINEAL (PLANEACION COMPUTARIZADA TRIDIMENSIONAL Y SIMULACION VIRTUAL) TECNICA CONFORMACIONAL [3D - CRT]	Teletherapy	teleterapia
Lung	922444	TELETERAPIA CON ACELERADOR LINEAL (PLANEACIÓN COMPUTARIZADA TRIDIMENSIONAL Y SIMULACIÓN VIRTUAL) TÉCNICA RADIOTERAPIA DE INTENSIDAD MODULADA [IMRT]	Teletherapy	teleterapia
Lung	922445	TELETERAPIA CON ACELERADOR LINEAL (PLANEACIÓN COMPUTARIZADA TRIDIMENSIONAL Y SIMULACIÓN VIRTUAL) TÉCNICA RADIOTERAPIA GUIADA POR IMÁGENES [IGRT]	Teletherapy	teleterapia
Lung	922446	TELETERAPIA CON ACELERADOR LINEAL (PLANEACIÓN COMPUTARIZADA TRIDIMENSIONAL Y SIMULACIÓN VIRTUAL) TÉCNICA RADIOTERAPIA - ARCOTERAPIA DE MODULACIÓN VOLUMÉTRICA [VMAT]	Teletherapy	teleterapia
Lung	922447	IRRADIACIÓN CORPORAL TOTAL	Irradiation	irradiacion
Lung	922448	IRRADIACIÓN CUTÁNEA TOTAL	Irradiation	irradiacion
Lung	922449	TELETERAPIA CON ACELERADOR LINEAL (PLANEACIÓN COMPUTARIZADA TRIDIMENSIONAL Y SIMULACIÓN VIRTUAL) TÉCNICA RADIOTERAPIA HELICOIDAL	Teletherapy	teleterapia
Lung	922504	TELETERAPIA CON ACELERADOR LINEAL DE ELECTRONES (PLANEACION COMPUTARIZADA BIDIMENSIONAL Y SIMULACION CONVENCIONAL)	Teletherapy	teleterapia
Lung	922505	TELETERAPIA CON ACELERADOR LINEAL DE ELECTRONES (PLANEACION COMPUTARIZADA TRIDIMENSIONAL Y SIMULACIÓN VIRTUAL)	Teletherapy	teleterapia
Lung	922506	RADIOTERAPIA INTRAOPERATORIA	Teletherapy	teleterapia
Lung	922601	BRAQUITERAPIA INTERSTICIAL (PLANEACION COMPUTARIZADA TRIDIMENSIONAL Y SIMULACION VIRTUAL) CON ALTA TASA DE DOSIS	Brachytherapy	braquiterapi a
Lung	922602	BRAQUITERAPIA INTERSTICIAL (PLANEACION COMPUTARIZADA TRIDIMENSIONAL Y SIMULACIÓN VIRTUAL) CON BAJA TASA DE DOSIS	Brachytherapy	braquiterapi a
Lung	922603	BRAQUITERAPIA INTRALUMINAL (PLANEACION COMPUTARIZADA BIDIMENSIONAL Y SIMULACION CONVENCIONAL) CON ALTA TASA DE DOSIS	Brachytherapy	braquiterapi a
Lung	922604	BRAQUITERAPIA INTRALUMINAL CON BAJA TASA DE DOSIS	Brachytherapy	braquiterapi a
Lung	922605	BRAQUITERAPIA INTRACAVITARIA (PLANEACION COMPUTARIZADA BIDIMENSIONAL Y SIMULACION CONVENCIONAL) CON ALTA TASA DE DOSIS	Brachytherapy	braquiterapi a
Lung	922606	BRAQUITERAPIA INTRACAVITARIA (PLANEACION COMPUTARIZADA BIDIMENSIONAL Y SIMULACION CONVENCIONAL) CON BAJA TASA DE DOSIS	Brachytherapy	braquiterapi a
Lung	922607	BRAQUITERAPIA INTRACAVITARIA (PLANEACION COMPUTARIZADA TRIDIMENSIONAL Y SIMULACION VIRTUAL) CON ALTA TASA DE DOSIS	Brachytherapy	braquiterapi a
Lung	922608	BRAQUITERAPIA INTRACAVITARIA (PLANEACIÓN COMPUTARIZADA TRIDIMENSIONAL Y SIMULACIÓN VIRTUAL) CON BAJA TASA DE DOSIS	Brachytherapy	braquiterapi a
Lung	922609	BRAQUITERAPIA INTRALUMINAL (PLANEACIÓN COMPUTARIZADA TRIDIMENSIONAL Y SIMULACIÓN VIRTUAL) CON ALTA TASA DE DOSIS	Brachytherapy	braquiterapi a
Lung	922611	BRAQUITERAPIA DE CONTACTO (PLANEACION COMPUTARIZADA BIDIMENSIONAL Y SIMULACION CONVENCIONAL) CON ALTA TASA DE DOSIS	Brachytherapy	braquiterapi a
Lung	922612	BRAQUITERAPIA DE CONTACTO (PLANEACIÓN COMPUTARIZADA TRIDIMENSIONAL Y SIMULACIÓN VIRTUAL) CON ALTA TASA DE DOSIS	Brachytherapy	braquiterapi a
Lung	922613	BRAQUITERAPIA EPIESCLERAL DE CONTACTO (PLANEACIÓN COMPUTARIZADA TRIDIMENSIONAL Y SIMULACIÓN VIRTUAL) CON BAJA TASA DE DOSIS	Brachytherapy	braquiterapi a
Lung	922614	BRAQUITERAPIA METABÓLICA	Brachytherapy	braquiterapi a
Lung	922615	BRAQUITERAPIA INTERSTICIAL (PLANEACIÓN COMPUTARIZADA BIDIMENSIONAL Y SIMULACIÓN CONVENCIONAL) CON ALTA TASA DE DOSIS	Brachytherapy	braquiterapi a

Lung	922616	BRAQUITERAPIA INTERSTICIAL (PLANEACIÓN COMPUTARIZADA TRIDIMENSIONAL Y SIMULACIÓN VIRTUAL) CON ALTA TASA DE DOSIS	Brachytherapy	braquiterapia
Lung	922800	TERAPIA CON RADIOISOTOPOS SOD	Radiotherapy	terapia
Lung	922801	TERAPIA CON RADIOISÓTOPOS	Radiotherapy	terapia
Lung	922805	TERAPIA CON ITRIUM 90	Radiotherapy	terapia
Lung	922810	TERAPIA CON METAIODOBENCILGUANIDINA SUPRARRENAL	Radiotherapy	terapia
Lung	922830	TERAPIA DE METÁSTASIS CON ESTRONCIO	Radiotherapy	terapia
Prostate	602001	RESECCIÓN O ENUCLEACIÓN TRANSURETRAL DE ADENOMA DE PRÓSTATA IRTUP] O ADENOMECTOMIA	Prostatectomy	prostatectomia
Prostate	602002	ADENOMECTOMIA O PROSTATECTOMIA TRANSVESICAL	Prostatectomy	prostatectomia
Prostate	602003	ADENOMECTOMIA O PROSTATECTOMIA RETROPUBICA O TRANSVESICOCAPSULAR	Prostatectomy	prostatectomia
Prostate	602004	ADENOMECTOMIA O PROSTATECTOMIA POR LAPAROSCOPIA	Prostatectomy	prostatectomia
Prostate	602005	ADENOMECTOMIA POR ABLACION DE PROSTATA	Prostatectomy	prostatectomia
Prostate	605101	RESECCION DE PROSTATA [PROSTATECTOMIA] RADICAL [PROSTATOVESICULECTOMIA]	Prostatectomy	prostatectomia
Prostate	605111	PROSTATECTOMIA RADICAL POR LAPAROSCOPIA	Prostatectomy	prostatectomia
Prostate	605112	PROSTATECTOMIA RADICAL POR ABLACION	Prostatectomy	prostatectomia
Prostate	603100	ADENOMECTOMÍA O PROSTATECTOMÍA TRANSVESICAL	Prostatectomy	prostatectomia
Prostate	602004	ADENOMECTOMÍA O PROSTATECTOMÍA POR LAPAROSCOPIA	Prostatectomy	prostatectomia
Prostate	604001	ADENOMECTOMÍA O PROSTATECTOMÍA RETROPÚBICA O TRANSVESICOCAPSULAR	Prostatectomy	prostatectomia
Prostate	604100	ADENOMECTOMÍA O PROSTATECTOMÍA RETROPÚBICA O TRANSVESICOCAPSULAR	Prostatectomy	prostatectomia
Prostate	602101	ADENOMECTOMÍA POR ABLACIÓN DE PRÓSTATA	Prostatectomy	prostatectomia
Prostate	602005	ADENOMECTOMÍA POR ABLACIÓN DE PRÓSTATA	Prostatectomy	prostatectomia
Prostate	890237	CONSULTA DE PRIMERA VEZ POR ESPECIALISTA EN CIRUGÍA ONCOLÓGICA	Surgical consultation	cons_cirugia
Prostate	890337	CONSULTA DE CONTROL O DE SEGUIMIENTO POR ESPECIALISTA EN CIRUGÍA ONCOLÓGICA	Surgical consultation	cons_cirugia
Prostate	890437	INTERCONSULTA POR ESPECIALISTA EN CIRUGÍA ONCOLÓGICA	Surgical consultation	cons_cirugia
Prostate	890243	CONSULTA DE PRIMERA VEZ POR ESPECIALISTA EN DOLOR Y CUIDADOS PALIATIVOS	Palliative care consultation	cons_paliativo
Prostate	890343	CONSULTA DE CONTROL O DE SEGUIMIENTO POR ESPECIALISTA EN DOLOR Y CUIDADOS PALIATIVOS	Palliative care consultation	cons_paliativo
Prostate	890443	INTERCONSULTA POR ESPECIALISTA EN DOLOR Y CUIDADOS PALIATIVOS	Palliative care consultation	cons_paliativo
Prostate	890287	CONSULTA DE PRIMERA VEZ POR ESPECIALISTA EN RADIOTERAPIA	Radiology consultation	cons_radio

Prostate	890387	CONSULTA DE CONTROL O DE SEGUIMIENTO POR ESPECIALISTA EN RADIOTERAPIA	Radiology consultation	cons_radio
Prostate	890487	INTERCONSULTA POR ESPECIALISTA EN RADIOTERAPIA	Radiology consultation	cons_radio
Prostate	890248	CONSULTA DE PRIMERA VEZ POR ESPECIALISTA EN GENETICA MEDICA	Follow-up consultation	cons_seguim
Prostate	890278	CONSULTA DE PRIMERA VEZ POR ESPECIALISTA EN ONCOLOGÍA	Follow-up consultation	cons_seguim
Prostate	890348	CONSULTA DE CONTROL O DE SEGUIMIENTO POR ESPECIALISTA EN GENETICA MEDICA	Follow-up consultation	cons_seguim
Prostate	890378	CONSULTA DE CONTROL O DE SEGUIMIENTO POR ESPECIALISTA EN ONCOLOGÍA	Follow-up consultation	cons_seguim
Prostate	890448	INTERCONSULTA POR ESPECIALISTA EN GENÉTICA MÉDICA	Follow-up consultation	cons_seguim
Prostate	890478	INTERCONSULTA POR ESPECIALISTA EN ONCOLOGÍA	Follow-up consultation	cons_seguim
Prostate	601101	BIOPSIA CERRADA DE PROSTATA POR ABORDAJE TRANSRECTAL	Screening	diagnostico
Prostate	601102	BIOPSIA CERRADA DE PROSTATA POR ABORDAJE PERINEAL	Screening	diagnostico
Prostate	601103	BIOPSIA CERRADA DE PROSTATA POR SATURACION ABORDAJE TRANSRECTAL	Screening	diagnostico
Prostate	601104	BIOPSIA CERRADA DE PROSTATA POR SATURACION ABORDAJE PERINEAL	Screening	diagnostico
Prostate	601201	BIOPSIA DE PROSTATA VIA ABIERTA	Screening	diagnostico
Prostate	601301	BIOPSIA CERRADA (PERCUTANEA) (AGUJA) DE VESICULAS SEMINALES POR ABORDAJE TRANSRECTAL	Screening	diagnostico
Prostate	601311	BIOPSIA DE VESICULA SEMINAL POR LAPAROSCOPIA	Screening	diagnostico
Prostate	906611	ANTÍGENO ESPECÍFICO DE PRÓSTATA FRACCIÓN LIBRE SEMIAUTOMATIZADO O AUTOMATIZADO	Screening	diagnostico
Prostate	906610	ANTÍGENO ESPECÍFICO DE PRÓSTATA SEMIAUTOMATIZADO O AUTOMATIZADO	Screening	diagnostico
Prostate	906612	ANTÍGENO ESPECÍFICO DE PRÓSTATA VELOCIDAD DE CAMBIO 2 MUESTRAS SEMIAUTOMATIZADO O AUTOMATIZADO	Screening	diagnostico
Prostate	623001	ORQUIECTOMIA CON EPIDIDIDECTOMIA (RADICAL)	Spread procedures	diseminacion
Prostate	441302	ESOFAGOGASTRODUODENOSCOPIA [EGD] CON O SIN BIOPSIA	Spread procedures	diseminacion
Prostate	452301	COLONOSCOPIA TOTAL	Spread procedures	diseminacion
Prostate	452305	COLONOSCOPIA TOTAL CON O SIN BIOPSIA	Spread procedures	diseminacion
Prostate	482101	PROCTOSIGMOIDOSCOPIA TRANSABDOMINAL	Spread procedures	diseminacion
Prostate	501002	BIOPSIA CERRADA (PERCUTANEA) (AGUJA) DE HIGADO	Spread procedures	diseminacion
Prostate	542801	PARACENTESIS ABDOMINAL DIAGNOSTICA VIA PERCUTANEA	Spread procedures	diseminacion
Prostate	542802	PARACENTESIS ABDOMINAL TERAPEUTICA VIA PERCUTANEA	Spread procedures	diseminacion
Prostate	543301	ESCISION DE LESION AMPLIA EN LA PARED ABDOMINAL CON ROTACION DE COLGAJO	Spread procedures	diseminacion
Prostate	547503	PLASTIA DE PARED ABDOMINAL VIA ABIERTA	Spread procedures	diseminacion
Prostate	547505	RECONSTRUCCION DE PARED ABDOMINAL ANATOMICA Y FUNCIONAL VIA ABIERTA	Spread procedures	diseminacion

Prostate	872002	RADIOGRAFIA DE ABDOMEN SIMPLE	Spread procedures	diseminacio n
Prostate	872011	RADIOGRAFIA DE ABDOMEN SIMPLE CON PROYECCIONES ADICIONALES (SERIE DE ABDOMEN AGUDO)	Spread procedures	diseminacio n
Prostate	879111	TOMOGRAFIA COMPUTADA DE CRANEO SIMPLE	Spread procedures	diseminacio n
Prostate	879112	TOMOGRAFIA COMPUTADA DE CRANEO CON CONTRASTE	Spread procedures	diseminacio n
Prostate	879113	TOMOGRAFIA COMPUTADA DE CRANEO SIMPLE Y CON CONTRASTE	Spread procedures	diseminacio n
Prostate	879201	TOMOGRAFIA COMPUTADA DE COLUMNA SEGMENTOS CERVICAL, TORACICO, LUMBAR O SACRO, POR CADA NIVEL (TRES ESPACIOS)	Spread procedures	diseminacio n
Prostate	879205	TOMOGRAFIA COMPUTADA DE COLUMNA SEGMENTOS CERVICAL, TORACICO, LUMBAR O SACRO, COMPLEMENTO A MIELOGRAFIA (CADA SEGMENTO)	Spread procedures	diseminacio n
Prostate	879410	TOMOGRAFÍA COMPUTADA DE ABDOMEN SUPERIOR	Spread procedures	diseminacio n
Prostate	879420	TOMOGRAFIA COMPUTADA DE ABDOMEN Y PELVIS (ABDOMEN TOTAL)	Spread procedures	diseminacio n
Prostate	879460	TOMOGRAFIA COMPUTADA DE PELVIS	Spread procedures	diseminacio n
Prostate	881213	ECOGRAFIA ENDOSCOPICA DE MEDIASTINO, TRAQUEA Y BRONQUIOS	Spread procedures	diseminacio n
Prostate	881301	ECOGRAFIA DE TEJIDOS BLANDOS DE PARED ABDOMINAL Y DE PELVIS	Spread procedures	diseminacio n
Prostate	881302	ECOGRAFIA DE ABDOMEN TOTAL	Spread procedures	diseminacio n
Prostate	881302	ECOGRAFIA DE ABDOMEN TOTAL	Spread procedures	diseminacio n
Prostate	881305	ECOGRAFIA DE ABDOMEN SUPERIOR	Spread procedures	diseminacio n
Prostate	881306	ECOGRAFIA DE HIGADO, PANCREAS, VIA BILIAR Y VESICULA	Spread procedures	diseminacio n
Prostate	883101	RESONANCIA MAGNETICA DE CEREBRO	Spread procedures	diseminacio n
Prostate	883401	RESONANCIA MAGNETICA DE ABDOMEN	Spread procedures	diseminacio n
Prostate	886011	OSTEODENSITOMETRIA POR TC	Spread procedures	diseminacio n
Prostate	886012	OSTEODENSITOMETRIA POR ABSORCION DUAL	Spread procedures	diseminacio n
Prostate	886013	OSTEODENSITOMETRIA Y COMPOSICION CORPORAL (TEJIDOS BLANDOS)	Spread procedures	diseminacio n
Prostate	903809	BILIRRUBINAS TOTAL Y DIRECTA	Spread procedures	diseminacio n
Prostate	903856	NITROGENO UREICO	Spread procedures	diseminacio n
Prostate	903857	NITROGENO UREICO EN ORINA DE 24 HORAS	Spread procedures	diseminacio n

Prostate	903866	TRANSAMINASA GLUTAMICO-PIRUVICA [ALANINO AMINO TRANSFERASA]	Spread procedures	diseminacio n
Prostate	903867	TRANSAMINASA GLUTAMICO OXALACETICA [ASPARTATO AMINO TRANSFERASA]	Spread procedures	diseminacio n
Prostate	903876	CREATININA EN ORINA PARCIAL	Spread procedures	diseminacio n
Prostate	903881	CREATINA EN ORINA DE 24 HORAS	Spread procedures	diseminacio n
Prostate	903895	CREATININA EN SUERO U OTROS FLUIDOS	Spread procedures	diseminacio n
Prostate	890602	CUIDADO (MANEJO) INTRAHOSPITALARIO POR MEDICINA ESPECIALIZADA	Inpatient services	internacion
Prostate	107M01	INTERNACIÓN EN UNIDAD DE CUIDADO INTERMEDIO ADULTO	Inpatient services	internacion
Prostate	10A001	INTERNACIÓN COMPLEJIDAD ALTA HABITACION UNIPERSONAL (INCLUYE AISLAMIENTO)	Inpatient services	internacion
Prostate	10A002	INTERNACIÓN COMPLEJIDAD ALTA HABITACION BIPERSONAL	Inpatient services	internacion
Prostate	10A003	INTERNACIÓN COMPLEJIDAD ALTA TRES CAMAS	Inpatient services	internacion
Prostate	10A004	INTERNACIÓN COMPLEJIDAD ALTA CUATRO O MÁS CAMAS	Inpatient services	internacion
Prostate	10A005	INTERNACIÓN EN UNIDAD DE TRASPLANTE	Inpatient services	internacion
Prostate	10M001	INTERNACIÓN COMPLEJIDAD MEDIANA HABITACION UNIPERSONAL (INCLUYE AISLAMIENTO)	Inpatient services	internacion
Prostate	10M002	INTERNACIÓN COMPLEJIDAD MEDIANA HABITACION BIPERSONAL	Inpatient services	internacion
Prostate	10M003	INTERNACIÓN COMPLEJIDAD MEDIANA HABITACION TRES CAMAS	Inpatient services	internacion
Prostate	10M004	INTERNACIÓN COMPLEJIDAD MEDIANA HABITACION CUATRO O MÁS CAMAS	Inpatient services	internacion
Prostate	110A01	INTERNACIÓN EN UNIDAD DE CUIDADO INTENSIVO ADULTOS	Inpatient services	internacion
Prostate	121M01	INTERNACIÓN DE PACIENTE CRÓNICO TERMINAL SIN VENTILADOR COMPLEJIDAD MEDIANA	Inpatient services	internacion
Prostate	121M02	INTERNACIÓN DE PACIENTE CRÓNICO TERMINAL CON VENTILADOR COMPLEJIDAD MEDIANA	Inpatient services	internacion
Prostate	S11201	INTERNACIÓN COMPLEJIDAD MEDIANA HABITACION UNIPERSONAL (INCLUYE AISLAMIENTO)	Inpatient services	internacion
Prostate	S11202	INTERNACIÓN COMPLEJIDAD MEDIANA HABITACION BIPERSONAL	Inpatient services	internacion
Prostate	S11203	INTERNACIÓN COMPLEJIDAD MEDIANA HABITACION TRES CAMAS	Inpatient services	internacion
Prostate	S11204	INTERNACIÓN COMPLEJIDAD MEDIANA HABITACION CUATRO O MÁS CAMAS	Inpatient services	internacion
Prostate	S11301	INTERNACIÓN COMPLEJIDAD ALTA HABITACION UNIPERSONAL (INCLUYE AISLAMIENTO)	Inpatient services	internacion
Prostate	S11302	INTERNACIÓN COMPLEJIDAD ALTA HABITACION BIPERSONAL	Inpatient services	internacion
Prostate	S11303	INTERNACIÓN COMPLEJIDAD ALTA TRES CAMAS	Inpatient services	internacion
Prostate	S11304	INTERNACIÓN COMPLEJIDAD ALTA CUATRO O MÁS CAMAS	Inpatient services	internacion
Prostate	S12103	INTERNACIÓN EN UNIDAD DE CUIDADO INTENSIVO ADULTOS	Inpatient services	internacion
Prostate	S12203	INTERNACIÓN EN UNIDAD DE CUIDADO INTERMEDIO ADULTO	Inpatient services	internacion
Prostate	S12600	INTERNACIÓN EN UNIDAD DE TRASPLANTE	Inpatient services	internacion
Prostate	898003	ESTUDIO DE COLORACION BASICA EN CITOLOGIA POR ASPIRACION DE CUALQUIER TEJIDO U ORGANO [BACAF]	Laboratory tests	laboratorio
Prostate	898007	ESTUDIO DE COLORACION HISTOQUIMICA EN CITOLOGIA POR ASPIRACION DE CUALQUIER TEJIDO U ORGANO [BACAF]	Laboratory tests	laboratorio
Prostate	898011	ESTUDIO DE COLORACION INMUNOHISTOQUIMICA EN CITOLOGIA POR ASPIRACION DE CUALQUIER TEJIDO U ORGANO [BACAF]	Laboratory tests	laboratorio
Prostate	898033	ESTUDIO DE RECEPTORES HORMONALES EN CITOLOGIA	Laboratory tests	laboratorio
Prostate	898101	ESTUDIO DE COLORACION BASICA EN BIOPSIA	Laboratory tests	laboratorio
Prostate	898102	ESTUDIO DE COLORACION HISTOQUIMICA EN BIOPSIA	Laboratory tests	laboratorio
Prostate	898103	ESTUDIO DE COLORACION INMUNOHISTOQUIMICA EN BIOPSIA	Laboratory tests	laboratorio
Prostate	898104	ESTUDIO DE COLORACION DE INMUNOFLUORESCENCIA EN BIOPSIA	Laboratory tests	laboratorio
Prostate	898105	ESTUDIO DE BIOLOGIA MOLECULAR EN BIOPSIA	Laboratory tests	laboratorio
Prostate	898106	ESTUDIO DE CITOMETRIA DE FLUJO EN BIOPSIA	Laboratory tests	laboratorio
Prostate	898107	ESTUDIO DE MICROSCOPIA ELECTRONICA EN BIOPSIA	Laboratory tests	laboratorio

Prostate	898110	ESTUDIO DE RECEPTORES HORMONALES EN BIOPSIA	Laboratory tests	laboratorio
Prostate	898201	ESTUDIO DE COLORACION BASICA EN ESPECIMEN DE RECONOCIMIENTO	Laboratory tests	laboratorio
Prostate	898202	ESTUDIO DE COLORACION HISTOQUIMICA EN ESPECIMEN DE RECONOCIMIENTO	Laboratory tests	laboratorio
Prostate	898203	ESTUDIO DE COLORACION INMUNOHISTOQUIMICA EN ESPECIMEN DE RECONOCIMIENTO	Laboratory tests	laboratorio
Prostate	898205	ESTUDIO DE BIOLOGIA MOLECULAR EN ESPECIMEN DE RECONOCIMIENTO	Laboratory tests	laboratorio
Prostate	898210	ESTUDIO DE RECEPTORES HORMONALES EN ESPECIMEN DE RECONOCIMIENTO	Laboratory tests	laboratorio
Prostate	898221	ESTUDIO DE COLORACION BASICA EN ESPECIMEN CON MULTIPLE MUESTREO	Laboratory tests	laboratorio
Prostate	898222	ESTUDIO DE COLORACION HISTOQUIMICA EN ESPECIMEN CON MULTIPLE MUESTREO	Laboratory tests	laboratorio
Prostate	898226	ESTUDIO DE CITOMETRIA DE FLUJO EN ESPECIMEN CON MULTIPLE MUESTREO	Laboratory tests	laboratorio
Prostate	898230	ESTUDIO DE RECEPTORES HORMONALES EN ESPECIMEN CON MULTIPLE MUESTREO	Laboratory tests	laboratorio
Prostate	898241	ESTUDIO DE COLORACION BASICA EN ESPECIMEN CON RESECCION DE MARGENES	Laboratory tests	laboratorio
Prostate	898242	ESTUDIO DE COLORACION HISTOQUIMICA EN ESPECIMEN CON RESECCION DE MARGENES	Laboratory tests	laboratorio
Prostate	898243	ESTUDIO DE COLORACION INMUNOHISTOQUIMICA EN ESPECIMEN CON RESECCION DE MARGENES	Laboratory tests	laboratorio
Prostate	898250	ESTUDIO DE RECEPTORES HORMONALES EN ESPECIMEN CON RESECCION DE MARGENES	Laboratory tests	laboratorio
Prostate	898262	ESTUDIO DE COLORACION HISTOQUIMICA EN ESPECIMEN CON MAPEO	Laboratory tests	laboratorio
Prostate	908420	ESTUDIOS MOLECULARES DE GENES (ESPECIFICOS)	Laboratory tests	laboratorio
Prostate	920901	GAMAGRAFIA OSEA (CORPORAL TOTAL O SEGMENTARIA)	Laboratory tests	laboratorio
Prostate	920902	GAMAGRAFIA OSEA DE TRES FASES	Laboratory tests	laboratorio
Prostate	920903	GAMAGRAFIA OSEA CON SPECT	Laboratory tests	laboratorio
Prostate	992503	MONOTERAPIA ANTINEOPLÁSICA DE BAJA TOXICIDAD	Monoterapy	monoterapia
Prostate	992506	ADMINISTRACIÓN (INFUSIÓN O PERFUSIÓN) DE TERAPIA ANTINEOPLÁSICA INTRARTERIAL (REGIONAL)	Monoterapy	monoterapia
Prostate	992509	MONOTERAPIA ANTINEOPLÁSICA DE BAJA TOXICIDAD	Monoterapy	monoterapia
Prostate	992511	MONOTERAPIA ANTINEOPLÁSICA DE ALTA TOXICIDAD	Monoterapy	monoterapia
Prostate	992504	POLITERAPIA ANTINEOPLÁSICA DE BAJA TOXICIDAD	Polyterapy	politerapia
Prostate	992505	POLITERAPIA ANTINEOPLÁSICA DE ALTA TOXICIDAD	Polyterapy	politerapia
Prostate	922601	BRAQUITERAPIA INTERSTICIAL (PLANEACION COMPUTARIZADA TRIDIMENSIONAL Y SIMULACION VIRTUAL) CON ALTA TASA DE DOSIS	Brachytherapy	braquiterapi a
Prostate	922602	BRAQUITERAPIA INTERSTICIAL (PLANEACION COMPUTARIZADA TRIDIMENSIONAL Y SIMULACIÓN VIRTUAL) CON BAJA TASA DE DOSIS	Brachytherapy	braquiterapi a
Prostate	922603	BRAQUITERAPIA INTRALUMINAL (PLANEACION COMPUTARIZADA BIDIMENSIONAL Y SIMULACION CONVENCIONAL) CON ALTA TASA DE DOSIS	Brachytherapy	braquiterapi a
Prostate	922604	BRAQUITERAPIA INTRALUMINAL CON BAJA TASA DE DOSIS	Brachytherapy	braquiterapi a
Prostate	922605	BRAQUITERAPIA INTRACAVITARIA (PLANEACION COMPUTARIZADA BIDIMENSIONAL Y SIMULACION CONVENCIONAL) CON ALTA TASA DE DOSIS	Brachytherapy	braquiterapi a
Prostate	922606	BRAQUITERAPIA INTRACAVITARIA (PLANEACION COMPUTARIZADA BIDIMENSIONAL Y SIMULACION CONVENCIONAL) CON BAJA TASA DE DOSIS	Brachytherapy	braquiterapi a
Prostate	922607	BRAQUITERAPIA INTRACAVITARIA (PLANEACION COMPUTARIZADA TRIDIMENSIONAL Y SIMULACION VIRTUAL) CON ALTA TASA DE DOSIS	Brachytherapy	braquiterapi a
Prostate	922608	BRAQUITERAPIA INTRACAVITARIA (PLANEACIÓN COMPUTARIZADA TRIDIMENSIONAL Y SIMULACIÓN VIRTUAL) CON BAJA TASA DE DOSIS	Brachytherapy	braquiterapi a
Prostate	922609	BRAQUITERAPIA INTRALUMINAL (PLANEACIÓN COMPUTARIZADA TRIDIMENSIONAL Y SIMULACIÓN VIRTUAL) CON ALTA TASA DE DOSIS	Brachytherapy	braquiterapi a
Prostate	922611	BRAQUITERAPIA DE CONTACTO (PLANEACION COMPUTARIZADA BIDIMENSIONAL Y SIMULACION CONVENCIONAL) CON ALTA TASA DE DOSIS	Brachytherapy	braquiterapi a
Prostate	922612	BRAQUITERAPIA DE CONTACTO (PLANEACIÓN COMPUTARIZADA TRIDIMENSIONAL Y SIMULACIÓN VIRTUAL) CON ALTA TASA DE DOSIS	Brachytherapy	braquiterapi a

Prostate	922613	BRAQUITERAPIA EPIESCLERAL DE CONTACTO (PLANEACIÓN COMPUTARIZADA TRIDIMENSIONAL Y SIMULACIÓN VIRTUAL) CON BAJA TASA DE DOSIS	Brachytherapy	braquiterapia
Prostate	922614	BRAQUITERAPIA METABÓLICA	Brachytherapy	braquiterapia
Prostate	922615	BRAQUITERAPIA INTERSTICIAL (PLANEACIÓN COMPUTARIZADA BIDIMENSIONAL Y SIMULACIÓN CONVENCIONAL) CON ALTA TASA DE DOSIS	Brachytherapy	braquiterapia
Prostate	922616	BRAQUITERAPIA INTERSTICIAL (PLANEACIÓN COMPUTARIZADA TRIDIMENSIONAL Y SIMULACIÓN VIRTUAL) CON ALTA TASA DE DOSIS	Brachytherapy	braquiterapia
Prostate	922447	IRRADIACIÓN CORPORAL TOTAL	Irradiation	irradiacion
Prostate	922448	IRRADIACIÓN CUTÁNEA TOTAL	Irradiation	irradiacion
Prostate	922201	TELETERAPIA CON ORTOVOLTAJE	Teletherapy	teleterapia
Prostate	922321	TELETERAPIA CON COBALTO (PLANEACIÓN COMPUTARIZADA BIDIMENSIONAL Y SIMULACIÓN CONVENCIONAL)	Teletherapy	teleterapia
Prostate	922322	TELETERAPIA CON COBALTO (PLANEACIÓN COMPUTARIZADA TRIDIMENSIONAL Y SIMULACIÓN VIRTUAL)	Teletherapy	teleterapia
Prostate	922441	TELETERAPIA CON ACELERADOR LINEAL (PLANEACIÓN COMPUTARIZADA BIDIMENSIONAL Y SIMULACIÓN CONVENCIONAL) TÉCNICA RADIOTERAPIA CONVENCIONAL	Teletherapy	teleterapia
Prostate	922442	TELETERAPIA CON ACELERADOR LINEAL (PLANEACIÓN COMPUTARIZADA TRIDIMENSIONAL Y SIMULACIÓN VIRTUAL) TÉCNICA RADIOTERAPIA CONVENCIONAL	Teletherapy	teleterapia
Prostate	922443	TELETERAPIA CON ACELERADOR LINEAL (PLANEACION COMPUTARIZADA TRIDIMENSIONAL Y SIMULACION VIRTUAL) TECNICA CONFORMACIONAL [3D - CRT]	Teletherapy	teleterapia
Prostate	922444	TELETERAPIA CON ACELERADOR LINEAL (PLANEACIÓN COMPUTARIZADA TRIDIMENSIONAL Y SIMULACIÓN VIRTUAL) TÉCNICA RADIOTERAPIA DE INTENSIDAD MODULADA [IMRT]	Teletherapy	teleterapia
Prostate	922445	TELETERAPIA CON ACELERADOR LINEAL (PLANEACIÓN COMPUTARIZADA TRIDIMENSIONAL Y SIMULACIÓN VIRTUAL) TÉCNICA RADIOTERAPIA GUIADA POR IMÁGENES [IGRT]	Teletherapy	teleterapia
Prostate	922446	TELETERAPIA CON ACELERADOR LINEAL (PLANEACIÓN COMPUTARIZADA TRIDIMENSIONAL Y SIMULACIÓN VIRTUAL) TÉCNICA RADIOTERAPIA - ARCOTERAPIA DE MODULACIÓN VOLUMÉTRICA [VMAT]	Teletherapy	teleterapia
Prostate	922449	TELETERAPIA CON ACELERADOR LINEAL (PLANEACIÓN COMPUTARIZADA TRIDIMENSIONAL Y SIMULACIÓN VIRTUAL) TÉCNICA RADIOTERAPIA HELICOIDAL	Teletherapy	teleterapia
Prostate	922504	TELETERAPIA CON ACELERADOR LINEAL DE ELECTRONES (PLANEACION COMPUTARIZADA BIDIMENSIONAL Y SIMULACION CONVENCIONAL)	Teletherapy	teleterapia
Prostate	922505	TELETERAPIA CON ACELERADOR LINEAL DE ELECTRONES (PLANEACION COMPUTARIZADA TRIDIMENSIONAL Y SIMULACIÓN VIRTUAL)	Teletherapy	teleterapia
Prostate	922506	RADIOTERAPIA INTRAOPERATORIA	Teletherapy	teleterapia
Prostate	922800	TERAPIA CON RADIOISOTOPOS SOD	Radiotherapy	terapia
Prostate	922801	TERAPIA CON RADIOISÓTOPOS	Radiotherapy	terapia
Prostate	922805	TERAPIA CON ITRIUM 90	Radiotherapy	terapia
Prostate	922810	TERAPIA CON METAIODOBENCILGUANIDINA SUPRARRENAL	Radiotherapy	terapia
Prostate	922830	TERAPIA DE METÁSTASIS CON ESTRONCIO	Radiotherapy	terapia
Stomach	890234	CONSULTA DE PRIMERA VEZ POR ESPECIALISTA EN CIRUGÍA GASTROINTESTINAL	Surgical consultation	cons_cirugia
Stomach	890237	CONSULTA DE PRIMERA VEZ POR ESPECIALISTA EN CIRUGÍA ONCOLÓGICA	Surgical consultation	cons_cirugia
Stomach	890243	CONSULTA DE PRIMERA VEZ POR ESPECIALISTA EN DOLOR Y CUIDADOS PALIATIVOS	Palliative care consultation	cons_paliativo
Stomach	890246	CONSULTA DE PRIMERA VEZ POR ESPECIALISTA EN GASTROENTEROLOGIA	Follow-up consultation	cons_seguim
Stomach	890248	CONSULTA DE PRIMERA VEZ POR ESPECIALISTA EN GENETICA MEDICA	Follow-up consultation	cons_seguim
Stomach	890268	CONSULTA DE PRIMERA VEZ POR ESPECIALISTA EN NEFROLOGÍA	Follow-up consultation	cons_seguim

Stomach	890278	CONSULTA DE PRIMERA VEZ POR ESPECIALISTA EN ONCOLOGÍA	Follow-up consultation	cons_seguim
Stomach	890287	CONSULTA DE PRIMERA VEZ POR ESPECIALISTA EN RADIOTERAPIA	Radiology consultation	cons_radio
Stomach	890334	CONSULTA DE CONTROL O DE SEGUIMIENTO POR ESPECIALISTA EN CIRUGÍA GASTROINTESTINAL	Surgical consultation	cons_cirugia
Stomach	890337	CONSULTA DE CONTROL O DE SEGUIMIENTO POR ESPECIALISTA EN CIRUGÍA ONCOLÓGICA	Surgical consultation	cons_cirugia
Stomach	890343	CONSULTA DE CONTROL O DE SEGUIMIENTO POR ESPECIALISTA EN DOLOR Y CUIDADOS PALIATIVOS	Palliative care consultation	cons_paliativo
Stomach	890346	CONSULTA DE CONTROL O DE SEGUIMIENTO POR ESPECIALISTA EN GASTROENTEROLOGÍA	Follow-up consultation	cons_seguim
Stomach	890348	CONSULTA DE CONTROL O DE SEGUIMIENTO POR ESPECIALISTA EN GENETICA MEDICA	Follow-up consultation	cons_seguim
Stomach	890368	CONSULTA DE CONTROL O DE SEGUIMIENTO POR ESPECIALISTA EN NEFROLOGÍA	Follow-up consultation	cons_seguim
Stomach	890378	CONSULTA DE CONTROL O DE SEGUIMIENTO POR ESPECIALISTA EN ONCOLOGÍA	Follow-up consultation	cons_seguim
Stomach	890387	CONSULTA DE CONTROL O DE SEGUIMIENTO POR ESPECIALISTA EN RADIOTERAPIA	Radiology consultation	cons_radio
Stomach	890434	INTERCONSULTA POR ESPECIALISTA EN CIRUGÍA GASTROINTESTINAL	Surgical consultation	cons_cirugia
Stomach	890437	INTERCONSULTA POR ESPECIALISTA EN CIRUGÍA ONCOLÓGICA	Surgical consultation	cons_cirugia
Stomach	890443	INTERCONSULTA POR ESPECIALISTA EN DOLOR Y CUIDADOS PALIATIVOS	Palliative care consultation	cons_paliativo
Stomach	890446	INTERCONSULTA POR ESPECIALISTA EN GASTROENTEROLOGÍA	Follow-up consultation	cons_seguim
Stomach	890448	INTERCONSULTA POR ESPECIALISTA EN GENÉTICA MÉDICA	Follow-up consultation	cons_seguim
Stomach	890468	INTERCONSULTA POR ESPECIALISTA EN NEFROLOGÍA	Follow-up consultation	cons_seguim
Stomach	890478	INTERCONSULTA POR ESPECIALISTA EN ONCOLOGÍA	Follow-up consultation	cons_seguim
Stomach	890487	INTERCONSULTA POR ESPECIALISTA EN RADIOTERAPIA	Radiology consultation	cons_radio
Stomach	541101	LAPAROTOMÍA DE PRECISIÓN (ESTADIFICACIÓN)	Screening	diagnostico
Stomach	541102	LAPAROTOMIA EXPLORATORIA	Screening	diagnostico
Stomach	541200	LAPAROTOMIA EXPLORATORIA SOD	Screening	diagnostico
Stomach	542100	LAPAROSCOPIA DIAGNOSTICA SOD	Screening	diagnostico
Stomach	542101	LAPAROSCOPIA EXPLORATORIA	Screening	diagnostico
Stomach	542102	LAPAROSCOPIA DE PRECISION (ESTADIFICACION)	Screening	diagnostico
Stomach	881701	ECOGRAFÍA COMO GUÍA PARA PROCEDIMIENTOS	Screening	diagnostico
Stomach	348202	SUTURA DE LACERACIÓN DIAFRAGMÁTICA VÍA ABDOMINAL POR LAPAROTOMÍA	Spread procedures	diseminacion
Stomach	348204	SUTURA DE LACERACION DIAFRAGMATICA TRANSTORACICA VIA ABIERTA	Spread procedures	diseminacion
Stomach	413101	BIOPSIA POR ASPIRACION DE MEDULA OSEA	Spread procedures	diseminacion

Stomach	422003	ESOFAGOSCOPIA VIA ORAL EXPLORATORIA O DIAGNOSTICA SIN BIOPSIA	Spread procedures	diseminacio n
Stomach	423301	POLIPECTOMIA DE ESOFAGO VIA ENDOSCOPICA	Spread procedures	diseminacio n
Stomach	425101	ESOFAGOESOFAGOSTOMIA INTRATORACICA O CERVICAL VIA ABIERTA	Spread procedures	diseminacio n
Stomach	429209	DILATACION ESOFAGICA CON DISPOSITIVO	Spread procedures	diseminacio n
Stomach	436100	GASTRODUODENOSTOMIA SOD	Spread procedures	diseminacio n
Stomach	437100	GASTROYEYUNOSTOMIA SOD	Spread procedures	diseminacio n
Stomach	437101	GASTROYEYUNOSTOMIA VIA ABIERTA	Spread procedures	diseminacio n
Stomach	438301	GASTROENTEROANASTOMOSIS DERIVATIVA (DUODENO O YEYUNO) CON EXCLUSION PILORICA VIA ABIERTA	Spread procedures	diseminacio n
Stomach	438302	GASTROENTEROANASTOMOSIS DERIVATIVA (DUODENO O YEYUNO) CON EXCLUSIÓN PILÓRICA VÍA LAPAROSCÓPICA	Spread procedures	diseminacio n
Stomach	438303	GASTROENTEROANASTOMOSIS DERIVATIVA (DUODENO O YEYUNO) SIN EXCLUSIÓN PILÓRICA VÍA ABIERTA	Spread procedures	diseminacio n
Stomach	438304	GASTROENTEROANASTOMOSIS DERIVATIVA (DUODENO O YEYUNO) SIN EXCLUSIÓN PILÓRICA VÍA LAPAROSCÓPICA	Spread procedures	diseminacio n
Stomach	438401	GASTRECTOMÍA VERTICAL [MANGA GÁSTRICA] VÍA ABIERTA	Spread procedures	diseminacio n
Stomach	438402	GASTRECTOMÍA VERTICAL [MANGA GÁSTRICA] POR LAPAROSCOPIA	Spread procedures	diseminacio n
Stomach	438501	REINTERVENCIÓN O REVISIÓN DE GASTRECTOMÍA VERTICAL [MANGA GÁSTRICA] VÍA ABIERTA	Spread procedures	diseminacio n
Stomach	438502	REINTERVENCIÓN O REVISIÓN DE GASTRECTOMÍA VERTICAL [MANGA GÁSTRICA] POR LAPAROSCOPIA	Spread procedures	diseminacio n
Stomach	438503	CONVERSIÓN DE GASTRECTOMÍA VERTICAL [MANGA GÁSTRICA] A OTRA CIRUGÍA VÍA ABIERTA	Spread procedures	diseminacio n
Stomach	438504	CONVERSIÓN DE GASTRECTOMÍA VERTICAL [MANGA GÁSTRICA] A OTRA CIRUGÍA POR LAPAROSCOPIA	Spread procedures	diseminacio n
Stomach	439300	ESOFAGOGASTRECTOMIA SOD	Spread procedures	diseminacio n
Stomach	439301	ESOFAGOGASTRECTOMIA VIA ABIERTA	Spread procedures	diseminacio n
Stomach	439302	ESOFAGOGASTRECTOMÍA VÍA LAPAROSCÓPICA	Spread procedures	diseminacio n
Stomach	441302	ESOFAGOGASTRODUODENOSCOPIA [EGD] CON O SIN BIOPSIA	Spread procedures	diseminacio n
Stomach	441303	ESOFAGOGASTRODUODENOSCOPIA [EGD] CON MAGNIFICACION O CROMOENDOSCOPIA	Spread procedures	diseminacio n
Stomach	451600	ESOFAGOGASTRODUODENOSCOPIA [EGD] CON O SIN BIOPSIA	Spread procedures	diseminacio n
Stomach	452301	COLONOSCOPIA TOTAL	Spread procedures	diseminacio n

Stomach	452305	COLONOSCOPIA TOTAL CON O SIN BIOPSIA	Spread procedures	diseminacio n
Stomach	459101	ANASTOMOSIS DE INTESTINO DELGADO A INTESTINO DELGADO VIA ABIERTA	Spread procedures	diseminacio n
Stomach	482101	PROCTOSIGMOIDOSCOPIA TRANSABDOMINAL	Spread procedures	diseminacio n
Stomach	501002	BIOPSIA CERRADA (PERCUTANEA) (AGUJA) DE HIGADO	Spread procedures	diseminacio n
Stomach	540014	DRENAJE DE COLECCION INTRAPERITONEAL VIA PERCUTANEA	Spread procedures	diseminacio n
Stomach	541506	RESECCION DE TUMOR RETROPERITONEAL CON DISECCION DE ESTRUCTURAS VASCULARES U ORGANOS RETROPERITONEALES VIA ABIERTA	Spread procedures	diseminacio n
Stomach	541701	LAVADO PERITONEAL TERAPEUTICO VIA ABIERTA	Spread procedures	diseminacio n
Stomach	542302	BIOPSIA DE PERITONEO VIA ABIERTA	Spread procedures	diseminacio n
Stomach	542304	BIOPSIA DE PERITONEO VIA LAPAROSCOPICA	Spread procedures	diseminacio n
Stomach	542801	PARACENTESIS ABDOMINAL DIAGNOSTICA VIA PERCUTANEA	Spread procedures	diseminacio n
Stomach	542802	PARACENTESIS ABDOMINAL TERAPEUTICA VIA PERCUTANEA	Spread procedures	diseminacio n
Stomach	542803	PARACENTESIS ABDOMINAL TERAPEUTICA VIA LAPAROSCOPICA	Spread procedures	diseminacio n
Stomach	543101	RESECCIÓN DE TUMOR BENIGNO EN LA PARED ABDOMINAL VÍA ABIERTA	Spread procedures	diseminacio n
Stomach	543201	RESECCIÓN DE TUMOR MALIGNO EN LA PARED ABDOMINAL VÍA ABIERTA	Spread procedures	diseminacio n
Stomach	543202	RESECCIÓN DE TUMOR MALIGNO EN LA PARED ABDOMINAL VÍA LAPAROSCÓPICA	Spread procedures	diseminacio n
Stomach	544101	OMENTECTOMIA PARCIAL	Spread procedures	diseminacio n
Stomach	544102	OMENTECTOMIA TOTAL	Spread procedures	diseminacio n
Stomach	544104	OMENTECTOMÍA PARCIAL VÍA ABIERTA	Spread procedures	diseminacio n
Stomach	544105	OMENTECTOMÍA PARCIAL VÍA LAPAROSCÓPICA	Spread procedures	diseminacio n
Stomach	544106	OMENTECTOMÍA TOTAL VÍA ABIERTA	Spread procedures	diseminacio n
Stomach	544107	OMENTECTOMÍA TOTAL VÍA LAPAROSCÓPICA	Spread procedures	diseminacio n
Stomach	545000	LISIS DE ADHERENCIAS PERITONEALES POR LAPAROTOMIA SOD	Spread procedures	diseminacio n
Stomach	545100	LISIS DE ADHERENCIAS PERITONEALES POR LAPAROSCOPIA SOD	Spread procedures	diseminacio n
Stomach	871121	RADIOGRAFIA DE TORAX (P.A. O A.P. Y LATERAL, DECUBITO LATERAL, OBLICUAS O LATERAL)	Spread procedures	diseminacio n

Stomach	872101	RADIOGRAFIA DE TRANSITO INTESTINAL CONVENCIONAL	Spread procedures	diseminacio n
Stomach	879111	TOMOGRAFIA COMPUTADA DE CRANEO SIMPLE	Spread procedures	diseminacio n
Stomach	879112	TOMOGRAFIA COMPUTADA DE CRANEO CON CONTRASTE	Spread procedures	diseminacio n
Stomach	879113	TOMOGRAFIA COMPUTADA DE CRANEO SIMPLE Y CON CONTRASTE	Spread procedures	diseminacio n
Stomach	879391	TOMOGRAFIA COMPUTADA DE TORAX EXTENDIDO AL ABDOMEN SUPERIOR CON SUPRARRENALES	Spread procedures	diseminacio n
Stomach	881211	ECOGRAFIA DE TORAX (PERICARDIO O PLEURA)	Spread procedures	diseminacio n
Stomach	883101	RESONANCIA MAGNETICA DE CEREBRO	Spread procedures	diseminacio n
Stomach	434200	RESECCIÓN DE LESIÓN O TUMOR SUBMUCOSO GÁSTRICO VÍA ENDOSCÓPICA	Endoscopy	endoscopia_ diges
Stomach	434201	RESECCIÓN DE LESIÓN O TUMOR SUBMUCOSO GÁSTRICO VÍA ENDOSCÓPICA	Endoscopy	endoscopia_ diges
Stomach	451100	ENDOSCOPIA TRANSABDOMINAL DE INTESTINO DELGADO SOD	Endoscopy	endoscopia_ diges
Stomach	451200	ENDOSCOPIA DE INTESTINO DELGADO A TRAVÉS DE ESTOMA ARTIFICIAL SOD	Endoscopy	endoscopia_ diges
Stomach	451302	ENTEROSCOPIA O ENDOSCOPIA DE INTESTINO DELGADO DESPUÉS DE DUODENO	Endoscopy	endoscopia_ diges
Stomach	451303	INSERCIÓN ENDOSCÓPICA DE DISPOSTIVO DUODENAL	Endoscopy	endoscopia_ diges
Stomach	451304	ENTEROSCOPIA (INTRAQUIRÚRGICA) TRANSABDOMINAL VÍA ABIERTA	Endoscopy	endoscopia_ diges
Stomach	451305	ENTEROSCOPIA (INTRAQUIRÚRGICA) TRANSABDOMINAL VÍA LAPAROSCÓPICA	Endoscopy	endoscopia_ diges
Stomach	451306	ENTEROSCOPIA O ENDOSCOPIA DE INTESTINO DELGADO DESPUÉS DE DUODENO CON BIOPSIA	Endoscopy	endoscopia_ diges
Stomach	451308	RESECCIÓN O ABLACIÓN DE LESIÓN DE INTESTINO DELGADO VÍA ENDOSCÓPICA	Endoscopy	endoscopia_ diges
Stomach	451309	DILATACIÓN DE INTESTINO DELGADO VÍA ENDOSCÓPICA	Endoscopy	endoscopia_ diges
Stomach	451310	MARCACIÓN DE LESIÓN EN INTESTINO DELGADO VÍA ENDOSCÓPICA	Endoscopy	endoscopia_ diges
Stomach	451401	ENTEROSCOPIA O ENDOSCOPIA DE INTESTINO DELGADO DESPUÉS DE DUODENO CON BIOPSIA	Endoscopy	endoscopia_ diges
Stomach	452101	ENDOSCOPIA TRANSABDOMINAL DE INTESTINO GRUESO (INTRAQUIRÚRGICA) VÍA ABIERTA	Endoscopy	endoscopia_ diges
Stomach	452102	ENDOSCOPIA TRANSABDOMINAL DE INTESTINO GRUESO (INTRAQUIRÚRGICA) VÍA LAPAROSCÓPICA	Endoscopy	endoscopia_ diges
Stomach	452200	ENDOSCOPIA DE INTESTINO GRUESO A TRAVES DE ESTOMA ARTIFICIAL SOD	Endoscopy	endoscopia_ diges
Stomach	452201	ENDOSCOPIA DEL INTESTINO GRUESO A TRAVÉS DE ESTOMA ARTIFICIAL	Endoscopy	endoscopia_ diges

Stomach	452304	MARCACIÓN DE LESIÓN EN COLON VÍA ENDOSCÓPICA	Endoscopy	endoscopia_ diges
Stomach	452502	BIOPSIA DE INTESTINO GRUESO ESCALONADA VÍA ENDOSCÓPICA (10 O MÁS)	Endoscopy	endoscopia_ diges
Stomach	542901	BIOPSIA POR PUNCION Y ASPIRACION GUIADA POR ECOENDOSCOPIA	Endoscopy	endoscopia_ diges
Stomach	881314	ECOGRAFÍA ENDOSCÓPICA DE ESTÓMAGO O DUODENO	Endoscopy	endoscopia_ diges
Stomach	425301	RECONSTRUCCION O ANASTOMOSIS ESOFAGICA CON INTERPOSICION DEL INTESTINO DELGADO VIA ABIERTA	Gastrectomy	gastrectomia
Stomach	438100	GASTRECTOMIA SUBTOTAL RADICAL SOD	Gastrectomy	gastrectomia
Stomach	438101	GASTRECTOMÍA SUBTOTAL RADICAL VÍA ABIERTA	Gastrectomy	gastrectomia
Stomach	438102	GASTRECTOMÍA SUBTOTAL RADICAL VÍA LAPAROSCÓPICA	Gastrectomy	gastrectomia
Stomach	438200	GASTRECTOMIA PARCIAL, CON RECONSTRUCCION CON O SIN VAGOTOMIA SOD	Gastrectomy	gastrectomia
Stomach	438201	GASTRECTOMÍA PARCIAL CON RECONSTRUCCIÓN CON VAGOTOMÍA VÍA ABIERTA	Gastrectomy	gastrectomia
Stomach	438202	GASTRECTOMÍA PARCIAL CON RECONSTRUCCIÓN CON VAGOTOMÍA VÍA LAPAROSCÓPICA	Gastrectomy	gastrectomia
Stomach	438203	GASTRECTOMÍA PARCIAL CON RECONSTRUCCIÓN SIN VAGOTOMÍA VÍA ABIERTA	Gastrectomy	gastrectomia
Stomach	438204	GASTRECTOMÍA PARCIAL CON RECONSTRUCCIÓN SIN VAGOTOMÍA VÍA LAPAROSCÓPICA	Gastrectomy	gastrectomia
Stomach	439001	GASTRECTOMÍA TOTAL VÍA ABIERTA	Gastrectomy	gastrectomia
Stomach	439002	GASTRECTOMÍA TOTAL VÍA LAPAROSCÓPICA	Gastrectomy	gastrectomia
Stomach	439003	GASTRECTOMÍA TOTAL RADICAL VÍA ABIERTA	Gastrectomy	gastrectomia
Stomach	439004	GASTRECTOMÍA TOTAL RADICAL VÍA LAPAROSCÓPICA	Gastrectomy	gastrectomia
Stomach	439100	GASTRECTOMIA TOTAL CON INTERPOSICION INTESTINAL SOD	Gastrectomy	gastrectomia
Stomach	439101	RECONSTRUCCIÓN GÁSTRICA CON INTERPOSICIÓN INTESTINAL VÍA ABIERTA	Gastrectomy	gastrectomia
Stomach	439102	RECONSTRUCCIÓN GÁSTRICA CON INTERPOSICIÓN INTESTINAL VÍA LAPAROSCÓPICA	Gastrectomy	gastrectomia
Stomach	439201	RECONSTRUCCIÓN GASTROINTESTINAL EN Y DE ROUX VÍA ABIERTA	Gastrectomy	gastrectomia
Stomach	439202	RECONSTRUCCIÓN GASTROINTESTINAL EN Y DE ROUX VÍA LAPAROSCÓPICA	Gastrectomy	gastrectomia
Stomach	872002	RADIOGRAFÍA DE ABDOMEN SIMPLE	Diagnostic imaging	imag_diagno s
Stomach	872011	RADIOGRAFÍA DE ABDOMEN SIMPLE CON PROYECCIONES ADICIONALES (SERIE DE ABDOMEN AGUDO)	Diagnostic imaging	imag_diagno s
Stomach	872121	RADIOGRAFÍA DE VÍAS DIGESTIVAS ALTAS (ESÓFAGO, ESTÓMAGO Y DUODENO)	Diagnostic imaging	imag_diagno s
Stomach	872122	RADIOGRAFÍA DE VÍAS DIGESTIVAS ALTAS (ESÓFAGO, ESTÓMAGO Y DUODENO) CON DOBLE CONTRASTE	Diagnostic imaging	imag_diagno s
Stomach	872123	RADIOGRAFÍA DE VÍAS DIGESTIVAS ALTAS (ESÓFAGO, ESTÓMAGO Y DUODENO) Y TRÁNSITO INTESTINAL	Diagnostic imaging	imag_diagno s
Stomach	879301	TOMOGRAFIA COMPUTADA DE TORAX	Diagnostic imaging	imag_diagno s
Stomach	879410	TOMOGRAFÍA COMPUTADA DE ABDOMEN SUPERIOR	Diagnostic imaging	imag_diagno s
Stomach	879420	TOMOGRAFIA COMPUTADA DE ABDOMEN Y PELVIS (ABDOMEN TOTAL)	Diagnostic imaging	imag_diagno s
Stomach	879601	TOMOGRAFIA POR EMISION DE POSITRONES [PET-TC]	Diagnostic imaging	imag_diagno s
Stomach	881301	ECOGRAFÍA DE TEJIDOS BLANDOS DE PARED ABDOMINAL Y DE PELVIS	Diagnostic imaging	imag_diagno s
Stomach	881302	ECOGRAFÍA DE ABDOMEN TOTAL (HÍGADO, PÁNCREAS, VESÍCULA, VÍAS BILIARES, RIÑONES, BAZO, GRANDES VASOS, PELVIS Y FLANCOS)	Diagnostic imaging	imag_diagno s

Stomach	881305	ECOGRAFÍA DE ABDOMEN SUPERIOR (HÍGADO, PÁNCREAS, VÍAS BILIARES, RIÑONES, BAZO Y GRANDES VASOS)	Diagnostic imaging	imag_diagnos
Stomach	881313	ECOGRAFÍA DE ABDOMEN (PÍLORO)	Diagnostic imaging	imag_diagnos
Stomach	881321	ECOGRAFÍA LAPAROSCÓPICA DE ABDOMEN	Diagnostic imaging	imag_diagnos
Stomach	881340	ECOGRAFÍA DE ABDOMEN (MASAS ABDOMINALES Y DE RETROPERITONEO)	Diagnostic imaging	imag_diagnos
Stomach	881390	ECOGRAFÍA DEL ABDOMEN Y PELVIS COMO GUÍA DE PROCEDIMIENTO QUIRÚRGICO O INTERVENCIONISTA	Diagnostic imaging	imag_diagnos
Stomach	883401	RESONANCIA MAGNETICA DE ABDOMEN	Diagnostic imaging	imag_diagnos
Stomach	883440	RESONANCIA MAGNETICA DE PELVIS	Diagnostic imaging	imag_diagnos
Stomach	890602	CUIDADO (MANEJO) INTRAHOSPITALARIO POR MEDICINA ESPECIALIZADA	Inpatient services	internacion
Stomach	107M01	INTERNACIÓN EN UNIDAD DE CUIDADO INTERMEDIO ADULTO	Inpatient services	internacion
Stomach	10A001	INTERNACIÓN COMPLEJIDAD ALTA HABITACION UNIPERSONAL (INCLUYE AISLAMIENTO)	Inpatient services	internacion
Stomach	10A002	INTERNACIÓN COMPLEJIDAD ALTA HABITACION BIPERSONAL	Inpatient services	internacion
Stomach	10A003	INTERNACIÓN COMPLEJIDAD ALTA TRES CAMAS	Inpatient services	internacion
Stomach	10A004	INTERNACIÓN COMPLEJIDAD ALTA CUATRO O MÁS CAMAS	Inpatient services	internacion
Stomach	10A005	INTERNACIÓN EN UNIDAD DE TRASPLANTE	Inpatient services	internacion
Stomach	10M001	INTERNACIÓN COMPLEJIDAD MEDIANA HABITACION UNIPERSONAL (INCLUYE AISLAMIENTO)	Inpatient services	internacion
Stomach	10M002	INTERNACIÓN COMPLEJIDAD MEDIANA HABITACION BIPERSONAL	Inpatient services	internacion
Stomach	10M003	INTERNACIÓN COMPLEJIDAD MEDIANA HABITACION TRES CAMAS	Inpatient services	internacion
Stomach	10M004	INTERNACIÓN COMPLEJIDAD MEDIANA HABITACION CUATRO O MÁS CAMAS	Inpatient services	internacion
Stomach	110A01	INTERNACIÓN EN UNIDAD DE CUIDADO INTENSIVO ADULTOS	Inpatient services	internacion
Stomach	121M01	INTERNACIÓN DE PACIENTE CRÓNICO TERMINAL SIN VENTILADOR COMPLEJIDAD MEDIANA	Inpatient services	internacion
Stomach	121M02	INTERNACIÓN DE PACIENTE CRÓNICO TERMINAL CON VENTILADOR COMPLEJIDAD MEDIANA	Inpatient services	internacion
Stomach	S11201	INTERNACIÓN COMPLEJIDAD MEDIANA HABITACION UNIPERSONAL (INCLUYE AISLAMIENTO)	Inpatient services	internacion
Stomach	S11202	INTERNACIÓN COMPLEJIDAD MEDIANA HABITACION BIPERSONAL	Inpatient services	internacion
Stomach	S11203	INTERNACIÓN COMPLEJIDAD MEDIANA HABITACION TRES CAMAS	Inpatient services	internacion
Stomach	S11204	INTERNACIÓN COMPLEJIDAD MEDIANA HABITACION CUATRO O MÁS CAMAS	Inpatient services	internacion
Stomach	S11301	INTERNACIÓN COMPLEJIDAD ALTA HABITACION UNIPERSONAL (INCLUYE AISLAMIENTO)	Inpatient services	internacion
Stomach	S11302	INTERNACIÓN COMPLEJIDAD ALTA HABITACION BIPERSONAL	Inpatient services	internacion
Stomach	S11303	INTERNACIÓN COMPLEJIDAD ALTA TRES CAMAS	Inpatient services	internacion
Stomach	S11304	INTERNACIÓN COMPLEJIDAD ALTA CUATRO O MÁS CAMAS	Inpatient services	internacion
Stomach	S12103	INTERNACIÓN EN UNIDAD DE CUIDADO INTENSIVO ADULTOS	Inpatient services	internacion
Stomach	S12203	INTERNACIÓN EN UNIDAD DE CUIDADO INTERMEDIO ADULTO	Inpatient services	internacion
Stomach	S12600	INTERNACIÓN EN UNIDAD DE TRASPLANTE	Inpatient services	internacion
Stomach	898101	ESTUDIO DE COLORACION BASICA EN BIOPSIA	Laboratory tests	laboratorio
Stomach	898102	ESTUDIO DE COLORACION HISTOQUIMICA EN BIOPSIA	Laboratory tests	laboratorio
Stomach	898103	ESTUDIO DE COLORACION INMUNOHISTOQUIMICA EN BIOPSIA	Laboratory tests	laboratorio
Stomach	405101	VACIAMIENTO RADICAL LINFÁTICO AXILAR VÍA ABIERTA	Lymphadenectomy	linfadenectomia
Stomach	405201	VACIAMIENTO RADICAL LINFÁTICO (LINFADENECTOMÍA) DE MEDIASTINO VÍA ABIERTA	Lymphadenectomy	linfadenectomia
Stomach	405202	VACIAMIENTO RADICAL LINFÁTICO (LINFADENECTOMÍA) DE MEDIASTINO VÍA TORACOSCÓPICA	Lymphadenectomy	linfadenectomia

Stomach	405202	VACIAMIENTO RADICAL LINFÁTICO (LINFADENECTOMÍA) DE MEDIASTINO VÍA TORACOSCÓPICA	Lymphadenectomy	linfadenecto mia
Stomach	405203	VACIAMIENTO RADICAL LINFÁTICO (LINFADENECTOMÍA) DE MEDIASTINO POR MEDIASTINOSCOPIA	Lymphadenectomy	linfadenecto mia
Stomach	405301	LINFADENECTOMIA RADICAL INGUINOFEMORAL, UNILATERAL	Lymphadenectomy	linfadenecto mia
Stomach	405302	LINFADENECTOMIA RADICAL INGUINOFEMORAL O ILIACA BILATERAL	Lymphadenectomy	linfadenecto mia
Stomach	405304	LINFADENECTOMÍA RADICAL INGUINOFEMORAL, UNILATERAL VÍA ABIERTA	Lymphadenectomy	linfadenecto mia
Stomach	405305	LINFADENECTOMÍA RADICAL INGUINOFEMORAL, UNILATERAL VÍA LAPAROSCÓPICA	Lymphadenectomy	linfadenecto mia
Stomach	405306	LINFADENECTOMÍA RADICAL INGUINOILÍACO BILATERAL VÍA ABIERTA	Lymphadenectomy	linfadenecto mia
Stomach	405307	LINFADENECTOMÍA RADICAL INGUINOILÍACO BILATERAL VÍA LAPAROSCÓPICA	Lymphadenectomy	linfadenecto mia
Stomach	405401	LINFADENECTOMIA RADICAL PELVICA	Lymphadenectomy	linfadenecto mia
Stomach	405402	LINFADENECTOMIA RADICAL EXTRAPERITONEAL	Lymphadenectomy	linfadenecto mia
Stomach	405404	LINFADENECTOMÍA RADICAL ABDOMINAL VÍA ABIERTA	Lymphadenectomy	linfadenecto mia
Stomach	405405	LINFADENECTOMÍA RADICAL ABDOMINAL VÍA LAPAROSCÓPICA	Lymphadenectomy	linfadenecto mia
Stomach	405406	LINFADENECTOMÍA RADICAL PÉLVICA VÍA ABIERTA	Lymphadenectomy	linfadenecto mia
Stomach	405407	LINFADENECTOMÍA RADICAL PÉLVICA VÍA LAPAROSCÓPICA	Lymphadenectomy	linfadenecto mia
Stomach	405408	LINFADENECTOMÍA RADICAL EXTRAPERITONEAL VÍA ABIERTA	Lymphadenectomy	linfadenecto mia
Stomach	405409	LINFADENECTOMÍA RADICAL EXTRAPERITONEAL VÍA LAPAROSCÓPICA	Lymphadenectomy	linfadenecto mia
Stomach	405411	LINFADENECTOMÍA RADICAL ABDOMINO INGUINAL VÍA ABIERTA	Lymphadenectomy	linfadenecto mia
Stomach	405412	LINFADENECTOMÍA RADICAL ABDOMINO INGUINAL VÍA LAPAROSCÓPICA	Lymphadenectomy	linfadenecto mia
Stomach	405501	RESECCION RADICAL DE GANGLIOS LINFATICOS RETROPERITONEALES	Lymphadenectomy	linfadenecto mia
Stomach	405502	RESECCIÓN RADICAL DE GANGLIOS LINFÁTICOS RETROPERITONEALES VÍA ABIERTA	Lymphadenectomy	linfadenecto mia
Stomach	405503	LINFADENECTOMÍA RETROPERITONEAL VÍA LAPAROSCÓPICA	Lymphadenectomy	linfadenecto mia
Stomach	405601	VACIAMIENTO RADICAL EPITROCLEAR VÍA ABIERTA	Lymphadenectomy	linfadenecto mia
Stomach	405602	VACIAMIENTO RADICAL POPLITEO VÍA ABIERTA	Lymphadenectomy	linfadenecto mia
Stomach	992503	MONOTERAPIA ANTINEOPLÁSICA DE BAJA TOXICIDAD	Monoterapy	monoterapia
Stomach	992504	POLITERAPIA ANTINEOPLÁSICA DE BAJA TOXICIDAD	Polyterapy	politerapia
Stomach	992505	POLITERAPIA ANTINEOPLÁSICA DE ALTA TOXICIDAD	Polyterapy	politerapia

Stomach	992506	ADMINISTRACIÓN (INFUSIÓN O PERFUSIÓN) DE TERAPIA ANTINEOPLÁSICA INTRARTERIAL (REGIONAL)	Monoterapy	monoterapia
Stomach	992509	MONOTERAPIA ANTINEOPLÁSICA DE BAJA TOXICIDAD	Monoterapy	monoterapia
Stomach	992511	MONOTERAPIA ANTINEOPLÁSICA DE ALTA TOXICIDAD	Monoterapy	monoterapia
Stomach	922201	TELETERAPIA CON ORTOVOLTAJE	Teletherapy	teleterapia
Stomach	922321	TELETERAPIA CON COBALTO (PLANEACIÓN COMPUTARIZADA BIDIMENSIONAL Y SIMULACIÓN CONVENCIONAL)	Teletherapy	teleterapia
Stomach	922322	TELETERAPIA CON COBALTO (PLANEACIÓN COMPUTARIZADA TRIDIMENSIONAL Y SIMULACIÓN VIRTUAL)	Teletherapy	teleterapia
Stomach	922441	TELETERAPIA CON ACELERADOR LINEAL (PLANEACIÓN COMPUTARIZADA BIDIMENSIONAL Y SIMULACIÓN CONVENCIONAL) TÉCNICA RADIOTERAPIA CONVENCIONAL	Teletherapy	teleterapia
Stomach	922442	TELETERAPIA CON ACELERADOR LINEAL (PLANEACIÓN COMPUTARIZADA TRIDIMENSIONAL Y SIMULACIÓN VIRTUAL) TÉCNICA RADIOTERAPIA CONVENCIONAL	Teletherapy	teleterapia
Stomach	922443	TELETERAPIA CON ACELERADOR LINEAL (PLANEACION COMPUTARIZADA TRIDIMENSIONAL Y SIMULACION VIRTUAL) TECNICA CONFORMACIONAL [3D - CRT]	Teletherapy	teleterapia
Stomach	922444	TELETERAPIA CON ACELERADOR LINEAL (PLANEACIÓN COMPUTARIZADA TRIDIMENSIONAL Y SIMULACIÓN VIRTUAL) TÉCNICA RADIOTERAPIA DE INTENSIDAD MODULADA [IMRT]	Teletherapy	teleterapia
Stomach	922445	TELETERAPIA CON ACELERADOR LINEAL (PLANEACIÓN COMPUTARIZADA TRIDIMENSIONAL Y SIMULACIÓN VIRTUAL) TÉCNICA RADIOTERAPIA GUIADA POR IMÁGENES [IGRT]	Teletherapy	teleterapia
Stomach	922446	TELETERAPIA CON ACELERADOR LINEAL (PLANEACIÓN COMPUTARIZADA TRIDIMENSIONAL Y SIMULACIÓN VIRTUAL) TÉCNICA RADIOTERAPIA - ARCOTERAPIA DE MODULACIÓN VOLUMÉTRICA [VMAT]	Teletherapy	teleterapia
Stomach	922447	IRRADIACIÓN CORPORAL TOTAL	Irradiation	irradiacion
Stomach	922448	IRRADIACIÓN CUTÁNEA TOTAL	Irradiation	irradiacion
Stomach	922449	TELETERAPIA CON ACELERADOR LINEAL (PLANEACIÓN COMPUTARIZADA TRIDIMENSIONAL Y SIMULACIÓN VIRTUAL) TÉCNICA RADIOTERAPIA HELICOIDAL	Teletherapy	teleterapia
Stomach	922504	TELETERAPIA CON ACELERADOR LINEAL DE ELECTRONES (PLANEACION COMPUTARIZADA BIDIMENSIONAL Y SIMULACION CONVENCIONAL)	Teletherapy	teleterapia
Stomach	922505	TELETERAPIA CON ACELERADOR LINEAL DE ELECTRONES (PLANEACION COMPUTARIZADA TRIDIMENSIONAL Y SIMULACIÓN VIRTUAL)	Teletherapy	teleterapia
Stomach	922506	RADIOTERAPIA INTRAOPERATORIA	Teletherapy	teleterapia
Stomach	922601	BRAQUITERAPIA INTERSTICIAL (PLANEACION COMPUTARIZADA TRIDIMENSIONAL Y SIMULACION VIRTUAL) CON ALTA TASA DE DOSIS	Brachytherapy	braquiterapi a
Stomach	922602	BRAQUITERAPIA INTERSTICIAL (PLANEACION COMPUTARIZADA TRIDIMENSIONAL Y SIMULACIÓN VIRTUAL) CON BAJA TASA DE DOSIS	Brachytherapy	braquiterapi a
Stomach	922603	BRAQUITERAPIA INTRALUMINAL (PLANEACION COMPUTARIZADA BIDIMENSIONAL Y SIMULACION CONVENCIONAL) CON ALTA TASA DE DOSIS	Brachytherapy	braquiterapi a
Stomach	922604	BRAQUITERAPIA INTRALUMINAL CON BAJA TASA DE DOSIS	Brachytherapy	braquiterapi a
Stomach	922605	BRAQUITERAPIA INTRACAVITARIA (PLANEACION COMPUTARIZADA BIDIMENSIONAL Y SIMULACION CONVENCIONAL) CON ALTA TASA DE DOSIS	Brachytherapy	braquiterapi a
Stomach	922606	BRAQUITERAPIA INTRACAVITARIA (PLANEACION COMPUTARIZADA BIDIMENSIONAL Y SIMULACION CONVENCIONAL) CON BAJA TASA DE DOSIS	Brachytherapy	braquiterapi a
Stomach	922607	BRAQUITERAPIA INTRACAVITARIA (PLANEACION COMPUTARIZADA TRIDIMENSIONAL Y SIMULACION VIRTUAL) CON ALTA TASA DE DOSIS	Brachytherapy	braquiterapi a
Stomach	922608	BRAQUITERAPIA INTRACAVITARIA (PLANEACIÓN COMPUTARIZADA TRIDIMENSIONAL Y SIMULACIÓN VIRTUAL) CON BAJA TASA DE DOSIS	Brachytherapy	braquiterapi a
Stomach	922609	BRAQUITERAPIA INTRALUMINAL (PLANEACIÓN COMPUTARIZADA TRIDIMENSIONAL Y SIMULACIÓN VIRTUAL) CON ALTA TASA DE DOSIS	Brachytherapy	braquiterapi a
Stomach	922611	BRAQUITERAPIA DE CONTACTO (PLANEACION COMPUTARIZADA BIDIMENSIONAL Y SIMULACION CONVENCIONAL) CON ALTA TASA DE DOSIS	Brachytherapy	braquiterapi a
Stomach	922612	BRAQUITERAPIA DE CONTACTO (PLANEACIÓN COMPUTARIZADA TRIDIMENSIONAL Y SIMULACIÓN VIRTUAL) CON ALTA TASA DE DOSIS	Brachytherapy	braquiterapi a

Stomach	922613	BRAQUITERAPIA EPIESCLERAL DE CONTACTO (PLANEACIÓN COMPUTARIZADA TRIDIMENSIONAL Y SIMULACIÓN VIRTUAL) CON BAJA TASA DE DOSIS	Brachytherapy	braquiterapi a
Stomach	922614	BRAQUITERAPIA METABÓLICA	Brachytherapy	braquiterapi a
Stomach	922615	BRAQUITERAPIA INTERSTICIAL (PLANEACIÓN COMPUTARIZADA BIDIMENSIONAL Y SIMULACIÓN CONVENCIONAL) CON ALTA TASA DE DOSIS	Brachytherapy	braquiterapi a
Stomach	922616	BRAQUITERAPIA INTERSTICIAL (PLANEACIÓN COMPUTARIZADA TRIDIMENSIONAL Y SIMULACIÓN VIRTUAL) CON ALTA TASA DE DOSIS	Brachytherapy	braquiterapi a
Stomach	922800	TERAPIA CON RADIOISOTOPOS SOD	Radiotherapy	terapia
Stomach	922801	TERAPIA CON RADIOISÓTOPOS	Radiotherapy	terapia
Stomach	922805	TERAPIA CON ITRIUM 90	Radiotherapy	terapia
Stomach	922810	TERAPIA CON METAIODOBENCILGUANIDINA SUPRARRENAL	Radiotherapy	terapia
Stomach	922830	TERAPIA DE METÁSTASIS CON ESTRONCIO	Radiotherapy	terapia

Table A2.2. Drugs considered for the analysis

Cancer	ATC	Descripcion ATC	CUM
Breast	L01XE50	ABEMACICLIB	20187782-3
Breast	L01XE50	ABEMACICLIB	20187782-2
Breast	L01XE50	ABEMACICLIB	20187782-1
Breast	L01XE50	ABEMACICLIB	20187776-6
Breast	L01XE50	ABEMACICLIB	20187776-3
Breast	L01XE50	ABEMACICLIB	20187776-5
Breast	L01XE50	ABEMACICLIB	20187776-4
Breast	L01XE50	ABEMACICLIB	20187776-1
Breast	L01XE50	ABEMACICLIB	20188001-1
Breast	L01XE50	ABEMACICLIB	20188001-2
Breast	L01XE50	ABEMACICLIB	20188001-3
Breast	L01XE50	ABEMACICLIB	20188001-4
Breast	L01XE50	ABEMACICLIB	20187776-2
Breast	L01XE50	ABEMACICLIB	20187782-4
Breast	L02BG03	ANASTROZOL	20086962-2
Breast	L02BG03	ANASTROZOL	20092750-1
Breast	L02BG03	ANASTROZOL	20008399-5
Breast	L02BG03	ANASTROZOL	20008399-4
Breast	L02BG03	ANASTROZOL	20008399-3
Breast	L02BG03	ANASTROZOL	20008399-2
Breast	L02BG03	ANASTROZOL	20008399-1
Breast	L02BG03	ANASTROZOL	19993886-4
Breast	L02BG03	ANASTROZOL	19993886-3
Breast	L02BG03	ANASTROZOL	19993886-2
Breast	L02BG03	ANASTROZOL	19993886-1
Breast	L02BG03	ANASTROZOL	20047794-1
Breast	L02BG03	ANASTROZOL	20057635-1
Breast	L02BG03	ANASTROZOL	20047794-3
Breast	L02BG03	ANASTROZOL	20135463-4

Breast	L02BG03	ANASTROZOL	20135463-3
Breast	L02BG03	ANASTROZOL	206742-1
Breast	L02BG03	ANASTROZOL	206742-2
Breast	L02BG03	ANASTROZOL	206742-3
Breast	L02BG03	ANASTROZOL	20135463-2
Breast	L02BG03	ANASTROZOL	20135463-1
Breast	L02BG03	ANASTROZOL	20047794-2
Breast	L02BG03	ANASTROZOL	20135463-5
Breast	L02BG03	ANASTROZOL	20038017-2
Breast	L02BG03	ANASTROZOL	20038017-1
Breast	L02BG03	ANASTROZOL	20086962-1
Breast	L02BG03	ANASTROZOL	19910957-1
Breast	L02BG03	ANASTROZOL	19910957-2
Breast	L02BG03	ANASTROZOL	19910957-3
Breast	L02BG03	ANASTROZOL	19910957-4
Breast	L02BG03	ANASTROZOL	19910957-6
Breast	L02BG03	ANASTROZOL	19910957-5
Breast	L02BG03	ANASTROZOL	19910957-7
Breast	L01XC32	ATEZOLIZUMAB	20145962-2
Breast	L01XC32	ATEZOLIZUMAB	20145962-1
Breast	L01XC07	BEVACIZUMAB	20169679-3
Breast	L01XC07	BEVACIZUMAB	20169679-4
Breast	L01XC07	BEVACIZUMAB	20179635-1
Breast	L01XC07	BEVACIZUMAB	20169679-1
Breast	L01XC07	BEVACIZUMAB	20149223-4
Breast	L01XC07	BEVACIZUMAB	20195592-4
Breast	L01XC07	BEVACIZUMAB	20195592-1
Breast	L01XC07	BEVACIZUMAB	20149223-1
Breast	L01XC07	BEVACIZUMAB	20169679-2
Breast	L01XC07	BEVACIZUMAB	20149223-3
Breast	L01XC07	BEVACIZUMAB	20149223-2
Breast	L01XC07	BEVACIZUMAB	20195592-2
Breast	L01XC07	BEVACIZUMAB	20195592-3
Breast	L01BC06	CAPECITABINA	20155908-1
Breast	L01BC06	CAPECITABINA	20152744-1
Breast	L01BC06	CAPECITABINA	20146928-1
Breast	L01BC06	CAPECITABINA	20132906-4
Breast	L01BC06	CAPECITABINA	20132906-3
Breast	L01BC06	CAPECITABINA	20132906-2
Breast	L01BC06	CAPECITABINA	20132906-1
Breast	L01BC06	CAPECITABINA	20132905-5
Breast	L01BC06	CAPECITABINA	20132905-4
Breast	L01BC06	CAPECITABINA	20132905-3
Breast	L01BC06	CAPECITABINA	20132905-2
Breast	L01BC06	CAPECITABINA	20132905-1
Breast	L01BC06	CAPECITABINA	20116817-9
Breast	L01BC06	CAPECITABINA	20116817-8
Breast	L01BC06	CAPECITABINA	20116817-7

Breast	L01BC06	CAPECITABINA	20116817-6
Breast	L01BC06	CAPECITABINA	20116817-5
Breast	L01BC06	CAPECITABINA	20116817-4
Breast	L01BC06	CAPECITABINA	20116817-3
Breast	L01BC06	CAPECITABINA	20116817-2
Breast	L01BC06	CAPECITABINA	20116817-10
Breast	L01BC06	CAPECITABINA	20116817-1
Breast	L01BC06	CAPECITABINA	20111735-1
Breast	L01BC06	CAPECITABINA	20104404-1
Breast	L01BC06	CAPECITABINA	20060323-9
Breast	L01BC06	CAPECITABINA	20060323-8
Breast	L01BC06	CAPECITABINA	20060323-7
Breast	L01BC06	CAPECITABINA	20060323-6
Breast	L01BC06	CAPECITABINA	20060323-5
Breast	L01BC06	CAPECITABINA	20060323-4
Breast	L01BC06	CAPECITABINA	20060323-3
Breast	L01BC06	CAPECITABINA	20060323-2
Breast	L01BC06	CAPECITABINA	20060323-15
Breast	L01BC06	CAPECITABINA	20060323-14
Breast	L01BC06	CAPECITABINA	20060323-13
Breast	L01BC06	CAPECITABINA	20053629-1
Breast	L01BC06	CAPECITABINA	20060323-1
Breast	L01BC06	CAPECITABINA	20060323-10
Breast	L01BC06	CAPECITABINA	20060323-11
Breast	L01BC06	CAPECITABINA	20060323-12
Breast	L01BC06	CAPECITABINA	229745-1
Breast	L01BC06	CAPECITABINA	20160260-4
Breast	L01BC06	CAPECITABINA	20160260-3
Breast	L01BC06	CAPECITABINA	20160260-2
Breast	L01BC06	CAPECITABINA	20160260-1
Breast	L01BC06	CAPECITABINA	20155908-9
Breast	L01BC06	CAPECITABINA	20155908-8
Breast	L01BC06	CAPECITABINA	20155908-7
Breast	L01BC06	CAPECITABINA	20155908-6
Breast	L01BC06	CAPECITABINA	20155908-5
Breast	L01BC06	CAPECITABINA	20155908-4
Breast	L01BC06	CAPECITABINA	20155908-3
Breast	L01BC06	CAPECITABINA	20155908-2
Breast	L01AA01	CICLOFOSFAMIDA	20014778-9
Breast	L01AA01	CICLOFOSFAMIDA	21531-1
Breast	L01AA01	CICLOFOSFAMIDA	20103436-1
Breast	L01AA01	CICLOFOSFAMIDA	20121471-1
Breast	L01AA01	CICLOFOSFAMIDA	20090481-1
Breast	L01AA01	CICLOFOSFAMIDA	20014778-1
Breast	L01AA01	CICLOFOSFAMIDA	20014778-10
Breast	L01AA01	CICLOFOSFAMIDA	20014778-2
Breast	L01AA01	CICLOFOSFAMIDA	20014778-3
Breast	L01AA01	CICLOFOSFAMIDA	20014778-4

Breast	L01AA01	CICLOFOSFAMIDA	20014778-5
Breast	L01AA01	CICLOFOSFAMIDA	20014778-6
Breast	L01AA01	CICLOFOSFAMIDA	20014778-7
Breast	L01AA01	CICLOFOSFAMIDA	20014778-8
Breast	L01AA01	CICLOFOSFAMIDA	19935953-1
Breast	L01AA01	CICLOFOSFAMIDA	21531-2
Breast	L01AA01	CICLOFOSFAMIDA	21534-1
Breast	L01AA01	CICLOFOSFAMIDA	20014777-1
Breast	L01AA01	CICLOFOSFAMIDA	19935953-2
Breast	L01AA01	CICLOFOSFAMIDA	21535-1
Breast	L01AA01	CICLOFOSFAMIDA	19961041-1
Breast	L01AA01	CICLOFOSFAMIDA	19957078-1
Breast	L01AA01	CICLOFOSFAMIDA	20016861-2
Breast	L01AA01	CICLOFOSFAMIDA	20016861-1
Breast	L01XA01	CISPLATINO	20006179-1
Breast	L01XA01	CISPLATINO	19982072-1
Breast	L01XA01	CISPLATINO	19957108-1
Breast	L01XA01	CISPLATINO	20035123-1
Breast	L01XA01	CISPLATINO	20141904-1
Breast	L01XA01	CISPLATINO	19992871-1
Breast	L01XA01	CISPLATINO	19992872-1
Breast	L01XA01	CISPLATINO	19992872-2
Breast	L01XA01	CISPLATINO	44823-1
Breast	L01XA01	CISPLATINO	20095210-1
Breast	L01XA01	CISPLATINO	19976715-1
Breast	L01XA01	CISPLATINO	20035122-1
Breast	L01XA01	CISPLATINO	19964252-1
Breast	L01XA01	CISPLATINO	19963386-1
Breast	L01XA01	CISPLATINO	20142068-1
Breast	L01XA01	CISPLATINO	19969323-1
Breast	L01XA01	CISPLATINO	19947842-1
Breast	L01XA01	CISPLATINO	20095072-1
Breast	L01XA01	CISPLATINO	19913980-1
Breast	L01XA01	CISPLATINO	19963386-3
Breast	L01XA01	CISPLATINO	20013777-1
Breast	L01XA01	CISPLATINO	20013777-2
Breast	L01XA01	CISPLATINO	20047565-1
Breast	L01XA01	CISPLATINO	20047564-1
Breast	L01XA01	CISPLATINO	20013777-3
Breast	L01XA01	CISPLATINO	19963386-4
Breast	L01XA01	CISPLATINO	19963386-2
Breast	L01XA01	CISPLATINO	44821-1
Breast	L01XA01	CISPLATINO	20095072-2
Breast	L01BC08	DECITABINA	19991325-1
Breast	L01BC08	DECITABINA	19991325-2
Breast	L01BC08	DECITABINA	20097119-2
Breast	L01BC08	DECITABINA	20097119-1
Breast	L01BC08	DECITABINA	20096772-1

Breast	L01BC08	DECITABINA	20148281-1
Breast	L01BC08	DECITABINA	20192584-1
Breast	L01BC08	DECITABINA	20114014-1
Breast	L01CD02	DOCETAXEL	20045960-1
Breast	L01CD02	DOCETAXEL	19969842-1
Breast	L01CD02	DOCETAXEL	19969842-2
Breast	L01CD02	DOCETAXEL	19969842-3
Breast	L01CD02	DOCETAXEL	19969253-1
Breast	L01CD02	DOCETAXEL	19969253-2
Breast	L01CD02	DOCETAXEL	19969253-3
Breast	L01CD02	DOCETAXEL	19969253-4
Breast	L01CD02	DOCETAXEL	19969253-5
Breast	L01CD02	DOCETAXEL	20057737-1
Breast	L01CD02	DOCETAXEL	20064412-1
Breast	L01CD02	DOCETAXEL	20064412-2
Breast	L01CD02	DOCETAXEL	20028651-3
Breast	L01CD02	DOCETAXEL	20028651-2
Breast	L01CD02	DOCETAXEL	20028651-1
Breast	L01CD02	DOCETAXEL	20024849-3
Breast	L01CD02	DOCETAXEL	20024849-2
Breast	L01CD02	DOCETAXEL	20024849-1
Breast	L01CD02	DOCETAXEL	20022245-3
Breast	L01CD02	DOCETAXEL	20022245-2
Breast	L01CD02	DOCETAXEL	20022245-1
Breast	L01CD02	DOCETAXEL	20064412-3
Breast	L01CD02	DOCETAXEL	20064460-1
Breast	L01CD02	DOCETAXEL	20064460-2
Breast	L01CD02	DOCETAXEL	20064460-3
Breast	L01CD02	DOCETAXEL	20020736-3
Breast	L01CD02	DOCETAXEL	20020736-2
Breast	L01CD02	DOCETAXEL	20020736-1
Breast	L01CD02	DOCETAXEL	20064497-1
Breast	L01CD02	DOCETAXEL	20064497-2
Breast	L01CD02	DOCETAXEL	20064497-3
Breast	L01CD02	DOCETAXEL	20064497-4
Breast	L01CD02	DOCETAXEL	20064497-5
Breast	L01CD02	DOCETAXEL	20064499-1
Breast	L01CD02	DOCETAXEL	20045955-1
Breast	L01CD02	DOCETAXEL	19974847-1
Breast	L01CD02	DOCETAXEL	20067008-1
Breast	L01CD02	DOCETAXEL	20047646-1
Breast	L01CD02	DOCETAXEL	20111936-2
Breast	L01CD02	DOCETAXEL	20113688-1
Breast	L01CD02	DOCETAXEL	20111936-1
Breast	L01CD02	DOCETAXEL	20113692-1
Breast	L01CD02	DOCETAXEL	20111935-2
Breast	L01CD02	DOCETAXEL	20111935-1
Breast	L01CD02	DOCETAXEL	20047644-1

Breast	L01CD02	DOCETAXEL	230187-1
Breast	L01CD02	DOCETAXEL	230189-2
Breast	L01CD02	DOCETAXEL	19996764-1
Breast	L01CD02	DOCETAXEL	19996765-1
Breast	L01CD02	DOCETAXEL	20007286-1
Breast	L01CD02	DOCETAXEL	20049388-1
Breast	L01CD02	DOCETAXEL	20057736-1
Breast	L01DB01	DOXORUBICINA	58178-1
Breast	L01DB01	DOXORUBICINA	19947745-2
Breast	L01DB01	DOXORUBICINA	1980838-2
Breast	L01DB01	DOXORUBICINA	20061064-1
Breast	L01DB01	DOXORUBICINA	20061064-2
Breast	L01DB01	DOXORUBICINA	20061064-3
Breast	L01DB01	DOXORUBICINA	20062344-1
Breast	L01DB01	DOXORUBICINA	20134950-1
Breast	L01DB01	DOXORUBICINA	20043047-2
Breast	L01DB01	DOXORUBICINA	20043047-1
Breast	L01DB01	DOXORUBICINA	20043044-1
Breast	L01DB01	DOXORUBICINA	1980838-3
Breast	L01DB01	DOXORUBICINA	1980838-4
Breast	L01DB01	DOXORUBICINA	20037727-3
Breast	L01DB01	DOXORUBICINA	20037727-2
Breast	L01DB01	DOXORUBICINA	20037727-1
Breast	L01DB01	DOXORUBICINA	20057171-1
Breast	L01DB01	DOXORUBICINA	19971373-3
Breast	L01DB01	DOXORUBICINA	19971373-4
Breast	L01DB01	DOXORUBICINA	58174-1
Breast	L01DB01	DOXORUBICINA	1980838-6
Breast	L01DB01	DOXORUBICINA	20118394-1
Breast	L01DB01	DOXORUBICINA	20057168-1
Breast	L01DB01	DOXORUBICINA	19947745-1
Breast	L01DB01	DOXORUBICINA	20155365-2
Breast	L01DB01	DOXORUBICINA	20196716-1
Breast	L01DB01	DOXORUBICINA	20155365-1
Breast	L01DB01	DOXORUBICINA	20196716-2
Breast	L01DB01	DOXORUBICINA	19967936-1
Breast	L01DB01	DOXORUBICINA	19989735-1
Breast	L01DB01	DOXORUBICINA	19947745-3
Breast	L01DB01	DOXORUBICINA	19947745-4
Breast	L01DB01	DOXORUBICINA	20005641-1
Breast	L01DB01	DOXORUBICINA	19976713-1
Breast	L01DB01	DOXORUBICINA	19976712-1
Breast	L01DB01	DOXORUBICINA	19969115-1
Breast	L01DB01	DOXORUBICINA	1980838-5
Breast	L01DB01	DOXORUBICINA	19969115-3
Breast	L01DB01	DOXORUBICINA	38447-1
Breast	L01DB01	DOXORUBICINA	19947766-1
Breast	L01DB01	DOXORUBICINA	1980838-1

Breast	L01DB01	DOXORUBICINA	20010928-1
Breast	L01DB01	DOXORUBICINA	19953724-1
Breast	L01DB01	DOXORUBICINA	19953724-2
Breast	L01DB01	DOXORUBICINA	20091053-1
Breast	L01DB01	DOXORUBICINA	20011067-1
Breast	L01DB01	DOXORUBICINA	19953724-3
Breast	L01DB01	DOXORUBICINA	19971373-1
Breast	L01DB01	DOXORUBICINA	19971373-2
Breast	L01DB01	DOXORUBICINA	19969115-2
Breast	L01DB01	DOXORUBICINA	38446-1
Breast	L01DB03	EPIRRUBICINA	20134303-1
Breast	L01DB03	EPIRRUBICINA	19956731-1
Breast	L01DB03	EPIRRUBICINA	19974845-1
Breast	L01DB03	EPIRRUBICINA	20014047-1
Breast	L01DB03	EPIRRUBICINA	19973149-2
Breast	L01DB03	EPIRRUBICINA	19973149-1
Breast	L01DB03	EPIRRUBICINA	19926703-1
Breast	L01DB03	EPIRRUBICINA	19956731-2
Breast	L01DB03	EPIRRUBICINA	20063700-1
Breast	L01DB03	EPIRRUBICINA	20063700-2
Breast	L01DB03	EPIRRUBICINA	20063700-3
Breast	L01DB03	EPIRRUBICINA	19926703-3
Breast	L01DB03	EPIRRUBICINA	19926703-2
Breast	L01DB03	EPIRRUBICINA	20062983-1
Breast	L01DB03	EPIRRUBICINA	19973149-3
Breast	L01BC02	FLUOROURACILO	20071503-1
Breast	L01BC02	FLUOROURACILO	20071503-2
Breast	L01BC02	FLUOROURACILO	20121175-1
Breast	L01BC02	FLUOROURACILO	20121175-2
Breast	L01BC02	FLUOROURACILO	20125230-1
Breast	L01BC02	FLUOROURACILO	20125230-2
Breast	L01BC02	FLUOROURACILO	20133414-1
Breast	L01BC02	FLUOROURACILO	216264-1
Breast	L01BC02	FLUOROURACILO	216264-2
Breast	L01BC02	FLUOROURACILO	216264-3
Breast	L01BC02	FLUOROURACILO	227728-1
Breast	L01BC02	FLUOROURACILO	227728-2
Breast	L01BC02	FLUOROURACILO	19964018-8
Breast	L01BC02	FLUOROURACILO	19930999-1
Breast	L01BC02	FLUOROURACILO	19930999-2
Breast	L01BC02	FLUOROURACILO	19930999-3
Breast	L01BC02	FLUOROURACILO	19930999-4
Breast	L01BC02	FLUOROURACILO	19930999-5
Breast	L01BC02	FLUOROURACILO	19964018-1
Breast	L01BC02	FLUOROURACILO	19964018-2
Breast	L01BC02	FLUOROURACILO	19964018-3
Breast	L01BC02	FLUOROURACILO	19964018-4
Breast	L01BC02	FLUOROURACILO	19964018-5

Breast	L01BC02	FLUOROURACILO	19964018-6
Breast	L01BC02	FLUOROURACILO	19964018-7
Breast	L01BC02	FLUOROURACILO	20043050-1
Breast	L01BC02	FLUOROURACILO	20043050-2
Breast	L01BC02	FLUOROURACILO	20043050-3
Breast	L01BC02	FLUOROURACILO	20043050-4
Breast	L01BC02	FLUOROURACILO	20043050-5
Breast	L01BC02	FLUOROURACILO	20043050-6
Breast	L01BC02	FLUOROURACILO	20043050-7
Breast	L01BC02	FLUOROURACILO	20053621-1
Breast	L02BA03	FLUVESTRANT	20136464-1
Breast	L02BA03	FLUVESTRANT	20118041-1
Breast	L02BA03	FLUVESTRANT	19955642-1
Breast	L02BA03	FLUVESTRANT	19955642-2
Breast	L02BA03	FLUVESTRANT	20136464-2
Breast	L02BA03	FLUVESTRANT	20118041-2
Breast	L02BA03	FLUVESTRANT	20118041-3
Breast	L02BA03	FLUVESTRANT	19955642-3
Breast	L02BA03	FLUVESTRANT	20118041-4
Breast	L02BA03	FLUVESTRANT	20177494-3
Breast	L02BA03	FLUVESTRANT	20177494-2
Breast	L02BA03	FLUVESTRANT	20177494-1
Breast	L02BA03	FLUVESTRANT	20132157-1
Breast	L02BA03	FLUVESTRANT	19955642-4
Breast	L01BC05	GEMCITABINA	20078381-1
Breast	L01BC05	GEMCITABINA	19970173-2
Breast	L01BC05	GEMCITABINA	19970173-3
Breast	L01BC05	GEMCITABINA	19970173-4
Breast	L01BC05	GEMCITABINA	19970173-5
Breast	L01BC05	GEMCITABINA	19970173-6
Breast	L01BC05	GEMCITABINA	19970173-7
Breast	L01BC05	GEMCITABINA	19970173-8
Breast	L01BC05	GEMCITABINA	19978376-1
Breast	L01BC05	GEMCITABINA	19997219-1
Breast	L01BC05	GEMCITABINA	19997220-1
Breast	L01BC05	GEMCITABINA	20010932-1
Breast	L01BC05	GEMCITABINA	20010934-1
Breast	L01BC05	GEMCITABINA	20092153-1
Breast	L01BC05	GEMCITABINA	20091316-1
Breast	L01BC05	GEMCITABINA	20011002-1
Breast	L01BC05	GEMCITABINA	20090678-1
Breast	L01BC05	GEMCITABINA	20011867-1
Breast	L01BC05	GEMCITABINA	20080049-1
Breast	L01BC05	GEMCITABINA	20078383-1
Breast	L01BC05	GEMCITABINA	19970173-1
Breast	L01BC05	GEMCITABINA	20149569-1
Breast	L01BC05	GEMCITABINA	19955836-1
Breast	L01BC05	GEMCITABINA	19955835-1

Breast	L01BC05	GEMCITABINA	20165644-1
Breast	L01BC05	GEMCITABINA	215183-1
Breast	L01BC05	GEMCITABINA	215183-2
Breast	L01BC05	GEMCITABINA	215185-2
Breast	L01BC05	GEMCITABINA	215185-3
Breast	L01BC05	GEMCITABINA	20048010-4
Breast	L01BC05	GEMCITABINA	20048010-5
Breast	L01BC05	GEMCITABINA	20048012-1
Breast	L01BC05	GEMCITABINA	20054873-1
Breast	L01BC05	GEMCITABINA	20055392-1
Breast	L01BC05	GEMCITABINA	20055393-1
Breast	L01BC05	GEMCITABINA	20055730-1
Breast	L01BC05	GEMCITABINA	20055728-1
Breast	L01BC05	GEMCITABINA	20048010-3
Breast	L01BC05	GEMCITABINA	20048010-2
Breast	L01BC05	GEMCITABINA	20048010-1
Breast	L01BC05	GEMCITABINA	20045931-1
Breast	L01BC05	GEMCITABINA	20043052-2
Breast	L01BC05	GEMCITABINA	20043052-1
Breast	L01BC05	GEMCITABINA	20043051-2
Breast	L01BC05	GEMCITABINA	20043051-1
Breast	L01BC05	GEMCITABINA	20062273-1
Breast	L01BC05	GEMCITABINA	20062338-1
Breast	L01BC05	GEMCITABINA	20063469-1
Breast	L01BC05	GEMCITABINA	20063469-2
Breast	L01BC05	GEMCITABINA	20063485-1
Breast	L01BC05	GEMCITABINA	20063486-1
Breast	L01BC05	GEMCITABINA	20063548-1
Breast	L01BC05	GEMCITABINA	20063548-2
Breast	L01BC05	GEMCITABINA	20029564-1
Breast	L01BC05	GEMCITABINA	20021229-1
Breast	L01BC05	GEMCITABINA	20021227-1
Breast	L01BC05	GEMCITABINA	20064604-1
Breast	L01BC05	GEMCITABINA	20013832-1
Breast	L02AE03	GOSERELINA	47155-1
Breast	L02AE03	GOSERELINA	201182-4
Breast	L02AE03	GOSERELINA	201182-5
Breast	L02AE03	GOSERELINA	201182-6
Breast	L02AE03	GOSERELINA	47155-7
Breast	L02AE03	GOSERELINA	47155-2
Breast	L02AE03	GOSERELINA	47155-3
Breast	L02AE03	GOSERELINA	47155-6
Breast	L02AE03	GOSERELINA	201182-2
Breast	L02AE03	GOSERELINA	201182-3
Breast	L02AE03	GOSERELINA	47155-4
Breast	L02AE03	GOSERELINA	47155-5
Breast	L02AE03	GOSERELINA	201182-1
Breast	L01XE07	LAPATINIB	19981554-2

Breast	L01XE07	LAPATINIB	19981554-3
Breast	L01XE07	LAPATINIB	19981554-4
Breast	L01XE07	LAPATINIB	19981554-5
Breast	L01XE07	LAPATINIB	19981554-1
Breast	L01XE07	LAPATINIB	19981554-6
Breast	L01XE07	LAPATINIB	19981554-7
Breast	N02AA01	MORFINA	218192-2
Breast	N02AA01	MORFINA	20013906-1
Breast	N02AA01	MORFINA	20013906-2
Breast	N02AA01	MORFINA	218190-1
Breast	L01CD01	PACLITAXEL	19969254-2
Breast	L01CD01	PACLITAXEL	55979-1
Breast	L01CD01	PACLITAXEL	19915547-1
Breast	L01CD01	PACLITAXEL	19915547-2
Breast	L01CD01	PACLITAXEL	19915547-3
Breast	L01CD01	PACLITAXEL	19937677-1
Breast	L01CD01	PACLITAXEL	222085-1
Breast	L01CD01	PACLITAXEL	19937677-2
Breast	L01CD01	PACLITAXEL	19937677-3
Breast	L01CD01	PACLITAXEL	20057023-1
Breast	L01CD01	PACLITAXEL	20055486-1
Breast	L01CD01	PACLITAXEL	20192030-1
Breast	L01CD01	PACLITAXEL	19946485-1
Breast	L01CD01	PACLITAXEL	19946485-2
Breast	L01CD01	PACLITAXEL	19946485-3
Breast	L01CD01	PACLITAXEL	19946485-4
Breast	L01CD01	PACLITAXEL	19946485-5
Breast	L01CD01	PACLITAXEL	19946485-6
Breast	L01CD01	PACLITAXEL	19952097-1
Breast	L01CD01	PACLITAXEL	19952097-2
Breast	L01CD01	PACLITAXEL	19952097-3
Breast	L01CD01	PACLITAXEL	19952097-4
Breast	L01CD01	PACLITAXEL	19952097-5
Breast	L01CD01	PACLITAXEL	19952097-6
Breast	L01CD01	PACLITAXEL	19952097-7
Breast	L01CD01	PACLITAXEL	19952097-8
Breast	L01CD01	PACLITAXEL	19952110-1
Breast	L01CD01	PACLITAXEL	20061216-1
Breast	L01CD01	PACLITAXEL	20055740-1
Breast	L01CD01	PACLITAXEL	20055999-1
Breast	L01CD01	PACLITAXEL	20047567-1
Breast	L01CD01	PACLITAXEL	19957354-1
Breast	L01CD01	PACLITAXEL	19960065-1
Breast	L01CD01	PACLITAXEL	19963086-1
Breast	L01CD01	PACLITAXEL	19967319-1
Breast	L01CD01	PACLITAXEL	19968458-1
Breast	L01CD01	PACLITAXEL	20148763-1
Breast	L01CD01	PACLITAXEL	19969254-1

Breast	L01CD01	PACLITAXEL	55979-2
Breast	L01CD01	PACLITAXEL	55979-3
Breast	L01CD01	PACLITAXEL	55979-4
Breast	L01CD01	PACLITAXEL	55979-5
Breast	L01CD01	PACLITAXEL	55979-6
Breast	L01CD01	PACLITAXEL	55979-7
Breast	L01CD01	PACLITAXEL	55979-8
Breast	L01CD01	PACLITAXEL	55979-9
Breast	L01CD01	PACLITAXEL	19912566-1
Breast	L01CD01	PACLITAXEL	20107415-1
Breast	L01CD01	PACLITAXEL	19992001-4
Breast	L01CD01	PACLITAXEL	19992001-5
Breast	L01CD01	PACLITAXEL	19992001-6
Breast	L01CD01	PACLITAXEL	19992001-7
Breast	L01CD01	PACLITAXEL	20020276-1
Breast	L01CD01	PACLITAXEL	20018484-1
Breast	L01CD01	PACLITAXEL	20007285-1
Breast	L01CD01	PACLITAXEL	20018483-1
Breast	L01CD01	PACLITAXEL	20018482-1
Breast	L01CD01	PACLITAXEL	20057021-1
Breast	L01CD01	PACLITAXEL	20014772-1
Breast	L01CD01	PACLITAXEL	20014726-1
Breast	L01CD01	PACLITAXEL	20012769-1
Breast	L01CD01	PACLITAXEL	20012772-1
Breast	L01CD01	PACLITAXEL	20071691-1
Breast	L01CD01	PACLITAXEL	20071695-1
Breast	L01CD01	PACLITAXEL	20071697-1
Breast	L01CD01	PACLITAXEL	20107420-1
Breast	L01CD01	PACLITAXEL	19992001-3
Breast	L01CD01	PACLITAXEL	19992001-2
Breast	L01CD01	PACLITAXEL	19992001-1
Breast	L01CD01	PACLITAXEL	19991106-1
Breast	L01CD01	PACLITAXEL	19991105-2
Breast	L01CD01	PACLITAXEL	19991105-1
Breast	L01CD01	PACLITAXEL	19989803-6
Breast	L01CD01	PACLITAXEL	19989803-5
Breast	L01CD01	PACLITAXEL	19989803-4
Breast	L01CD01	PACLITAXEL	19989803-3
Breast	L01CD01	PACLITAXEL	19989803-2
Breast	L01CD01	PACLITAXEL	19989803-1
Breast	L01CD01	PACLITAXEL	20020276-2
Breast	L01CD01	PACLITAXEL	20020276-3
Breast	L01CD01	PACLITAXEL	20056002-1
Breast	L01CD01	PACLITAXEL	20056001-2
Breast	L01CD01	PACLITAXEL	19980164-1
Breast	L01CD01	PACLITAXEL	19979611-1
Breast	L01CD01	PACLITAXEL	20022186-1
Breast	L01CD01	PACLITAXEL	19976519-2

Breast	L01CD01	PACLITAXEL	19976519-1
Breast	L01CD01	PACLITAXEL	20022187-1
Breast	L01CD01	PACLITAXEL	19976518-2
Breast	L01CD01	PACLITAXEL	19976518-1
Breast	L01CD01	PACLITAXEL	19976352-3
Breast	L01CD01	PACLITAXEL	19976352-2
Breast	L01CD01	PACLITAXEL	19976352-1
Breast	L01CD01	PACLITAXEL	19976351-3
Breast	L01CD01	PACLITAXEL	19976351-2
Breast	L01CD01	PACLITAXEL	19976351-1
Breast	L01CD01	PACLITAXEL	19973180-1
Breast	L01CD01	PACLITAXEL	19973179-1
Breast	L01CD01	PACLITAXEL	19973178-1
Breast	L01CD01	PACLITAXEL	19973177-1
Breast	L01CD01	PACLITAXEL	20135292-1
Breast	L01CD01	PACLITAXEL	20047566-1
Breast	L01CD01	PACLITAXEL	20029990-1
Breast	L01CD01	PACLITAXEL	20064116-2
Breast	L01CD01	PACLITAXEL	20064116-1
Breast	L01CD01	PACLITAXEL	20029991-1
Breast	L01CD01	PACLITAXEL	20029992-1
Breast	L01CD01	PACLITAXEL	20031267-1
Breast	L01CD01	PACLITAXEL	20137724-1
Breast	L01CD01	PACLITAXEL	20137724-2
Breast	L01CD01	PACLITAXEL	20137724-3
Breast	L01CD01	PACLITAXEL	20137724-4
Breast	L01CD01	PACLITAXEL	19969254-3
Breast	L01XE33	PALBOCICLIB	20196171-1
Breast	L01XE33	PALBOCICLIB	20145979-7
Breast	L01XE33	PALBOCICLIB	20145979-8
Breast	L01XE33	PALBOCICLIB	20145979-1
Breast	L01XE33	PALBOCICLIB	20145979-10
Breast	L01XE33	PALBOCICLIB	20145979-11
Breast	L01XE33	PALBOCICLIB	20145979-12
Breast	L01XE33	PALBOCICLIB	20145979-2
Breast	L01XE33	PALBOCICLIB	20145979-6
Breast	L01XE33	PALBOCICLIB	20145979-5
Breast	L01XE33	PALBOCICLIB	20145979-9
Breast	L01XE33	PALBOCICLIB	20195993-1
Breast	L01XE33	PALBOCICLIB	20195984-1
Breast	L01XE33	PALBOCICLIB	20145979-3
Breast	L01XE33	PALBOCICLIB	20145979-4
Breast	L01XC13	PERTUZUMAB	20060320-2
Breast	L01XC13	PERTUZUMAB	20060320-1
Breast	L01XY02	PERTUZUMAB Y TRASTUZUMAB	20195976-1
Breast	L01XY02	PERTUZUMAB Y TRASTUZUMAB	20195976-2
Breast	L01XE42	RIBOCICLIB	20115059-3
Breast	L01XE42	RIBOCICLIB	20115059-2

Breast	L01XE42	RIBOCICLIB	20115059-4
Breast	L01XE42	RIBOCICLIB	20115059-5
Breast	L01XE42	RIBOCICLIB	20115059-6
Breast	L01XE42	RIBOCICLIB	20115059-1
Breast	L02BA01	TAMOXIFENO	35448-2
Breast	L02BA01	TAMOXIFENO	35448-3
Breast	L02BA01	TAMOXIFENO	35448-4
Breast	L02BA01	TAMOXIFENO	35448-5
Breast	L02BA01	TAMOXIFENO	35448-6
Breast	L02BA01	TAMOXIFENO	201175-3
Breast	L02BA01	TAMOXIFENO	201175-2
Breast	L02BA01	TAMOXIFENO	201175-1
Breast	L02BA01	TAMOXIFENO	19946123-2
Breast	L02BA01	TAMOXIFENO	19946123-3
Breast	L02BA01	TAMOXIFENO	19953720-1
Breast	L02BA01	TAMOXIFENO	19966221-1
Breast	L02BA01	TAMOXIFENO	19946123-1
Breast	L02BA01	TAMOXIFENO	19944708-9
Breast	L02BA01	TAMOXIFENO	19944708-8
Breast	L02BA01	TAMOXIFENO	19944708-7
Breast	L02BA01	TAMOXIFENO	19944708-6
Breast	L02BA01	TAMOXIFENO	19944708-5
Breast	L02BA01	TAMOXIFENO	19944708-4
Breast	L02BA01	TAMOXIFENO	19944708-3
Breast	L02BA01	TAMOXIFENO	19944708-2
Breast	L02BA01	TAMOXIFENO	19944708-15
Breast	L02BA01	TAMOXIFENO	19944708-14
Breast	L02BA01	TAMOXIFENO	19944708-13
Breast	L02BA01	TAMOXIFENO	19944708-12
Breast	L02BA01	TAMOXIFENO	19944708-11
Breast	L02BA01	TAMOXIFENO	19944708-10
Breast	L02BA01	TAMOXIFENO	19944708-1
Breast	L02BA01	TAMOXIFENO	35442-1
Breast	L02BA01	TAMOXIFENO	35442-2
Breast	L02BA01	TAMOXIFENO	35448-1
Breast	L02BA01	TAMOXIFENO	35442-3
Breast	L01XC03	TRASTUZUMAB	20143846-1
Breast	L01XC03	TRASTUZUMAB	20194635-1
Breast	L01XC03	TRASTUZUMAB	20142329-1
Breast	L01XC03	TRASTUZUMAB	20130360-1
Breast	L01XC03	TRASTUZUMAB	20176291-1
Breast	L01XC03	TRASTUZUMAB	19903070-1
Breast	L01XC03	TRASTUZUMAB	20176292-1
Breast	L01XC03	TRASTUZUMAB	20144826-1
Breast	L01XC14	TRASTUZUMAB EMTANSINA	20064940-1
Breast	L01XC14	TRASTUZUMAB EMTANSINA	20058197-1
Breast	L01XC14	TRASTUZUMAB EMTANSINA	20058197-2
Breast	L01XC14	TRASTUZUMAB EMTANSINA	20064940-2

Cervical	L01XC07	BEVACIZUMAB	20179635-1
Cervical	L01XC07	BEVACIZUMAB	20149223-1
Cervical	L01XC07	BEVACIZUMAB	20169679-3
Cervical	L01XC07	BEVACIZUMAB	20169679-1
Cervical	L01XC07	BEVACIZUMAB	20169679-4
Cervical	L01XC07	BEVACIZUMAB	20149223-2
Cervical	L01XC07	BEVACIZUMAB	20195592-3
Cervical	L01XC07	BEVACIZUMAB	20149223-3
Cervical	L01XC07	BEVACIZUMAB	20195592-2
Cervical	L01XC07	BEVACIZUMAB	20195592-1
Cervical	L01XC07	BEVACIZUMAB	20149223-4
Cervical	L01XC07	BEVACIZUMAB	20195592-4
Cervical	L01XC07	BEVACIZUMAB	20169679-2
Cervical	L01XA02	CARBOPLATINO	19951644-2
Cervical	L01XA02	CARBOPLATINO	19951644-1
Cervical	L01XA02	CARBOPLATINO	19969956-2
Cervical	L01XA02	CARBOPLATINO	19964163-1
Cervical	L01XA02	CARBOPLATINO	20014600-1
Cervical	L01XA02	CARBOPLATINO	19974848-4
Cervical	L01XA02	CARBOPLATINO	19974848-3
Cervical	L01XA02	CARBOPLATINO	19974848-2
Cervical	L01XA02	CARBOPLATINO	19974848-1
Cervical	L01XA02	CARBOPLATINO	19976993-1
Cervical	L01XA02	CARBOPLATINO	19976994-1
Cervical	L01XA02	CARBOPLATINO	19941539-1
Cervical	L01XA02	CARBOPLATINO	19941538-1
Cervical	L01XA02	CARBOPLATINO	19982073-1
Cervical	L01XA02	CARBOPLATINO	20055732-1
Cervical	L01XA02	CARBOPLATINO	19969956-3
Cervical	L01XA02	CARBOPLATINO	20025935-8
Cervical	L01XA02	CARBOPLATINO	19951645-1
Cervical	L01XA02	CARBOPLATINO	19951645-2
Cervical	L01XA02	CARBOPLATINO	19951645-3
Cervical	L01XA02	CARBOPLATINO	20055731-1
Cervical	L01XA02	CARBOPLATINO	19969956-1
Cervical	L01XA02	CARBOPLATINO	20014048-1
Cervical	L01XA02	CARBOPLATINO	19976710-1
Cervical	L01XA02	CARBOPLATINO	20025935-1
Cervical	L01XA02	CARBOPLATINO	20025935-2
Cervical	L01XA02	CARBOPLATINO	20025935-3
Cervical	L01XA02	CARBOPLATINO	19976709-1
Cervical	L01XA02	CARBOPLATINO	20025935-9
Cervical	L01XA02	CARBOPLATINO	19935951-1
Cervical	L01XA02	CARBOPLATINO	20025935-4
Cervical	L01XA02	CARBOPLATINO	20025935-5
Cervical	L01XA02	CARBOPLATINO	20025935-6
Cervical	L01XA02	CARBOPLATINO	19969956-4
Cervical	L01XA02	CARBOPLATINO	19912569-2

Cervical	L01XA02	CARBOPLATINO	19912569-1
Cervical	L01XA02	CARBOPLATINO	19912568-1
Cervical	L01XA02	CARBOPLATINO	20025935-7
Cervical	L01XA02	CARBOPLATINO	20104331-1
Cervical	L01XA02	CARBOPLATINO	20104165-1
Cervical	L01XA02	CARBOPLATINO	19951644-3
Cervical	L01XA01	CISPLATINO	20047565-1
Cervical	L01XA01	CISPLATINO	20006179-1
Cervical	L01XA01	CISPLATINO	19992872-2
Cervical	L01XA01	CISPLATINO	19992872-1
Cervical	L01XA01	CISPLATINO	19992871-1
Cervical	L01XA01	CISPLATINO	19969323-1
Cervical	L01XA01	CISPLATINO	20141904-1
Cervical	L01XA01	CISPLATINO	19982072-1
Cervical	L01XA01	CISPLATINO	20013777-2
Cervical	L01XA01	CISPLATINO	19964252-1
Cervical	L01XA01	CISPLATINO	19963386-4
Cervical	L01XA01	CISPLATINO	19963386-3
Cervical	L01XA01	CISPLATINO	19963386-2
Cervical	L01XA01	CISPLATINO	19963386-1
Cervical	L01XA01	CISPLATINO	20013777-3
Cervical	L01XA01	CISPLATINO	19957108-1
Cervical	L01XA01	CISPLATINO	20095210-1
Cervical	L01XA01	CISPLATINO	20095072-2
Cervical	L01XA01	CISPLATINO	20095072-1
Cervical	L01XA01	CISPLATINO	44821-1
Cervical	L01XA01	CISPLATINO	44823-1
Cervical	L01XA01	CISPLATINO	20035122-1
Cervical	L01XA01	CISPLATINO	20035123-1
Cervical	L01XA01	CISPLATINO	19947842-1
Cervical	L01XA01	CISPLATINO	20047564-1
Cervical	L01XA01	CISPLATINO	19913980-1
Cervical	L01XA01	CISPLATINO	19976715-1
Cervical	L01XA01	CISPLATINO	20142068-1
Cervical	L01XA01	CISPLATINO	20013777-1
Cervical	L01CD02	DOCETAXEL	20113692-1
Cervical	L01CD02	DOCETAXEL	20113688-1
Cervical	L01CD02	DOCETAXEL	20111936-2
Cervical	L01CD02	DOCETAXEL	20111936-1
Cervical	L01CD02	DOCETAXEL	19996764-1
Cervical	L01CD02	DOCETAXEL	19996765-1
Cervical	L01CD02	DOCETAXEL	20111935-2
Cervical	L01CD02	DOCETAXEL	20007286-1
Cervical	L01CD02	DOCETAXEL	20111935-1
Cervical	L01CD02	DOCETAXEL	20020736-1
Cervical	L01CD02	DOCETAXEL	20020736-2
Cervical	L01CD02	DOCETAXEL	20020736-3
Cervical	L01CD02	DOCETAXEL	20022245-1

Cervical	L01CD02	DOCETAXEL	20022245-2
Cervical	L01CD02	DOCETAXEL	20022245-3
Cervical	L01CD02	DOCETAXEL	20024849-1
Cervical	L01CD02	DOCETAXEL	20024849-2
Cervical	L01CD02	DOCETAXEL	20024849-3
Cervical	L01CD02	DOCETAXEL	20028651-1
Cervical	L01CD02	DOCETAXEL	20028651-2
Cervical	L01CD02	DOCETAXEL	20028651-3
Cervical	L01CD02	DOCETAXEL	20045955-1
Cervical	L01CD02	DOCETAXEL	20045960-1
Cervical	L01CD02	DOCETAXEL	20067008-1
Cervical	L01CD02	DOCETAXEL	20047644-1
Cervical	L01CD02	DOCETAXEL	20047646-1
Cervical	L01CD02	DOCETAXEL	20049388-1
Cervical	L01CD02	DOCETAXEL	20064499-1
Cervical	L01CD02	DOCETAXEL	20064497-5
Cervical	L01CD02	DOCETAXEL	20064412-2
Cervical	L01CD02	DOCETAXEL	20064412-1
Cervical	L01CD02	DOCETAXEL	20064460-1
Cervical	L01CD02	DOCETAXEL	20064460-2
Cervical	L01CD02	DOCETAXEL	20064460-3
Cervical	L01CD02	DOCETAXEL	20057737-1
Cervical	L01CD02	DOCETAXEL	20057736-1
Cervical	L01CD02	DOCETAXEL	20064497-1
Cervical	L01CD02	DOCETAXEL	20064497-2
Cervical	L01CD02	DOCETAXEL	20064497-3
Cervical	L01CD02	DOCETAXEL	20064497-4
Cervical	L01CD02	DOCETAXEL	20064412-3
Cervical	L01CD02	DOCETAXEL	230189-2
Cervical	L01CD02	DOCETAXEL	230187-1
Cervical	L01CD02	DOCETAXEL	19969253-1
Cervical	L01CD02	DOCETAXEL	19969253-2
Cervical	L01CD02	DOCETAXEL	19969253-3
Cervical	L01CD02	DOCETAXEL	19969253-4
Cervical	L01CD02	DOCETAXEL	19969253-5
Cervical	L01CD02	DOCETAXEL	19969842-1
Cervical	L01CD02	DOCETAXEL	19969842-2
Cervical	L01CD02	DOCETAXEL	19969842-3
Cervical	L01CD02	DOCETAXEL	19974847-1
Cervical	L01BC05	GEMCITABINA	19955836-1
Cervical	L01BC05	GEMCITABINA	20043052-2
Cervical	L01BC05	GEMCITABINA	20045931-1
Cervical	L01BC05	GEMCITABINA	20062273-1
Cervical	L01BC05	GEMCITABINA	19997219-1
Cervical	L01BC05	GEMCITABINA	20062338-1
Cervical	L01BC05	GEMCITABINA	20063469-1
Cervical	L01BC05	GEMCITABINA	20063485-1
Cervical	L01BC05	GEMCITABINA	19970173-1

Cervical	L01BC05	GEMCITABINA	19970173-2
Cervical	L01BC05	GEMCITABINA	19970173-3
Cervical	L01BC05	GEMCITABINA	20080049-1
Cervical	L01BC05	GEMCITABINA	20090678-1
Cervical	L01BC05	GEMCITABINA	20021229-1
Cervical	L01BC05	GEMCITABINA	20021227-1
Cervical	L01BC05	GEMCITABINA	19970173-4
Cervical	L01BC05	GEMCITABINA	20048012-1
Cervical	L01BC05	GEMCITABINA	20048010-5
Cervical	L01BC05	GEMCITABINA	20091316-1
Cervical	L01BC05	GEMCITABINA	20092153-1
Cervical	L01BC05	GEMCITABINA	215185-3
Cervical	L01BC05	GEMCITABINA	20048010-2
Cervical	L01BC05	GEMCITABINA	215185-2
Cervical	L01BC05	GEMCITABINA	215183-2
Cervical	L01BC05	GEMCITABINA	215183-1
Cervical	L01BC05	GEMCITABINA	19955835-1
Cervical	L01BC05	GEMCITABINA	20043052-1
Cervical	L01BC05	GEMCITABINA	20043051-2
Cervical	L01BC05	GEMCITABINA	20043051-1
Cervical	L01BC05	GEMCITABINA	20078383-1
Cervical	L01BC05	GEMCITABINA	20029564-1
Cervical	L01BC05	GEMCITABINA	20013832-1
Cervical	L01BC05	GEMCITABINA	19970173-6
Cervical	L01BC05	GEMCITABINA	19970173-7
Cervical	L01BC05	GEMCITABINA	19970173-8
Cervical	L01BC05	GEMCITABINA	20165644-1
Cervical	L01BC05	GEMCITABINA	20063548-2
Cervical	L01BC05	GEMCITABINA	19978376-1
Cervical	L01BC05	GEMCITABINA	20063486-1
Cervical	L01BC05	GEMCITABINA	20011867-1
Cervical	L01BC05	GEMCITABINA	20011002-1
Cervical	L01BC05	GEMCITABINA	20010934-1
Cervical	L01BC05	GEMCITABINA	20010932-1
Cervical	L01BC05	GEMCITABINA	20063469-2
Cervical	L01BC05	GEMCITABINA	20063548-1
Cervical	L01BC05	GEMCITABINA	20149569-1
Cervical	L01BC05	GEMCITABINA	20048010-4
Cervical	L01BC05	GEMCITABINA	20048010-3
Cervical	L01BC05	GEMCITABINA	19997220-1
Cervical	L01BC05	GEMCITABINA	20055730-1
Cervical	L01BC05	GEMCITABINA	20055728-1
Cervical	L01BC05	GEMCITABINA	20064604-1
Cervical	L01BC05	GEMCITABINA	20055393-1
Cervical	L01BC05	GEMCITABINA	20078381-1
Cervical	L01BC05	GEMCITABINA	20054873-1
Cervical	L01BC05	GEMCITABINA	20055392-1
Cervical	L01BC05	GEMCITABINA	19970173-5

Cervical	L01BC05	GEMCITABINA	20048010-1
Cervical	L01XX19	IRINOTECAN	20045957-1
Cervical	L01XX19	IRINOTECAN	20141121-1
Cervical	L01XX19	IRINOTECAN	20141121-2
Cervical	L01XX19	IRINOTECAN	207609-12
Cervical	L01XX19	IRINOTECAN	207609-11
Cervical	L01XX19	IRINOTECAN	207609-10
Cervical	L01XX19	IRINOTECAN	19984431-1
Cervical	L01XX19	IRINOTECAN	207609-1
Cervical	L01XX19	IRINOTECAN	19973304-1
Cervical	L01XX19	IRINOTECAN	19974846-1
Cervical	L01XX19	IRINOTECAN	19976711-1
Cervical	L01XX19	IRINOTECAN	207609-2
Cervical	L01XX19	IRINOTECAN	207609-3
Cervical	L01XX19	IRINOTECAN	207609-4
Cervical	L01XX19	IRINOTECAN	19993941-1
Cervical	L01XX19	IRINOTECAN	207609-5
Cervical	L01XX19	IRINOTECAN	20055733-1
Cervical	L01XX19	IRINOTECAN	19938138-1
Cervical	L01XX19	IRINOTECAN	19938138-2
Cervical	L01XX19	IRINOTECAN	19938138-3
Cervical	L01XX19	IRINOTECAN	20025051-3
Cervical	L01XX19	IRINOTECAN	20025051-2
Cervical	L01XX19	IRINOTECAN	20025051-1
Cervical	L01XX19	IRINOTECAN	19938138-4
Cervical	L01XX19	IRINOTECAN	19938138-5
Cervical	L01XX19	IRINOTECAN	19952096-1
Cervical	L01XX19	IRINOTECAN	19952096-2
Cervical	L01XX19	IRINOTECAN	19952096-3
Cervical	L01XX19	IRINOTECAN	19952096-4
Cervical	L01XX19	IRINOTECAN	19952096-5
Cervical	L01XX19	IRINOTECAN	20018486-1
Cervical	L01XX19	IRINOTECAN	207609-9
Cervical	L01XX19	IRINOTECAN	207609-8
Cervical	L01XX19	IRINOTECAN	207609-7
Cervical	L01XX19	IRINOTECAN	19967207-1
Cervical	L01XX19	IRINOTECAN	19967207-2
Cervical	L01XX19	IRINOTECAN	19967207-3
Cervical	L01XX19	IRINOTECAN	20012770-1
Cervical	L01XX19	IRINOTECAN	20111292-1
Cervical	L01XX19	IRINOTECAN	20012621-1
Cervical	L01XX19	IRINOTECAN	20010926-1
Cervical	L01XX19	IRINOTECAN	207609-6
Cervical	L01XX19	IRINOTECAN	20053624-1
Cervical	N02AA01	MORFINA	218192-2
Cervical	N02AA01	MORFINA	20013906-1
Cervical	N02AA01	MORFINA	20013906-2
Cervical	N02AA01	MORFINA	218190-1

Cervical	L01CD01	PACLITAXEL	20071691-1
Cervical	L01CD01	PACLITAXEL	20071695-1
Cervical	L01CD01	PACLITAXEL	20071697-1
Cervical	L01CD01	PACLITAXEL	20107415-1
Cervical	L01CD01	PACLITAXEL	20107420-1
Cervical	L01CD01	PACLITAXEL	20137724-1
Cervical	L01CD01	PACLITAXEL	20137724-2
Cervical	L01CD01	PACLITAXEL	20137724-3
Cervical	L01CD01	PACLITAXEL	20137724-4
Cervical	L01CD01	PACLITAXEL	20148763-1
Cervical	L01CD01	PACLITAXEL	20192030-1
Cervical	L01CD01	PACLITAXEL	222085-1
Cervical	L01CD01	PACLITAXEL	55979-1
Cervical	L01CD01	PACLITAXEL	55979-2
Cervical	L01CD01	PACLITAXEL	55979-3
Cervical	L01CD01	PACLITAXEL	55979-4
Cervical	L01CD01	PACLITAXEL	55979-5
Cervical	L01CD01	PACLITAXEL	55979-6
Cervical	L01CD01	PACLITAXEL	55979-7
Cervical	L01CD01	PACLITAXEL	55979-8
Cervical	L01CD01	PACLITAXEL	55979-9
Cervical	L01CD01	PACLITAXEL	20135292-1
Cervical	L01CD01	PACLITAXEL	19912566-1
Cervical	L01CD01	PACLITAXEL	19915547-1
Cervical	L01CD01	PACLITAXEL	19915547-2
Cervical	L01CD01	PACLITAXEL	19915547-3
Cervical	L01CD01	PACLITAXEL	19937677-1
Cervical	L01CD01	PACLITAXEL	19937677-2
Cervical	L01CD01	PACLITAXEL	19937677-3
Cervical	L01CD01	PACLITAXEL	19946485-1
Cervical	L01CD01	PACLITAXEL	19946485-2
Cervical	L01CD01	PACLITAXEL	19946485-3
Cervical	L01CD01	PACLITAXEL	19946485-4
Cervical	L01CD01	PACLITAXEL	19946485-5
Cervical	L01CD01	PACLITAXEL	19946485-6
Cervical	L01CD01	PACLITAXEL	19952097-1
Cervical	L01CD01	PACLITAXEL	19952097-2
Cervical	L01CD01	PACLITAXEL	19952097-3
Cervical	L01CD01	PACLITAXEL	19952097-4
Cervical	L01CD01	PACLITAXEL	19952097-5
Cervical	L01CD01	PACLITAXEL	19952097-6
Cervical	L01CD01	PACLITAXEL	19952097-7
Cervical	L01CD01	PACLITAXEL	19952097-8
Cervical	L01CD01	PACLITAXEL	19952110-1
Cervical	L01CD01	PACLITAXEL	19957354-1
Cervical	L01CD01	PACLITAXEL	19960065-1
Cervical	L01CD01	PACLITAXEL	19963086-1
Cervical	L01CD01	PACLITAXEL	19967319-1

Cervical	L01CD01	PACLITAXEL	19968458-1
Cervical	L01CD01	PACLITAXEL	19969254-1
Cervical	L01CD01	PACLITAXEL	19969254-2
Cervical	L01CD01	PACLITAXEL	19969254-3
Cervical	L01CD01	PACLITAXEL	19973177-1
Cervical	L01CD01	PACLITAXEL	19973178-1
Cervical	L01CD01	PACLITAXEL	19973179-1
Cervical	L01CD01	PACLITAXEL	19973180-1
Cervical	L01CD01	PACLITAXEL	19976351-1
Cervical	L01CD01	PACLITAXEL	19976351-2
Cervical	L01CD01	PACLITAXEL	19976351-3
Cervical	L01CD01	PACLITAXEL	19976352-1
Cervical	L01CD01	PACLITAXEL	19976352-2
Cervical	L01CD01	PACLITAXEL	19976352-3
Cervical	L01CD01	PACLITAXEL	19976518-1
Cervical	L01CD01	PACLITAXEL	19976518-2
Cervical	L01CD01	PACLITAXEL	19976519-1
Cervical	L01CD01	PACLITAXEL	19976519-2
Cervical	L01CD01	PACLITAXEL	19979611-1
Cervical	L01CD01	PACLITAXEL	19980164-1
Cervical	L01CD01	PACLITAXEL	19989803-1
Cervical	L01CD01	PACLITAXEL	19989803-2
Cervical	L01CD01	PACLITAXEL	19989803-3
Cervical	L01CD01	PACLITAXEL	19989803-4
Cervical	L01CD01	PACLITAXEL	19989803-5
Cervical	L01CD01	PACLITAXEL	19989803-6
Cervical	L01CD01	PACLITAXEL	19991105-1
Cervical	L01CD01	PACLITAXEL	19991105-2
Cervical	L01CD01	PACLITAXEL	19991106-1
Cervical	L01CD01	PACLITAXEL	19992001-1
Cervical	L01CD01	PACLITAXEL	19992001-2
Cervical	L01CD01	PACLITAXEL	19992001-3
Cervical	L01CD01	PACLITAXEL	19992001-4
Cervical	L01CD01	PACLITAXEL	19992001-5
Cervical	L01CD01	PACLITAXEL	19992001-6
Cervical	L01CD01	PACLITAXEL	19992001-7
Cervical	L01CD01	PACLITAXEL	20007285-1
Cervical	L01CD01	PACLITAXEL	20012769-1
Cervical	L01CD01	PACLITAXEL	20012772-1
Cervical	L01CD01	PACLITAXEL	20014726-1
Cervical	L01CD01	PACLITAXEL	20014772-1
Cervical	L01CD01	PACLITAXEL	20018482-1
Cervical	L01CD01	PACLITAXEL	20018483-1
Cervical	L01CD01	PACLITAXEL	20018484-1
Cervical	L01CD01	PACLITAXEL	20020276-1
Cervical	L01CD01	PACLITAXEL	20020276-2
Cervical	L01CD01	PACLITAXEL	20020276-3
Cervical	L01CD01	PACLITAXEL	20022186-1

Cervical	L01CD01	PACLITAXEL	20022187-1
Cervical	L01CD01	PACLITAXEL	20029990-1
Cervical	L01CD01	PACLITAXEL	20029991-1
Cervical	L01CD01	PACLITAXEL	20029992-1
Cervical	L01CD01	PACLITAXEL	20031267-1
Cervical	L01CD01	PACLITAXEL	20047566-1
Cervical	L01CD01	PACLITAXEL	20047567-1
Cervical	L01CD01	PACLITAXEL	20055486-1
Cervical	L01CD01	PACLITAXEL	20055740-1
Cervical	L01CD01	PACLITAXEL	20055999-1
Cervical	L01CD01	PACLITAXEL	20056001-2
Cervical	L01CD01	PACLITAXEL	20056002-1
Cervical	L01CD01	PACLITAXEL	20057021-1
Cervical	L01CD01	PACLITAXEL	20057023-1
Cervical	L01CD01	PACLITAXEL	20061216-1
Cervical	L01CD01	PACLITAXEL	20064116-1
Cervical	L01CD01	PACLITAXEL	20064116-2
Cervical	L01XX17	TOPOTECAN	19986863-1
Cervical	L01XX17	TOPOTECAN	20010613-1
Cervical	L01XX17	TOPOTECAN	230185-3
Cervical	L01XX17	TOPOTECAN	230185-2
Cervical	L01XX17	TOPOTECAN	230185-1
Colorectal	S01LA05	AFLIBERCEPT	20039088-3
Colorectal	S01LA05	AFLIBERCEPT	20039088-7
Colorectal	S01LA05	AFLIBERCEPT	20039088-8
Colorectal	S01LA05	AFLIBERCEPT	20039088-4
Colorectal	S01LA05	AFLIBERCEPT	20039088-6
Colorectal	S01LA05	AFLIBERCEPT	20039088-5
Colorectal	S01LA05	AFLIBERCEPT	20039088-9
Colorectal	S01LA05	AFLIBERCEPT	20039088-1
Colorectal	S01LA05	AFLIBERCEPT	20039088-11
Colorectal	S01LA05	AFLIBERCEPT	20039088-10
Colorectal	S01LA05	AFLIBERCEPT	20039088-12
Colorectal	S01LA05	AFLIBERCEPT	20039088-2
Colorectal	L01XC07	BEVACIZUMAB	20149223-2
Colorectal	L01XC07	BEVACIZUMAB	20149223-3
Colorectal	L01XC07	BEVACIZUMAB	20149223-4
Colorectal	L01XC07	BEVACIZUMAB	20195592-3
Colorectal	L01XC07	BEVACIZUMAB	20195592-4
Colorectal	L01XC07	BEVACIZUMAB	20169679-1
Colorectal	L01XC07	BEVACIZUMAB	20169679-2
Colorectal	L01XC07	BEVACIZUMAB	20169679-3
Colorectal	L01XC07	BEVACIZUMAB	20169679-4
Colorectal	L01XC07	BEVACIZUMAB	20179635-1
Colorectal	L01XC07	BEVACIZUMAB	20195592-1
Colorectal	L01XC07	BEVACIZUMAB	20195592-2
Colorectal	L01XC07	BEVACIZUMAB	20149223-1
Colorectal	V03AF03	CALCIO FOLINATO	19940090-1

Colorectal	V03AF03	CALCIO FOLINATO	20139379-1
Colorectal	V03AF03	CALCIO FOLINATO	20139379-2
Colorectal	V03AF03	CALCIO FOLINATO	19989251-1
Colorectal	V03AF03	CALCIO FOLINATO	19989251-2
Colorectal	V03AF03	CALCIO FOLINATO	19989251-3
Colorectal	V03AF03	CALCIO FOLINATO	19989251-4
Colorectal	V03AF03	CALCIO FOLINATO	19989251-5
Colorectal	V03AF03	CALCIO FOLINATO	48266-4
Colorectal	V03AF03	CALCIO FOLINATO	19953722-1
Colorectal	V03AF03	CALCIO FOLINATO	19952111-1
Colorectal	V03AF03	CALCIO FOLINATO	20138364-1
Colorectal	V03AF03	CALCIO FOLINATO	20139847-2
Colorectal	V03AF03	CALCIO FOLINATO	201934-1
Colorectal	V03AF03	CALCIO FOLINATO	20139847-1
Colorectal	V03AF03	CALCIO FOLINATO	51488-2
Colorectal	V03AF03	CALCIO FOLINATO	51488-1
Colorectal	V03AF03	CALCIO FOLINATO	48266-6
Colorectal	V03AF03	CALCIO FOLINATO	19930613-1
Colorectal	V03AF03	CALCIO FOLINATO	20006242-1
Colorectal	V03AF03	CALCIO FOLINATO	48266-5
Colorectal	V03AF03	CALCIO FOLINATO	48266-3
Colorectal	V03AF03	CALCIO FOLINATO	48266-2
Colorectal	V03AF03	CALCIO FOLINATO	48266-1
Colorectal	V03AF03	CALCIO FOLINATO	19953721-1
Colorectal	V03AF03	CALCIO FOLINATO	20213225-1
Colorectal	V03AF03	CALCIO FOLINATO	20139379-3
Colorectal	V03AF03	CALCIO FOLINATO	51488-3
Colorectal	L01BC06	CAPECITABINA	20053629-1
Colorectal	L01BC06	CAPECITABINA	20060323-1
Colorectal	L01BC06	CAPECITABINA	20060323-10
Colorectal	L01BC06	CAPECITABINA	20060323-11
Colorectal	L01BC06	CAPECITABINA	20060323-12
Colorectal	L01BC06	CAPECITABINA	20060323-13
Colorectal	L01BC06	CAPECITABINA	20060323-14
Colorectal	L01BC06	CAPECITABINA	20060323-15
Colorectal	L01BC06	CAPECITABINA	20060323-2
Colorectal	L01BC06	CAPECITABINA	20060323-3
Colorectal	L01BC06	CAPECITABINA	20060323-4
Colorectal	L01BC06	CAPECITABINA	20060323-5
Colorectal	L01BC06	CAPECITABINA	20060323-6
Colorectal	L01BC06	CAPECITABINA	20060323-7
Colorectal	L01BC06	CAPECITABINA	20060323-8
Colorectal	L01BC06	CAPECITABINA	20060323-9
Colorectal	L01BC06	CAPECITABINA	20104404-1
Colorectal	L01BC06	CAPECITABINA	20111735-1
Colorectal	L01BC06	CAPECITABINA	20116817-10
Colorectal	L01BC06	CAPECITABINA	20116817-2
Colorectal	L01BC06	CAPECITABINA	20116817-3

Colorectal	L01BC06	CAPECITABINA	20116817-4
Colorectal	L01BC06	CAPECITABINA	20116817-5
Colorectal	L01BC06	CAPECITABINA	20116817-6
Colorectal	L01BC06	CAPECITABINA	20116817-7
Colorectal	L01BC06	CAPECITABINA	20116817-8
Colorectal	L01BC06	CAPECITABINA	20116817-9
Colorectal	L01BC06	CAPECITABINA	20132905-2
Colorectal	L01BC06	CAPECITABINA	20132905-3
Colorectal	L01BC06	CAPECITABINA	20132905-4
Colorectal	L01BC06	CAPECITABINA	20132905-5
Colorectal	L01BC06	CAPECITABINA	20132906-1
Colorectal	L01BC06	CAPECITABINA	20132906-2
Colorectal	L01BC06	CAPECITABINA	20132906-3
Colorectal	L01BC06	CAPECITABINA	20132906-4
Colorectal	L01BC06	CAPECITABINA	20146928-1
Colorectal	L01BC06	CAPECITABINA	20152744-1
Colorectal	L01BC06	CAPECITABINA	20155908-1
Colorectal	L01BC06	CAPECITABINA	20155908-2
Colorectal	L01BC06	CAPECITABINA	20155908-3
Colorectal	L01BC06	CAPECITABINA	20155908-4
Colorectal	L01BC06	CAPECITABINA	20155908-5
Colorectal	L01BC06	CAPECITABINA	20155908-6
Colorectal	L01BC06	CAPECITABINA	20155908-7
Colorectal	L01BC06	CAPECITABINA	20155908-8
Colorectal	L01BC06	CAPECITABINA	20155908-9
Colorectal	L01BC06	CAPECITABINA	20160260-1
Colorectal	L01BC06	CAPECITABINA	20160260-2
Colorectal	L01BC06	CAPECITABINA	20160260-3
Colorectal	L01BC06	CAPECITABINA	20160260-4
Colorectal	L01BC06	CAPECITABINA	229745-1
Colorectal	L01BC06	CAPECITABINA	20132905-1
Colorectal	L01BC06	CAPECITABINA	20116817-1
Colorectal	L01FE01	CETUXIMAB	19953428-3
Colorectal	L01FE01	CETUXIMAB	19953428-4
Colorectal	L01FE01	CETUXIMAB	19953428-2
Colorectal	L01FE01	CETUXIMAB	19953428-5
Colorectal	L01FE01	CETUXIMAB	19953428-1
Colorectal	L01BC08	DECITABINA	20097119-2
Colorectal	L01BC08	DECITABINA	20114014-1
Colorectal	L01BC08	DECITABINA	20096772-1
Colorectal	L01BC08	DECITABINA	20148281-1
Colorectal	L01BC08	DECITABINA	19991325-2
Colorectal	L01BC08	DECITABINA	20192584-1
Colorectal	L01BC08	DECITABINA	19991325-1
Colorectal	L01BC08	DECITABINA	20097119-1
Colorectal	L01BC02	FLUOROURACILO	19964018-2
Colorectal	L01BC02	FLUOROURACILO	19964018-3
Colorectal	L01BC02	FLUOROURACILO	19964018-4

Colorectal	L01BC02	FLUOROURACILO	19964018-5
Colorectal	L01BC02	FLUOROURACILO	19964018-6
Colorectal	L01BC02	FLUOROURACILO	19930999-2
Colorectal	L01BC02	FLUOROURACILO	20071503-1
Colorectal	L01BC02	FLUOROURACILO	20071503-2
Colorectal	L01BC02	FLUOROURACILO	19964018-1
Colorectal	L01BC02	FLUOROURACILO	19964018-7
Colorectal	L01BC02	FLUOROURACILO	20053621-1
Colorectal	L01BC02	FLUOROURACILO	19930999-5
Colorectal	L01BC02	FLUOROURACILO	19930999-4
Colorectal	L01BC02	FLUOROURACILO	20133414-1
Colorectal	L01BC02	FLUOROURACILO	20043050-4
Colorectal	L01BC02	FLUOROURACILO	19930999-1
Colorectal	L01BC02	FLUOROURACILO	20043050-3
Colorectal	L01BC02	FLUOROURACILO	20043050-1
Colorectal	L01BC02	FLUOROURACILO	20043050-2
Colorectal	L01BC02	FLUOROURACILO	227728-2
Colorectal	L01BC02	FLUOROURACILO	19930999-3
Colorectal	L01BC02	FLUOROURACILO	227728-1
Colorectal	L01BC02	FLUOROURACILO	20043050-7
Colorectal	L01BC02	FLUOROURACILO	20043050-6
Colorectal	L01BC02	FLUOROURACILO	20043050-5
Colorectal	L01BC02	FLUOROURACILO	216264-3
Colorectal	L01BC02	FLUOROURACILO	216264-2
Colorectal	L01BC02	FLUOROURACILO	216264-1
Colorectal	L01BC02	FLUOROURACILO	20121175-1
Colorectal	L01BC02	FLUOROURACILO	20121175-2
Colorectal	L01BC02	FLUOROURACILO	20125230-1
Colorectal	L01BC02	FLUOROURACILO	20125230-2
Colorectal	L01BC02	FLUOROURACILO	19964018-8
Colorectal	L01XX19	IRINOTECAN	19984431-1
Colorectal	L01XX19	IRINOTECAN	207609-7
Colorectal	L01XX19	IRINOTECAN	207609-6
Colorectal	L01XX19	IRINOTECAN	19976711-1
Colorectal	L01XX19	IRINOTECAN	19974846-1
Colorectal	L01XX19	IRINOTECAN	207609-5
Colorectal	L01XX19	IRINOTECAN	207609-4
Colorectal	L01XX19	IRINOTECAN	19973304-1
Colorectal	L01XX19	IRINOTECAN	207609-3
Colorectal	L01XX19	IRINOTECAN	207609-2
Colorectal	L01XX19	IRINOTECAN	207609-12
Colorectal	L01XX19	IRINOTECAN	207609-11
Colorectal	L01XX19	IRINOTECAN	207609-10
Colorectal	L01XX19	IRINOTECAN	20141121-1
Colorectal	L01XX19	IRINOTECAN	20141121-2
Colorectal	L01XX19	IRINOTECAN	207609-1
Colorectal	L01XX19	IRINOTECAN	19967207-3
Colorectal	L01XX19	IRINOTECAN	19967207-2

Colorectal	L01XX19	IRINOTECAN	19967207-1
Colorectal	L01XX19	IRINOTECAN	19952096-1
Colorectal	L01XX19	IRINOTECAN	19952096-2
Colorectal	L01XX19	IRINOTECAN	19938138-5
Colorectal	L01XX19	IRINOTECAN	20053624-1
Colorectal	L01XX19	IRINOTECAN	20055733-1
Colorectal	L01XX19	IRINOTECAN	19952096-5
Colorectal	L01XX19	IRINOTECAN	19938138-4
Colorectal	L01XX19	IRINOTECAN	19938138-3
Colorectal	L01XX19	IRINOTECAN	207609-9
Colorectal	L01XX19	IRINOTECAN	19993941-1
Colorectal	L01XX19	IRINOTECAN	20045957-1
Colorectal	L01XX19	IRINOTECAN	20010926-1
Colorectal	L01XX19	IRINOTECAN	20012621-1
Colorectal	L01XX19	IRINOTECAN	20111292-1
Colorectal	L01XX19	IRINOTECAN	19952096-3
Colorectal	L01XX19	IRINOTECAN	20012770-1
Colorectal	L01XX19	IRINOTECAN	19952096-4
Colorectal	L01XX19	IRINOTECAN	19938138-1
Colorectal	L01XX19	IRINOTECAN	19938138-2
Colorectal	L01XX19	IRINOTECAN	20018486-1
Colorectal	L01XX19	IRINOTECAN	20025051-1
Colorectal	L01XX19	IRINOTECAN	20025051-2
Colorectal	L01XX19	IRINOTECAN	20025051-3
Colorectal	L01XX19	IRINOTECAN	207609-8
Colorectal	N02AA01	MORFINA	218192-2
Colorectal	N02AA01	MORFINA	20013906-1
Colorectal	N02AA01	MORFINA	20013906-2
Colorectal	N02AA01	MORFINA	218190-1
Colorectal	L01XA03	OXALIPLATINO	19939159-2
Colorectal	L01XA03	OXALIPLATINO	20044067-3
Colorectal	L01XA03	OXALIPLATINO	223928-1
Colorectal	L01XA03	OXALIPLATINO	223932-1
Colorectal	L01XA03	OXALIPLATINO	19963088-1
Colorectal	L01XA03	OXALIPLATINO	19963084-1
Colorectal	L01XA03	OXALIPLATINO	19939159-3
Colorectal	L01XA03	OXALIPLATINO	19939159-4
Colorectal	L01XA03	OXALIPLATINO	19952112-1
Colorectal	L01XA03	OXALIPLATINO	19952112-2
Colorectal	L01XA03	OXALIPLATINO	20044067-2
Colorectal	L01XA03	OXALIPLATINO	20044068-1
Colorectal	L01XA03	OXALIPLATINO	20044068-2
Colorectal	L01XA03	OXALIPLATINO	20044068-3
Colorectal	L01XA03	OXALIPLATINO	20044068-4
Colorectal	L01XA03	OXALIPLATINO	20044068-5
Colorectal	L01XA03	OXALIPLATINO	20045956-1
Colorectal	L01XA03	OXALIPLATINO	20045959-1
Colorectal	L01XA03	OXALIPLATINO	20047606-1

Colorectal	L01XA03	OXALIPLATINO	20044067-1
Colorectal	L01XA03	OXALIPLATINO	20056003-1
Colorectal	L01XA03	OXALIPLATINO	20056004-1
Colorectal	L01XA03	OXALIPLATINO	20029508-1
Colorectal	L01XA03	OXALIPLATINO	20062339-1
Colorectal	L01XA03	OXALIPLATINO	20063579-1
Colorectal	L01XA03	OXALIPLATINO	20067533-1
Colorectal	L01XA03	OXALIPLATINO	20104411-1
Colorectal	L01XA03	OXALIPLATINO	20109105-1
Colorectal	L01XA03	OXALIPLATINO	20109106-1
Colorectal	L01XA03	OXALIPLATINO	20011830-1
Colorectal	L01XA03	OXALIPLATINO	19997648-1
Colorectal	L01XA03	OXALIPLATINO	19993868-1
Colorectal	L01XA03	OXALIPLATINO	19989995-4
Colorectal	L01XA03	OXALIPLATINO	19989995-3
Colorectal	L01XA03	OXALIPLATINO	19989995-2
Colorectal	L01XA03	OXALIPLATINO	19989995-1
Colorectal	L01XA03	OXALIPLATINO	19978042-1
Colorectal	L01XA03	OXALIPLATINO	19976714-1
Colorectal	L01XA03	OXALIPLATINO	19973306-1
Colorectal	L01XA03	OXALIPLATINO	19973305-1
Colorectal	L01XA03	OXALIPLATINO	19969843-6
Colorectal	L01XA03	OXALIPLATINO	19969843-5
Colorectal	L01XA03	OXALIPLATINO	19969843-4
Colorectal	L01XA03	OXALIPLATINO	19969843-3
Colorectal	L01XA03	OXALIPLATINO	19969843-2
Colorectal	L01XA03	OXALIPLATINO	19969843-1
Colorectal	L01XA03	OXALIPLATINO	20151658-1
Colorectal	L01XA03	OXALIPLATINO	20151658-2
Colorectal	L01XA03	OXALIPLATINO	20151658-3
Colorectal	L01XA03	OXALIPLATINO	19939159-1
Colorectal	L01XC08	PANITUMUMAB	20025916-1
Colorectal	L01XC08	PANITUMUMAB	20025916-2
Colorectal	L01XC08	PANITUMUMAB	20025916-3
Colorectal	L01XC18	PEMBROLIZUMAB	20085509-1
Colorectal	L01XC21	RAMUCIRUMAB	20111011-3
Colorectal	L01XC21	RAMUCIRUMAB	20111011-4
Colorectal	L01XC21	RAMUCIRUMAB	20111011-2
Colorectal	L01XC21	RAMUCIRUMAB	20111011-1
Colorectal	L01XE21	REGORAFENIB	20152989-3
Colorectal	L01XE21	REGORAFENIB	20152989-2
Colorectal	L01XE21	REGORAFENIB	20152989-1
Lung	L01EB03	AFATINIB	20066634-2
Lung	L01EB03	AFATINIB	20093590-3
Lung	L01EB03	AFATINIB	20093591-1
Lung	L01EB03	AFATINIB	20093591-3
Lung	L01EB03	AFATINIB	20066634-3
Lung	L01EB03	AFATINIB	20066634-1

Lung	L01EB03	AFATINIB	20093590-1
Lung	L01EB03	AFATINIB	20093591-2
Lung	L01EB03	AFATINIB	20093590-2
Lung	L01ED03	ALECTINIB	20157000-2
Lung	L01ED03	ALECTINIB	20157000-1
Lung	L01XC32	ATEZOLIZUMAB	20145962-2
Lung	L01XC32	ATEZOLIZUMAB	20145962-1
Lung	L01XC07	BEVACIZUMAB	20179635-1
Lung	L01XC07	BEVACIZUMAB	20169679-4
Lung	L01XC07	BEVACIZUMAB	20169679-1
Lung	L01XC07	BEVACIZUMAB	20169679-2
Lung	L01XC07	BEVACIZUMAB	20195592-1
Lung	L01XC07	BEVACIZUMAB	20149223-4
Lung	L01XC07	BEVACIZUMAB	20195592-3
Lung	L01XC07	BEVACIZUMAB	20149223-3
Lung	L01XC07	BEVACIZUMAB	20195592-4
Lung	L01XC07	BEVACIZUMAB	20149223-2
Lung	L01XC07	BEVACIZUMAB	20169679-3
Lung	L01XC07	BEVACIZUMAB	20195592-2
Lung	L01XC07	BEVACIZUMAB	20149223-1
Lung	L01ED04	BRIGATINIB	20193918-1
Lung	L01ED04	BRIGATINIB	20193918-2
Lung	L01XA02	CARBOPLATINO	19951644-3
Lung	L01XA02	CARBOPLATINO	19951645-1
Lung	L01XA02	CARBOPLATINO	19951645-2
Lung	L01XA02	CARBOPLATINO	19951645-3
Lung	L01XA02	CARBOPLATINO	19974848-4
Lung	L01XA02	CARBOPLATINO	20014600-1
Lung	L01XA02	CARBOPLATINO	19974848-3
Lung	L01XA02	CARBOPLATINO	20014048-1
Lung	L01XA02	CARBOPLATINO	19974848-2
Lung	L01XA02	CARBOPLATINO	19974848-1
Lung	L01XA02	CARBOPLATINO	20104165-1
Lung	L01XA02	CARBOPLATINO	19964163-1
Lung	L01XA02	CARBOPLATINO	19982073-1
Lung	L01XA02	CARBOPLATINO	20104331-1
Lung	L01XA02	CARBOPLATINO	19969956-4
Lung	L01XA02	CARBOPLATINO	19969956-3
Lung	L01XA02	CARBOPLATINO	19969956-1
Lung	L01XA02	CARBOPLATINO	19976994-1
Lung	L01XA02	CARBOPLATINO	19976993-1
Lung	L01XA02	CARBOPLATINO	19976710-1
Lung	L01XA02	CARBOPLATINO	19976709-1
Lung	L01XA02	CARBOPLATINO	20055732-1
Lung	L01XA02	CARBOPLATINO	20055731-1
Lung	L01XA02	CARBOPLATINO	20025935-9
Lung	L01XA02	CARBOPLATINO	20025935-8
Lung	L01XA02	CARBOPLATINO	20025935-7

Lung	L01XA02	CARBOPLATINO	20025935-6
Lung	L01XA02	CARBOPLATINO	19969956-2
Lung	L01XA02	CARBOPLATINO	20025935-5
Lung	L01XA02	CARBOPLATINO	20025935-4
Lung	L01XA02	CARBOPLATINO	20025935-3
Lung	L01XA02	CARBOPLATINO	20025935-2
Lung	L01XA02	CARBOPLATINO	20025935-1
Lung	L01XA02	CARBOPLATINO	19912568-1
Lung	L01XA02	CARBOPLATINO	19912569-1
Lung	L01XA02	CARBOPLATINO	19912569-2
Lung	L01XA02	CARBOPLATINO	19935951-1
Lung	L01XA02	CARBOPLATINO	19941538-1
Lung	L01XA02	CARBOPLATINO	19941539-1
Lung	L01XA02	CARBOPLATINO	19951644-1
Lung	L01XA02	CARBOPLATINO	19951644-2
Lung	L01AA01	CICLOFOSFAMIDA	19961041-1
Lung	L01AA01	CICLOFOSFAMIDA	20014778-5
Lung	L01AA01	CICLOFOSFAMIDA	20014778-3
Lung	L01AA01	CICLOFOSFAMIDA	20014778-6
Lung	L01AA01	CICLOFOSFAMIDA	20014778-7
Lung	L01AA01	CICLOFOSFAMIDA	20014778-8
Lung	L01AA01	CICLOFOSFAMIDA	20014778-9
Lung	L01AA01	CICLOFOSFAMIDA	20016861-1
Lung	L01AA01	CICLOFOSFAMIDA	20016861-2
Lung	L01AA01	CICLOFOSFAMIDA	20121471-1
Lung	L01AA01	CICLOFOSFAMIDA	20014778-4
Lung	L01AA01	CICLOFOSFAMIDA	20090481-1
Lung	L01AA01	CICLOFOSFAMIDA	21535-1
Lung	L01AA01	CICLOFOSFAMIDA	19957078-1
Lung	L01AA01	CICLOFOSFAMIDA	20014778-2
Lung	L01AA01	CICLOFOSFAMIDA	20014778-10
Lung	L01AA01	CICLOFOSFAMIDA	20014778-1
Lung	L01AA01	CICLOFOSFAMIDA	20014777-1
Lung	L01AA01	CICLOFOSFAMIDA	19935953-2
Lung	L01AA01	CICLOFOSFAMIDA	19935953-1
Lung	L01AA01	CICLOFOSFAMIDA	20103436-1
Lung	L01AA01	CICLOFOSFAMIDA	21531-2
Lung	L01AA01	CICLOFOSFAMIDA	21531-1
Lung	L01AA01	CICLOFOSFAMIDA	21534-1
Lung	L01XA01	CISPLATINO	19992872-2
Lung	L01XA01	CISPLATINO	19992872-1
Lung	L01XA01	CISPLATINO	19992871-1
Lung	L01XA01	CISPLATINO	20141904-1
Lung	L01XA01	CISPLATINO	19976715-1
Lung	L01XA01	CISPLATINO	20006179-1
Lung	L01XA01	CISPLATINO	20035122-1
Lung	L01XA01	CISPLATINO	20035123-1
Lung	L01XA01	CISPLATINO	19964252-1

Lung	L01XA01	CISPLATINO	19963386-4
Lung	L01XA01	CISPLATINO	19963386-3
Lung	L01XA01	CISPLATINO	19963386-2
Lung	L01XA01	CISPLATINO	19963386-1
Lung	L01XA01	CISPLATINO	20013777-1
Lung	L01XA01	CISPLATINO	20013777-2
Lung	L01XA01	CISPLATINO	20013777-3
Lung	L01XA01	CISPLATINO	19957108-1
Lung	L01XA01	CISPLATINO	20095210-1
Lung	L01XA01	CISPLATINO	44821-1
Lung	L01XA01	CISPLATINO	44823-1
Lung	L01XA01	CISPLATINO	19947842-1
Lung	L01XA01	CISPLATINO	20095072-2
Lung	L01XA01	CISPLATINO	20095072-1
Lung	L01XA01	CISPLATINO	19913980-1
Lung	L01XA01	CISPLATINO	19982072-1
Lung	L01XA01	CISPLATINO	20047564-1
Lung	L01XA01	CISPLATINO	20047565-1
Lung	L01XA01	CISPLATINO	20142068-1
Lung	L01XA01	CISPLATINO	19969323-1
Lung	L01DB01	DOXORUBICINA	19989735-1
Lung	L01DB01	DOXORUBICINA	58174-1
Lung	L01DB01	DOXORUBICINA	20134950-1
Lung	L01DB01	DOXORUBICINA	20118394-1
Lung	L01DB01	DOXORUBICINA	20005641-1
Lung	L01DB01	DOXORUBICINA	20010928-1
Lung	L01DB01	DOXORUBICINA	20011067-1
Lung	L01DB01	DOXORUBICINA	20091053-1
Lung	L01DB01	DOXORUBICINA	20037727-1
Lung	L01DB01	DOXORUBICINA	20037727-2
Lung	L01DB01	DOXORUBICINA	20057168-1
Lung	L01DB01	DOXORUBICINA	20061064-1
Lung	L01DB01	DOXORUBICINA	20061064-2
Lung	L01DB01	DOXORUBICINA	20061064-3
Lung	L01DB01	DOXORUBICINA	20062344-1
Lung	L01DB01	DOXORUBICINA	20043047-2
Lung	L01DB01	DOXORUBICINA	20043047-1
Lung	L01DB01	DOXORUBICINA	20043044-1
Lung	L01DB01	DOXORUBICINA	20037727-3
Lung	L01DB01	DOXORUBICINA	20057171-1
Lung	L01DB01	DOXORUBICINA	1980838-1
Lung	L01DB01	DOXORUBICINA	1980838-2
Lung	L01DB01	DOXORUBICINA	1980838-3
Lung	L01DB01	DOXORUBICINA	1980838-4
Lung	L01DB01	DOXORUBICINA	1980838-5
Lung	L01DB01	DOXORUBICINA	1980838-6
Lung	L01DB01	DOXORUBICINA	20196716-1
Lung	L01DB01	DOXORUBICINA	20196716-2

Lung	L01DB01	DOXORUBICINA	19947745-1
Lung	L01DB01	DOXORUBICINA	19947745-2
Lung	L01DB01	DOXORUBICINA	19947745-3
Lung	L01DB01	DOXORUBICINA	19947745-4
Lung	L01DB01	DOXORUBICINA	19947766-1
Lung	L01DB01	DOXORUBICINA	58178-1
Lung	L01DB01	DOXORUBICINA	38447-1
Lung	L01DB01	DOXORUBICINA	38446-1
Lung	L01DB01	DOXORUBICINA	19953724-1
Lung	L01DB01	DOXORUBICINA	19953724-2
Lung	L01DB01	DOXORUBICINA	19953724-3
Lung	L01DB01	DOXORUBICINA	19967936-1
Lung	L01DB01	DOXORUBICINA	19969115-1
Lung	L01DB01	DOXORUBICINA	19969115-2
Lung	L01DB01	DOXORUBICINA	19969115-3
Lung	L01DB01	DOXORUBICINA	19971373-1
Lung	L01DB01	DOXORUBICINA	19971373-2
Lung	L01DB01	DOXORUBICINA	19971373-3
Lung	L01DB01	DOXORUBICINA	19971373-4
Lung	L01DB01	DOXORUBICINA	20155365-2
Lung	L01DB01	DOXORUBICINA	20155365-1
Lung	L01DB01	DOXORUBICINA	19976712-1
Lung	L01DB01	DOXORUBICINA	19976713-1
Lung	L01XC28	DURVALUMAB	20143749-2
Lung	L01XC28	DURVALUMAB	20143749-1
Lung	L01XC28	DURVALUMAB	20143749-4
Lung	L01XC28	DURVALUMAB	20143749-3
Lung	L01XE03	ERLOTINIB	20146021-2
Lung	L01XE03	ERLOTINIB	20107770-1
Lung	L01XE03	ERLOTINIB	20151436-2
Lung	L01XE03	ERLOTINIB	20116587-1
Lung	L01XE03	ERLOTINIB	20151600-1
Lung	L01XE03	ERLOTINIB	20168010-1
Lung	L01XX34	ERLOTINIB	19961228-1
Lung	L01XE03	ERLOTINIB	20132367-1
Lung	L01XX34	ERLOTINIB	19961230-1
Lung	L01XX34	ERLOTINIB	19961229-1
Lung	L01XE03	ERLOTINIB	20146021-1
Lung	L01CB01	ETOPOSIDO	19952107-1
Lung	L01CB01	ETOPOSIDO	19952107-2
Lung	L01CB01	ETOPOSIDO	19952107-3
Lung	L01CB01	ETOPOSIDO	19982071-1
Lung	L01CB01	ETOPOSIDO	19967166-1
Lung	L01CB01	ETOPOSIDO	19967166-2
Lung	L01CB01	ETOPOSIDO	19949457-1
Lung	L01CB01	ETOPOSIDO	20094214-1
Lung	L01CB01	ETOPOSIDO	20048821-1
Lung	L01CB01	ETOPOSIDO	19998213-1

Lung	L01CB01	ETOPOSIDO	19951646-1
Lung	L01XE02	GEFITINIB	20165119-1
Lung	L01XE02	GEFITINIB	20150402-1
Lung	L01XE02	GEFITINIB	20019022-2
Lung	L01XE02	GEFITINIB	20019022-1
Lung	N02AA01	MORFINA	218192-2
Lung	N02AA01	MORFINA	20013906-1
Lung	N02AA01	MORFINA	20013906-2
Lung	N02AA01	MORFINA	218190-1
Lung	L01XE35	OSIMERTINIB	20135473-1
Lung	L01XE35	OSIMERTINIB	20135473-2
Lung	L01XE35	OSIMERTINIB	20118029-2
Lung	L01XE35	OSIMERTINIB	20118029-1
Lung	L01CD01	PACLITAXEL	20061216-1
Lung	L01CD01	PACLITAXEL	20064116-1
Lung	L01CD01	PACLITAXEL	20064116-2
Lung	L01CD01	PACLITAXEL	20071691-1
Lung	L01CD01	PACLITAXEL	20071695-1
Lung	L01CD01	PACLITAXEL	20071697-1
Lung	L01CD01	PACLITAXEL	20107415-1
Lung	L01CD01	PACLITAXEL	20107420-1
Lung	L01CD01	PACLITAXEL	20135292-1
Lung	L01CD01	PACLITAXEL	20137724-1
Lung	L01CD01	PACLITAXEL	20137724-2
Lung	L01CD01	PACLITAXEL	20137724-3
Lung	L01CD01	PACLITAXEL	20137724-4
Lung	L01CD01	PACLITAXEL	20148763-1
Lung	L01CD01	PACLITAXEL	55979-8
Lung	L01CD01	PACLITAXEL	55979-7
Lung	L01CD01	PACLITAXEL	20192030-1
Lung	L01CD01	PACLITAXEL	55979-1
Lung	L01CD01	PACLITAXEL	55979-6
Lung	L01CD01	PACLITAXEL	55979-5
Lung	L01CD01	PACLITAXEL	55979-4
Lung	L01CD01	PACLITAXEL	55979-2
Lung	L01CD01	PACLITAXEL	55979-3
Lung	L01CD01	PACLITAXEL	222085-1
Lung	L01CD01	PACLITAXEL	19952097-2
Lung	L01CD01	PACLITAXEL	55979-9
Lung	L01CD01	PACLITAXEL	19912566-1
Lung	L01CD01	PACLITAXEL	19915547-1
Lung	L01CD01	PACLITAXEL	19915547-2
Lung	L01CD01	PACLITAXEL	19915547-3
Lung	L01CD01	PACLITAXEL	19937677-1
Lung	L01CD01	PACLITAXEL	19937677-2
Lung	L01CD01	PACLITAXEL	19937677-3
Lung	L01CD01	PACLITAXEL	19946485-1
Lung	L01CD01	PACLITAXEL	19946485-2

Lung	L01CD01	PACLITAXEL	19946485-3
Lung	L01CD01	PACLITAXEL	19946485-4
Lung	L01CD01	PACLITAXEL	19946485-5
Lung	L01CD01	PACLITAXEL	19946485-6
Lung	L01CD01	PACLITAXEL	19952097-1
Lung	L01CD01	PACLITAXEL	19952097-3
Lung	L01CD01	PACLITAXEL	19952097-4
Lung	L01CD01	PACLITAXEL	19952097-5
Lung	L01CD01	PACLITAXEL	19952097-6
Lung	L01CD01	PACLITAXEL	19952097-7
Lung	L01CD01	PACLITAXEL	19952097-8
Lung	L01CD01	PACLITAXEL	19952110-1
Lung	L01CD01	PACLITAXEL	19957354-1
Lung	L01CD01	PACLITAXEL	19960065-1
Lung	L01CD01	PACLITAXEL	19963086-1
Lung	L01CD01	PACLITAXEL	19967319-1
Lung	L01CD01	PACLITAXEL	19968458-1
Lung	L01CD01	PACLITAXEL	19969254-1
Lung	L01CD01	PACLITAXEL	19969254-2
Lung	L01CD01	PACLITAXEL	19969254-3
Lung	L01CD01	PACLITAXEL	19973177-1
Lung	L01CD01	PACLITAXEL	19973178-1
Lung	L01CD01	PACLITAXEL	19973179-1
Lung	L01CD01	PACLITAXEL	19973180-1
Lung	L01CD01	PACLITAXEL	19976351-1
Lung	L01CD01	PACLITAXEL	19976351-2
Lung	L01CD01	PACLITAXEL	19976351-3
Lung	L01CD01	PACLITAXEL	19976352-1
Lung	L01CD01	PACLITAXEL	19976352-2
Lung	L01CD01	PACLITAXEL	19976352-3
Lung	L01CD01	PACLITAXEL	19976518-1
Lung	L01CD01	PACLITAXEL	19976518-2
Lung	L01CD01	PACLITAXEL	19976519-1
Lung	L01CD01	PACLITAXEL	19976519-2
Lung	L01CD01	PACLITAXEL	19979611-1
Lung	L01CD01	PACLITAXEL	19980164-1
Lung	L01CD01	PACLITAXEL	19989803-1
Lung	L01CD01	PACLITAXEL	19989803-2
Lung	L01CD01	PACLITAXEL	19989803-3
Lung	L01CD01	PACLITAXEL	19989803-4
Lung	L01CD01	PACLITAXEL	19989803-5
Lung	L01CD01	PACLITAXEL	19989803-6
Lung	L01CD01	PACLITAXEL	19991105-1
Lung	L01CD01	PACLITAXEL	19991105-2
Lung	L01CD01	PACLITAXEL	19991106-1
Lung	L01CD01	PACLITAXEL	19992001-1
Lung	L01CD01	PACLITAXEL	19992001-2
Lung	L01CD01	PACLITAXEL	19992001-3

Lung	L01CD01	PACLITAXEL	19992001-4
Lung	L01CD01	PACLITAXEL	20057021-1
Lung	L01CD01	PACLITAXEL	20056002-1
Lung	L01CD01	PACLITAXEL	20056001-2
Lung	L01CD01	PACLITAXEL	20055999-1
Lung	L01CD01	PACLITAXEL	20055740-1
Lung	L01CD01	PACLITAXEL	20055486-1
Lung	L01CD01	PACLITAXEL	20047567-1
Lung	L01CD01	PACLITAXEL	20047566-1
Lung	L01CD01	PACLITAXEL	20031267-1
Lung	L01CD01	PACLITAXEL	20029992-1
Lung	L01CD01	PACLITAXEL	20029991-1
Lung	L01CD01	PACLITAXEL	20029990-1
Lung	L01CD01	PACLITAXEL	20022187-1
Lung	L01CD01	PACLITAXEL	20022186-1
Lung	L01CD01	PACLITAXEL	20020276-3
Lung	L01CD01	PACLITAXEL	20020276-2
Lung	L01CD01	PACLITAXEL	20020276-1
Lung	L01CD01	PACLITAXEL	20018484-1
Lung	L01CD01	PACLITAXEL	20018483-1
Lung	L01CD01	PACLITAXEL	20018482-1
Lung	L01CD01	PACLITAXEL	20014772-1
Lung	L01CD01	PACLITAXEL	20014726-1
Lung	L01CD01	PACLITAXEL	20012772-1
Lung	L01CD01	PACLITAXEL	20012769-1
Lung	L01CD01	PACLITAXEL	20007285-1
Lung	L01CD01	PACLITAXEL	19992001-7
Lung	L01CD01	PACLITAXEL	19992001-6
Lung	L01CD01	PACLITAXEL	19992001-5
Lung	L01CD01	PACLITAXEL	20057023-1
Lung	L01XC18	PEMBROLIZUMAB	20085509-1
Lung	L01BA04	PEMETREXED	20199958-1
Lung	L01BA04	PEMETREXED	20062095-1
Lung	L01BA04	PEMETREXED	20055746-1
Lung	L01BA04	PEMETREXED	20122255-1
Lung	L01BA04	PEMETREXED	20064421-1
Lung	L01BA04	PEMETREXED	20126300-1
Lung	L01BA04	PEMETREXED	20054874-1
Lung	L01BA04	PEMETREXED	20052990-1
Lung	L01BA04	PEMETREXED	20067757-1
Lung	L01BA04	PEMETREXED	20067757-2
Lung	L01BA04	PEMETREXED	20067757-3
Lung	L01BA04	PEMETREXED	20127801-1
Lung	L01BA04	PEMETREXED	20130441-1
Lung	L01BA04	PEMETREXED	19951013-2
Lung	L01BA04	PEMETREXED	20071995-1
Lung	L01BA04	PEMETREXED	20072986-1
Lung	L01BA04	PEMETREXED	20073021-1

Lung	L01BA04	PEMETREXED	20077800-1
Lung	L01BA04	PEMETREXED	20083481-1
Lung	L01BA04	PEMETREXED	20169034-1
Lung	L01BA04	PEMETREXED	19951013-1
Lung	L01BA04	PEMETREXED	20199958-2
Lung	L01BA04	PEMETREXED	20120667-2
Lung	L01BA04	PEMETREXED	20120667-1
Lung	L01BA04	PEMETREXED	20120416-2
Lung	L01BA04	PEMETREXED	20120416-1
Lung	L01BA04	PEMETREXED	20108592-1
Lung	L01BA04	PEMETREXED	20099991-1
Lung	L01BA04	PEMETREXED	20099989-1
Lung	L01BA04	PEMETREXED	20097112-3
Lung	L01BA04	PEMETREXED	20097112-2
Lung	L01BA04	PEMETREXED	20097112-1
Lung	L01BA04	PEMETREXED	20096415-4
Lung	L01BA04	PEMETREXED	20096415-3
Lung	L01BA04	PEMETREXED	20096415-2
Lung	L01BA04	PEMETREXED	20096415-1
Lung	L01BA04	PEMETREXED	20142739-1
Lung	L01BA04	PEMETREXED	20142738-1
Lung	L01XX17	TOPOTECAN	20010613-1
Lung	L01XX17	TOPOTECAN	19986863-1
Lung	L01XX17	TOPOTECAN	230185-1
Lung	L01XX17	TOPOTECAN	230185-3
Lung	L01XX17	TOPOTECAN	230185-2
Lung	L01CA02	VINCRISTINA	19949459-1
Lung	L01CA02	VINCRISTINA	19956730-1
Lung	L01CA02	VINCRISTINA	19913977-1
Lung	L01CA02	VINCRISTINA	19935952-2
Lung	L01CA02	VINCRISTINA	19935952-1
Lung	L01CA02	VINCRISTINA	19952092-2
Lung	L01CA02	VINCRISTINA	20006838-3
Lung	L01CA02	VINCRISTINA	19952092-3
Lung	L01CA02	VINCRISTINA	20006838-1
Lung	L01CA02	VINCRISTINA	20097927-3
Lung	L01CA02	VINCRISTINA	20097927-2
Lung	L01CA02	VINCRISTINA	20097927-1
Lung	L01CA02	VINCRISTINA	20006838-2
Lung	L01CA02	VINCRISTINA	19952092-1
Lung	L01CA02	VINCRISTINA	58188-1
Prostate	L02BX03	ABIRATERONA	20120942-4
Prostate	L02BX03	ABIRATERONA	20120942-5
Prostate	L02BX03	ABIRATERONA	20128309-1
Prostate	L02BX03	ABIRATERONA	20128309-2
Prostate	L02BX03	ABIRATERONA	20128309-3
Prostate	L02BX03	ABIRATERONA	20137931-1
Prostate	L02BX03	ABIRATERONA	20036026-1

Prostate	L02BX03	ABIRATERONA	20036026-2
Prostate	L02BX03	ABIRATERONA	20149212-1
Prostate	L02BX03	ABIRATERONA	20148436-2
Prostate	L02BX03	ABIRATERONA	20148436-1
Prostate	L02BX03	ABIRATERONA	20146583-2
Prostate	L02BX03	ABIRATERONA	20146583-1
Prostate	L02BX03	ABIRATERONA	20105885-2
Prostate	L02BX03	ABIRATERONA	20105885-1
Prostate	L02BX03	ABIRATERONA	20115120-1
Prostate	L02BX03	ABIRATERONA	20116619-1
Prostate	L02BX03	ABIRATERONA	20116619-2
Prostate	L02BX03	ABIRATERONA	20120942-1
Prostate	L02BX03	ABIRATERONA	20120942-3
Prostate	L02BX03	ABIRATERONA	20120942-2
Prostate	L02BB05	APALUTAMIDA	20137853-1
Prostate	L02BB05	APALUTAMIDA	20137853-2
Prostate	L01CD04	CABAZITAXEL	20104913-1
Prostate	L01CD04	CABAZITAXEL	20104913-4
Prostate	L01CD04	CABAZITAXEL	20132251-1
Prostate	L01CD04	CABAZITAXEL	20104913-3
Prostate	L01CD04	CABAZITAXEL	20039453-1
Prostate	L01CD02	DOCETAXEL	20022245-1
Prostate	L01CD02	DOCETAXEL	20064497-1
Prostate	L01CD02	DOCETAXEL	20064460-3
Prostate	L01CD02	DOCETAXEL	20064460-2
Prostate	L01CD02	DOCETAXEL	20064460-1
Prostate	L01CD02	DOCETAXEL	20064412-3
Prostate	L01CD02	DOCETAXEL	20064412-2
Prostate	L01CD02	DOCETAXEL	20064412-1
Prostate	L01CD02	DOCETAXEL	20057737-1
Prostate	L01CD02	DOCETAXEL	20057736-1
Prostate	L01CD02	DOCETAXEL	20049388-1
Prostate	L01CD02	DOCETAXEL	20047646-1
Prostate	L01CD02	DOCETAXEL	20047644-1
Prostate	L01CD02	DOCETAXEL	20045960-1
Prostate	L01CD02	DOCETAXEL	20045955-1
Prostate	L01CD02	DOCETAXEL	20111935-2
Prostate	L01CD02	DOCETAXEL	20111936-1
Prostate	L01CD02	DOCETAXEL	20028651-3
Prostate	L01CD02	DOCETAXEL	20028651-2
Prostate	L01CD02	DOCETAXEL	20028651-1
Prostate	L01CD02	DOCETAXEL	20024849-3
Prostate	L01CD02	DOCETAXEL	20024849-2
Prostate	L01CD02	DOCETAXEL	20024849-1
Prostate	L01CD02	DOCETAXEL	20022245-3
Prostate	L01CD02	DOCETAXEL	20022245-2
Prostate	L01CD02	DOCETAXEL	20064497-2
Prostate	L01CD02	DOCETAXEL	20064497-3

Prostate	L01CD02	DOCETAXEL	20064497-4
Prostate	L01CD02	DOCETAXEL	20064497-5
Prostate	L01CD02	DOCETAXEL	20064499-1
Prostate	L01CD02	DOCETAXEL	20067008-1
Prostate	L01CD02	DOCETAXEL	19969253-1
Prostate	L01CD02	DOCETAXEL	230189-2
Prostate	L01CD02	DOCETAXEL	19969253-3
Prostate	L01CD02	DOCETAXEL	19969253-2
Prostate	L01CD02	DOCETAXEL	230187-1
Prostate	L01CD02	DOCETAXEL	19974847-1
Prostate	L01CD02	DOCETAXEL	19969842-3
Prostate	L01CD02	DOCETAXEL	19969842-2
Prostate	L01CD02	DOCETAXEL	19969842-1
Prostate	L01CD02	DOCETAXEL	19969253-5
Prostate	L01CD02	DOCETAXEL	19969253-4
Prostate	L01CD02	DOCETAXEL	19996764-1
Prostate	L01CD02	DOCETAXEL	19996765-1
Prostate	L01CD02	DOCETAXEL	20007286-1
Prostate	L01CD02	DOCETAXEL	20111935-1
Prostate	L01CD02	DOCETAXEL	20113692-1
Prostate	L01CD02	DOCETAXEL	20113688-1
Prostate	L01CD02	DOCETAXEL	20111936-2
Prostate	L01CD02	DOCETAXEL	20020736-1
Prostate	L01CD02	DOCETAXEL	20020736-3
Prostate	L01CD02	DOCETAXEL	20020736-2
Prostate	L02BB04	ENZALUTAMIDA	20198992-2
Prostate	L02BB04	ENZALUTAMIDA	20198992-1
Prostate	L02BB04	ENZALUTAMIDA	20196062-1
Prostate	L02BB04	ENZALUTAMIDA	20195068-2
Prostate	L02BB04	ENZALUTAMIDA	20195068-1
Prostate	L02AE03	GOSERELINA	201182-3
Prostate	L02AE03	GOSERELINA	201182-4
Prostate	L02AE03	GOSERELINA	47155-3
Prostate	L02AE03	GOSERELINA	47155-4
Prostate	L02AE03	GOSERELINA	47155-7
Prostate	L02AE03	GOSERELINA	47155-6
Prostate	L02AE03	GOSERELINA	47155-5
Prostate	L02AE03	GOSERELINA	47155-2
Prostate	L02AE03	GOSERELINA	47155-1
Prostate	L02AE03	GOSERELINA	201182-1
Prostate	L02AE03	GOSERELINA	201182-5
Prostate	L02AE03	GOSERELINA	201182-6
Prostate	L02AE03	GOSERELINA	201182-2
Prostate	N02AA01	MORFINA	218192-2
Prostate	N02AA01	MORFINA	20013906-1
Prostate	N02AA01	MORFINA	20013906-2
Prostate	N02AA01	MORFINA	218190-1
Prostate	L01XX46	OLAPARIB	20124752-3

Prostate	L01XX46	OLAPARIB	20124752-1
Prostate	L01XX46	OLAPARIB	20142204-4
Prostate	L01XX46	OLAPARIB	20124752-4
Prostate	L01XX46	OLAPARIB	20142204-1
Prostate	L01XX46	OLAPARIB	20142204-2
Prostate	L01XX46	OLAPARIB	20142204-3
Prostate	L01XX46	OLAPARIB	20124752-2
Prostate	H02AB06	PREDNISOLONA	20107496-5
Prostate	H02AB06	PREDNISOLONA	20107499-1
Prostate	H02AB06	PREDNISOLONA	20107499-2
Prostate	H02AB06	PREDNISOLONA	20118914-1
Prostate	H02AB06	PREDNISOLONA	20118914-2
Prostate	A07EA01	PREDNISOLONA	20128975-1
Prostate	A07EA01	PREDNISOLONA	20128975-2
Prostate	A07EA01	PREDNISOLONA	20128975-3
Prostate	A07EA01	PREDNISOLONA	20128975-4
Prostate	A07EA01	PREDNISOLONA	20128975-5
Prostate	H02AB06	PREDNISOLONA	20179194-1
Prostate	H02AB06	PREDNISOLONA	20179194-10
Prostate	H02AB06	PREDNISOLONA	20179194-2
Prostate	H02AB06	PREDNISOLONA	20179194-3
Prostate	H02AB06	PREDNISOLONA	20179194-4
Prostate	H02AB06	PREDNISOLONA	20179194-5
Prostate	H02AB06	PREDNISOLONA	20179194-6
Prostate	H02AB06	PREDNISOLONA	20179194-7
Prostate	H02AB06	PREDNISOLONA	20179194-8
Prostate	H02AB06	PREDNISOLONA	20179194-9
Prostate	H02AB06	PREDNISOLONA	20179246-1
Prostate	H02AB06	PREDNISOLONA	20179246-10
Prostate	H02AB06	PREDNISOLONA	20179246-11
Prostate	H02AB06	PREDNISOLONA	20179246-12
Prostate	H02AB06	PREDNISOLONA	20179246-2
Prostate	H02AB06	PREDNISOLONA	20179246-3
Prostate	H02AB06	PREDNISOLONA	20179246-4
Prostate	H02AB06	PREDNISOLONA	20179246-5
Prostate	H02AB06	PREDNISOLONA	20179246-6
Prostate	H02AB06	PREDNISOLONA	20179246-7
Prostate	H02AB06	PREDNISOLONA	20179246-8
Prostate	H02AB06	PREDNISOLONA	20216528-1
Prostate	H02AB06	PREDNISOLONA	20216528-2
Prostate	H02AB06	PREDNISOLONA	20600-1
Prostate	H02AB06	PREDNISOLONA	20600-2
Prostate	H02AB06	PREDNISOLONA	20600-3
Prostate	H02AB06	PREDNISOLONA	20600-4
Prostate	H02AB06	PREDNISOLONA	20600-5
Prostate	H02AB06	PREDNISOLONA	20600-6
Prostate	H02AB06	PREDNISOLONA	20600-7
Prostate	S01BA04	PREDNISOLONA	230341-1

Prostate	S01BA04	PREDNISOLONA	230341-2
Prostate	H02AB06	PREDNISOLONA	54371-1
Prostate	H02AB06	PREDNISOLONA	54371-2
Prostate	H02AB06	PREDNISOLONA	1984100-3
Prostate	H02AB06	PREDNISOLONA	1984100-4
Prostate	H02AB06	PREDNISOLONA	1984100-5
Prostate	H02AB06	PREDNISOLONA	1984100-2
Prostate	H02AB06	PREDNISOLONA	1984100-1
Prostate	H02AB06	PREDNISOLONA	20179246-9
Prostate	S01BA04	PREDNISOLONA	1984629-2
Prostate	S01BA04	PREDNISOLONA	1984629-1
Prostate	S01BA04	PREDNISOLONA	19912602-1
Prostate	S01BA04	PREDNISOLONA	19912602-2
Prostate	S01BA04	PREDNISOLONA	19912759-1
Prostate	S01BA04	PREDNISOLONA	19912759-3
Prostate	H02AB06	PREDNISOLONA	19914128-1
Prostate	H02AB06	PREDNISOLONA	19914128-2
Prostate	H02AB06	PREDNISOLONA	19914128-3
Prostate	H02AB06	PREDNISOLONA	19914128-4
Prostate	S01BA04	PREDNISOLONA	19954550-1
Prostate	S01BA04	PREDNISOLONA	19981546-1
Prostate	S01BA04	PREDNISOLONA	19981546-2
Prostate	S01BA04	PREDNISOLONA	19982087-1
Prostate	S01BA04	PREDNISOLONA	19982087-2
Prostate	S01BA04	PREDNISOLONA	19982087-3
Prostate	S01BA04	PREDNISOLONA	19982087-4
Prostate	S01BA04	PREDNISOLONA	19982087-5
Prostate	S01BA04	PREDNISOLONA	19982087-6
Prostate	S01BA04	PREDNISOLONA	19982087-7
Prostate	H02AB06	PREDNISOLONA	19990437-1
Prostate	S01BA04	PREDNISOLONA	20009756-1
Prostate	S01BA04	PREDNISOLONA	20009756-2
Prostate	H02AB06	PREDNISOLONA	20011084-1
Prostate	H02AB06	PREDNISOLONA	20011084-2
Prostate	H02AB06	PREDNISOLONA	20011084-3
Prostate	H02AB06	PREDNISOLONA	20011084-4
Prostate	H02AB06	PREDNISOLONA	20011084-5
Prostate	H02AB06	PREDNISOLONA	20011084-6
Prostate	H02AB06	PREDNISOLONA	20011084-7
Prostate	H02AB06	PREDNISOLONA	20017682-1
Prostate	H02AB06	PREDNISOLONA	20017682-2
Prostate	H02AB06	PREDNISOLONA	20017682-3
Prostate	H02AB06	PREDNISOLONA	20017682-4
Prostate	H02AB06	PREDNISOLONA	20017682-5
Prostate	H02AB06	PREDNISOLONA	20107496-3
Prostate	H02AB06	PREDNISOLONA	20107496-2
Prostate	H02AB06	PREDNISOLONA	20107496-1
Prostate	H02AB06	PREDNISOLONA	20103747-8

Prostate	H02AB06	PREDNISOLONA	20103747-7
Prostate	H02AB06	PREDNISOLONA	20103747-6
Prostate	H02AB06	PREDNISOLONA	20103747-5
Prostate	H02AB06	PREDNISOLONA	20103747-4
Prostate	H02AB06	PREDNISOLONA	20103747-3
Prostate	H02AB06	PREDNISOLONA	20103747-2
Prostate	H02AB06	PREDNISOLONA	20103747-1
Prostate	H02AB06	PREDNISOLONA	20103606-9
Prostate	H02AB06	PREDNISOLONA	20103606-8
Prostate	H02AB06	PREDNISOLONA	20103606-7
Prostate	H02AB06	PREDNISOLONA	20103606-6
Prostate	H02AB06	PREDNISOLONA	20103606-5
Prostate	H02AB06	PREDNISOLONA	20103606-4
Prostate	H02AB06	PREDNISOLONA	20103606-3
Prostate	H02AB06	PREDNISOLONA	20103606-2
Prostate	H02AB06	PREDNISOLONA	20103606-10
Prostate	H02AB06	PREDNISOLONA	20103606-1
Prostate	H02AB06	PREDNISOLONA	20100049-2
Prostate	H02AB06	PREDNISOLONA	20100049-1
Prostate	S01BA04	PREDNISOLONA	20099657-1
Prostate	S01BA04	PREDNISOLONA	20093741-4
Prostate	S01BA04	PREDNISOLONA	20093741-3
Prostate	S01BA04	PREDNISOLONA	20093741-2
Prostate	S01BA04	PREDNISOLONA	20093741-1
Prostate	H02AB06	PREDNISOLONA	20084373-8
Prostate	H02AB06	PREDNISOLONA	20084373-7
Prostate	H02AB06	PREDNISOLONA	20084373-6
Prostate	H02AB06	PREDNISOLONA	20084373-5
Prostate	H02AB06	PREDNISOLONA	20084373-4
Prostate	H02AB06	PREDNISOLONA	20084373-3
Prostate	H02AB06	PREDNISOLONA	20084373-2
Prostate	H02AB06	PREDNISOLONA	20084373-1
Prostate	S01BA04	PREDNISOLONA	20074441-3
Prostate	S01BA04	PREDNISOLONA	20074441-2
Prostate	S01BA04	PREDNISOLONA	20074441-1
Prostate	H02AB06	PREDNISOLONA	20071241-9
Prostate	H02AB06	PREDNISOLONA	20071241-8
Prostate	H02AB06	PREDNISOLONA	20071241-7
Prostate	H02AB06	PREDNISOLONA	20071241-6
Prostate	H02AB06	PREDNISOLONA	20071241-5
Prostate	H02AB06	PREDNISOLONA	20071241-4
Prostate	H02AB06	PREDNISOLONA	20071241-3
Prostate	H02AB06	PREDNISOLONA	20071241-2
Prostate	H02AB06	PREDNISOLONA	20071241-13
Prostate	H02AB06	PREDNISOLONA	20071241-12
Prostate	H02AB06	PREDNISOLONA	20071241-11
Prostate	H02AB06	PREDNISOLONA	20071241-10
Prostate	H02AB06	PREDNISOLONA	20071241-1

Prostate	S01BA04	PREDNISOLONA	20058767-1
Prostate	S01BA04	PREDNISOLONA	20040316-3
Prostate	S01BA04	PREDNISOLONA	20040316-2
Prostate	S01BA04	PREDNISOLONA	20040316-1
Prostate	S01BA04	PREDNISOLONA	20025559-1
Prostate	H02AB06	PREDNISOLONA	20017682-9
Prostate	H02AB06	PREDNISOLONA	20017682-8
Prostate	H02AB06	PREDNISOLONA	20017682-7
Prostate	H02AB06	PREDNISOLONA	20017682-6
Prostate	H02AB06	PREDNISOLONA	20107496-4
Prostate	H02AB07	PREDNISONA	19906237-3
Prostate	H02AB07	PREDNISONA	19906237-1
Prostate	H02AB07	PREDNISONA	26006-1
Prostate	H02AB07	PREDNISONA	19906237-2
Prostate	H02AB07	PREDNISONA	19906237-5
Prostate	H02AB07	PREDNISONA	19906237-4
Stomach	V03AF03	CALCIO FOLINATO	48266-5
Stomach	V03AF03	CALCIO FOLINATO	51488-1
Stomach	V03AF03	CALCIO FOLINATO	48266-4
Stomach	V03AF03	CALCIO FOLINATO	48266-6
Stomach	V03AF03	CALCIO FOLINATO	51488-2
Stomach	V03AF03	CALCIO FOLINATO	48266-2
Stomach	V03AF03	CALCIO FOLINATO	48266-3
Stomach	V03AF03	CALCIO FOLINATO	19989251-1
Stomach	V03AF03	CALCIO FOLINATO	19989251-2
Stomach	V03AF03	CALCIO FOLINATO	19989251-3
Stomach	V03AF03	CALCIO FOLINATO	19989251-5
Stomach	V03AF03	CALCIO FOLINATO	19989251-4
Stomach	V03AF03	CALCIO FOLINATO	51488-3
Stomach	V03AF03	CALCIO FOLINATO	20139847-1
Stomach	V03AF03	CALCIO FOLINATO	19952111-1
Stomach	V03AF03	CALCIO FOLINATO	20139379-3
Stomach	V03AF03	CALCIO FOLINATO	20139379-2
Stomach	V03AF03	CALCIO FOLINATO	19953721-1
Stomach	V03AF03	CALCIO FOLINATO	19953722-1
Stomach	V03AF03	CALCIO FOLINATO	19930613-1
Stomach	V03AF03	CALCIO FOLINATO	20139379-1
Stomach	V03AF03	CALCIO FOLINATO	20138364-1
Stomach	V03AF03	CALCIO FOLINATO	48266-1
Stomach	V03AF03	CALCIO FOLINATO	20213225-1
Stomach	V03AF03	CALCIO FOLINATO	201934-1
Stomach	V03AF03	CALCIO FOLINATO	20006242-1
Stomach	V03AF03	CALCIO FOLINATO	19940090-1
Stomach	V03AF03	CALCIO FOLINATO	20139847-2
Stomach	L01BC06	CAPECITABINA	20132906-1
Stomach	L01BC06	CAPECITABINA	20132905-5
Stomach	L01BC06	CAPECITABINA	20132905-4
Stomach	L01BC06	CAPECITABINA	20132905-3

Stomach	L01BC06	CAPECITABINA	20132905-2
Stomach	L01BC06	CAPECITABINA	20132905-1
Stomach	L01BC06	CAPECITABINA	20116817-9
Stomach	L01BC06	CAPECITABINA	20116817-8
Stomach	L01BC06	CAPECITABINA	20116817-7
Stomach	L01BC06	CAPECITABINA	20116817-6
Stomach	L01BC06	CAPECITABINA	20116817-4
Stomach	L01BC06	CAPECITABINA	20116817-3
Stomach	L01BC06	CAPECITABINA	20116817-2
Stomach	L01BC06	CAPECITABINA	20116817-10
Stomach	L01BC06	CAPECITABINA	20116817-1
Stomach	L01BC06	CAPECITABINA	20111735-1
Stomach	L01BC06	CAPECITABINA	20104404-1
Stomach	L01BC06	CAPECITABINA	20060323-9
Stomach	L01BC06	CAPECITABINA	20060323-8
Stomach	L01BC06	CAPECITABINA	20060323-7
Stomach	L01BC06	CAPECITABINA	20060323-6
Stomach	L01BC06	CAPECITABINA	20060323-5
Stomach	L01BC06	CAPECITABINA	20060323-4
Stomach	L01BC06	CAPECITABINA	20060323-3
Stomach	L01BC06	CAPECITABINA	20060323-2
Stomach	L01BC06	CAPECITABINA	20060323-15
Stomach	L01BC06	CAPECITABINA	20060323-14
Stomach	L01BC06	CAPECITABINA	20060323-13
Stomach	L01BC06	CAPECITABINA	20060323-12
Stomach	L01BC06	CAPECITABINA	20060323-11
Stomach	L01BC06	CAPECITABINA	20060323-10
Stomach	L01BC06	CAPECITABINA	20060323-1
Stomach	L01BC06	CAPECITABINA	20053629-1
Stomach	L01BC06	CAPECITABINA	20116817-5
Stomach	L01BC06	CAPECITABINA	229745-1
Stomach	L01BC06	CAPECITABINA	20160260-4
Stomach	L01BC06	CAPECITABINA	20160260-3
Stomach	L01BC06	CAPECITABINA	20160260-2
Stomach	L01BC06	CAPECITABINA	20160260-1
Stomach	L01BC06	CAPECITABINA	20155908-9
Stomach	L01BC06	CAPECITABINA	20155908-8
Stomach	L01BC06	CAPECITABINA	20155908-7
Stomach	L01BC06	CAPECITABINA	20155908-6
Stomach	L01BC06	CAPECITABINA	20155908-5
Stomach	L01BC06	CAPECITABINA	20155908-4
Stomach	L01BC06	CAPECITABINA	20155908-3
Stomach	L01BC06	CAPECITABINA	20155908-2
Stomach	L01BC06	CAPECITABINA	20155908-1
Stomach	L01BC06	CAPECITABINA	20152744-1
Stomach	L01BC06	CAPECITABINA	20146928-1
Stomach	L01BC06	CAPECITABINA	20132906-4
Stomach	L01BC06	CAPECITABINA	20132906-3

Stomach	L01BC06	CAPECITABINA	20132906-2
Stomach	L01XA01	CISPLATINO	20095072-2
Stomach	L01XA01	CISPLATINO	19963386-1
Stomach	L01XA01	CISPLATINO	44821-1
Stomach	L01XA01	CISPLATINO	19964252-1
Stomach	L01XA01	CISPLATINO	44823-1
Stomach	L01XA01	CISPLATINO	19913980-1
Stomach	L01XA01	CISPLATINO	19957108-1
Stomach	L01XA01	CISPLATINO	20141904-1
Stomach	L01XA01	CISPLATINO	19992872-2
Stomach	L01XA01	CISPLATINO	19992872-1
Stomach	L01XA01	CISPLATINO	19992871-1
Stomach	L01XA01	CISPLATINO	20035122-1
Stomach	L01XA01	CISPLATINO	20035123-1
Stomach	L01XA01	CISPLATINO	20013777-3
Stomach	L01XA01	CISPLATINO	20013777-1
Stomach	L01XA01	CISPLATINO	19947842-1
Stomach	L01XA01	CISPLATINO	19982072-1
Stomach	L01XA01	CISPLATINO	19976715-1
Stomach	L01XA01	CISPLATINO	20047564-1
Stomach	L01XA01	CISPLATINO	20047565-1
Stomach	L01XA01	CISPLATINO	20095072-1
Stomach	L01XA01	CISPLATINO	19963386-2
Stomach	L01XA01	CISPLATINO	19963386-3
Stomach	L01XA01	CISPLATINO	19963386-4
Stomach	L01XA01	CISPLATINO	20013777-2
Stomach	L01XA01	CISPLATINO	20142068-1
Stomach	L01XA01	CISPLATINO	19969323-1
Stomach	L01XA01	CISPLATINO	20095210-1
Stomach	L01XA01	CISPLATINO	20006179-1
Stomach	L01BC08	DECITABINA	20192584-1
Stomach	L01BC08	DECITABINA	20114014-1
Stomach	L01BC08	DECITABINA	19991325-2
Stomach	L01BC08	DECITABINA	19991325-1
Stomach	L01BC08	DECITABINA	20148281-1
Stomach	L01BC08	DECITABINA	20097119-1
Stomach	L01BC08	DECITABINA	20096772-1
Stomach	L01BC08	DECITABINA	20097119-2
Stomach	L01CD02	DOCETAXEL	20028651-3
Stomach	L01CD02	DOCETAXEL	20067008-1
Stomach	L01CD02	DOCETAXEL	20064499-1
Stomach	L01CD02	DOCETAXEL	20022245-3
Stomach	L01CD02	DOCETAXEL	19996764-1
Stomach	L01CD02	DOCETAXEL	19996765-1
Stomach	L01CD02	DOCETAXEL	19969842-1
Stomach	L01CD02	DOCETAXEL	19969842-2
Stomach	L01CD02	DOCETAXEL	19969842-3
Stomach	L01CD02	DOCETAXEL	230189-2

Stomach	L01CD02	DOCETAXEL	20064497-5
Stomach	L01CD02	DOCETAXEL	20028651-1
Stomach	L01CD02	DOCETAXEL	20024849-1
Stomach	L01CD02	DOCETAXEL	19969253-1
Stomach	L01CD02	DOCETAXEL	19969253-2
Stomach	L01CD02	DOCETAXEL	19969253-3
Stomach	L01CD02	DOCETAXEL	19969253-4
Stomach	L01CD02	DOCETAXEL	20049388-1
Stomach	L01CD02	DOCETAXEL	20022245-2
Stomach	L01CD02	DOCETAXEL	20057736-1
Stomach	L01CD02	DOCETAXEL	20007286-1
Stomach	L01CD02	DOCETAXEL	20064497-4
Stomach	L01CD02	DOCETAXEL	20113692-1
Stomach	L01CD02	DOCETAXEL	20022245-1
Stomach	L01CD02	DOCETAXEL	20028651-2
Stomach	L01CD02	DOCETAXEL	19974847-1
Stomach	L01CD02	DOCETAXEL	20064497-1
Stomach	L01CD02	DOCETAXEL	20047646-1
Stomach	L01CD02	DOCETAXEL	20020736-2
Stomach	L01CD02	DOCETAXEL	20020736-1
Stomach	L01CD02	DOCETAXEL	20113688-1
Stomach	L01CD02	DOCETAXEL	20047644-1
Stomach	L01CD02	DOCETAXEL	20064497-3
Stomach	L01CD02	DOCETAXEL	20111936-2
Stomach	L01CD02	DOCETAXEL	20057737-1
Stomach	L01CD02	DOCETAXEL	20024849-3
Stomach	L01CD02	DOCETAXEL	230187-1
Stomach	L01CD02	DOCETAXEL	20064497-2
Stomach	L01CD02	DOCETAXEL	20024849-2
Stomach	L01CD02	DOCETAXEL	20111936-1
Stomach	L01CD02	DOCETAXEL	20064412-1
Stomach	L01CD02	DOCETAXEL	20064412-2
Stomach	L01CD02	DOCETAXEL	20064412-3
Stomach	L01CD02	DOCETAXEL	20064460-1
Stomach	L01CD02	DOCETAXEL	20064460-2
Stomach	L01CD02	DOCETAXEL	19969253-5
Stomach	L01CD02	DOCETAXEL	20111935-2
Stomach	L01CD02	DOCETAXEL	20045955-1
Stomach	L01CD02	DOCETAXEL	20045960-1
Stomach	L01CD02	DOCETAXEL	20111935-1
Stomach	L01CD02	DOCETAXEL	20064460-3
Stomach	L01CD02	DOCETAXEL	20020736-3
Stomach	L01BC02	FLUOROURACILO	20043050-1
Stomach	L01BC02	FLUOROURACILO	20043050-2
Stomach	L01BC02	FLUOROURACILO	20043050-3
Stomach	L01BC02	FLUOROURACILO	20043050-4
Stomach	L01BC02	FLUOROURACILO	20043050-5
Stomach	L01BC02	FLUOROURACILO	20043050-6

Stomach	L01BC02	FLUOROURACILO	20043050-7
Stomach	L01BC02	FLUOROURACILO	20053621-1
Stomach	L01BC02	FLUOROURACILO	20071503-1
Stomach	L01BC02	FLUOROURACILO	20071503-2
Stomach	L01BC02	FLUOROURACILO	20121175-1
Stomach	L01BC02	FLUOROURACILO	20121175-2
Stomach	L01BC02	FLUOROURACILO	20125230-1
Stomach	L01BC02	FLUOROURACILO	20125230-2
Stomach	L01BC02	FLUOROURACILO	20133414-1
Stomach	L01BC02	FLUOROURACILO	216264-1
Stomach	L01BC02	FLUOROURACILO	216264-2
Stomach	L01BC02	FLUOROURACILO	216264-3
Stomach	L01BC02	FLUOROURACILO	227728-2
Stomach	L01BC02	FLUOROURACILO	227728-1
Stomach	L01BC02	FLUOROURACILO	19930999-1
Stomach	L01BC02	FLUOROURACILO	19930999-2
Stomach	L01BC02	FLUOROURACILO	19930999-3
Stomach	L01BC02	FLUOROURACILO	19930999-4
Stomach	L01BC02	FLUOROURACILO	19930999-5
Stomach	L01BC02	FLUOROURACILO	19964018-1
Stomach	L01BC02	FLUOROURACILO	19964018-2
Stomach	L01BC02	FLUOROURACILO	19964018-3
Stomach	L01BC02	FLUOROURACILO	19964018-4
Stomach	L01BC02	FLUOROURACILO	19964018-5
Stomach	L01BC02	FLUOROURACILO	19964018-6
Stomach	L01BC02	FLUOROURACILO	19964018-7
Stomach	L01BC02	FLUOROURACILO	19964018-8
Stomach	L01XX19	IRINOTECAN	19984431-1
Stomach	L01XX19	IRINOTECAN	19938138-1
Stomach	L01XX19	IRINOTECAN	19938138-2
Stomach	L01XX19	IRINOTECAN	19938138-3
Stomach	L01XX19	IRINOTECAN	19938138-4
Stomach	L01XX19	IRINOTECAN	19938138-5
Stomach	L01XX19	IRINOTECAN	207609-9
Stomach	L01XX19	IRINOTECAN	207609-8
Stomach	L01XX19	IRINOTECAN	207609-7
Stomach	L01XX19	IRINOTECAN	207609-6
Stomach	L01XX19	IRINOTECAN	207609-5
Stomach	L01XX19	IRINOTECAN	207609-4
Stomach	L01XX19	IRINOTECAN	207609-3
Stomach	L01XX19	IRINOTECAN	207609-2
Stomach	L01XX19	IRINOTECAN	207609-12
Stomach	L01XX19	IRINOTECAN	207609-11
Stomach	L01XX19	IRINOTECAN	207609-10
Stomach	L01XX19	IRINOTECAN	207609-1
Stomach	L01XX19	IRINOTECAN	19952096-1
Stomach	L01XX19	IRINOTECAN	19952096-2
Stomach	L01XX19	IRINOTECAN	19952096-3

Stomach	L01XX19	IRINOTECAN	19952096-4
Stomach	L01XX19	IRINOTECAN	19952096-5
Stomach	L01XX19	IRINOTECAN	20141121-2
Stomach	L01XX19	IRINOTECAN	20141121-1
Stomach	L01XX19	IRINOTECAN	19967207-1
Stomach	L01XX19	IRINOTECAN	19967207-2
Stomach	L01XX19	IRINOTECAN	19967207-3
Stomach	L01XX19	IRINOTECAN	19973304-1
Stomach	L01XX19	IRINOTECAN	20111292-1
Stomach	L01XX19	IRINOTECAN	19974846-1
Stomach	L01XX19	IRINOTECAN	19976711-1
Stomach	L01XX19	IRINOTECAN	20025051-1
Stomach	L01XX19	IRINOTECAN	20025051-3
Stomach	L01XX19	IRINOTECAN	20025051-2
Stomach	L01XX19	IRINOTECAN	20018486-1
Stomach	L01XX19	IRINOTECAN	20045957-1
Stomach	L01XX19	IRINOTECAN	20012770-1
Stomach	L01XX19	IRINOTECAN	20012621-1
Stomach	L01XX19	IRINOTECAN	20010926-1
Stomach	L01XX19	IRINOTECAN	20053624-1
Stomach	L01XX19	IRINOTECAN	19993941-1
Stomach	L01XX19	IRINOTECAN	20055733-1
Stomach	N02AA01	MORFINA	218192-2
Stomach	N02AA01	MORFINA	20013906-1
Stomach	N02AA01	MORFINA	20013906-2
Stomach	N02AA01	MORFINA	218190-1
Stomach	L01XC17	NIVOLUMAB	20091924-1
Stomach	L01XC17	NIVOLUMAB	20091924-2
Stomach	L01XC17	NIVOLUMAB	20108161-2
Stomach	L01XC17	NIVOLUMAB	20108161-1
Stomach	L01XA03	OXALIPLATINO	19969843-1
Stomach	L01XA03	OXALIPLATINO	20044067-1
Stomach	L01XA03	OXALIPLATINO	19973305-1
Stomach	L01XA03	OXALIPLATINO	20044068-5
Stomach	L01XA03	OXALIPLATINO	20062339-1
Stomach	L01XA03	OXALIPLATINO	20045956-1
Stomach	L01XA03	OXALIPLATINO	20063579-1
Stomach	L01XA03	OXALIPLATINO	20045959-1
Stomach	L01XA03	OXALIPLATINO	19969843-6
Stomach	L01XA03	OXALIPLATINO	19969843-5
Stomach	L01XA03	OXALIPLATINO	19969843-4
Stomach	L01XA03	OXALIPLATINO	19969843-3
Stomach	L01XA03	OXALIPLATINO	19969843-2
Stomach	L01XA03	OXALIPLATINO	20104411-1
Stomach	L01XA03	OXALIPLATINO	20044067-2
Stomach	L01XA03	OXALIPLATINO	19939159-1
Stomach	L01XA03	OXALIPLATINO	20109105-1
Stomach	L01XA03	OXALIPLATINO	20109106-1

Stomach	L01XA03	OXALIPLATINO	20044067-3
Stomach	L01XA03	OXALIPLATINO	20044068-1
Stomach	L01XA03	OXALIPLATINO	20044068-2
Stomach	L01XA03	OXALIPLATINO	20044068-3
Stomach	L01XA03	OXALIPLATINO	20044068-4
Stomach	L01XA03	OXALIPLATINO	19973306-1
Stomach	L01XA03	OXALIPLATINO	20029508-1
Stomach	L01XA03	OXALIPLATINO	223928-1
Stomach	L01XA03	OXALIPLATINO	19939159-2
Stomach	L01XA03	OXALIPLATINO	19939159-3
Stomach	L01XA03	OXALIPLATINO	19939159-4
Stomach	L01XA03	OXALIPLATINO	19997648-1
Stomach	L01XA03	OXALIPLATINO	20067533-1
Stomach	L01XA03	OXALIPLATINO	20056004-1
Stomach	L01XA03	OXALIPLATINO	19993868-1
Stomach	L01XA03	OXALIPLATINO	19989995-4
Stomach	L01XA03	OXALIPLATINO	19989995-3
Stomach	L01XA03	OXALIPLATINO	20047606-1
Stomach	L01XA03	OXALIPLATINO	19963088-1
Stomach	L01XA03	OXALIPLATINO	20011830-1
Stomach	L01XA03	OXALIPLATINO	19963084-1
Stomach	L01XA03	OXALIPLATINO	19989995-2
Stomach	L01XA03	OXALIPLATINO	19989995-1
Stomach	L01XA03	OXALIPLATINO	20151658-3
Stomach	L01XA03	OXALIPLATINO	20151658-2
Stomach	L01XA03	OXALIPLATINO	20151658-1
Stomach	L01XA03	OXALIPLATINO	20056003-1
Stomach	L01XA03	OXALIPLATINO	19952112-2
Stomach	L01XA03	OXALIPLATINO	19952112-1
Stomach	L01XA03	OXALIPLATINO	223932-1
Stomach	L01XA03	OXALIPLATINO	19976714-1
Stomach	L01XA03	OXALIPLATINO	19978042-1
Stomach	L01CD01	PACLITAXEL	19952097-6
Stomach	L01CD01	PACLITAXEL	19952097-5
Stomach	L01CD01	PACLITAXEL	19952097-4
Stomach	L01CD01	PACLITAXEL	20148763-1
Stomach	L01CD01	PACLITAXEL	19952097-3
Stomach	L01CD01	PACLITAXEL	19952097-2
Stomach	L01CD01	PACLITAXEL	19952097-1
Stomach	L01CD01	PACLITAXEL	20047567-1
Stomach	L01CD01	PACLITAXEL	20047566-1
Stomach	L01CD01	PACLITAXEL	20022186-1
Stomach	L01CD01	PACLITAXEL	19946485-6
Stomach	L01CD01	PACLITAXEL	19946485-5
Stomach	L01CD01	PACLITAXEL	19946485-4
Stomach	L01CD01	PACLITAXEL	19946485-3
Stomach	L01CD01	PACLITAXEL	19946485-2
Stomach	L01CD01	PACLITAXEL	19946485-1

Stomach	L01CD01	PACLITAXEL	20192030-1
Stomach	L01CD01	PACLITAXEL	20012769-1
Stomach	L01CD01	PACLITAXEL	20022187-1
Stomach	L01CD01	PACLITAXEL	20018484-1
Stomach	L01CD01	PACLITAXEL	20012772-1
Stomach	L01CD01	PACLITAXEL	222085-1
Stomach	L01CD01	PACLITAXEL	20014726-1
Stomach	L01CD01	PACLITAXEL	20014772-1
Stomach	L01CD01	PACLITAXEL	20018482-1
Stomach	L01CD01	PACLITAXEL	19937677-3
Stomach	L01CD01	PACLITAXEL	19960065-1
Stomach	L01CD01	PACLITAXEL	19937677-2
Stomach	L01CD01	PACLITAXEL	19937677-1
Stomach	L01CD01	PACLITAXEL	55979-9
Stomach	L01CD01	PACLITAXEL	55979-8
Stomach	L01CD01	PACLITAXEL	55979-7
Stomach	L01CD01	PACLITAXEL	55979-6
Stomach	L01CD01	PACLITAXEL	55979-5
Stomach	L01CD01	PACLITAXEL	55979-4
Stomach	L01CD01	PACLITAXEL	55979-3
Stomach	L01CD01	PACLITAXEL	55979-2
Stomach	L01CD01	PACLITAXEL	55979-1
Stomach	L01CD01	PACLITAXEL	20018483-1
Stomach	L01CD01	PACLITAXEL	20020276-1
Stomach	L01CD01	PACLITAXEL	20020276-2
Stomach	L01CD01	PACLITAXEL	20020276-3
Stomach	L01CD01	PACLITAXEL	20031267-1
Stomach	L01CD01	PACLITAXEL	19992001-7
Stomach	L01CD01	PACLITAXEL	20057023-1
Stomach	L01CD01	PACLITAXEL	20057021-1
Stomach	L01CD01	PACLITAXEL	20056002-1
Stomach	L01CD01	PACLITAXEL	19992001-6
Stomach	L01CD01	PACLITAXEL	19992001-5
Stomach	L01CD01	PACLITAXEL	19992001-4
Stomach	L01CD01	PACLITAXEL	19992001-3
Stomach	L01CD01	PACLITAXEL	19992001-2
Stomach	L01CD01	PACLITAXEL	19992001-1
Stomach	L01CD01	PACLITAXEL	19991106-1
Stomach	L01CD01	PACLITAXEL	19991105-2
Stomach	L01CD01	PACLITAXEL	19991105-1
Stomach	L01CD01	PACLITAXEL	19989803-6
Stomach	L01CD01	PACLITAXEL	20061216-1
Stomach	L01CD01	PACLITAXEL	20064116-1
Stomach	L01CD01	PACLITAXEL	20064116-2
Stomach	L01CD01	PACLITAXEL	19989803-5
Stomach	L01CD01	PACLITAXEL	19989803-4
Stomach	L01CD01	PACLITAXEL	19989803-3
Stomach	L01CD01	PACLITAXEL	19989803-2

Stomach	L01CD01	PACLITAXEL	19989803-1
Stomach	L01CD01	PACLITAXEL	20056001-2
Stomach	L01CD01	PACLITAXEL	19980164-1
Stomach	L01CD01	PACLITAXEL	19979611-1
Stomach	L01CD01	PACLITAXEL	20055999-1
Stomach	L01CD01	PACLITAXEL	19976519-2
Stomach	L01CD01	PACLITAXEL	19976519-1
Stomach	L01CD01	PACLITAXEL	19976518-2
Stomach	L01CD01	PACLITAXEL	19976518-1
Stomach	L01CD01	PACLITAXEL	20071691-1
Stomach	L01CD01	PACLITAXEL	20071695-1
Stomach	L01CD01	PACLITAXEL	20071697-1
Stomach	L01CD01	PACLITAXEL	19976352-3
Stomach	L01CD01	PACLITAXEL	19976352-2
Stomach	L01CD01	PACLITAXEL	19976352-1
Stomach	L01CD01	PACLITAXEL	19976351-3
Stomach	L01CD01	PACLITAXEL	19976351-2
Stomach	L01CD01	PACLITAXEL	19976351-1
Stomach	L01CD01	PACLITAXEL	20055740-1
Stomach	L01CD01	PACLITAXEL	20107415-1
Stomach	L01CD01	PACLITAXEL	20107420-1
Stomach	L01CD01	PACLITAXEL	20029992-1
Stomach	L01CD01	PACLITAXEL	20055486-1
Stomach	L01CD01	PACLITAXEL	19973180-1
Stomach	L01CD01	PACLITAXEL	19973179-1
Stomach	L01CD01	PACLITAXEL	19973178-1
Stomach	L01CD01	PACLITAXEL	19973177-1
Stomach	L01CD01	PACLITAXEL	19969254-3
Stomach	L01CD01	PACLITAXEL	19969254-2
Stomach	L01CD01	PACLITAXEL	19969254-1
Stomach	L01CD01	PACLITAXEL	19968458-1
Stomach	L01CD01	PACLITAXEL	19967319-1
Stomach	L01CD01	PACLITAXEL	20029991-1
Stomach	L01CD01	PACLITAXEL	20029990-1
Stomach	L01CD01	PACLITAXEL	19915547-3
Stomach	L01CD01	PACLITAXEL	19915547-2
Stomach	L01CD01	PACLITAXEL	19915547-1
Stomach	L01CD01	PACLITAXEL	19912566-1
Stomach	L01CD01	PACLITAXEL	19963086-1
Stomach	L01CD01	PACLITAXEL	20135292-1
Stomach	L01CD01	PACLITAXEL	20137724-1
Stomach	L01CD01	PACLITAXEL	20137724-2
Stomach	L01CD01	PACLITAXEL	20137724-3
Stomach	L01CD01	PACLITAXEL	20137724-4
Stomach	L01CD01	PACLITAXEL	19957354-1
Stomach	L01CD01	PACLITAXEL	19952110-1
Stomach	L01CD01	PACLITAXEL	19952097-8
Stomach	L01CD01	PACLITAXEL	20007285-1

Stomach	L01CD01	PACLITAXEL	19952097-7
Stomach	L01XY02	PERTUZUMAB Y TRASTUZUMAB	20195976-2
Stomach	L01XY02	PERTUZUMAB Y TRASTUZUMAB	20195976-1
Stomach	L01XC21	RAMUCIRUMAB	20111011-2
Stomach	L01XC21	RAMUCIRUMAB	20111011-1
Stomach	L01XC21	RAMUCIRUMAB	20111011-4
Stomach	L01XC21	RAMUCIRUMAB	20111011-3
Stomach	L01XC03	TRASTUZUMAB	20142329-1
Stomach	L01XC03	TRASTUZUMAB	20176292-1
Stomach	L01XC03	TRASTUZUMAB	20176291-1
Stomach	L01XC03	TRASTUZUMAB	20130360-1
Stomach	L01XC03	TRASTUZUMAB	20194635-1
Stomach	L01XC03	TRASTUZUMAB	19903070-1
Stomach	L01XC03	TRASTUZUMAB	20144826-1
Stomach	L01XC03	TRASTUZUMAB	20143846-1

Annex 3. Descriptive statistics

Balance tests are performed using STATA 17 and pstest command created by Leuven and Sianesi (2018). The standardized % bias is the % difference of the sample means in the treated and non-treated sub-samples as a percentage of the square root of the average of the sample variances in the treated and non-treated groups.

Two-years follow-up cohorts (2YC)

Table A3.1. Breast cancer cohorts 2YC - covariate balance before and after propensity score matching in Colombia, individual-level administrative data, 2017–2021

	Original sample			NN5 matched sample			
Variable	Mean treatment	Mean comparision	Standardised difference (%)	Mean treatment	Mean comparision	Standardised difference (%)	Reduction standardized bias (%)
Stage							
Stage In situ	0.108	0.061	16.7	0.036	0.033	1.3	92.4
Stage I	0.207	0.226	-4.6	0.276	0.271	1.2	74.4
Stage II	0.301	0.293	1.8	0.422	0.427	-0.9	47.3
Stage III	0.194	0.185	2.5	0.212	0.214	-0.6	77.8
Stage IV-V	0.168	0.181	-3.5	0.054	0.055	-0.5	85.4
Medical intervention objetive							
Pre-treatment observation	0.267	0.109	41.5	0.158	0.145	3.4	91.8
Curative or paliative treatment (initial or relapse)	0.701	0.858	-38.5	0.834	0.849	-3.6	90.7
Oncologic follow-up after initial treatment	0.022	0.017	3.2	0.008	0.007	1.2	62.9
Treatment objetive							
Curative treatment	0.91	0.824	25.6	0.977	0.975	0.7	97.2
Paliative treatment	0.072	0.126	-18.2	0.023	0.025	-0.8	95.6
Gaps days between diagnostic and appointment with oncologist							
<16 days	0.425	0.449	-4.9	0.41	0.423	-2.6	47.2
15-30 days	0.243	0.27	-6.1	0.28	0.277	0.8	86.5
+30 days	0.331	0.281	11	0.31	0.301	2	82
HER2 tumor marker tests							
Her2-Positive	0.056	0.045	5.1	0.177	0.168	4.3	15.3
Her2-borderline	0.034	0.027	4.3	0.111	0.108	1.7	59.7
Her2-negative	0.228	0.223	1.1	0.712	0.724	-3	-166.8
Age at diagnosis							
Age 30-34	0.031	0.03	0.2	0.03	0.031	-0.5	-103.3
Age 35-39	0.049	0.042	3.3	0.043	0.047	-1.8	45.8
Age 40-44	0.056	0.052	1.6	0.073	0.073	-0.2	89.8
Age 45-49	0.075	0.066	3.4	0.123	0.123	-0.2	94.7
Age 50-54	0.096	0.082	4.8	0.137	0.152	-5.2	-8.2
Age 55-59	0.108	0.114	-1.8	0.131	0.126	1.5	16.5
Age 60-64	0.136	0.136	-0.1	0.131	0.127	1.1	-670.6
Age 65-69	0.135	0.139	-0.9	0.125	0.117	2.3	-154.4
Age 70-74	0.119	0.112	1.9	0.074	0.069	1.6	16
Age +74	0.181	0.209	-7.1	0.134	0.135	-0.3	95.5
EPS (insurer company)							
EPS 1	0.127	0.088	12.8	0.133	0.14	-2.2	82.9
EPS 2	0.297	0.26	8.2	0.309	0.33	-4.6	43.7
EPS 3	0.331	0.264	14.6	0.351	0.339	2.7	81.3
EPS 4	0.077	0.094	-6.1	0.047	0.043	1.7	72.2
EPS 5	0.169	0	-30	0.16	0.149	2.4	91.9
Residence region							
Antioquia	0.291	0	-3	0.321	0.303	4.1	-52.1
Bogota	0.235	0	8	0.248	0.279	-7.5	4.5
Valle Del Cauca	0.141	0	-10	0.129	0.123	1.7	83.7
North States	0.073	0	-1	0.074	0.073	0.5	45
Center States	0.207	0.189	4.6	0.184	0.178	1.5	66.8
Southwest States	0.035	0.04	-2.6	0.029	0.027	1.2	56.3
Southeast States	0.019	0.012	5.8	0.014	0.018	-2.9	49.6
Lineal distance to nearest main city from residence							
<20km	0.684	0.678	1.4	0.722	0.725	-0.7	52
20-59km	0	0.074	-3.3	0.056	0.054	0.5	86.2

60-179km	0.216	0.222	-1.5	0.19	0.188	0.3	77.4
+179km	0.034	0.026	4.8	0.033	0.032	0.4	92.6
Quarter at diagnostic							
Quarter III	0.62	0.624	-0.9	0.646	0.657	-2.4	-167.5
Quarter IV	0.38	0.376	0.9	0.354	0.343	2.4	-167.5

Table A3.2. Cervical cancer cohorts 2YC - covariate balance before and after propensity score matching in Colombia, individual-level administrative data, 2017–2021

	Original sample			NN5 matched sample			
Variable	Mean treatment	Mean comparison	Standardised difference (%)	Mean treatment	Mean comparison	Standardised difference (%)	Reduction standardized bias (%)
Stage							
Stage In situ	0.108	0.061	16.7	0.376	0.392	-5.7	66
Stage I	0.207	0.226	-4.6	0.276	0.267	2.2	53
Stage II	0.301	0.293	1.8	0.154	0.152	0.5	69.5
Stage III	0.194	0.185	2.5	0.154	0.17	-4	-60.3
Stage IV-V	0.168	0.181	-3.5	0.039	0.019	5.3	-53.3
Treatment objetive							
Curative treatment	0.91	0.824	25.6	0.961	0.977	-4.8	81.2
Paliative treatment	0.072	0.126	-18.2	0.039	0.023	5.5	70.1
Medical intervention objetive							
Pre-treatment observation	0.267	0.109	41.5	0.315	0.331	-4.2	90
Curative or paliative treatment (initial or relapse)	0.701	0.858	-38.5	0.677	0.664	3.2	91.6
Oncologic follow-up after initial treatment	0.022	0.017	3.2	0.007	0.004	1.9	40.3
Gaps days between diagnostic and appointment with oncologist							
<16 days	0.425	0.449	-4.9	0.462	0.459	0.6	87.8
15-30 days	0.243	0.27	-6.1	0.251	0.257	-1.5	75.4
+30 days	0.331	0.281	11	0.287	0.283	0.8	92.9
Age at diagnosis							
Age 30-34	0.031	0.03	0.2	0.151	0.164	-7.9	-3375.1
Age 35-39	0.049	0.042	3.3	0.215	0.21	2.4	26.5
Age 40-44	0.056	0.052	1.6	0.204	0.17	15.1	-864.2
Age 45-49	0.075	0.066	3.4	0.1	0.118	-6.9	-100.7
Age 50-54	0.096	0.082	4.8	0.057	0.05	2.8	43.2
Age 55-59	0.108	0.114	-1.8	0.09	0.102	-4	-121.5
Age 60-64	0.136	0.136	-0.1	0.075	0.07	1.5	-988
Age 65-69	0.135	0.139	-0.9	0.057	0.049	2.4	-162
Age 70-74	0.119	0.112	1.9	0.018	0.038	-6.3	-229.6
Age +74	0.181	0.209	-7.1	0.032	0.029	0.9	87.3
EPS (insurer company)							
EPS 1	0.127	0.088	12.8	0.176	0.163	4	69.1
EPS 2	0.297	0.26	8.2	0.326	0.331	-1.1	86
EPS 3	0.331	0.264	14.6	0.319	0.355	-7.8	46.3
EPS 4	0.077	0.094	-6.1	0.068	0.052	5.7	7.7
EPS 5	0.169	0.294	-30.1	0.111	0.098	3.1	89.8
Lineal distance to nearest main city from residence							
<20km	0.684	0.678	1.4	0.631	0.639	-1.8	-28.9
20-59km	0.065	0.074	-3.3	0.05	0.048	0.8	76.3
60-179km	0.216	0	-2	0.294	0.297	-0.8	48.5
+179km	0.034	0	5	0.025	0.015	5.8	-20.8
Residence region							
Antioquia	0.291	0	-3	0.233	0.281	-10.5	-290
Bogota	0.235	0	8	0.237	0.236	0.1	98.3
Valle Del Cauca	0.141	0	-10	0.14	0.093	12.8	-23.7
North States	0.073	0.076	-1	0.057	0.066	-3.4	-242.2
Center States	0.207	0.189	4.6	0.251	0.224	6.7	-46.3
Southwest States	0.035	0.04	-2.6	0.039	0.049	-5	-90.3
Southeast States	0.019	0.012	5.8	0.043	0.051	-6.5	-12.3
Quarter at diagnostic							
Quarter III	1	0.624	-0.9	0.606	0.653	-9.8	-1010.1
Quarter IV	0.38	0.376	0.9	0.394	0.347	9.8	-1010.1

Table A3.3. Colorectal cancer cohorts 2YC - covariate balance before and after propensity score matching in Colombia, individual-level administrative data, 2017–2021

	Original sample			NN5 matched sample			
Variable	Mean treatment	Mean comparision	Standardised difference (%)	Mean treatment	Mean comparision	Standardised difference (%)	Reduction standardized bias (%)
Sex							
Men	0.349	0.376	-5.7	0.488	0.539	-10.6	-86.1
Women	0.651	0.624	5.7	0.512	0.461	10.6	-86.1
Stage							
Stage I	0.207	0.226	-4.6	0.084	0.06	5.8	-25
Stage II	0.301	0.293	1.8	0.337	0.33	1.6	11.5
Stage III	0.194	0.185	2.5	0.347	0.367	-5	-98.5
Stage IV-V	0.168	0.181	-3.5	0.232	0.243	-3	11.8
Medical intervention objetive							
Pre-treatment observation	0.267	0.109	41.5	0.151	0.128	5.9	85.7
Curative or paliative treatment (initial or relapse)	0.701	0.858	-38.5	0.818	0.849	-7.6	80.2
Oncologic follow-up after initial treatment	0.022	0.017	3.2	0.032	0.023	6	-86.9
Treatment objetive							
Curative treatment	0.91	0.824	25.6	0.909	0.912	-1	96.2
Paliative treatment	0.072	0.126	-18.2	0.091	0.088	1.1	93.9
Gaps days between diagnostic and appointment with oncologist							
<16 days	0.425	0.449	-4.9	0.474	0.459	2.9	40.4
15-30 days	0.243	0.27	-6.1	0.242	0.245	-0.6	89.4
+30 days	0.331	0.281	11	0.284	0.296	-2.5	77.2
Age at diagnosis							
Age 50-54	0.096	0.082	4.8	0.102	0.102	0	99.8
Age 55-59	0.108	0.114	-1.8	0.13	0.166	-11.5	-537.1
Age 60-64	0.136	0.136	-0.1	0.165	0.151	3.9	-2740.9
Age 65-69	0.135	0.139	-0.9	0.161	0.172	-2.9	-221.8
Age 70-74	0.119	0.112	1.9	0.179	0.165	4.4	-131
Age +74	0.181	0.209	-7.1	0.263	0.245	4.6	34.3
EPS (insurer company)							
EPS 1	0.127	0.088	12.8	0.14	0.144	-1.2	90.5
EPS 2	0.297	0.26	8.2	0.218	0.203	3.2	60.7
EPS 3	0.331	0.264	14.6	0.421	0.447	-5.8	60.6
EPS 4	0.077	0.094	-6.1	0.046	0.04	2	67.6
EPS 5	0.169	0.294	-30.1	0.175	0.165	2.4	92
Lineal distance to nearest main city from residence							
<20km	0.684	0.678	1.4	0.74	0.755	-3.2	-122.9
20-59km	0.065	0.074	-3.3	0.053	0.047	2.2	33.5
60-179km	0.216	0.222	-1.5	0.189	0.194	-1	35.8
+179km	0.034	0.026	4.8	0.018	0.004	7.8	-63
Residence region							
Antioquia	0.291	0	-3	0.34	0.359	-4	-49.1
Bogota	0.235	0	8	0.274	0.259	3.6	53.8
Valle Del Cauca	0.141	0	-10	0.105	0.082	6.3	38.9
North States	0.073	0	-1	0.053	0.08	-10.5	-951.2
Center States	0.207	0	5	0.193	0.178	3.8	17
Southwest States	0.035	0.04	-2.6	0.021	0.026	-2.8	-7.2
Southeast States	0.019	0.012	5.8	0.014	0.016	-1.8	69.5
Quarter at diagnostic							
Quarter III	0.62	0.624	-0.9	0.688	0.685	0.5	39
Quarter IV	0.38	0.376	0.9	0.312	0.315	-0.5	39

Table A3.4. Lung cancer cohorts 2YC - covariate balance before and after propensity score matching in Colombia, individual-level administrative data, 2017–2021

	Original sample			NN5 matched sample			
Variable	Mean treatment	Mean comparision	Standardised difference (%)	Mean treatment	Mean comparision	Standardised difference (%)	Reduction standardized bias (%)
Sex							

Men	0.472	0.573	-20.1	0.476	0.484	-1.5	92.5
Women	0.528	0.427	20.1	0.524	0.516	1.5	92.5
Stage							
Stage In situ	0.008	0.023	-12.2	0.008	0	6.4	47.2
Stage I	0.102	0.053	18.3	0.103	0.055	17.9	2
Stage II	0.071	0.046	10.7	0.071	0.059	5.4	49.7
Stage III	0.213	0.061	45	0.206	0.211	-1.4	97
Stage IV-V	0.606	0.817	-47.6	0.611	0.675	-14.4	69.7
Treatment objetive							
Curative treatment	0.701	0.481	45.7	0.698	0.682	3.4	92.6
Paliative treatment	0.299	0.519	-45.7	0.302	0.318	-3.4	92.6
Gaps days between diagnostic and appointment with oncologist							
<16 days	0.535	0.435	20.1	0.532	0.528	0.7	96.3
15-30 days	0.197	0.305	-25.1	0.198	0.192	1.4	94.4
+30 days	0.268	0.26	1.8	0.27	0.28	-2.2	-19
Age at diagnosis							
Age 50-54	0.087	0.092	-1.7	0.079	0.076	1.3	24.4
Age 55-59	0.11	0.092	6.2	0.111	0.121	-3.1	49.2
Age 60-64	0.15	0.176	-7	0.151	0.204	-14.3	-103.2
Age 65-69	0.228	0.206	5.4	0.23	0.186	10.8	-100.2
Age 70-74	0.134	0.137	-1	0.135	0.133	0.5	47.7
Age +74	0.291	0.298	-1.4	0.294	0.282	2.6	-89.2
EPS (insurer company)							
EPS 1	0.071	0.038	14.4	0.071	0.053	8.1	44
EPS 2	0.157	0.176	-4.8	0.159	0.157	0.5	90.5
EPS 3	0.504	0.351	31.1	0.5	0.578	-15.9	49.1
EPS 4	0.039	0.046	-3.2	0.04	0.056	-8.2	-158.3
EPS 5	0.228	0.389	-35.2	0.23	0.156	16.3	53.8
Lineal distance to nearest main city from residence							
<20km	0.756	0.725	7	0.754	0.797	-9.8	-40.1
20-59km	0.079	0.084	-1.9	0.079	0.093	-4.8	-153.9
60-179km	0.165	0.191	-6.6	0.167	0.11	14.7	-120.9
Residence region							
Antioquia	0.52	0.466	10.8	0.524	0.585	-12.2	-12.7
Bogota	0.15	0.176	-7	0.151	0.159	-2.3	66.7
Valle Del Cauca	0.126	0.092	11	0.119	0.105	4.4	60
North States	0.047	0	5	0.048	0.025	11.1	-148.7
Center States	0.126	0	-18	0.127	0.103	6.5	63.6
Southwest States	0.024	0	-4	0.024	0.022	1.3	70.5
Southeast States	0.008	0	0	0.008	0	8.6	-3057.2
Quarter at diagnostic							
Quarter III	0.646	1	9	0.643	0.579	13.1	-48.9
Quarter IV	0.354	0	-9	0.357	0.421	-13.1	-48.9

Table A3.5. Prostate cancer cohorts 2YC - covariate balance before and after propensity score matching in Colombia, individual-level administrative data, 2017–2021

	Original sample			NN5 matched sample			
Variable	Mean treatment	Mean comparision	Standardised difference (%)	Mean treatment	Mean comparision	Standardised difference (%)	Reduction standardized bias (%)
Stage							
Stage In situ	0.108	0.061	16.7	0.009	0.014	-1.5	90.8
Stage I	0.207	0.226	-4.6	0.332	0.327	1.2	73.6
Stage II	0.301	0.293	1.8	0.409	0.386	5	-182.5
Stage III	0.194	0.185	2.5	0.101	0.092	2.3	6.8
Stage IV-V	0.168	0.181	-3.5	0.148	0.181	-8.7	-151.4
Treatment objetive							
Curative treatment	0.91	0.824	25.6	0.967	0.97	-0.8	96.7
Paliative treatment	0.072	0.126	-18.2	0.033	0.03	0.9	94.8
Medical intervention objetive							
Pre-treatment observation	0.267	0.109	41.5	0.315	0.291	6.3	84.8
Curative or paliative treatment (initial or relapse)	0.701	0.858	-38.5	0.675	0.702	-6.7	82.7
Oncologic follow-up after initial treatment	0.022	0.017	3.2	0.009	0.006	2.2	32
Gaps days between diagnostic and appointment with oncologist							
<16 days	0.425	0.449	-4.9	0.311	0.307	0.8	83.8
15-30 days	0.243	0.27	-6.1	0.205	0.201	0.9	84.7
+30 days	0.331	0.281	11	0.485	0.493	-1.7	84.3
Age at diagnosis							
Age 50-54	0.096	0.082	4.8	0.026	0.02	2.1	57.2
Age 55-59	0.108	0.114	-1.8	0.078	0.105	-8.7	-385.3
Age 60-64	0.136	0.136	-0.1	0.184	0.172	3.5	-2415.7

Age 65-69	0.135	0.139	-0.9	0.205	0.208	-0.9	0.5
Age 70-74	0.119	0.112	1.9	0.24	0.207	10.3	-439.1
Age +74	0.181	0.209	-7.1	0.268	0.289	-5.1	27.2
EPS (insurer company)							
EPS 1	0.127	0.088	12.8	0.094	0.1	-1.9	84.8
EPS 2	0.297	0.26	8.2	0.278	0.274	0.9	88.7
EPS 3	0.331	0.264	14.6	0.386	0.403	-3.8	73.7
EPS 4	0.077	0.094	-6.1	0.064	0.045	6.6	-7.1
EPS 5	0.169	0.294	-30.1	0.179	0.178	0.2	99.2
Lineal distance to nearest main city from residence							
<20km	0.684	0.678	1.4	0.72	0.707	2.9	-99.1
20-59km	0.065	0.074	-3.3	0.078	0.092	-5.5	-63.9
60-179km	0.216	0.222	-1.5	0.179	0.181	-0.6	61.7
+179km	0.034	0.026	4.8	0.024	0.02	1.8	62.5
Residence region							
Antioquia	0.291	0.303	-2.7	0.379	0.405	-5.8	-114.4
Bogota	0.235	0	8	0.174	0.154	4.8	39.1
Valle Del Cauca	0.141	0	-10	0.165	0.159	1.6	84.5
North States	0.073	0	-1	0.061	0.072	-4	-299.2
Center States	0.207	0	5	0.186	0.185	0.2	96.2
Southwest States	0.035	0	-3	0.028	0.019	5.1	-93
Southeast States	0.019	0	6	0.007	0.006	0.7	88.3
Quarter at diagnostic							
Quarter III	0.62	0.624	-0.9	0.696	0.677	4	-357.6
Quarter IV	0.38	0.376	0.9	0.304	0.323	-4	-357.6

Table A3.6. Stomach cancer cohorts 2YC - covariate balance before and after propensity score matching in Colombia, individual-level administrative data, 2017–2021

	Original sample			NN5 matched sample			
Variable	Mean treatment	Mean comparision	Standardised difference (%)	Mean treatment	Mean comparision	Standardised difference (%)	Reduction standardized bias (%)
Sex							
Men	0.349	0.376	-5.7	0.615	0.601	3	47.1
Women	0.651	0.624	5.7	0.385	0.4	-3	47.1
Stage							
Stage I	0.207	0.226	-4.6	0.12	0.133	-3.1	33.3
Stage II	0.301	0.293	1.8	0.185	0.207	-4.8	-166.7
Stage III	0.194	0.185	2.5	0.28	0.267	3.3	-30.5
Stage IV-V	0.168	0.181	-3.5	0.415	0.393	5.7	-65.5
Treatment objetive							
Curative treatment	0.91	0.824	25.6	0.74	0.783	-12.7	50.5
Paliative treatment	0.072	0.126	-18.2	0.26	0.217	14.4	21.3
Gaps days between diagnostic and appointment with oncologist							
<16 days	0.425	0.449	-4.9	0.43	0.44	-1.9	60.3
15-30 days	0.243	0.27	-6.1	0.26	0.221	9	-48.9
+30 days	0.331	0.281	11	0.31	0.34	-6.5	40.9
Age at diagnosis							
Age 45-49	0.075	0.066	3.4	0.075	0.071	1.7	52
Age 50-54	0.096	0.082	4.8	0.15	0.137	4.6	4.5
Age 55-59	0.108	0.114	-1.8	0.1	0.113	-4.2	-136.1
Age 60-64	0.136	0.136	-0.1	0.165	0.153	3.4	-2366.2
Age 65-69	0.135	0.139	-0.9	0.125	0.162	-10.8	-1077.6
Age 70-74	0.119	0.112	1.9	0.145	0.149	-1.1	42.4
Age +74	0.181	0.209	-7.1	0.24	0.215	6.2	11.8
EPS (insurer company)							
EPS 1	0.127	0.088	12.8	0.115	0.148	-10.8	15.9
EPS 2	0.297	0.26	8.2	0.3	0.316	-3.6	56.1
EPS 3	0.331	0.264	14.6	0.34	0.31	6.6	54.8
EPS 4	0.077	0.094	-6.1	0.065	0.066	-0.5	91.4
EPS 5	0.169	0.294	-30.1	0.18	0.159	5	83.5
Lineal distance to nearest main city from residence							
<20km	0.684	0.678	1.4	0.74	0.774	-7.4	-415
20-59km	0.065	0.074	-3.3	0.055	0.06	-1.9	43.3
60-179km	0.216	0.222	-1.5	0.19	0.147	10.3	-571.8
+179km	0.034	0.026	4.8	0.015	0.018	-2	59.1

Residence region							
Antioquia	0.291	0.303	-2.7	0.29	0.305	-3.3	-23.3
Bogota	0.235	0.202	7.9	0.315	0.321	-1.5	80.5
Valle Del Cauca	0.141	0	-10	0.105	0.1	1.4	86.6
North States	0.073	0	-1	0.02	0.018	0.8	22.3
Center States	0.207	0	5	0.245	0.227	4.6	0.8
Southwest States	0.035	0	-3	0.02	0.026	-3.1	-19.3
Southeast States	0.019	0	6	0.005	0.003	1.7	70.4
Quarter at diagnostic							
Quarter III	0.62	1	-1	0.67	0.722	-10.7	-1115.9
Quarter IV	0.38	0	1	0.33	0.278	10.7	-1115.9

15 months follow-up cohorts (15MC)

Table A3.1. Breast cancer cohorts 15MC - covariate balance before and after propensity score matching in Colombia, individual-level administrative data, 2017–2021

	Original sample			NN5 matched sample			
Variable	Mean treatment	Mean comparision	Standardised difference (%)	Mean treatment	Mean comparision	Standardised difference (%)	Reduction standardized bias (%)
Stage							
Stage In situ	0.108	0.103	1.8	0.036	0.02	5.3	-204.6
Stage I	0.207	0.238	-7.4	0.276	0.274	0.5	93.7
Stage II	0.301	0.295	1.4	0.422	0.427	-1.1	21.2
Stage III	0.194	0.193	0.5	0.212	0.223	-2.7	-479.6
Stage IV-V	0.168	0.163	1.3	0.054	0.057	-0.8	39.7
Medical intervention objetive							
Pre-treatment observation	0.267	0.289	-4.8	0.158	0.142	3.6	26.3
Curative or paliative treatment (initial or relapse)	0.701	0.698	0.7	0.834	0.853	-4.1	-520.4
Oncologic follow-up after initial treat ment	0.022	0.011	8.1	0.008	0.006	2	74.7
Treatment objetive							
Curative treatment	0.91	0.896	4.7	0.977	0.977	0.2	96.5
Paliative treatment	0.072	0.103	-11.1	0.023	0.023	-0.2	98.4
Gaps days between diagnostic and appointment with oncologist							
<16 days	0.425	0.442	-3.3	0.41	0.41	0	99.8
15-30 days	0.243	0.245	-0.4	0.28	0.272	1.9	-353.2
+30 days	0.331	0.313	3.9	0.31	0.318	-1.8	53.9
HER2 tumor marker tests							
Her2-Positive	0.056	0.067	-4.3	0.177	0.17	3	30.6
Her2-borderline	0.034	0.03	2.3	0.111	0.112	-0.4	83.4
Her2-negative	0.228	0.217	2.6	0.712	0.718	-1.6	39.2
Age at diagnosis							
Age 30-34	0.031	0.036	-2.7	0.03	0.03	-0.2	91.5
Age 35-39	0.049	0.049	-0.1	0.043	0.045	-0.9	-962.1
Age 40-44	0.056	0.053	1.1	0.073	0.073	0	96
Age 45-49	0.075	0.068	2.5	0.123	0.135	-4.6	-85.6
Age 50-54	0.096	0.089	2.5	0.137	0.144	-2.4	2.8
Age 55-59	0.108	0.122	-4.3	0.131	0.127	1.3	69.6
Age 60-64	0.136	0.136	-0.1	0.131	0.128	0.9	-1290
Age 65-69	0.135	0.136	-0.1	0.125	0.116	2.6	-2295.8

Age 70-74	0.119	0.116	0.7	0.074	0.075	-0.2	73.6
Age +74	0.181	0.174	1.8	0.134	0.128	1.5	13.3
EPS (insurer company)							
EPS 1	0.127	0.159	-9.1	0.133	0.129	1.2	87.1
EPS 2	0.297	0.235	14.1	0.309	0.303	1.3	90.6
EPS 3	0.331	0.327	0.7	0.351	0.373	-4.6	-512.9
EPS 4	0.077	0.063	5.5	0.047	0.048	-0.4	92.8
EPS 5	0.169	0	-12	0.16	0.147	3.2	73.5
Residence region							
Antioquia	0.291	0	-13	0.321	0.328	-1.5	88.4
Bogota	0.235	0	5	0.248	0.239	2.1	60.2
Valle Del Cauca	0.141	0	-2	0.129	0.127	0.6	74.3
North States	0.073	0	-3	0.074	0.074	0.1	97.9
Center States	0.207	0.156	13.2	0.184	0.193	-2.2	83.5
Southwest States	0.035	0.034	0.5	0.029	0.023	3	-500.7
Southeast States	0.019	0.016	2.3	0.014	0.015	-0.7	68.4
Lineal distance to nearest main city from residence							
<20km	0.684	0.698	-2.9	0.722	0.723	-0.1	95.7
20-59km	0	0.072	-2.5	0.056	0.054	0.6	77
60-179km	0.216	0.188	7	0.19	0.19	-0.2	97.5
+179km	0.034	0.042	-4.2	0.033	0.033	-0.1	97.8
Quarter at diagnostic							
Quarter III	0.62	0.647	-5.7	0.646	0.657	-2.2	60.3
Quarter IV	0.38	0.353	5.7	0.354	0.343	2.2	60.3

Table A3.2. Breast cancer cohorts 15MC - covariate balance before and after propensity score matching in Colombia, individual-level administrative data, 2017–2021

	Original sample			NN5 matched sample			
Variable	Mean treatment	Mean comparision	Standardised difference (%)	Mean treatment	Mean comparision	Standardised difference (%)	Reduction standardized bias (%)
Stage							
Stage In situ	0.108	0.103	1.800	0.376	0.392	-5.200	-197.100
Stage I	0.207	0.238	-7.400	0.276	0.279	-0.800	89.700
Stage II	0.301	0.295	1.400	0.154	0.133	4.700	-244.200
Stage III	0.194	0.193	0.500	0.154	0.154	0.100	69.300
Stage IV-V	0.168	0.163	1.300	0.039	0.042	-0.700	44.400
Treatment objetive							
Curative treatment	0.910	0.896	4.700	0.961	0.968	-2.400	47.700
Paliative treatment	0.072	0.103	-11.100	0.039	0.032	2.600	76.900
Medical intervention objetive							
Pre-treatment observation	0.267	0.289	-4.800	0.315	0.338	-5.100	-5.600
Curative or paliative treatment (initial or relapse)	0.701	0.698	0.700	0.677	0.656	4.600	-598.700
Oncologic follow-up after initial treatment	0.022	0.011	8.100	0.007	0.005	1.500	81.200
Gaps days between diagnostic and appointment with oncologist							
<16 days	0.425	0.442	-3.300	0.462	0.484	-4.400	-34.100
15-30 days	0.243	0.245	-0.400	0.251	0.238	2.900	-578.100
+30 days	0.331	0.313	3.900	0.287	0.277	2.000	48.700
Age at diagnosis							
Age 30-34	0.031	0.036	-2.700	0.151	0.122	15.800	-485.900
Age 35-39	0.049	0.049	-0.100	0.215	0.226	-5.300	-5814.800
Age 40-44	0.056	0.053	1.100	0.204	0.217	-5.700	-438.900
Age 45-49	0.075	0.068	2.500	0.100	0.105	-2.000	21.800
Age 50-54	0.096	0.089	2.500	0.057	0.053	1.400	44.000
Age 55-59	0.108	0.122	-4.300	0.090	0.106	-5.100	-17.300
Age 60-64	0.136	0.136	-0.100	0.075	0.071	1.100	-1680.400
Age 65-69	0.135	0.136	-0.100	0.057	0.056	0.500	-341.000
Age 70-74	0.119	0.116	0.700	0.018	0.017	0.400	45.200
Age +74	0.181	0.174	1.800	0.032	0.026	1.700	2.200
EPS (insurer company)							
EPS 1	0.127	0.159	-9.100	0.176	0.170	1.600	82.400
EPS 2	0.297	0.235	14.100	0.326	0.325	0.200	98.600
EPS 3	0.331	0.327	0.700	0.319	0.313	1.300	-71.600
EPS 4	0.077	0.063	5.500	0.068	0.073	-2.100	61.700
EPS 5	0.169	0.217	-12.100	0.111	0.118	-1.800	84.900

Lineal distance to nearest main city from residence							
<20km	0.684	0.698	-2.900	0.631	0.657	-5.700	-94.800
20-59km	0.065	0.072	-2.500	0.050	0.060	-4.000	-57.000
60-179km	0.216	0.188	7.000	0.294	0.257	9.200	-31.000
+179km	0.034	0.042	-4.200	0.025	0.025	-0.200	96.000
Residence region							
Antioquia	0.291	0.353	-13.200	0.233	0.248	-3.200	75.900
Bogota	0.235	0.212	5.300	0.237	0.244	-1.800	66.600
Valle Del Cauca	0.141	0.149	-2.200	0.140	0.164	-6.700	-205.900
North States	0.073	0.080	-2.800	0.057	0.053	1.800	37.300
Center States	0.207	0.156	13.200	0.251	0.211	10.300	21.900
Southwest States	0.035	0.034	0.500	0.039	0.029	5.700	-1032.300
Southeast States	0.019	0.016	2.300	0.043	0.052	-6.700	-196.700
Quarter at diagnostic							
Quarter III	0.620	0.647	-5.700	0.606	0.604	0.300	94.800
Quarter IV	0.380	0.353	5.700	0.394	0.396	-0.300	94.800

Table A3.3. Colorectal cancer cohorts 15MC - covariate balance before and after propensity score matching in Colombia, individual-level administrative data, 2017–2021

	Original sample			NN5 matched sample			
Variable	Mean treatment	Mean comparison	Standardised difference (%)	Mean treatment	Mean comparison	Standardised difference (%)	Reduction standardized bias (%)
Sex							
Men	0.349	0.35	-0.1	0.484	0.479	1	-592.8
Women	0.651	0.65	0.1	0.516	0.521	-1	-592.8
Stage							
Stage In situ	0.108	0.103	1.8	0.01	0.01	0.1	96.7
Stage I	0.207	0.238	-7.4	0.078	0.06	4.4	40.1
Stage II	0.301	0.295	1.4	0.331	0.345	-3.1	-126.1
Stage III	0.194	0.193	0.5	0.357	0.35	1.8	-292.2
Stage IV-V	0.168	0.163	1.3	0.224	0.236	-3.1	-139.5
Medical intervention objetive							
Pre-treatment observation	0.267	0.289	-4.8	0.205	0.192	2.8	41.6
Curative or paliative treatment (initial or relapse)	0.701	0.698	0.7	0.766	0.783	-3.7	-470.5
Oncologic follow-up after initial treatment	0.022	0.011	8.1	0.029	0.025	3.5	57
Treatment objetive							
Curative treatment	0.91	0.896	4.7	0.912	0.907	1.9	58.9
Paliative treatment	0.072	0.103	-11.1	0.088	0.093	-2	81.8
Gaps days between diagnostic and appointment with oncologist							
<16 days	0.425	0.442	-3.3	0.455	0.437	3.6	-9.6
15-30 days	0.243	0.245	-0.4	0.24	0.243	-0.7	-51.7
+30 days	0.331	0.313	3.9	0.305	0.32	-3.2	17.1
Age at diagnosis							
Age 50-54	0.096	0.089	2.5	0.094	0.108	-4.9	-98.4
Age 55-59	0.108	0.122	-4.3	0.127	0.141	-4.5	-3.4
Age 60-64	0.136	0.136	-0.1	0.162	0.142	6	-9367.3
Age 65-69	0.135	0.136	-0.1	0.172	0.176	-1.3	-1096.1
Age 70-74	0.119	0.116	0.7	0.179	0.183	-1.5	-127.4
Age +74	0.181	0.174	1.8	0.266	0.249	4.5	-153.5
EPS (insurer company)							
EPS 1	0.127	0.159	-9.1	0.133	0.122	3.1	66.1
EPS 2	0.297	0.235	14.1	0.201	0.219	-3.9	72.1
EPS 3	0.331	0.327	0.7	0.445	0.451	-1.2	-66.1
EPS 4	0.077	0.063	5.5	0.055	0.053	0.8	84.9
EPS 5	0.169	0.217	-12.1	0.166	0.155	2.6	78.5
Lineal distance to nearest main city from residence							
<20km	0.684	0.698	-2.9	0.734	0.738	-0.8	72.3
20-59km	0.065	0.072	-2.5	0.049	0.048	0.4	83.8
60-179km	0.216	0.188	7	0.201	0.198	0.9	87.5
+179km	0.034	0	-4	0.016	0.017	-0.4	90.3
Residence region							
Antioquia	0.291	0	-13	0.328	0.334	-1.4	89.4
Bogota	0.235	0	5	0.266	0.271	-1.1	79.9
Valle Del Cauca	0.141	0	-2	0.11	0.104	1.9	15.1
North States	0.073	0	-3	0.052	0.048	1.5	47.8

Center States	0.207	0.156	13.2	0.211	0.215	-1	92.8
Southwest States	0.035	0.034	0.5	0.019	0.017	1.2	-129.8
Southeast States	0.019	0.016	2.3	0.013	0.011	1.6	28.8

Quarter at diagnostic							
Quarter III	0.62	0.647	-5.7	0.685	0.699	-2.8	50.5

Table A3.4. Lung cancer cohorts 15MC - covariate balance before and after propensity score matching in Colombia, individual-level administrative data, 2017–2021

	Original sample			NN5 matched sample			
Variable	Mean treatment	Mean comparision	Standardised difference (%)	Mean treatment	Mean comparision	Standardised difference (%)	Reduction standardized bias (%)
Sex							
Men	0.477	0.585	-21.8	0.484	0.531	-9.4	56.7
Women	0.523	0.415	21.8	0.516	0.469	9.4	56.7
Stage							
Stage In situ	0.008	0.024	-13.3	0.008	0.022	-11.4	14.2
Stage I	0.1	0.146	-14.1	0.102	0.113	-3.6	74.7
Stage II	0.069	0.065	1.7	0.07	0.045	10.1	-507.9
Stage III	0.208	0.146	16.1	0.203	0.199	1	93.7
Stage IV-V	0.615	0.618	-0.5	0.617	0.62	-0.7	-31.3
Treatment objetive							
Curative treatment	0.708	0.569	29	0.703	0.697	1.3	95.4
Paliative treatment	0.292	0.431	-29	0.297	0.303	-1.3	95.4
Gaps days between diagnostic and appointment with oncologist							
<16 days	0.538	0.488	10.1	0.531	0.524	1.3	86.7
15-30 days	0.192	0.268	-18	0.195	0.215	-4.6	74.6
+30 days	0.269	0.244	5.8	0.273	0.261	2.9	50.4
Age at diagnosis							
Age 50-54	0.085	0.033	22.2	0.078	0.059	8	64
Age 55-59	0.108	0.049	22	0.109	0.096	4.8	78.1
Age 60-64	0.146	0.114	9.6	0.141	0.198	-17	-77.4
Age 65-69	0.231	0.203	6.7	0.234	0.24	-1.3	81
Age 70-74	0.131	0.293	-40.3	0.133	0.143	-2.7	93.4
Age +74	0.3	0.309	-1.9	0.305	0.263	9	-364.7
EPS (insurer company)							
EPS 1	0.069	0.073	-1.5	0.07	0.084	-5.4	-253.4
EPS 2	0.154	0.211	-14.9	0.156	0.148	2.2	85.1
EPS 3	0.492	0.366	25.7	0.484	0.525	-8.3	67.8
EPS 4	0.038	0.033	3.2	0.039	0.023	8.6	-170.1
EPS 5	0.246	0.317	-15.8	0.25	0.22	6.7	57.6
Lineal distance to nearest main city from residence							
<20km	0.738	0.756	-4	0.742	0.746	-0.9	77.3
20-59km	0.077	0.073	1.4	0.078	0.068	4	-178.9
60-179km	0.162	0.122	11.3	0.156	0.163	-1.8	84
+179km	0.023	0.049	-13.8	0.023	0.024	-0.1	99.6
Residence region							
Antioquia	0.515	0.463	10.4	0.516	0.531	-3	70.8
Bogota	0.146	0.203	-15	0.148	0.13	4.8	67.7
Valle Del Cauca	0.123	0	0	0.125	0.143	-5.6	-1536.2
North States	0.054	0	-8	0.055	0.06	-2.3	71.3
Center States	0.123	0	8	0.117	0.1	5.4	33
Southwest States	0.023	0	-1	0.023	0.018	3.6	-319.9
Southeast States	0.015	0.016	-0.7	0.016	0.018	-1.6	-121.8
Quarter at diagnostic							
Quarter III	0.638	0.756	-25.7	0.648	0.618	6.6	74.4
Quarter IV	0.362	0.244	25.7	0.352	0.382	-6.6	74.4

Table A3.5. Prostate cancer cohorts 15MC - covariate balance before and after propensity score matching in Colombia, individual-level administrative data, 2017–2021

	Original sample			NN5 matched sample			
Variable	Mean treatment	Mean comparision	Standardised difference (%)	Mean treatment	Mean comparision	Standardised difference (%)	Reduction standardized bias (%)

Stage							
Stage In situ	0.108	0.103	1.8	0.009	0.011	-0.5	72.1
Stage I	0.207	0.238	-7.4	0.332	0.329	0.6	92.2
Stage II	0.301	0.295	1.4	0.409	0.402	1.7	-23.3
Stage III	0.194	0.193	0.5	0.101	0.109	-2	-327.7
Stage IV-V	0.168	0.163	1.3	0.148	0.149	-0.2	83.9
Treatment objetive							
Curative treatment	0.91	0.896	4.7	0.967	0.976	-3	35.8
Paliative treatment	0.072	0.103	-11.1	0.033	0.024	3.1	71.7
Medical intervention objetive							
Pre-treatment observation	0.267	0.289	-4.8	0.315	0.317	-0.5	90.2
Curative or paliative treatment (initial or relapse)	0.701	0.698	0.7	0.675	0.675	0.2	76.8
Oncologic follow-up after initial treatment	0.022	0.011	8.1	0.009	0.008	1.1	86.2
Gaps days between diagnostic and appointment with oncologist							
<16 days	0.425	0.442	-3.3	0.311	0.313	-0.5	84.1
15-30 days	0.243	0.245	-0.4	0.205	0.214	-2.2	-410.4
+30 days	0.331	0.313	3.9	0.485	0.473	2.6	33.6
Age at diagnosis							
Age 50-54	0.096	0.089	2.5	0.026	0.029	-1.1	54.9
Age 55-59	0.108	0.122	-4.3	0.078	0.087	-2.8	35
Age 60-64	0.136	0.136	-0.1	0.184	0.17	3.9	-6099.2
Age 65-69	0.135	0.136	-0.1	0.205	0.225	-5.9	-5420.4
Age 70-74	0.119	0.116	0.7	0.24	0.23	3.1	-373.9
Age +74	0.181	0.174	1.8	0.268	0.259	2.3	-30.2
EPS (insurer company)							
EPS 1	0.127	0.159	-9.1	0.094	0.103	-2.5	72.5
EPS 2	0.297	0.235	14.1	0.278	0.275	0.6	96
EPS 3	0.331	0.327	0.7	0.386	0.391	-1.1	-47.1
EPS 4	0.077	0.063	5.5	0.064	0.063	0.1	98.5
EPS 5	0.169	0.217	-12.1	0.179	0.168	2.9	76.3
Lineal distance to nearest main city from residence							
<20km	0.684	0.698	-2.9	0.72	0.724	-0.9	70.1
20-59km	0.065	0.072	-2.5	0.078	0.079	-0.5	78.4
60-179km	0.216	0.188	7	0.179	0.168	2.8	59.7
+179km	0.034	0.042	-4.2	0.024	0.029	-3.1	27.6
Residence region							
Antioquia	0.291	0.353	-13.2	0.379	0.394	-3.3	75.4
Bogota	0.235	0	5	0.174	0.164	2.4	54.1
Valle Del Cauca	0.141	0	-2	0.165	0.161	1.1	50.4
North States	0.073	0	-3	0.061	0.069	-2.9	-3.6
Center States	0.207	0	13	0.186	0.18	1.5	88.7
Southwest States	0.035	0	1	0.028	0.029	-0.4	15.4
Southeast States	0.019	0	2	0.007	0.003	3	-32
Quarter at diagnostic							
Quarter III	0.62	0.647	-5.7	0.696	0.701	-1	82.8
Quarter IV	0.38	0.353	5.7	0.304	0.299	1	82.8

Table A3.6. Stomach cancer cohorts 15MC - covariate balance before and after propensity score matching in Colombia, individual-level administrative data, 2017–2021

	Original sample			NN5 matched sample			
Variable	Mean treatment	Mean comparision	Standardised difference (%)	Mean treatment	Mean comparision	Standardised difference (%)	Reduction standardized bias (%)
Sex							
Men	0.349	0.35	-0.1	0.626	0.646	-4.1	-2842.6
Women	0.651	0.65	0.1	0.374	0.354	4.1	-2842.6
Stage							
Stage In situ	0.108	0.103	1.8	0.016	0.019	-1.2	33.1
Stage I	0.207	0.238	-7.4	0.121	0.122	-0.2	96.8
Stage II	0.301	0.295	1.4	0.179	0.174	1.2	13.1
Stage III	0.194	0.193	0.5	0.268	0.262	1.7	-278.4
Stage IV-V	0.168	0.163	1.3	0.416	0.423	-2.1	-56.3
Treatment objetive							
Curative treatment	0.91	0.896	4.7	0.747	0.783	-12.1	-158.9
Paliative treatment	0.072	0.103	-11.1	0.253	0.217	12.7	-14.3

Gaps days between diagnostic and appointment with oncologist							
<16 days	0.425	0.442	-3.3	0.426	0.409	3.5	-7.5
15-30 days	0.243	0.245	-0.4	0.263	0.271	-1.8	-324.9
+30 days	0.331	0.313	3.9	0.311	0.32	-2.1	46.8
Age at diagnosis							

Age 45-49	0.075	0.068	2.5	0.079	0.071	2.9	-16.6
Age 50-54	0.096	0.089	2.5	0.132	0.111	7	-185.8
Age 55-59	0.108	0.122	-4.3	0.095	0.099	-1.4	67.9
Age 60-64	0.136	0.136	-0.1	0.163	0.168	-1.5	-2265.6
Age 65-69	0.135	0.136	-0.1	0.126	0.105	6.1	-5594.4
Age 70-74	0.119	0.116	0.7	0.158	0.166	-2.4	-268
Age +74	0.181	0.174	1.8	0.247	0.279	-8.2	-363.6
EPS (insurer company)							
EPS 1	0.127	0.159	-9.1	0.105	0.099	1.7	81.5
EPS 2	0.297	0.235	14.1	0.289	0.303	-3	78.8
EPS 3	0.331	0.327	0.7	0.342	0.329	2.7	-263.1
EPS 4	0.077	0.063	5.5	0.068	0.074	-2.2	60
EPS 5	0.169	0.217	-12.1	0.195	0.195	0	99.9
Lineal distance to nearest main city from residence							
<20km	0.684	0.698	-2.9	0.721	0.729	-1.7	41.7
20-59km	0.065	0.072	-2.5	0.058	0.063	-1.8	28
60-179km	0.216	0.188	7	0.205	0.195	2.6	62.3
+179km	0.034	0.042	-4.2	0.016	0.014	1	76.2
Residence region							
Antioquia	0.291	0.353	-13.2	0.316	0.313	0.6	95.5
Bogota	0.235	0	5	0.332	0.328	0.8	84.7
Valle Del Cauca	0.141	0	-2	0.111	0.116	-1.4	35.3
North States	0.073	0	-3	0.021	0.022	-0.4	84.6
Center States	0.207	0	13	0.189	0.184	1.3	90
Southwest States	0.035	0	1	0.026	0.035	-4.5	-801.9
Southeast States	0.019	0	2	0.005	0.002	2.4	-7.5
Quarter at diagnostic							
Quarter III	0.62	1	-6	0.674	0.68	-1.3	77.5
Quarter IV	0.38	0	6	0.326	0.32	1.3	77.5

Annex 4. Overall balance test of propensity score

The table presents the standard general tests used to assess the balance of the propensity score. Pseudo R2 measures how well the covariates explain treatment assignment, with lower values after matching indicating better balance. The LR-Chi2 test evaluates the joint significance of covariates, and non-significant values post-matching demonstrate improved balance. Mean and median biases quantify the average differences in covariates between treated and control groups, with lower values post-matching indicating reduced bias.

For colorectal, cervical, stomach, breast, and prostate cancers, the Pseudo-R2 values approached zero, the LR-Chi2 statistics became non-significant, and both mean and median biases substantially decreased post-matching. Rubin's B, which compares the standardized differences in covariates between groups, also showed improvement but remained above the acceptable threshold of 25% for lung cancer in the 15-month cohort. In the 2-year cohort, Rubin's B did not meet acceptable values, indicating residual imbalance. Conversely, Rubin's R, which assesses the ratio of variances of the propensity score between treated and control groups, exhibited acceptable values across all cancer types.

Table A4. Matching performance tests

Two-years follow-up cohorts

Cancer	Sample	Pseudo R2	LR-Chi2	P>Chi2	Mean Bias	Med Bias	Rubins' B	Rubins' R	Untreated	Treated	Obs
Colorectal	Unmatched	0.067	458.35	0.000	8	4.8	61.7*	.84	296	285	581
Colorectal	Matched	0.016	12.31	0.996	3.3	2.8	29.3*	1.38			
Cervical	Unmatched	0.067	458.07	0.000	8.1	4.6	61.7*	.84	169	279	448
Cervical	Matched	0.02	15.38	0.994	4.1	3.4	33.3*	1.27			
Stomach	Unmatched	0.048	327.45	0.000	6.7	4.6	51.6*	.61	131	200	331
Stomach	Matched	0.017	9.44	0.999	4.3	3.3	30.9*	1.29			
Breast	Unmatched	0.069	470.29	0.000	7.8	4.6	62.5*	.84	691	971	1662
Breast	Matched	0.003	7.30	1.000	1.7	1.3	12.3	1.43			
Prostate	Unmatched	0.067	458.07	0.000	8.1	4.6	61.7*	.84	291	425	716
Prostate	Matched	0.011	12.76	0.994	2.8	1.8	24.5	1.15			
Lung	Unmatched	0.134	47.80	0.006	14.6	8.8	89.7*	.87	131	126	257
Lung	Matched	0.033	11.34	0.991	6.9	5.4	42.2*	1.4			

15 months follow-up cohorts

Cancer	Sample	Pseudo R2	LR-Chi2	P>Chi2	Mean Bias	Med Bias	Rubins' B	Rubins' R	Untreated	Treated	Obs
Colorectal	Unmatched	0.031	214.31	0.000	4.6	2.9	40.9*	1.3	281	308	589
	Matched	0.005	3.92	1.000	1.9	1.5	16	1.32			
Cervical	Unmatched	0.031	213.71	0.000	4.8	3.3	40.8*	1.31	250	279	529
	Matched	0.009	6.87	1.000	3.1	2	22.2	1.28			
Stomach	Unmatched	0.028	192.34	0.000	4.5	2.8	38.8*	1.29	165	190	355
	Matched	0.007	3.70	1.000	2.5	1.7	19.7	1.1			
Breast	Unmatched	0.033	225.16	0.000	4.6	2.9	42.0*	1.27	697	971	1668
	Matched	0.004	10.57	1.000	1.4	1.1	14.7	1.68			
Prostate	Unmatched	0.031	213.71	0.000	4.8	3.3	40.8*	1.31	344	425	769
	Matched	0.004	4.39	1.000	1.5	1.1	14.4	1.01			
Lung	Unmatched	0.135	47.30	0.009	13.1	12.3	90.4*	.87	123	128	251
	Matched	0.02	7.10	1.000	4.9	4.3	33.3*	1.08			

Note: Balance tests are performed using STATA 17 and pstest command created by Leuven and Sianesi (23)*. Pseudo R2 from probit estimation of the conditional propensity score on all the variables in varlist on raw samples, matched samples before and after matching. LR p-values tests joint insignificance of all the regressors before and after matching. The Mean Bias and Median Bias summarize the distribution of the abs(%bias) in appendix 3. Rubins' B is the absolute standardized difference of the means of the linear index of the propensity score in the treated and (matched) non-treated group and Rubin's R is the ratio of treated to (matched) non-treated variances of the propensity score index. Rubin* (24) *recommends that B<25 and that R [0.5-2] to be considered sufficiently balanced. * means that fall outside those limits.*

Annex 5. Additional results

A5.1. Propensity Score Matching

Breast cancer is where the most effects were observed. In the extensive margin, there is a reduction in the probability of receiving a quadrantectomy in both cohort groups (2YC and 15MC), with a decrease of 4 percentage points (S.E. 2 p.p.) compared to a Potential Outcome Mean (P.O. Mean) of 21%. Conversely, there is an increase in the probability of receiving monotherapy, with an increase of 10 to 7 percentage points (S.E. 2 p.p. approx.) in the probability of receiving this treatment, compared to a P.O. Mean of 22%.

In the intensive margin, the direction of these two results is maintained. For quadrantectomies, the frequency of use per patient decreases by 0.05-
0.06 (S.E. 0.02) compared to a P.O. Mean of 0.246, equivalent to a reduction of approximately 20-24%, depending on the cohort group. For monotherapies, the frequencies increase by 0.72 (S.E. 0.173) in the 2YC cohort group and 0.24 (S.E. 0.08) in the 15MC group, equivalent to an increase of 60% and 40%, respectively. Additionally, there are increases in the frequency of use for palliative care consultations (55% 2YC, 28% 15MC), follow-up consultations (96% 2YC, 27% 15MC), dissemination procedures (43% 2YC, 13% 15MC), a reduction in emergency consultations (-32% 2YC, -23% 15MC), and mixed results for teletherapy (29% 2YC, -10% 15MC) and radiotherapy (16% 2YC, -7% 15MC).

In the real expenditure case, only a significant reduction in expenditure per patient for quadrantectomies of 47% in the 15MC group was found. For monotherapies, there is an increase in expenditure of 66% and 90% in the 2YC and 15MC groups, respectively. In other services, an increase in real expenditure is observed in diagnostic imaging (36% 2YC, 18% 15MC), laboratory tests (78% 2YC, 29% 15MC), and in the medication tamoxifen (+170% 2YC, 92% 15MC). Finally, the expenditure per patient for all services increases by 18% in the 2YC group and 9% in the 15MC group.

Other results for breast cancer show effects only in the 2YC cohort. In this case, there is an increase in the number of people consulting for radiology (8 p.p. compared to P.O. Mean 55%), follow-up (9 p.p. compared to P.O. Mean 80%), screening (6 p.p. compared to P.O. Mean 24%), laboratory tests (4 p.p. compared to P.O. Mean 83%), teletherapy (15 p.p. compared to P.O. Mean 52%), and radiotherapy (11 p.p. compared to P.O. Mean 72%). In terms of frequencies, these increase for surgery consultations (61%), radiology consultations (16%), mammograms (18%), and tamoxifen prescriptions (21%). Regarding real expenditure, this increases by approximately 70% for surgery and radiology consultations, 47% for screening and dissemination processes, 22% for mammograms, 44% for teletherapy, and 24% for radiotherapy. In terms of real expenditure on cancer-specific procedures, there was a 30% increase.

For cervical, in the extensive margin, there is an increase in the probability of having a conization by 8 percentage points (S.E. 0.03) in 2YC and 6 percentage points (S.E. 0.03) in 15MC, which is significant considering that the P.O. Mean is around 11%. For hysterectomies, the results are mixed: an increase of 10 percentage points (S.E. 0.03) in 15MC, with a P.O. Mean of 9%, but a reduction of 4 percentage points (S.E. 0.01) in 2YC, with a
P.O. Mean of 24%.

In terms of frequencies (intensive margin), there is only a reduction in brachytherapy (43% 2YC, 33% 15MC), while increases are observed in conization (92% 2YC, 61% 15MC), palliative care consultations (127% 2YC, 110% 15MC), follow-up consultations (50% 2YC, 24% 15MC), and morphine prescriptions (104% 2YC, 112% 15MC). Hysterectomy shows mixed results (-17% 2YC, 120% 15MC).

Using the standardized real expenditure variable, increases are observed in follow-up consultations (62% 2YC, 22% 15MC), spread procedures (83% 2YC, 52% 15MC), and morphine (277% 2YC, 208% 15MC). Hysterectomy (-29% 2YC, 118% 15MC) and diagnostic imaging (84% 2YC, - 27% 15MC) have mixed results.

Other results for cervical cancer show effects only in the 2YC cohort. There is an increase in the probability of

surgical consultation (3 percentage points), palliative care consultation (8 percentage points), and teletherapy (21 percentage points), which are substantial increases relative to the P.O. Mean of each. In the intensive margin, there is an increase in the frequencies per patient of spread procedures (24%) and teletherapy (156%). In terms of expenditure per patient, there is an increase in teletherapy (184%) and radiotherapy (75%), and an overall increase in health care expenditure per patient by 29% and cancer care expenditure by 37%.

For Colorectal, in the extensive margin, there are no significant differences in the demand for any of the services analyzed. In the intensive margin, there is an increase in the frequency of surgical consultations (147% 2YC, 88% 15MC) and follow-up consultations (48% 2YC, 17% 15MC). For laboratory tests, the results are mixed (15% 2YC, -14% 15MC). Regarding real expenditure per patient, there is an increase in spending on colonoscopies (34% 2YC, 59% 15MC), surgical consultations (133% 2YC, 66% 15MC), and a significant increase in spending on morphine (150% 2YC, 387% 15MC).

Additionally, there are some services where effects are observed only in the 2YC cohort. There is a 60% increase in the frequency per patient of colectomies, a 66% increase in palliative care consultations, a 26% increase in dissemination procedures, and a 115% increase in home care, while emergency consultations decrease by 31%. In terms of real expenditure, there is a 97% increase in colectomies, a 72% increase in diagnostic imaging, a 41% increase in laboratory tests, and a 156% increase in teletherapy. Finally, the expenditure on cancer care-specific procedures increased by 26%.

For Stomach, in the extensive margin, there is an increase in the probability of having a surgical consultation (11 percentage points for 2YC and 15MC), which is significant given a P.O. Mean of 24%. Conversely, there is a reduction in the probability of having a follow-up consultation (-11 percentage points for 2YC, -8 percentage points for 15MC) against a P.O. Mean of approximately 80%. The frequency of emergency consultations decreased by -32% in 2YC and -24% in 15MC. In terms of real expenditure per patient, there is only an increase in spending on surgical consultations (126% 2YC, 100% 15MC).

Significant effects are observed only in the 2YC cohort, with a 52% increase in the frequency of follow-up consultations and an 88% increase in spending on laboratory tests. In the 15MC cohort, there is an increase in the probability of having a gastrectomy by 10 percentage points over a P.O. Mean of 18% and a 78% increase in the frequency of these procedures.

For Prostate, in the intensive margin, there is an increase in the probability of receiving brachytherapy by 3 percentage points over a P.O. Mean of 1%. For monotherapy, there is a reduction of -6 percentage points in 2YC and an increase of 6 percentage points in 15MC, over a P.O. Mean of 32% and 14%, respectively. The frequency per patient of brachytherapy increased by 320% in 2YC, relative to a small P.O. Mean of 0.01 per patient. The frequency of follow-up consultations also increased by approximately 40%. Polytherapy reduced their frequency of use (43% 2YC, 30% 15MC),

while for radiotherapies, the results were mixed (-16% 2YC, 25% 15MC). In terms of real expenditure per patient, there was a significant and substantial increase in spending on brachytherapy (342 thousand COP compared to a P.O. Mean of 28 thousand COP in 2YC) and monotherapies (91% 2YC, 375% 15MC). There were also positive effects only in the 2YC cohort in spending on teletherapy (40%) and radiotherapy per patient (52%).

Finally, in the case of lung, there is an increase only in the frequency of follow-up consultations (122% in 2YC, 48% in 15MC), the spending per person on these consultations (121% in 2YC, 62% in 15MC), and the expenditure on diagnostic imaging (187% in 2YC, 75% in 15MC). For the 15MC cohort, there is also an increase in the probability of having outpatient procedures by 19 percentage points relative to a P.O. Mean of 42%.

Table A5.1.1. Robustness checks: alternative specifications of the impact of COVID-19 on breast cancer outcomes in Colombia, propensity score–matched estimates, 2018–2021

	Cohort - 2017-S2 (720 days forward)				Cohort - 2018-S2 (450 days forward)			
Outcomes	ATT		S.E.	P.O. mean	ATT		S.E.	P.O. mean
Brachytherapy (binary)	-0.011		0.007	0.024	-0.012		0.008	0.024
Surgical consultation (binary)	0.038		0.025	0.490	0.028		0.023	0.439
Palliative care consultation (binary)	0.048	**	0.023	0.167	0.025		0.018	0.127
Radiology consultation (binary)	0.083	***	0.018	0.556	-0.003		0.028	0.575
Follow-up consultation (binary)	0.094	***	0.003	0.798	0.032		0.021	0.817
Quadrantectomy (binary)	-0.044	**	0.021	0.213	-0.053	**	0.022	0.212
Screening (binary)	0.061	***	0.023	0.245	0.014		0.023	0.251
Spread procedures (binary)	0.034	*	0.018	0.889	0.036	*	0.021	0.862
Diagnostic imaging (binary)	-0.006		0.011	0.809	-0.020		0.025	0.774
Inpatient services (binary)	0.006		0.024	0.213	-0.034		0.022	0.195
Laboratory tests (binary)	0.038	***	0.004	0.837	0.031		0.022	0.816
Mammography (binary)	0.030	**	0.013	0.773	0.020		0.027	0.630
Mastectomy (binary)	-0.034		0.023	0.196	0.031	*	0.016	0.120
Monoterapy (binary)	0.108	***	0.017	0.279	0.073	***	0.023	0.226
Polyterapy (binary)	0.047	*	0.025	0.533	-0.013		0.025	0.581
Breast reconstruction (binary)	-0.017		0.014	0.387	0.038		0.024	0.297
Teletherapy (binary)	0.151	***	0.021	0.520	-0.021		0.028	0.600
Radiotherapy (binary)	0.115	***	0.011	0.719	0.025		0.024	0.789
Brachytherapy Frequency	-0.007		0.008	0.024	-0.008		0.008	0.024
Surgical consultation Frequency	0.925	***	0.127	1.500	0.072		0.134	1.652
Palliative care consultation Frequency	0.169	***	0.057	0.308	0.049	*	0.029	0.174
Radiology consultation Frequency	0.149	***	0.034	0.886	0.021		0.044	0.730
Follow-up consultation Frequency	4.056	***	0.287	4.214	1.164	***	0.218	4.286
Quadrantectomy Frequency	-0.051	*	0.027	0.248	-0.060	**	0.026	0.246
Screening Frequency	0.073	*	0.042	0.359	0.031		0.036	0.331
Spread procedures Frequency	8.675	***	1.151	19.823	2.424	***	0.876	17.793
Diagnostic imaging Frequency	-0.018		0.149	3.102	-0.055		0.106	2.141
Inpatient services Frequency	-0.152		0.120	0.627	-0.076		0.056	0.386
Laboratory tests Frequency	0.315		0.212	3.936	0.145		0.140	2.969
Mammography Frequency	0.434	***	0.103	2.312	0.065		0.082	1.364
Mastectomy Frequency	-0.024		0.025	0.202	0.033	*	0.019	0.130
Monoterapy Frequency	0.722	***	0.173	1.217	0.243	***	0.082	0.622
Polyterapy Frequency	0.114		0.266	4.153	-0.594	**	0.239	4.406
Breast reconstruction Frequency	-0.036		0.040	0.515	0.052	*	0.030	0.355
Teletherapy Frequency	0.195	***	0.037	0.663	-0.079	*	0.040	0.787
Radiotherapy Frequency	1.025	***	0.323	6.056	-0.438	*	0.266	5.839
Emergency consultations frequency	-0.549	***	0.122	1.685	-0.255	***	0.097	1.074
Home care frequency	0.165		0.112	0.448	0.081		0.075	0.251
Anastrozol Rx frequency	-0.229		0.173	0.726	-0.001		0.068	0.196
Morfina Rx frequency	-0.026		0.029	0.108	-0.009		0.015	0.054
Tamoxifeno Rx frequency	0.967	***	0.291	4.585	0.087		0.177	2.246
Trastuzumab Rx frequency	-0.096		0.222	0.713	0.200	***	0.075	0.221
Brachytherapy real expenditure	-21194.008		24421.121	72494.109	#########		26780.822	78899.609
Brachytherapy real expenditure (stand.)	-3,842		17,407	43,690	-14,118		19,214	53,966
Surgical consultation real expenditure	45,977	***	6,155	71,906	3,722		7,280	88,673
Surgical consultation real expenditure (stand.)	59,173	***	12,346	82,058	5,859		13,862	103,953
Palliative care consultation real expenditure	10,287	***	3,502	18,796	3,321		2,020	12,315
Palliative care consultation real expenditure (stand.)	11,542	**	5,588	31,457	2,300		2,984	18,933
Radiology consultation real expenditure	7,635	***	1,897	39,048	1,218		2,091	34,253
Radiology consultation real expenditure (stand.)	4,405		4,630	43,204	-352		2,323	34,122
Follow-up consultation real expenditure	192,808	***	13,641	205,483	56,911	***	10,755	221,366
Follow-up consultation real expenditure (stand.)	212,280	***	19,783	290,150	22,504		18,510	313,260
Quadrantectomy real expenditure	-4,263		17,466	151,301	-43,024	**	21,542	198,544
Quadrantectomy real expenditure (stand.)	40,706		49,341	299,260	-297,345	***	92,136	625,934
Screening real expenditure	39,095	**	15,178	133,528	15,109		14,840	140,335
Screening real expenditure (stand.)	95,330	***	35,418	192,930	39,634		30,022	201,732
Spread procedures real expenditure	437,018	***	73,481	1,097,174	-32,823		68,572	1,246,662

Spread procedures real expenditure (stand.)	656,250	***	121,028	1,400,198	44,951		106,752	1,557,350
Diagnostic imaging real expenditure	210,744	***	52,649	458,161	67,205	*	39,403	346,633
Diagnostic imaging real expenditure (stand.)	195,506	***	55,520	531,808	72,951	*	42,784	395,452
Inpatient services real expenditure	26,461		71,798	432,831	-123,483		76,519	415,482
Inpatient services real expenditure (stand.)	228,630		202,305	912,563	-527,261	**	234,994	1,151,216
Laboratory tests real expenditure	472,895	***	88,960	1,002,658	143,749		98,597	1,146,945
Laboratory tests real expenditure (stand.)	874,927	***	115,902	1,112,654	398,750	***	139,757	1,369,888
Mammography real expenditure	39,931	**	16,648	229,227	14,232		14,935	168,388
Mammography real expenditure (stand.)	54,915	***	18,283	240,522	14,822		16,458	183,356
Mastectomy real expenditure	-124,020	***	44,201	335,326	29,921		31,603	196,892
Mastectomy real expenditure (stand.)	-113,991		70,967	470,173	-5,625		66,709	338,657
Monoterapy real expenditure	261,011	***	34,292	184,475	80,083	***	16,632	109,343
Monoterapy real expenditure (stand.)	1,352,626	**	620,093	2,043,669	762,523	**	313,444	841,087
Polyterapy real expenditure	76,976		164,434	2,568,143	-508,940	**	208,522	3,851,770
Polyterapy real expenditure (stand.)	96,531		898,277	7,418,380	-1,733,114	**	794,377	8,221,524
Breast reconstruction real expenditure	1,640		111,095	996,911	118,180		72,577	743,833
Breast reconstruction real expenditure (stand.)	132,998		160,000	1,283,550	-61,968		138,819	1,183,549
Teletherapy real expenditure	2,007,447	***	281,336	4,095,615	-161,647		290,975	5,024,157
Teletherapy real expenditure (stand.)	1,961,307	***	320,439	4,405,748	-624,967		380,292	5,688,960
Radiotherapy real expenditure	2,331,082	***	386,593	6,920,728	-618,104		402,830	9,064,170
Radiotherapy real expenditure (stand.)	3,413,199	***	1,228,250	13,911,487	-1,609,676		1,021,826	14,805,536
Emergency consultations real expenditure per person	-68,662	***	26,111	171,827	-12,950		16,252	98,154
Emergency consultations real expenditure per person (stand.)	-409,964		330,046	629,300	-54,499		44,951	218,683
Home care real expenditure per person	28,100		35,594	134,514	13,199		22,078	62,991
Home care real expenditure per person (stand.)	-4,319		53,640	238,358	-40,097		38,623	161,208
Impatient care real expenditure per person	473,849		391,697	3,057,248	-163,481		358,800	3,067,042
Impatient care real expenditure per person (stand.)	-95,028		548,886	5,390,301	-1,059,371	**	445,432	4,919,390
Cancer treatment real expenditure per person	-650,092		1,326,399	7,612,580	723,118		485,886	5,537,136
Cancer treatment real expenditure per person (stand.)	-644,340		1,947,300	16,363,464	375,129		987,530	11,157,093
Anastrozol Rx real expenditure per person	-20,440		12,557	38,117	-6,346		7,567	15,963
Anastrozol Rx real expenditure per person (stand.)	-19,023	*	10,320	39,494	-3,324		5,602	13,841
Morfina Rx real expenditure per person	-113		551	1,491	-260		302	885
Morfina Rx real expenditure per person (stand.)	455		664	1,683	-611		433	1,315
Tamoxifeno Rx real expenditure per person	7,558	**	3,086	46,261	-965		2,196	26,886
Tamoxifeno Rx real expenditure per person (stand.)	45,687	***	4,217	26,695	15,198	***	2,463	16,517
Trastuzumab Rx real expenditure per person	-502,803		1,203,922	3,856,044	1,088,488	***	410,838	1,199,846
Trastuzumab Rx real expenditure per person (stand.)	-815,097		1,492,437	4,828,376	1,446,411	***	489,186	1,352,424
Real expenditure per person	3,941,685		2,427,460	39,896,844	861,423		1,447,321	31,212,272
Real expenditure per person (procedures only)	6,295,807	***	1,570,127	25,842,544	-1,705,175		1,263,216	25,848,468
Real expenditure per person (drugs only)	-1,072,571		1,832,870	11,262,477	2,224,387	***	750,806	4,756,187
Real expenditure per person (stand.)	4,599,719	***	1,700,235	24,856,782	1,859,456	**	941,007	21,167,384
Real expenditure per person (stand., procedures only)	4,428,443	***	602,329	15,738,567	-97,361		610,631	16,919,476
Real expenditure per person (stand., drugs only)	224,738		1,468,632	8,867,599	1,927,143	***	670,954	4,155,141
Cancer care real expenditure per person	3,806,403	*	2,110,563	27,755,780	-503,163		1,258,177	24,458,752
Cancer care real expenditure per person (procedures only)	5,899,985	***	1,362,031	20,854,052	-1,845,991		1,177,672	22,361,016
Cancer care real expenditure per person (drugs only)	-2,093,582		1,659,330	6,901,729	1,342,828	**	526,063	2,097,737
Cancer care real expenditure per person (stand.)	2,712,627	*	1,437,346	17,002,624	836,390		667,500	15,652,981
Cancer care real expenditure per person (stand., procedures only)	3,701,119	***	465,265	12,142,053	-312,897		507,395	14,073,674
Cancer care real expenditure per person (stand., drugs only)	-988,492		1,283,670	4,860,572	1,149,288	***	423,741	1,579,307

*Note: Each row reports the Average Treatment Effect on the Treated (ATT) comparing patients exposed to the COVID-19 period (2020–2021) with matched pre-pandemic patients in Colombia. Estimates are obtained using nearest-neighbor propensity score matching with replacement, implemented with the STATA module PSMATCH2 (Leuven and Sianesi, 2018). Outcomes include measures of service use (extensive and intensive margins) and real expenditure per patient. The "P.O. mean" corresponds to the mean outcome in the matched pre-COVID cohort. Robust standard errors are reported in parentheses. *, **, *** denote statistical significance at the 10%, 5%, and 1% levels, respectively.*

Table A5.1.2. Robustness checks: alternative specifications of the impact of COVID-19 on cervical cancer outcomes in Colombia, propensity score–matched estimates, 2018–2021

	Cohort - 2017-S2 (720 days forward)				Cohort - 2018-S2 (450 days forward)			
Outcomes	ATT		S.E.	P.O. mean	ATT		S.E.	P.O. mean
Brachytherapy (binary)	0.006		0.038	0.299	-0.048		0.041	0.349
Cervicovaginal cytology (binary)	0.027		0.032	0.614	-0.080	*	0.045	0.471
Conization (binary)	0.086	**	0.035	0.104	0.063	**	0.028	0.127
Surgical consultation (binary)	0.030	***	0.010	0.002	0.012		0.009	0.006
Palliative care consultation (binary)	0.085	***	0.028	0.087	0.028		0.028	0.119
Radiology consultation (binary)	0.061		0.038	0.337	0.038		0.039	0.353
Follow-up consultation (binary)	-0.069	**	0.031	0.894	-0.049		0.034	0.805
Screening (binary)	0.062	**	0.027	0.164	-0.016		0.034	0.166
Spread procedures (binary)	0.019		0.047	0.874	-0.007		0.035	0.842
Hysterectomy (binary)	-0.041	***	0.010	0.238	0.106	***	0.034	0.088
Diagnostic imaging (binary)	-0.036		0.048	0.710	-0.017		0.041	0.612
Inpatient services (binary)	-0.066	*	0.037	0.392	0.042		0.037	0.223
Laboratory tests (binary)	-0.043		0.058	0.703	0.013		0.047	0.549
Monoterapy (binary)	0.040	**	0.017	0.028	0.013		0.019	0.037
Polyterapy (binary)	-0.034		0.039	0.356	-0.023		0.038	0.331
Teletherapy (binary)	0.219	***	0.034	0.154	-0.029		0.038	0.395
Radiotherapy (binary)	0.030		0.037	0.418	0.009		0.039	0.424
Brachytherapy Frequency	-0.267	*	0.145	0.607	-0.169	**	0.073	0.505
Cervicovaginal cytology Frequency	0.051		0.071	1.135	-0.069		0.060	0.581
Conization Frequency	0.104	***	0.039	0.111	0.082	***	0.032	0.133
Surgical consultation Frequency	0.052	**	0.020	0.002	0.017		0.010	0.008
Palliative care consultation Frequency	0.241	**	0.103	0.189	0.153	**	0.063	0.138
Radiology consultation Frequency	0.130		0.127	0.637	-0.004		0.077	0.596
Follow-up consultation Frequency	1.394	***	0.324	2.778	0.562	***	0.191	2.259
Screening Frequency	0.077		0.050	0.228	-0.036		0.046	0.208
Spread procedures Frequency	2.389	***	0.891	9.855	0.491		0.694	7.667
Hysterectomy Frequency	-0.041	***	0.010	0.238	0.106	***	0.034	0.088
Diagnostic imaging Frequency	0.260		0.181	1.500	-0.091		0.118	1.241
Inpatient services Frequency	0.023		0.178	0.995	0.298	**	0.122	0.451
Laboratory tests Frequency	-0.875	*	0.481	2.921	0.028		0.246	1.441
Monoterapy Frequency	0.036		0.031	0.053	-0.049		0.093	0.120
Polyterapy Frequency	0.186		0.164	0.842	-0.191		0.185	0.980
Teletherapy Frequency	0.256	***	0.039	0.164	-0.015		0.048	0.420
Radiotherapy Frequency	0.210		0.247	1.668	-0.423		0.284	2.025
Emergency consultations frequency	-0.599		0.368	2.585	-0.058		0.225	1.574
Home care frequency	-0.114		0.338	0.795	0.265	**	0.107	0.162
Carboplatino Rx frequency	-0.025		0.059	0.158	-0.089		0.099	0.121
Morfina Rx frequency	0.166	**	0.081	0.160	0.116	**	0.056	0.103

Paclitaxel Rx frequency	0.006		0.059	0.119	-0.047		0.061	0.076
Brachytherapy real expenditure	-373,897		407,456	1,821,004	-391,284		265,344	1,938,004
Brachytherapy real expenditure (stand.)	63,701		239,607	1,792,460	-209,007		273,765	2,035,620
Cervicovaginal cytology real expenditure	513		996	16,340	-966		871	8,251
Cervicovaginal cytology real expenditure (stand.)	2,432		1,479	16,877	-187		1,032	8,669
Conization real expenditure	-38,815		67,851	177,937	54,516	**	21,220	88,875
Conization real expenditure (stand.)	-36,794		69,098	194,411	70,640	***	24,021	86,977
Surgical consultation real expenditure	3,131	**	1,304	125	983		600	491
Surgical consultation real expenditure (stand.)	3,247	***	1,099	47	975		808	628
Palliative care consultation real expenditure	13,353	**	6,204	11,459	8,672	**	4,182	9,767
Palliative care consultation real expenditure (stand.)	20,314	**	10,139	16,111	11,818		8,506	15,750
Radiology consultation real expenditure	5,922		5,980	28,388	-232		3,644	28,160
Radiology consultation real expenditure (stand.)	8,458		7,648	33,179	3,169		4,253	27,468
Follow-up consultation real expenditure	62,474	***	15,231	132,454	25,266	***	9,276	109,154
Follow-up consultation real expenditure (stand.)	96,245	***	20,813	152,933	32,767	**	16,158	144,459
Screening real expenditure	7,921		4,978	22,692	-7,888		6,399	25,848
Screening real expenditure (stand.)	71,454		54,879	19,930	44,596		56,579	32,278
Spread procedures real expenditure	244,435	***	74,519	477,970	116,439	**	57,235	414,365
Spread procedures real expenditure (stand.)	449,543	***	136,340	540,812	234,247	**	106,531	450,410
Hysterectomy real expenditure	-335,843	***	50,626	754,321	244,630	***	80,264	202,159
Hysterectomy real expenditure (stand.)	-213,948	***	60,281	718,676	270,904	**	107,175	229,025
Diagnostic imaging real expenditure	387,841	***	102,751	358,112	-127,866		99,712	593,303
Diagnostic imaging real expenditure (stand.)	364,594	***	109,292	432,393	-194,209	*	110,491	700,242
Inpatient services real expenditure	197,521		153,685	750,204	347,212	***	123,614	389,272
Inpatient services real expenditure (stand.)	179,643		448,515	1,829,818	-138,966		536,844	1,700,423
Laboratory tests real expenditure	-61,668		52,211	203,876	13,026		17,147	94,606
Laboratory tests real expenditure (stand.)	13,529		34,361	195,455	54,071	**	25,178	113,150
Monoterapy real expenditure	12,323	**	5,394	7,347	-3,267		14,254	18,302
Monoterapy real expenditure (stand.)	58,765	**	29,584	23,326	19,496		40,549	52,877
Polyterapy real expenditure	113,879		100,701	516,476	-165,724		160,099	848,253
Polyterapy real expenditure (stand.)	512,868	**	245,235	744,970	4,538		223,377	937,876
Teletherapy real expenditure	2,167,345	***	312,840	941,708	-272,033		419,226	3,205,073
Teletherapy real expenditure (stand.)	2,183,906	***	370,244	1,180,533	-496,169		574,804	3,687,344
Radiotherapy real expenditure	1,910,126	***	552,725	3,296,060	-832,308		681,165	6,009,633
Radiotherapy real expenditure (stand.)	2,817,990	***	598,901	3,742,538	-681,141		837,805	6,713,717
Emergency consultations real expenditure per person	-26,085		45,370	215,266	36,110		35,358	124,721
Emergency consultations real expenditure per person (stand.)	35,878		103,604	479,860	-163,892		227,753	613,692
Home care real expenditure per person	-16,705		87,338	212,047	68,658	*	41,139	55,836
Home care real expenditure per person (stand.)	-28,213		153,234	440,199	122,890		109,875	153,796
Impatient care real expenditure per person	256,132		541,887	3,033,515	825,666	**	345,849	1,823,233
Impatient care real expenditure per person (stand.)	616,776		889,720	4,988,707	488,322		871,467	4,061,678
Cancer treatment real expenditure per person	85,661		128,084	660,463	-212,246		194,763	949,489
Cancer treatment real expenditure per person (stand.)	502,426	*	256,894	914,421	-62,054		239,311	1,120,838
Carboplatino Rx real expenditure per person	-4,845		10,279	28,363	-18,304		20,658	25,269
Carboplatino Rx real expenditure per person (stand.)	-10,986		12,711	31,643	-32,152		29,047	36,933
Morfina Rx real expenditure per person	2,639	*	1,537	2,824	1,294		1,105	2,091
Morfina Rx real expenditure per person (stand.)	7,816	**	3,143	2,819	5,251	*	2,694	2,523
Paclitaxel Rx real expenditure per person	8,818		28,252	45,324	-14,732		20,208	24,239
Paclitaxel Rx real expenditure per person (stand.)	-20,018		13,965	38,666	-28,952		25,960	33,414
Real expenditure per person	5,162,127	***	1,549,412	14,151,944	-3,736		1,424,890	15,397,109
Real expenditure per person (procedures only)	4,643,820	***	1,321,451	12,149,832	-563,551		1,346,484	14,415,760
Real expenditure per person (drugs only)	50,820		276,843	1,314,600	106,494		157,768	672,906
Real expenditure per person (stand.)	2,655,718	**	1,147,057	10,433,319	-351,916		881,998	11,052,684
Real expenditure per person (stand., procedures only)	2,684,024	***	969,216	9,204,985	-250,855		841,703	10,342,414
Real expenditure per person (stand., drugs only)	3,074		237,192	1,062,161	-119,968		128,354	646,876
Cancer care real expenditure per person	4,145,406	***	913,108	8,275,230	-140,293		1,018,814	10,777,761
Cancer care real expenditure per person (procedures only)	4,214,612	***	904,799	8,129,106	-54,204		999,785	10,647,676
Cancer care real expenditure per person (drugs only)	-69,206	*	40,809	146,125	-86,089		56,498	130,085

Cancer care real expenditure per person (stand.)	2,436,406	***	724,044	6,599,643	-105,150		736,287	8,330,842
Cancer care real expenditure per person (stand., procedures only)	2,476,947	***	707,200	6,463,004	-61,895		715,915	8,247,908
Cancer care real expenditure per person (stand., drugs only)	-40,541		48,368	136,640	-43,255		43,935	82,934

*Note: Each row reports the Average Treatment Effect on the Treated (ATT) comparing patients exposed to the COVID-19 period (2020–2021) with matched pre-pandemic patients in Colombia. Estimates are obtained using nearest-neighbor propensity score matching with replacement, implemented with the STATA module PSMATCH2 (Leuven and Sianesi, 2018). Outcomes include measures of service use (extensive and intensive margins) and real expenditure per patient. The "P.O. mean" corresponds to the mean outcome in the matched pre-COVID cohort. Robust standard errors are reported in parentheses. *, **, *** denote statistical significance at the 10%, 5%, and 1% levels, respectively.*

Table A5.1.3. Robustness checks: alternative specifications of the impact of COVID-19 on colorectal cancer outcomes in Colombia, propensity score–matched estimates, 2018–2021

	Cohort - 2017 -S2 (720 days forward)				Cohort - 2018-S2 (450 days forward)			
Outcomes	ATT		S.E.	P.O. mean	ATT		S.E.	P.O. mean
Colectomy (binary)	0.085	**	0.038	0.185	0.031		0.036	0.228
Colonoscopy (binary)	-0.070		0.046	0.583	-0.078	*	0.044	0.465
Surgical consultation (binary)	0.237	***	0.045	0.374	0.115	***	0.044	0.427
Palliative care consultation (binary)	0.095	**	0.039	0.218	0.003		0.033	0.228
Radiology consultation (binary)	0.051		0.032	0.205	0.021		0.035	0.206
Follow-up consultation (binary)	0.016		0.038	0.854	-0.011		0.035	0.812
Spread procedures (binary)	0.003		0.030	0.905	0.006		0.032	0.858
Diagnostic imaging (binary)	0.009		0.040	0.826	0.003		0.035	0.750
Inpatient services (binary)	-0.010		0.041	0.540	-0.046		0.043	0.471
Laboratory tests (binary)	0.014		0.036	0.849	-0.003		0.033	0.805
Monoterapy (binary)	0.033		0.022	0.087	0.004		0.029	0.103
Polyterapy (binary)	-0.038		0.047	0.603	0.004		0.038	0.522
Teletherapy (binary)	0.077	*	0.042	0.151	-0.009		0.035	0.224
Radiotherapy (binary)	0.024		0.049	0.636	-0.014		0.036	0.631
Irinotecan Rx frequency	-0.017		0.063	0.108	0.025		0.016	0.001
Morfina Rx frequency	0.050		0.156	0.392	0.141	**	0.070	0.144
Oxaliplatino Rx frequency	0.098		0.110	0.208	-0.041		0.067	0.197
Colectomy Frequency	0.119	***	0.045	0.197	0.058		0.041	0.244
Colonoscopy Frequency	-0.040		0.093	0.829	-0.077		0.058	0.555
Surgical consultation Frequency	1.152	***	0.168	0.782	0.608	***	0.118	0.691
Palliative care consultation Frequency	0.261	***	0.092	0.395	0.040		0.054	0.331
Radiology consultation Frequency	0.084		0.056	0.273	0.007		0.050	0.276
Follow-up consultation Frequency	1.728	***	0.479	3.574	0.490	*	0.266	2.786
Spread procedures Frequency	7.191	***	2.742	27.216	-2.598		1.744	24.397
Diagnostic imaging Frequency	0.383		0.250	3.143	0.014		0.204	2.233
Inpatient services Frequency	0.266		0.199	1.422	0.014		0.162	1.119
Laboratory tests Frequency	0.958	*	0.570	6.252	-0.732	**	0.369	5.138
Monoterapy Frequency	0.260	**	0.118	0.175	0.046		0.090	0.285
Polyterapy Frequency	-1.219	*	0.716	4.700	-0.476		0.294	3.125
Teletherapy Frequency	0.078		0.049	0.178	0.009		0.038	0.225
Radiotherapy Frequency	-0.881		0.741	5.053	-0.421		0.314	3.635
Emergency consultations frequency	-0.811	***	0.305	2.607	-0.150		0.157	1.582
Home care frequency	1.329	***	0.404	1.152	0.188		0.345	1.471
Colectomy real expenditure	813,889	**	380,766	1,628,683	244,593		372,886	2,265,294
Colectomy real expenditure (stand.)	1,522,159	***	514,472	1,560,796	371,961		611,236	2,693,722
Colonoscopy real expenditure	-11,684		32,044	285,951	-23,898		19,237	183,402
Colonoscopy real expenditure (stand.)	112,898	**	55,862	330,907	152,662	*	82,507	255,150
Surgical consultation real expenditure	68,277	***	10,164	46,390	36,586	***	7,005	39,921
Surgical consultation real expenditure (stand.)	71,197	***	10,941	53,327	34,445	***	8,634	51,637
Palliative care consultation real expenditure	15,240	***	5,514	24,348	2,617		3,711	22,778
Palliative care consultation real expenditure (stand.)	24,857		16,215	41,397	13,478		14,689	33,127
Radiology consultation real expenditure	3,941		2,482	11,826	435		2,334	12,808
Radiology consultation real expenditure (stand.)	7,023	**	3,254	10,669	369		2,812	13,627
Follow-up consultation real expenditure	78,252	***	22,207	170,454	23,246	*	12,677	136,276
Follow-up consultation real expenditure (stand.)	58,390	**	26,577	243,987	-26,702		24,947	235,255

Spread procedures real expenditure	98,440	***	33,590	382,389	-41,649		32,040	356,520
Spread procedures real expenditure (stand.)	96,103	*	49,365	560,409	-57,556		49,191	498,200
Diagnostic imaging real expenditure	795,601	***	151,476	911,820	184,434		135,769	857,448
Diagnostic imaging real expenditure (stand.)	772,115	***	154,335	1,062,946	97,607		149,830	1,021,480
Inpatient services real expenditure	23,042		202,130	1,824,722	-341,331	*	196,866	1,576,548
Inpatient services real expenditure (stand.)	-155,765		1,247,345	4,320,142	-548,298		626,964	3,387,088
Laboratory tests real expenditure	57,150	*	32,603	344,659	-39,721	*	23,793	308,126
Laboratory tests real expenditure (stand.)	172,503	***	46,036	418,435	-32,779		42,470	429,272
Monoterapy real expenditure	74,024	***	25,050	25,432	26,651		17,578	45,456
Monoterapy real expenditure (stand.)	389,846	*	234,004	80,438	32,860		83,813	184,805
Polyterapy real expenditure	-751,630	*	440,022	2,890,848	-414,896		255,598	2,716,159
Polyterapy real expenditure (stand.)	-1,395,029		3,032,594	12,239,105	-836,886		1,565,110	7,545,173
Teletherapy real expenditure	1,132,817	***	315,675	887,583	362,948		285,863	1,437,653
Teletherapy real expenditure (stand.)	1,374,331	***	370,706	878,769	460,257		336,783	1,585,750
Radiotherapy real expenditure	455,210		607,907	3,803,862	-25,298		373,725	4,199,267
Radiotherapy real expenditure (stand.)	369,147		3,089,610	13,198,312	-343,769		1,599,515	9,315,727
Emergency consultations real expenditure per person	-120,035	**	52,667	266,181	4,125		19,433	124,940
Emergency consultations real expenditure per person (stand.)	-280,590	*	156,761	689,225	9,328		76,923	352,684
Home care real expenditure per person	301,843	***	106,746	290,639	71,867		75,254	285,703
Home care real expenditure per person (stand.)	517,645		430,180	860,621	464,572		337,905	636,430
Impatient care real expenditure per person	2,859,771	**	1,171,596	9,057,505	1,438,451		881,357	7,970,327
Impatient care real expenditure per person (stand.)	551,610		2,294,243	17,279,416	-674,174		1,608,578	14,370,713
Cancer treatment real expenditure per person	-530,514		466,789	3,278,603	-386,077		283,254	3,113,130
Cancer treatment real expenditure per person (stand.)	-797,939		3,034,985	12,767,037	-729,587		1,583,648	8,087,829
Irinotecan Rx real expenditure per person	-5,412		26,189	42,247	10,311		7,054	416
Irinotecan Rx real expenditure per person (stand.)	-12,122		43,005	58,350	15,550		10,019	416
Morfina Rx real expenditure per person	1,260		3,829	8,339	4,470	***	1,599	2,093
Morfina Rx real expenditure per person (stand.)	9,519	**	4,460	6,310	8,186	***	3,035	2,110
Oxaliplatino Rx real expenditure per person	-1,708		29,495	68,723	-21,494		19,232	59,044
Oxaliplatino Rx real expenditure per person (stand.)	-2,335		35,890	75,202	-24,158		21,048	67,720
Real expenditure per person	3,389,361		4,763,594	45,479,676	-662,609		2,830,033	34,698,544
Real expenditure per person (procedures only)	3,537,733		4,327,347	36,092,480	-1,409,272		2,732,236	29,467,470
Real expenditure per person (drugs only)	287,079		1,525,261	5,636,645	926,206		637,143	2,871,485
Real expenditure per person (stand.)	4,217,629	*	2,548,844	23,804,350	1,631,078		1,394,203	19,293,898
Real expenditure per person (stand., procedures only)	4,120,648	**	1,770,456	18,363,842	718,570		1,140,746	16,656,849
Real expenditure per person (stand., drugs only)	-82,452		1,498,765	5,121,475	830,767		640,085	2,443,831
Cancer care real expenditure per person	3,607,058		3,853,766	22,671,946	-244,661		2,014,638	18,985,338
Cancer care real expenditure per person (procedures only)	3,393,908		3,859,597	22,224,374	-322,764		2,006,290	18,627,444
Cancer care real expenditure per person (drugs only)	213,150		138,995	447,571	78,103		80,187	357,894
Cancer care real expenditure per person (stand.)	2,752,487	***	973,609	10,100,132	-96,903		727,065	10,914,254
Cancer care real expenditure per person (stand., procedures only)	2,602,779	***	951,704	9,737,771	-101,742		713,564	10,562,701
Cancer care real expenditure per person (stand., drugs only)	149,708		100,767	362,361	4,838		77,096	351,553

*Note: Each row reports the Average Treatment Effect on the Treated (ATT) comparing patients exposed to the COVID-19 period (2020–2021) with matched pre-pandemic patients in Colombia. Estimates are obtained using nearest-neighbor propensity score matching with replacement, implemented with the STATA module PSMATCH2 (Leuven and Sianesi, 2018). Outcomes include measures of service use (extensive and intensive margins) and real expenditure per patient. The "P.O. mean" corresponds to the mean outcome in the matched pre-COVID cohort. Robust standard errors are reported in parentheses. *, **, *** denote statistical significance at the 10%, 5%, and 1% levels, respectively.*

Table A5.1.4. Robustness checks: alternative specifications of the impact of COVID-19 on lung cancer outcomes in Colombia, propensity score–matched estimates, 2018–2021

	Cohort - 2017-S2 (720 days forward)			Cohort - 2018-S2 (450 days forward)		
Outcomes	ATT	S.E.	P.O. mean	ATT	S.E.	P.O. mean
Bronchoscopy (binary)	0.014	0.020	0.017	0.014	0.012	0.009
Surgical consultation (binary)	0.022	0.047	0.200	-0.069	0.053	0.272
Palliative care consultation (binary)	-0.017	0.081	0.501	-0.008	0.059	0.414
Radiology consultation (binary)	-0.046	0.057	0.387	-0.050	0.043	0.378
Follow-up consultation (binary)	0.042	0.073	0.712	-0.003	0.054	0.745
Spread procedures (binary)	0.007	0.056	0.794	0.063	0.048	0.742

Diagnostic imaging (binary)	-0.018		0.070	0.716	0.017		0.060	0.670
Inpatient services (binary)	0.036		0.064	0.647	0.194	***	0.069	0.423
Laboratory tests (binary)	0.012		0.074	0.591	0.077		0.066	0.494
Respiratory tests (binary)	-0.069		0.051	0.450	-0.127	**	0.063	0.431
Monoterapy (binary)	0.044		0.056	0.162	0.039		0.042	0.141
Pleurodesis (binary)	0.002		0.009	0.006	-0.025		0.029	0.033
Polyterapy (binary)	-0.080		0.070	0.565	0.019		0.062	0.473
Teletherapy (binary)	0.091		0.067	0.282	0.084		0.066	0.259
Radiotherapy (binary)	-0.002		0.069	0.637	0.067		0.063	0.566
Bronchoscopy Frequency	0.010		0.022	0.022	0.014		0.012	0.009
Surgical consultation Frequency	0.075		0.108	0.330	-0.080		0.112	0.392
Palliative care consultation Frequency	0.037		0.298	1.233	0.020		0.171	0.847
Radiology consultation Frequency	0.028		0.135	0.599	0.039		0.093	0.500
Follow-up consultation Frequency	3.133	***	0.800	2.566	1.236	**	0.566	2.530
Spread procedures Frequency	6.226	**	3.169	18.338	1.948		2.221	14.489
Diagnostic imaging Frequency	0.564		0.428	2.817	0.536	**	0.273	1.855
Inpatient services Frequency	-0.027		0.288	1.844	0.431	*	0.230	1.045
Laboratory tests Frequency	0.917	**	0.367	1.495	0.081		0.344	1.559
Respiratory tests Frequency	-0.035		0.245	1.098	-0.195		0.187	0.867
Monoterapy Frequency	1.095	**	0.496	0.635	0.445		0.275	0.438
Pleurodesis Frequency	-0.005		0.013	0.013	-0.025		0.029	0.033
Polyterapy Frequency	-0.221		0.633	3.491	-0.670		0.521	3.327
Teletherapy Frequency	0.135		0.125	0.420	0.077		0.114	0.400
Radiotherapy Frequency	1.017		0.854	4.546	-0.148		0.635	4.164
Emergency consultations frequency	-1.688	*	0.890	3.418	0.013		0.239	1.362
Home care frequency	0.130		0.570	2.394	0.408		0.436	1.350
Carboplatino Rx frequency	-0.008		0.219	0.437	-0.194		0.168	0.600
Morfina Rx frequency	0.044		0.258	0.813	0.192		0.168	0.456
Paclitaxel Rx frequency	-0.162		0.140	0.281	-0.153		0.113	0.286
Bronchoscopy real expenditure	7527.605		14288.526	13340.323	8994.363		8375.367	7048.012
Bronchoscopy real expenditure (stand.)	45863.184		52849.543	26181.016	53068.352		43527.090	11940.666
Surgical consultation real expenditure	3051.991		4253.910	13366.593	-3958.466		4889.412	18031.242
Surgical consultation real expenditure (stand.)	6215.808		6999.983	20497.508	-1752.376		5952.413	23179.811
Palliative care consultation real expenditure	929.236		18225.713	75999.641	1076.178		11711.584	58431.617
Palliative care consultation real expenditure (stand.)	-1806.783		29543.908	124215.000	-3034.129		21020.869	85195.656
Radiology consultation real expenditure	2433.653		6058.865	25793.777	2318.290		4524.129	23435.305
Radiology consultation real expenditure (stand.)	8,614		8,290	25,019	9,253	*	4,878	20,732
Follow-up consultation real expenditure	143,175	***	37,112	123,048	54,670	**	26,161	124,602
Follow-up consultation real expenditure (stand.)	170,153	***	38,636	140,126	83,368	***	27,202	133,984
Spread procedures real expenditure	166,226		161,343	730,161	147,485		197,407	566,565
Spread procedures real expenditure (stand.)	442,562	*	228,976	818,782	279,672		269,506	689,793
Diagnostic imaging real expenditure	1,371,722	***	282,281	600,359	673,304	**	269,009	746,388
Diagnostic imaging real expenditure (stand.)	1,374,718	***	295,545	732,831	677,286	**	284,000	895,800
Inpatient services real expenditure	-483,806	*	283,351	2,505,391	179,996		363,628	1,682,685
Inpatient services real expenditure (stand.)	-181,752		591,129	4,013,266	237,678		1,168,407	3,289,109
Laboratory tests real expenditure	96,668		74,680	242,798	48,569		38,549	175,401
Laboratory tests real expenditure (stand.)	217,038	**	110,507	268,131	72,828		49,183	202,868
Respiratory tests real expenditure	10,202		18,222	60,076	-6,534		11,959	45,494
Respiratory tests real expenditure (stand.)	-29,679		28,625	104,005	-12,001		18,653	55,630
Monoterapy real expenditure	320,291	***	105,265	95,250	128,370	**	55,050	70,186
Monoterapy real expenditure (stand.)	3,561,609		2,293,073	245,546	2,876,983		2,012,079	147,705
Pleurodesis real expenditure	-66,906		90,394	101,830	-168,313		200,978	222,158
Pleurodesis real expenditure (stand.)	-19,924		56,143	54,292	-126,789		125,958	160,619
Polyterapy real expenditure	-158,729		394,067	2,183,197	-611,989		462,093	2,920,861
Polyterapy real expenditure (stand.)	414,195		885,839	3,202,819	897,028	*	540,153	2,143,266
Teletherapy real expenditure	1,670,717	**	710,622	2,047,831	1,141,397	*	644,897	1,984,852
Teletherapy real expenditure (stand.)	1,351,839	*	774,389	2,220,257	1,021,112		677,502	2,108,970
Radiotherapy real expenditure	1,885,005	**	896,629	4,326,278	657,778		835,995	4,975,899

Radiotherapy real expenditure (stand.)	5,340,377	*	2,850,815	5,668,622	4,795,123	**	2,386,906	4,399,940
Emergency consultations real expenditure per person	-116,469	**	50,645	228,758	-3,244		21,595	104,922
Emergency consultations real expenditure per person (stand.)	-543,598	***	189,022	1,038,092	-168,664		133,778	559,471
Home care real expenditure per person	-112,365		195,943	754,247	10,673		130,898	417,905
Home care real expenditure per person (stand.)	-35,388		332,652	1,230,996	64,419		260,818	833,954
Impatient care real expenditure per person	-2,578,797		1,932,164	9,253,352	-1,069,769	**	501,004	6,906,713
Impatient care real expenditure per person (stand.)	-4,382,738	*	2,566,367	14,027,869	-2,156,589		1,419,086	10,638,095
Cancer treatment real expenditure per person	162,633		420,622	2,468,129	-531,816		501,744	3,253,458
Cancer treatment real expenditure per person (stand.)	4,228,283		2,617,125	3,661,218	3,714,534		2,303,093	2,793,889
Carboplatino Rx real expenditure per person	25,427		48,403	82,116	-3,423		44,302	123,611
Carboplatino Rx real expenditure per person (stand.)	47,016		60,323	86,170	-40,601		54,722	159,800
Morfina Rx real expenditure per person	1,517		6,857	19,392	5,460		4,491	10,748
Morfina Rx real expenditure per person (stand.)	7,766		9,290	19,282	12,154	**	5,878	9,380
Paclitaxel Rx real expenditure per person	-31,214		41,561	84,626	-36,932		50,292	101,233
Paclitaxel Rx real expenditure per person (stand.)	188,888		231,103	103,882	-15,736		254,885	306,310
Real expenditure per person	4,730,724		4,859,224	28,879,180	4,221,373		3,091,145	22,440,266
Real expenditure per person (procedures only)	6,010,547		3,865,110	23,567,830	4,299,755		3,021,651	19,593,988
Real expenditure per person (drugs only)	-1,265,997		1,729,610	4,208,858	-267,560		531,642	2,444,581
Real expenditure per person (stand.)	1,610,307		2,967,330	20,520,456	873,852		1,477,390	16,747,910
Real expenditure per person (stand., procedures only)	2,473,705		2,214,380	17,091,738	1,266,878		1,296,741	14,528,381
Real expenditure per person (stand., drugs only)	-888,010		1,106,659	3,217,617	-399,481		402,244	2,091,988
Cancer care real expenditure per person	7,268,020	**	3,355,885	14,375,179	3,879,706		3,119,796	14,248,265
Cancer care real expenditure per person (procedures only)	7,015,641	**	3,362,313	14,162,226	3,939,183		3,079,315	13,745,347
Cancer care real expenditure per person (drugs only)	252,378		262,540	212,954	-59,477		287,182	502,918
Cancer care real expenditure per person (stand.)	2,801,631	**	1,412,491	10,959,078	1,250,417		1,379,683	10,791,288
Cancer care real expenditure per person (stand., procedures only)	2,800,734	**	1,425,610	10,769,222	1,298,615		1,360,110	10,528,876
Cancer care real expenditure per person (stand., drugs only)	898		82,957	189,856	-48,198		81,551	262,411

*Note: Each row reports the Average Treatment Effect on the Treated (ATT) comparing patients exposed to the COVID-19 period (2020–2021) with matched pre-pandemic patients in Colombia. Estimates are obtained using nearest-neighbor propensity score matching with replacement, implemented with the STATA module PSMATCH2 (Leuven and Sianesi, 2018). Outcomes include measures of service use (extensive and intensive margins) and real expenditure per patient. The "P.O. mean" corresponds to the mean outcome in the matched pre-COVID cohort. Robust standard errors are reported in parentheses. *, **, *** denote statistical significance at the 10%, 5%, and 1% levels, respectively.*

Table A5.1.5. Robustness checks: alternative specifications of the impact of COVID-19 on prostate cancer outcomes in Colombia, propensity score–matched estimates, 2018–2021

	Cohort - 2017-S2 (720 days forward)				Cohort - 2018-S2 (450 days forward)			
Outcomes	ATT		S.E.	P.O. mean	ATT		S.E.	P.O. mean
Brachytherapy (binary)	0.033	***	0.012	0.010	0.037	***	0.010	0.003
Surgical consultation (binary)	0.005		0.008	0.009	-0.008		0.009	0.020
Palliative care consultation (binary)	-0.035		0.034	0.162	0.010		0.022	0.084
Radiology consultation (binary)	0.005		0.039	0.548	0.027		0.035	0.479
Follow-up consultation (binary)	0.036		0.030	0.284	0.032		0.029	0.241
Screening (binary)	-0.011		0.027	0.888	-0.015		0.027	0.841
Spread procedures (binary)	0.003		0.023	0.915	0.019		0.026	0.859
Inpatient services (binary)	0.005		0.041	0.334	0.069	**	0.031	0.202
Laboratory tests (binary)	-0.042		0.033	0.720	-0.013		0.039	0.632
Monoterapy (binary)	-0.064	*	0.034	0.316	0.062	**	0.027	0.138
Polyterapy (binary)	-0.040		0.027	0.134	-0.012		0.015	0.087
Prostatectomy (binary)	0.029		0.028	0.164	0.030		0.028	0.147
Teletherapy (binary)	0.037		0.043	0.410	0.010		0.036	0.390
Radiotherapy (binary)	0.020		0.041	0.554	0.058		0.036	0.457
Brachytherapy Frequency	0.034	***	0.012	0.011	0.039	***	0.011	0.003
Surgical consultation Frequency	0.000		0.011	0.014	-0.031	**	0.015	0.043
Palliative care consultation Frequency	0.040		0.085	0.350	0.106	*	0.054	0.137
Radiology consultation Frequency	0.029		0.127	1.268	0.034		0.082	0.933
Follow-up consultation Frequency	0.486	**	0.242	1.239	0.315	**	0.135	0.728
Screening Frequency	-0.079		0.278	4.778	-0.262	*	0.159	2.931
Spread procedures Frequency	1.365	*	0.815	9.266	-0.053		0.589	6.846

Inpatient services Frequency	-0.001		0.119	0.700	0.076		0.094	0.442
Laboratory tests Frequency	-0.153		0.110	1.358	0.000		0.074	0.972
Monoterapy Frequency	-0.119		0.157	1.310	0.439	***	0.099	0.284
Polyterapy Frequency	-0.338	**	0.155	0.780	-0.160	*	0.094	0.517
Prostatectomy Frequency	0.025		0.029	0.173	0.027		0.028	0.149
Teletherapy Frequency	-0.034		0.066	0.559	-0.001		0.045	0.448
Radiotherapy Frequency	-0.441	*	0.227	2.659	0.319	**	0.145	1.264
Emergency consultations frequency	-0.496		0.302	1.699	-0.069		0.141	0.985
Home care frequency	0.297		0.202	0.579	0.084		0.129	0.373
Docetaxel Rx frequency	-0.078		0.081	0.179	-0.008		0.029	0.077
Morfina Rx frequency	-0.068		0.093	0.146	-0.037		0.044	0.079
Prednisona Rx frequency	-0.016		0.039	0.118	-0.047		0.043	0.123
Brachytherapy real expenditure	101586.828	***	38097.363	32027.594	#########	***	32825.188	10255.494
Brachytherapy real expenditure (stand.)	342722.594	***	104101.688	27912.936	#########	***	#########	2282.214
Surgical consultation real expenditure	-2.611		634.363	811.869	-1782.108	**	856.474	2465.562
Surgical consultation real expenditure (stand.)	98.788		650.133	664.247	-2416.123	**	1038.652	2959.845
Palliative care consultation real expenditure	2759.367		4923.240	20932.369	6767.663	*	3728.815	9710.767
Palliative care consultation real expenditure (stand.)	4,141		6,819	26,926	-2,105		6,783	20,320
Radiology consultation real expenditure	2,828		6,002	56,166	1,875		3,954	44,363
Radiology consultation real expenditure (stand.)	10,493		7,870	66,604	1,190		5,550	55,054
Follow-up consultation real expenditure	21,943	*	11,410	59,252	14,298	**	6,298	35,725
Follow-up consultation real expenditure (stand.)	9,286		11,994	71,281	7,202		9,147	42,084
Screening real expenditure	-3,866		16,970	284,309	-13,791		11,263	189,608
Screening real expenditure (stand.)	65,029	**	26,811	327,829	25,123		15,471	205,474
Spread procedures real expenditure	27,065		27,453	378,002	25,495		28,772	283,781
Spread procedures real expenditure (stand.)	15,375		47,399	459,762	-3,241		57,208	350,494
Inpatient services real expenditure	-72,033		148,551	684,658	29,317		86,749	411,433
Inpatient services real expenditure (stand.)	3,844		409,230	1,499,865	120,529		314,496	938,942
Laboratory tests real expenditure	-86,524		58,437	315,893	94		14,090	197,885
Laboratory tests real expenditure (stand.)	-62,721		60,107	335,628	-5,675		20,495	230,450
Monoterapy real expenditure	76,064	**	33,685	203,761	103,976	***	21,342	55,929
Monoterapy real expenditure (stand.)	393,177	***	135,615	429,463	403,981	***	90,023	107,600
Polyterapy real expenditure	-207,836	**	95,885	487,834	-147,505	*	85,566	471,231
Polyterapy real expenditure (stand.)	-114,726		127,827	564,248	-226,799		167,195	548,682
Prostatectomy real expenditure	-70,347		172,012	1,007,324	-34,641		159,923	825,270
Prostatectomy real expenditure (stand.)	210,327		181,920	893,611	271,846		196,781	732,213
Teletherapy real expenditure	1,165,906	***	447,815	2,938,483	411,170		358,296	3,022,755
Teletherapy real expenditure (stand.)	1,319,341	***	472,848	3,221,937	238,522		450,127	3,559,950
Radiotherapy real expenditure	1,245,143	***	475,027	3,662,104	504,061		381,358	3,637,329
Radiotherapy real expenditure (stand.)	2,225,969	***	582,426	4,243,561	946,323		591,005	4,333,519
Emergency consultations real expenditure per person	-72,215		59,804	197,333	717		16,386	89,193
Emergency consultations real expenditure per person (stand.)	-60,714		110,992	389,307	33,514		36,539	157,331
Home care real expenditure per person	38,397		64,870	172,012	7,283		34,186	89,904
Home care real expenditure per person (stand.)	14,077		119,623	362,643	-95,624		114,124	328,204
Impatient care real expenditure per person	120,152		591,231	3,362,253	367,492		395,057	2,286,311
Impatient care real expenditure per person (stand.)	514,102		1,008,262	5,144,569	1,114,367	*	625,516	3,008,032
Cancer treatment real expenditure per person	73,041		169,584	1,176,639	4,215		124,101	958,280
Cancer treatment real expenditure per person (stand.)	456,890	**	207,171	1,478,096	222,916		196,769	1,076,009
Docetaxel Rx real expenditure per person	-80,366		81,945	177,912	-12,828		29,848	76,003
Docetaxel Rx real expenditure per person (stand.)	-102,924		80,664	179,730	-31,532		38,280	87,232
Morfina Rx real expenditure per person	-343		1,426	2,144	-613		1,163	1,579
Morfina Rx real expenditure per person (stand.)	-1,078		2,991	4,330	265		988	1,142
Prednisona Rx real expenditure per person	-118		162	508	-183		164	464
Prednisona Rx real expenditure per person (stand.)	66		319	701	-128		307	654
Real expenditure per person	2,796,975	*	1,632,999	17,309,036	1,501,947		974,131	12,779,370
Real expenditure per person (procedures only)	2,866,094	**	1,360,752	13,429,344	1,237,322		915,709	10,542,509
Real expenditure per person (drugs only)	155,019		364,708	3,127,862	106,157		220,560	2,005,008
Real expenditure per person (stand.)	1,715,164		1,097,234	13,268,659	530,828		694,083	10,343,725

Real expenditure per person (stand., procedures only)	1,468,850	929,215	10,255,623	464,889	624,891	8,334,699
Real expenditure per person (stand., drugs only)	261,380	317,700	2,885,890	55,309	208,280	1,957,147
Cancer care real expenditure per person	2,660,656 ***	909,806	8,425,787	1,382,516 **	700,673	7,380,978
Cancer care real expenditure per person (procedures only)	2,482,217 ***	876,465	7,941,403	1,334,477 *	691,290	6,961,251
Cancer care real expenditure per person (drugs only)	178,439	118,778	484,384	48,038	76,502	419,727
Cancer care real expenditure per person (stand.)	1,270,479 **	635,615	6,968,890	579,918	432,922	6,088,886
Cancer care real expenditure per person (stand., procedures only)	1,065,665 *	584,010	6,483,846	530,112	413,247	5,657,767
Cancer care real expenditure per person (stand., drugs only)	204,814 *	118,723	485,045	49,806	75,236	431,119

*Note: Each row reports the Average Treatment Effect on the Treated (ATT) comparing patients exposed to the COVID-19 period (2020–2021) with matched pre-pandemic patients in Colombia. Estimates are obtained using nearest-neighbor propensity score matching with replacement, implemented with the STATA module PSMATCH2 (Leuven and Sianesi, 2018). Outcomes include measures of service use (extensive and intensive margins) and real expenditure per patient. The "P.O. mean" corresponds to the mean outcome in the matched pre-COVID cohort. Robust standard errors are reported in parentheses. *, **, *** denote statistical significance at the 10%, 5%, and 1% levels, respectively.*

Table A5.1.6. Robustness checks: alternative specifications of the impact of COVID-19 on stomach cancer outcomes in Colombia, propensity score–matched estimates, 2018–2021

	Cohort - 2017-S2 (720 days forward)			Cohort - 2018-S2 (450 days forward)		
Outcomes	ATT	S.E.	P.O. mean	ATT	S.E.	P.O. mean
Surgical consultation (binary)	0.118 **	0.060	0.247	0.118 **	0.048	0.230
Palliative care consultation (binary)	-0.016	0.065	0.291	-0.004	0.039	0.262
Radiology consultation (binary)	-0.058	0.056	0.258	-0.026	0.040	0.195
Follow-up consultation (binary)	-0.113 ***	0.044	0.838	-0.087 **	0.041	0.776
Screening (binary)	0.025	0.059	0.145	0.041	0.034	0.101
Spread procedures (binary)	-0.075	0.072	0.675	-0.063	0.055	0.626
Gastrectomy (binary)	-0.021	0.053	0.311	0.101 ***	0.039	0.183
Diagnostic imaging (binary)	-0.058	0.061	0.733	-0.032	0.048	0.680
Inpatient services (binary)	0.085	0.071	0.530	0.035	0.053	0.544
Laboratory tests (binary)	0.109 **	0.049	0.251	-0.036	0.057	0.357
Monoterapy (binary)	-0.007	0.038	0.097	0.015	0.027	0.069
Polyterapy (binary)	-0.042	0.055	0.607	-0.031	0.049	0.578
Teletherapy (binary)	-0.068	0.043	0.238	-0.040	0.038	0.192
Radiotherapy (binary)	-0.063	0.058	0.653	-0.040	0.049	0.614
Palliative care consultation Frequency	0.052	0.166	0.583	0.071	0.096	0.418
Radiology consultation Frequency	-0.080	0.083	0.385	-0.043	0.060	0.280
Follow-up consultation Frequency	1.497 ***	0.476	2.843	-0.020	0.390	3.283
Screening Frequency	-0.021	0.105	0.206	0.046	0.037	0.112
Spread procedures Frequency	-0.178	0.195	1.833	-0.355 **	0.178	1.592
Gastrectomy Frequency	0.029	0.059	0.331	0.151 ***	0.051	0.191
Diagnostic imaging Frequency	-0.242	0.345	3.392	-0.512 *	0.267	2.786
Inpatient services Frequency	0.210	0.170	1.540	0.021	0.217	1.585
Laboratory tests Frequency	0.134	0.088	0.406	-0.099	0.093	0.505
Monoterapy Frequency	0.205 *	0.123	0.170	0.037	0.115	0.232
Polyterapy Frequency	-0.254	0.340	3.004	-0.716 *	0.421	3.243
Teletherapy Frequency	-0.084 *	0.044	0.259	-0.077	0.048	0.229
Radiotherapy Frequency	-0.134	0.370	3.434	-0.756 *	0.428	3.704
Emergency consultations frequency	-0.959 **	0.379	2.964	-0.562 *	0.288	2.288
Home care frequency	0.281	0.258	0.854	0.047	0.260	0.995
Capecitabina Rx frequency	0.093	0.141	0.332	-0.070	0.130	0.412
Morfina Rx frequency	0.060	0.140	0.510	0.103	0.101	0.329
Surgical consultation real expenditure (stand.)	25734.533 ***	5782.911	20319.727	18928.547 ***	5219.810	19011.732
Palliative care consultation real expenditure	1592.599	10097.638	35794.520	2908.289	6418.688	29618.854
Palliative care consultation real expenditure (stand.)	3524.809	10920.913	47169.660	1610.287	8026.010	37420.441
Radiology consultation real expenditure	-3347.677	3720.731	17137.602	-1889.546	2889.826	13209.222
Radiology consultation real expenditure (stand.)	409	3,414	14,909	-1,932	3,585	14,618
Follow-up consultation real expenditure	49,234	30,718	155,977	-913	18,039	158,220
Follow-up consultation real expenditure (stand.)	30,828	50,587	227,094	-84,112 **	38,938	282,199
Screening real expenditure	40,848	81,343	162,480	12,080	56,174	175,370
Screening real expenditure (stand.)	-2,277,708	1,444,528	2,576,031	-22,131	91,980	254,316

Spread procedures real expenditure	212,206	***	72,382	273,594	-101,218		91,797	453,688
Spread procedures real expenditure (stand.)	580,289	***	220,493	354,183	-259,490		430,702	1,080,207
Gastrectomy real expenditure	-652,922		900,703	3,987,324	1,106,035	**	552,285	2,249,574
Gastrectomy real expenditure (stand.)	376,322		1,304,335	4,683,602	18,912		1,184,246	4,811,353
Diagnostic imaging real expenditure	225,489	*	117,814	687,615	-145,418		105,171	793,267
Diagnostic imaging real expenditure (stand.)	140,554		89,306	886,023	-185,540	**	93,451	911,058
Inpatient services real expenditure	212,501		309,577	1,568,931	-77,815		293,728	1,733,702
Inpatient services real expenditure (stand.)	1,339,577	**	573,180	3,486,282	-1,277,049		1,143,528	5,893,066
Laboratory tests real expenditure	37,798	***	12,029	46,954	-5,279		16,587	82,519
Laboratory tests real expenditure (stand.)	63,584	***	23,250	72,150	-17,687		56,672	135,105
Monoterapy real expenditure	66,253	**	27,330	24,440	20,816		23,723	40,043
Monoterapy real expenditure (stand.)	374,951	*	206,941	71,805	143,460		172,693	113,405
Polyterapy real expenditure	-165,186		209,457	1,854,959	-626,339	*	365,720	2,822,092
Polyterapy real expenditure (stand.)	1,977,586	**	921,374	3,797,798	-1,710,123		1,443,144	6,448,038
Teletherapy real expenditure	-661,379	*	394,942	2,018,341	-298,168		371,230	1,509,973
Teletherapy real expenditure (stand.)	-530,538		400,860	2,042,560	-506,924		531,667	1,884,504
Radiotherapy real expenditure	-760,312		485,313	3,897,740	-903,691	*	496,594	4,372,108
Radiotherapy real expenditure (stand.)	1,821,999	*	1,095,868	5,912,164	-2,073,587		1,527,534	8,445,946
Emergency consultations real expenditure per person	-112,032	**	54,898	297,949	-15,948		48,184	199,528
Emergency consultations real expenditure per person (stand.)	-507,700	**	227,620	1,120,828	-238,944		151,381	758,318
Home care real expenditure per person	103,059		100,588	267,928	-14,744		99,709	330,904
Home care real expenditure per person (stand.)	275,412		235,845	623,755	12,748		242,846	810,587
Impatient care real expenditure per person	74,928		890,029	7,693,525	107,796		1,434,128	7,441,126
Impatient care real expenditure per person (stand.)	786,528		2,903,380	15,658,134	-1,098,409		2,369,315	15,992,606
Cancer treatment real expenditure per person	-15,079		231,416	2,145,813	-637,123	*	379,265	3,179,531
Cancer treatment real expenditure per person (stand.)	2,360,385	**	932,153	4,224,937	-1,601,697		1,463,479	6,876,870
Capecitabina Rx real expenditure per person	46,834		69,354	161,612	-31,468		63,475	199,257
Capecitabina Rx real expenditure per person (stand.)	-54,418		96,799	241,269	-68,311		63,747	214,036
Morfina Rx real expenditure per person	3,745		3,662	8,982	1,619		2,619	8,258
Morfina Rx real expenditure per person (stand.)	5,153		6,157	11,651	2,178		4,060	10,910
Real expenditure per person	2,047,354		3,680,734	30,787,084	-3,977,664		3,130,868	32,674,812
Real expenditure per person (procedures only)	3,072,299		3,467,024	25,178,504	-5,217,739	*	2,930,633	29,808,650
Real expenditure per person (drugs only)	-417,313		653,953	3,285,957	814,860	*	441,805	1,733,596
Real expenditure per person (stand.)	-527,385		1,197,570	17,493,660	-633,145		1,688,963	16,576,249
Real expenditure per person (stand., procedures only)	-438,436		999,601	15,098,923	-1,071,801		1,579,263	15,007,433
Real expenditure per person (stand., drugs only)	-54,175		361,714	2,186,539	428,699		280,914	1,460,606
Cancer care real expenditure per person	2,068,520		2,721,038	18,855,434	-3,939,575		2,396,441	22,406,876
Cancer care real expenditure per person (procedures only)	2,060,672		2,740,442	18,500,100	-3,904,630		2,407,594	22,091,242
Cancer care real expenditure per person (drugs only)	7,848		104,741	355,334	-34,945		83,728	315,633
Cancer care real expenditure per person (stand.)	-593,109		739,970	11,333,497	-201,969		923,974	10,620,768
Cancer care real expenditure per person (stand., procedures only)	-676,964		719,967	11,067,083	-169,793		923,066	10,302,648
Cancer care real expenditure per person (stand., drugs only)	83,854		75,002	266,414	-32,175		76,463	318,120

*Note: Each row reports the Average Treatment Effect on the Treated (ATT) comparing patients exposed to the COVID-19 period (2020–2021) with matched pre-pandemic patients in Colombia. Estimates are obtained using nearest-neighbor propensity score matching with replacement, implemented with the STATA module PSMATCH2 (Leuven and Sianesi, 2018). Outcomes include measures of service use (extensive and intensive margins) and real expenditure per patient. The "P.O. mean" corresponds to the mean outcome in the matched pre-COVID cohort. Robust standard errors are reported in parentheses. *, **, *** denote statistical significance at the 10%, 5%, and 1% levels, respectively.*

A5.2. State-level model

We found that mortality rates per 100,000 affiliates from the CAC data increased for all cancers except stomach. This increase ranged from 0.41 (lung) to 5.05 (prostate), representing an average rate increase of 35%. However, this increase became non-significant when using the mortality measure from Vital Statistics - DANE, which has better control over COVID-related deaths, suggesting that the increase in mortality might be explained by excess COVID-related mortality, which was more severe among age groups with higher cancer incidence. On the other hand, incidence decreased for most cancers (except cervical), with reductions between 20% and 31%, with the greatest decline in prostate and the smallest in lung cancer, averaging a 25% decrease. This could suggest that the health emergency reduced screening and diagnosis, leading to a systematic reduction in new cases. Finally, there was a significant increase in the prevalence of all cancers, with increases ranging from 15% (lung) to 37% (stomach), averaging 25%. This result should be interpreted cautiously, as the 2019 dummy variable shows significance in breast, prostate, stomach, and lung cancers, but the coefficients are relatively small compared to the pandemic period, averaging -3%, with lung cancer showing the largest decrease (-11%).

Table A5.2.1. Robustness checks: alternative specifications of the impact of COVID-19 on breast cancer outcomes in Colombia, state-level panel data, 2018–2021

Variables	(1) Log mortality rate - CAC	(2)	(3) Log mortality rate - EV	(4)	(5) Log new case reported rate- CAC	(6)	(7) Log prevalence rate- CAC	(8)	(9) Log timeliness of cancer care (days) - CAC	(10)	(11) Women diagnosed with advanced-stage (%)- CAC	(12)	(13) Latality rate advanced-stage (%) - CAC	(14)
Post-Covid indicator	5.09***	3.05***	1.03***	0.64	-0.16	-5.04**	85.33***	62.73***	0.88	2.10	5.59***	-0.73	1.05**	1.41
	(0.68)	(1.02)	(0.34)	(0.76)	(0.91)	(1.91)	(7.51)	(9.71)	(2.40)	(5.61)	(1.82)	(4.59)	(0.41)	(0.96)
year = 2019	-0.31	-0.56	0.61	0.46	-6.19***	-7.90***	8.27***	1.47	3.38	3.55	0.11	-2.85	-0.61	-0.61
	(0.25)	(0.35)	(0.39)	(0.39)	(1.36)	(1.21)	(1.62)	(4.02)	(2.29)	(3.31)	(1.53)	(2.28)	(0.38)	(0.53)
year = 2020	-3.24***	-2.10**	-0.20	-0.27	2.13***	5.41***	-31.20***	-4.57	4.16**	5.38	-4.93***	3.84	-0.79**	-0.42
	(0.45)	(0.76)	(0.38)	(0.89)	(0.67)	(1.78)	(3.45)	(11.77)	(1.58)	(5.35)	(1.25)	(4.10)	(0.38)	(0.98)
Barriers to healthcare access (index)		-0.07		0.02		-0.26		0.23		0.17		-0.15		0.06
		(0.06)		(0.06)		(0.22)		(1.05)		(0.40)		(0.26)		(0.11)
Multidimensional Poverty Index, women		0.04		-0.10		-0.30		-0.21		-0.20		-0.22		-0.05
		(0.09)		(0.09)		(0.23)		(1.78)		(0.59)		(0.43)		(0.19)
Share of patients diagnosed at advanced nodal stage (N2–N3)		-1.35		0.96		2.84		-9.11		-7.28		1.56		-1.32
		(0.89)		(1.04)		(2.43)		(12.60)		(4.30)		(5.03)		(1.27)
Number of prevalent cases (numerator)		0.00		0.00**		0.00				-0.00**		-0.00		-0.00*
		(0.00)		(0.00)		(0.00)				(0.00)		(0.00)		(0.00)
GDP per capita		0.00		-0.00		0.00		0.00**		0.00		0.00***		0.00
		(0.00)		(0.00)		(0.00)		(0.00)		(0.00)		(0.00)		(0.00)
Observations	108	108	108	108	108	108	108	108	108	108	108	108	108	108
R-squared	0.777	0.810	0.095	0.150	0.553	0.635	0.844	0.868	0.090	0.143	0.154	0.246	0.168	0.221
Number of Units	27	27	27	27	27	27	27	27	27	27	27	27	27	27
State FE	Yes	Yes	Yes	Yes	Yes	Yes	Yes	Yes	Yes	Yes	Yes	Yes	Yes	Yes
Year FE	Yes	Yes	Yes	Yes	Yes	Yes	Yes	Yes	Yes	Yes	Yes	Yes	Yes	Yes
Controls	No	Yes	No	Yes	No	Yes	No	Yes	No	Yes	No	Yes	No	Yes
Sample mean	11.68	11.68	14.42	14.42	22.80	22.80	265.1	265.1	69.61	69.61	54.16	54.16	7.296	7.296

*Notes: This table presents robustness checks for the main fixed-effects estimates of the impact of the COVID-19 period on cancer outcomes in Colombia. The unit of observation is a state-year, using panel data for 2018–2021. Alternative specifications include variations in control variables, sample restrictions, and model definitions to assess the sensitivity of the baseline results reported in Table 4. The post-COVID indicator equals 1 for years 2020–2021 and 0 for 2018–2019. All models include state and year fixed effects unless otherwise specified. Robust standard errors are reported in parentheses. Statistical significance is indicated as follows: * p<0.10, ** p<0.05, *** p<0.01.*

Table A5.2.2. Robustness checks: alternative specifications of the impact of COVID-19 on cervical cancer outcomes in Colombia, state-level panel data, 2018–2021

	(1)	(2)	(3)	(4)	(5)	(6)	(7)	(8)	(9)	(10)	(11)	(12)	(13)	(14)
Variables	Log mortality rate - CAC		Log mortality rate - EV		Log new case reported rate- CAC		Log prevalence rate- CAC		Log timeliness of cancer care (days) - CAC		Women with early-stage receiving curative treatment (%)- CAC		Women with advanced-stage receiving chemoradiation and brachytherapy (%) - CAC	
Post-Covid indicator	1.88***	2.36***	0.42	0.44	-0.98**	-0.98	14.72***	13.28***	-5.25	-4.54	18.02***	29.61***	39.05***	35.83***
	(0.28)	(0.66)	(0.26)	(0.45)	(0.37)	(0.94)	(1.63)	(3.14)	(3.55)	(7.61)	(5.14)	(9.70)	(2.73)	(4.83)
year = 2019	-0.02	-0.06	0.39*	0.54**	-1.43***	-1.65***	0.36	-0.41	0.92	-0.58	14.66***	16.33***	-5.35***	-4.48**
	(0.18)	(0.29)	(0.20)	(0.24)	(0.39)	(0.42)	(0.48)	(1.02)	(3.16)	(4.15)	(4.80)	(5.35)	(1.57)	(1.71)
year = 2020	-1.39***	-1.40**	-0.11	-0.57	1.66***	1.88**	-5.80***	-3.87	9.95**	10.46	6.73	1.83	-41.72***	-44.06***
	(0.25)	(0.63)	(0.26)	(0.51)	(0.29)	(0.90)	(0.72)	(3.27)	(3.64)	(8.35)	(4.66)	(9.93)	(3.04)	(5.73)
Barriers to healthcare access (index)		0.05		-0.02		0.04		0.19		0.18		0.46		-0.59
		(0.04)		(0.04)		(0.06)		(0.19)		(0.79)		(1.18)		(0.45)
Multidimensional Poverty Index, women		-0.07		0.08		-0.10		-0.45		-1.38		1.61		0.52
		(0.06)		(0.07)		(0.08)		(0.40)		(1.30)		(1.61)		(0.72)
Share of patients diagnosed at advanced nodal stage (N2–N3)		-0.05		0.79		1.29		-0.82		-1.02		28.40*		-0.19
		(0.65)		(0.75)		(1.16)		(2.58)		(12.07)		(15.74)		(7.45)
Number of prevalent cases (numerator)		-0.00*		0.00***		0.00				-0.01		0.01		0.02*
		(0.00)		(0.00)		(0.00)				(0.01)		(0.02)		(0.01)
GDP per capita		0.00		-0.00*		-0.00		0.00		-0.00		-0.00		-0.00
		(0.00)		(0.00)		(0.00)		(0.00)		(0.00)		(0.00)		(0.00)
Observations	108	108	108	108	108	108	108	108	108	108	100	100	107	107
R-squared	0.523	0.550	0.039	0.120	0.362	0.385	0.774	0.781	0.111	0.168	0.265	0.334	0.874	0.881
Number of Units	27	27	27	27	27	27	27	27	27	27	27	27	27	27
State FE	Yes	Yes	Yes	Yes	Yes	Yes	Yes	Yes	Yes	Yes	Yes	Yes	Yes	Yes
Year FE	Yes	Yes	Yes	Yes	Yes	Yes	Yes	Yes	Yes	Yes	Yes	Yes	Yes	Yes
Controls	No	Yes	No	Yes	No	Yes	No	Yes	No	Yes	No	Yes	No	Yes
Sample mean	4.647	4.647	7.136	7.136	6.604	6.604	64.66	64.66	76.66	76.66	46.52	46.52	14.49	14.49

*Notes: This table presents robustness checks for the main fixed-effects estimates of the impact of the COVID-19 period on cancer outcomes in Colombia. The unit of observation is a state-year, using panel data for 2018–2021. Alternative specifications include variations in control variables, sample restrictions, and model definitions to assess the sensitivity of the baseline results reported in Table 4. The post-COVID indicator equals 1 for years 2020–2021 and 0 for 2018–2019. All models include state and year fixed effects unless otherwise specified. Robust standard errors are reported in parentheses. Statistical significance is indicated as follows: * $p<0.10$, ** $p<0.05$, *** $p<0.01$.*

Table A5.2.3. Robustness checks: alternative specifications of the impact of COVID-19 on colorectal cancer outcomes in Colombia, state-level panel data, 2018–2021

	(1)	(2)	(3)	(4)	(5)	(6)	(7)	(8)	(9)	(10)	(11)	(12)	(13)	(14)
Variables	Log mortality rate - CAC		Log mortality rate - EV		Log new case reported rate- CAC		Log prevalence rate- CAC		Log timeliness of treatment (days) - CAC		New cases identified at early stages (%)- CAC		Stage I-III patients with curative surgery (%) - CAC	
Post-Covid indicator	2.05***	1.26***	0.28	0.13	0.13	-1.34**	15.30***	11.51***	17.42***	12.63**	-17.77***	-26.99***	16.18***	17.50**
	(0.21)	(0.30)	(0.17)	(0.50)	(0.36)	(0.59)	(1.98)	(1.90)	(1.97)	(5.05)	(2.73)	(5.93)	(3.01)	(7.72)
year = 2019	-0.27*	-0.29	0.18	0.16	-1.85***	-1.87***	-0.42	-1.40**	22.43***	19.87***	-3.63	-5.11*	11.63***	11.86***
	(0.13)	(0.17)	(0.19)	(0.24)	(0.34)	(0.37)	(0.33)	(0.64)	(2.41)	(3.37)	(2.38)	(2.54)	(2.68)	(2.84)
year = 2020	-1.29***	-0.88**	0.06	0.23	0.39	0.65	-6.56***	-1.27	4.28*	12.45**	3.57*	9.54**	3.05	0.92
	(0.18)	(0.33)	(0.16)	(0.37)	(0.67)	(0.56)	(1.04)	(1.39)	(2.23)	(4.82)	(1.77)	(4.21)	(2.14)	(4.52)
Barriers to healthcare access (index)		-0.04*		0.03		-0.09**		-0.07		-0.20		-0.54		-0.04
		(0.02)		(0.03)		(0.04)		(0.12)		(0.41)		(0.36)		(0.38)
IPM ajustado		0.06*		-0.04		-0.03		0.75***		0.29		-0.09		-0.10
		(0.03)		(0.04)		(0.08)		(0.23)		(0.67)		(0.52)		(0.82)
Share of patients diagnosed at advanced nodal stage (N2–N3)		-0.63		-0.09		-0.32		-3.14		-4.36		-8.66		6.44
		(0.45)		(0.46)		(0.85)		(2.41)		(9.09)		(7.00)		(8.87)
Number of prevalent cases (numerator)		0.00***		0.00		0.00***				-0.01		0.00		0.00
		(0.00)		(0.00)		(0.00)				(0.00)		(0.00)		(0.01)
GDP per capita		0.00		0.00		-0.00		0.00***		0.00**		0.00		-0.00
		(0.00)		(0.00)		(0.00)		(0.00)		(0.00)		(0.00)		(0.00)
Observations	108	108	108	108	108	108	108	108	108	108	108	108	108	108
R-squared	0.733	0.805	0.057	0.082	0.450	0.547	0.799	0.873	0.543	0.573	0.478	0.507	0.360	0.367
Number of Units	27	27	27	27	27	27	27	27	27	27	27	27	27	27
State FE	Yes	Yes	Yes	Yes	Yes	Yes	Yes	Yes	Yes	Yes	Yes	Yes	Yes	Yes
Year FE	Yes	Yes	Yes	Yes	Yes	Yes	Yes	Yes	Yes	Yes	Yes	Yes	Yes	Yes
Controls	No	Yes	No	Yes	No	Yes	No	Yes	No	Yes	No	Yes	No	Yes
Sample mean	4.644	4.644	8.076	8.076	6.012	6.012	43.68	43.68	56.04	56.04	35.85	35.85	40.24	40.24

*Notes: This table presents robustness checks for the main fixed-effects estimates of the impact of the COVID-19 period on cancer outcomes in Colombia. The unit of observation is a state-year, using panel data for 2018–2021. Alternative specifications include variations in control variables, sample restrictions, and model definitions to assess the sensitivity of the baseline results reported in Table 4. The post-COVID indicator equals 1 for years 2020–2021 and 0 for 2018–2019. All models include state and year fixed effects unless otherwise specified. Robust standard errors are reported in parentheses. Statistical significance is indicated as follows: * p<0.10, ** p<0.05, *** p<0.01.*

Table A5.2.4. Robustness checks: alternative specifications of the impact of COVID-19 on lung cancer outcomes in Colombia, state-level panel data, 2018–2021

Variables	(1)	(2)	(3)	(4)	(5)	(6)	(7)	(8)
	Log mortality rate - CAC		Log mortality rate - EV		Log new case reported rate- CAC		Log prevalence rate- CAC	
Post-Covid indicator	0.87***	0.41*	-0.98***	-0.62	0.04	-0.48*	2.10***	1.26**
	(0.23)	(0.23)	(0.20)	(0.40)	(0.23)	(0.23)	(0.63)	(0.57)
year = 2019	-0.43**	-0.22	-0.51**	-0.48*	-0.55***	-0.48**	-0.97***	-1.37***
	(0.17)	(0.13)	(0.24)	(0.28)	(0.16)	(0.18)	(0.22)	(0.35)
year = 2020	-0.46**	-0.18	0.81***	0.65	0.33***	0.71***	-0.97***	0.50
	(0.17)	(0.24)	(0.28)	(0.45)	(0.07)	(0.20)	(0.29)	(0.43)
Barriers to healthcare access (index)		0.02		0.00		-0.01		-0.02
		(0.02)		(0.04)		(0.02)		(0.04)
IPM ajustado		-0.01		0.06		0.01		0.19***
		(0.02)		(0.06)		(0.03)		(0.06)
Share of patients diagnosed at advanced nodal stage (N2–N3)		-0.11		0.64		0.53		0.64
		(0.39)		(0.75)		(0.33)		(0.88)
Number of prevalent cases (numerator)		0.00***		-0.00		0.00***		
		(0.00)		(0.00)		(0.00)		
GDP per capita		-0.00		-0.00		0.00		0.00**
		(0.00)		(0.00)		(0.00)		(0.00)
Observations	104	104	104	104	104	104	108	108
R-squared	0.499	0.713	0.186	0.206	0.342	0.632	0.572	0.683
Number of Units	26	26	26	26	26	26	27	27
State FE	Yes	Yes	Yes	Yes	Yes	Yes	Yes	Yes
Year FE	Yes	Yes	Yes	Yes	Yes	Yes	Yes	Yes
Controls	No	Yes	No	Yes	No	Yes	No	Yes
Sample mean	2.757	2.757	9.211	9.211	2.461	2.461	8.447	8.447

*Notes: This table presents robustness checks for the main fixed-effects estimates of the impact of the COVID-19 period on cancer outcomes in Colombia. The unit of observation is a state-year, using panel data for 2018–2021. Alternative specifications include variations in control variables, sample restrictions, and model definitions to assess the sensitivity of the baseline results reported in Table 4. The post-COVID indicator equals 1 for years 2020–2021 and 0 for 2018–2019. All models include state and year fixed effects unless otherwise specified. Robust standard errors are reported in parentheses. Statistical significance is indicated as follows: * p<0.10, ** p<0.05, *** p<0.01.*

Table A5.2.5. Robustness checks: alternative specifications of the impact of COVID-19 on prostate cancer outcomes in Colombia, state-level panel data, 2018–2021

Variables	(1) Log mortality rate - CAC	(2)	(3) Log mortality rate - EV	(4)	(5) Log new case reported rate- CAC	(6)	(7) Log prevalence rate- CAC	(8)	(9) Log timeliness of diagnosis (days) - CAC	(10)	(11) Log timeliness of treatment (days) - CAC	(12)	(13) Patients with advanced-stages (III-IV) (%) - CAC	(14)
Post-Covid indicator	6.92***	5.05***	1.13***	-0.10	-1.57*	-3.87***	66.90***	41.19***	-1.83	-3.06	32.75***	51.02***	10.32***	8.81
	(0.74)	(0.95)	(0.38)	(0.65)	(0.81)	(1.34)	(8.97)	(7.39)	(4.19)	(13.48)	(3.42)	(9.91)	(3.20)	(6.83)
year = 2019	-0.33	-0.19	0.91***	0.38	-2.09***	-2.84***	3.96***	-2.96	1.33	1.14	23.95***	29.51***	0.32	0.22
	(0.26)	(0.33)	(0.26)	(0.27)	(0.65)	(0.94)	(1.17)	(2.96)	(5.68)	(6.72)	(4.61)	(4.11)	(3.06)	(3.78)
year = 2020	-4.84***	-4.50***	0.32	1.63**	4.47***	6.24***	-27.47***	1.01	16.51***	14.30	-4.46	-14.22	-8.82***	-9.18
	(0.47)	(0.82)	(0.33)	(0.70)	(0.53)	(0.95)	(4.03)	(8.30)	(4.90)	(13.16)	(3.74)	(9.82)	(2.57)	(5.59)
Barriers to healthcare access (index)		-0.03		0.04		-0.02		0.08		-0.43		1.21*		-0.03
		(0.06)		(0.04)		(0.12)		(0.97)		(1.13)		(0.61)		(0.54)
IPM hombres		0.07		-0.10		-0.15		-0.13		-0.09		1.22		-0.56
		(0.11)		(0.07)		(0.16)		(1.43)		(1.45)		(0.96)		(0.80)
Share of patients diagnosed at advanced nodal stage (N2–N3)		-0.66		1.73*		1.92		-15.21		6.59		-11.20		-5.71
		(0.91)		(0.86)		(1.96)		(10.38)		(18.92)		(10.47)		(9.31)
Number of prevalent cases (numerator)		0.00***		0.00**		0.00**				0.00		-0.01**		-0.00
		(0.00)		(0.00)		(0.00)				(0.00)		(0.00)		(0.00)
GDP per capita		-0.00		0.00		0.00		0.00***		-0.00		-0.00		-0.00
		(0.00)		(0.00)		(0.00)		(0.00)		(0.00)		(0.00)		(0.00)
Observations	108	108	108	108	104	104	108	108	104	104	105	105	106	106
R-squared	0.821	0.890	0.226	0.369	0.538	0.594	0.808	0.852	0.135	0.146	0.461	0.539	0.179	0.198
Number of Units	27	27	27	27	26	26	27	27	27	27	27	27	27	27
State FE	Yes	Yes	Yes	Yes	Yes	Yes	Yes	Yes	Yes	Yes	Yes	Yes	Yes	Yes
Year FE	Yes	Yes	Yes	Yes	Yes	Yes	Yes	Yes	Yes	Yes	Yes	Yes	Yes	Yes
Controls	No	Yes	No	Yes	No	Yes	No	Yes	No	Yes	No	Yes	No	Yes
Sample mean	9.191	9.191	14.03	14.03	12.47	12.47	154.5	154.5	66.01	66.01	81.05	81.05	36.43	36.43

*Notes: This table presents robustness checks for the main fixed-effects estimates of the impact of the COVID-19 period on cancer outcomes in Colombia. The unit of observation is a state-year, using panel data for 2018–2021. Alternative specifications include variations in control variables, sample restrictions, and model definitions to assess the sensitivity of the baseline results reported in Table 4. The post-COVID indicator equals 1 for years 2020–2021 and 0 for 2018–2019. All models include state and year fixed effects unless otherwise specified. Robust standard errors are reported in parentheses. Statistical significance is indicated as follows: * p<0.10, ** p<0.05, *** p<0.01.*

Table A5.2.6. Robustness checks: alternative specifications of the impact of COVID-19 on stomach cancer outcomes in Colombia, state-level panel data, 2018–2021

Variables	(1)	(2)	(3)	(4)	(5)	(6)	(7)	(8)	(9)	(10)	(11)	(12)
	Log mortality rate - CAC		Log mortality rate - EV		Log new case reported rate- CAC		Log prevalence rate- CAC		Log timeliness of treatment (days) - CAC		New cases identifie at early stages (%)- CAC	
Post-Covid indicator	1.44***	0.51	-0.12	-0.37	-0.05	-1.18**	8.30***	7.59***	19.37***	11.15*	-14.76***	-15.43***
	(0.19)	(0.32)	(0.31)	(0.81)	(0.26)	(0.44)	(1.31)	(2.42)	(2.57)	(6.27)	(1.88)	(4.39)
year = 2019	-0.64***	-0.37	0.06	0.02	-1.17***	-1.11***	-1.53***	-1.20*	28.14***	29.99***	-3.64	-5.02*
	(0.22)	(0.22)	(0.20)	(0.26)	(0.26)	(0.23)	(0.33)	(0.70)	(3.08)	(4.14)	(2.31)	(2.59)
year = 2020	-1.13***	-0.98***	-0.02	0.13	0.40	0.95**	-5.99***	-5.64*	9.26***	12.50*	0.44	2.11
	(0.21)	(0.31)	(0.19)	(0.56)	(0.25)	(0.38)	(1.30)	(2.76)	(2.59)	(6.20)	(1.33)	(3.91)
Barriers to healthcare access (index)		-0.04		-0.01		-0.05		-0.03		-0.03		-0.36
		(0.04)		(0.03)		(0.03)		(0.10)		(0.45)		(0.27)
IPM ajustado		-0.03		-0.02		-0.04		0.24		-0.52		0.35
		(0.06)		(0.08)		(0.06)		(0.18)		(0.66)		(0.42)
Share of patients diagnosed at advanced nodal stage (N2–N3)		-1.43**		-0.22		-0.16		-4.58		-4.97		10.62
		(0.59)		(0.76)		(0.51)		(2.86)		(9.24)		(8.28)
Number of prevalent cases (numerator)		0.00**		-0.00		0.00**				0.03***		0.00
		(0.00)		(0.00)		(0.00)				(0.01)		(0.01)
GDP per capita		-0.00		0.00		-0.00		0.00		-0.00		0.00
		(0.00)		(0.00)		(0.00)		(0.00)		(0.00)		(0.00)
Observations	108	108	108	108	108	108	108	108	108	108	108	108
R-squared	0.553	0.641	0.012	0.019	0.411	0.506	0.540	0.559	0.537	0.561	0.464	0.490
Number of Units	27	27	27	27	27	27	27	27	27	27	27	27
State FE	Yes	Yes	Yes	Yes	Yes	Yes	Yes	Yes	Yes	Yes	Yes	Yes
Year FE	Yes	Yes	Yes	Yes	Yes	Yes	Yes	Yes	Yes	Yes	Yes	Yes
Controls	No	Yes	No	Yes	No	Yes	No	Yes	No	Yes	No	Yes
Sample mean	4.267	4.267	10.75	10.75	4.057	4.057	20.25	20.25	48.75	48.75	23.11	23.11

*Notes: This table presents robustness checks for the main fixed-effects estimates of the impact of the COVID-19 period on cancer outcomes in Colombia. The unit of observation is a state-year, using panel data for 2018–2021. Alternative specifications include variations in control variables, sample restrictions, and model definitions to assess the sensitivity of the baseline results reported in Table 4. The post-COVID indicator equals 1 for years 2020–2021 and 0 for 2018–2019. All models include state and year fixed effects unless otherwise specified. Robust standard errors are reported in parentheses. Statistical significance is indicated as follows: * p<0.10, ** p<0.05, *** p<0.01.*
